# Supplementary material for: Dynamic assessment of the effectiveness of digital game-based literacy training in beginning readers: a cluster randomised controlled trial
Source: PeerJ. 2023 Jul 31;11:e15499. doi: 10.7717/peerj.15499 (PMC10399564; doi:10.7717/peerj.15499)
Supplement: Supplemental Information 1 — This appendix gives an in-depth description of the design and materials of the Dutch version of GraphoGame used for the present research. More precisely, two different versions of the game were conceived: One intended for supporting the development of early literacy skills (GG-NL) and an accompanying math version (GG-NL-Math) which was meant to be an active control condition. While some information about the GraphoGame method in general, as well as the available game modes apply equally to both versions, the focus is clearly on providing scientific background and discussing game content and level design choices for the reading variant. [file peerj-11-15499-s001.pdf]

Appendix 1: Design of the Dutch GraphoGame

Contents

List of Tables **1**

List of Figures **2**

1 Introduction **3**

2 Linguistic factors **3**

2.1 Properties of Dutch 3

2.2 Dutch in comparison to other European orthographies 4

2.3 Current teaching methods 5

3 The GraphoGame method **6**

3.1 Mathematics DGBL 8

4 The Dutch GraphoGame **10**

4.1 Game mechanics 10

4.2 Game modules 10

4.3 Gaming tasks 27

4.4 In-game assessments 29

4.4.1 Literacy assessments 29

4.4.2 Arithmetic assessments 30

4.4.3 Assessment order and time limits 31

4.5 Training material 32

4.6 Level design 38

References **39**

List of Tables

1 Content of the easy letter knowledge assessment. 32

2 Content of the difficult letter knowledge assessment. 32

3 Content of the written lexical decision assessment. 32

4 Content of the number knowledge assessment. 33

5 Order and duration of in-game assessments. 33

6 Training content in alphabetical order. 34

7 GG-NL trainig content in sequential order. 35

List of Figures

1 The character selector. 12
2 The labyrinth map. 12
3 The trial game. 13
4 The flow game. 13
5 The star game. 14
6 The balloon game. 14
7 The pirate game. 15
8 The fishing game. 15
9 The ladder game. 16
10 The frog game. 16
11 The duel game. 17
12 The racing game. 17
13 The quiz game. 18
14 The word forming game. 18
15 The blending demo. 19
16 The counting task. 19
17 The number knowledge and neighboring number task. 20
18 The number sorting task. 20
19 The addition task. 21
20 The subtraction task. 21
21 The quiz addition task. 22
22 The easy letter-sound identification assessment. 22
23 The difficult letter-sound identification assessment. 23
24 The written lexical decision assessment. 23
25 The number knowledge assessment. 24
26 The biggest dot assessment. 24
27 The counting assessment. 25
28 The bigger number assessment (single digit). 25
29 The bigger number assessment (double digit). 26
30 The missing number assessment. 26
31 Frequency distribution of syllable structures. 33
32 Frequency distribution of graphemes. 36
33 Frequency distribution of syllable onsets, nuclei and codas. 37

1 Introduction

This appendix[1] gives an in-depth description of the designs and materials of the Dutch version of GraphoGame used during our research. More precisely, two different versions of the game were conceived: One intended for supporting the development of early literacy skills (GG-NL) and an accompanying math version (GG-NL-Math) which was meant to be an active control condition within a randomized control trial. While some information about the GraphoGame method in general, as well as the available game modes apply equally to both versions, the focus is clearly on providing scientific background and discussing game content and level design choices for the reading variant.

2 Linguistic factors

To create an effective learning tool for Dutch, we first need to establish some terms regarding orthographic transparency, grain size and syllable structure. Transparency refers to the regularity of grapheme to phoneme correspondences in a given orthography. This can be seen as a continuum ranging from transparent or shallow languages with (almost) one-to-one correspondences between phonemes and graphemes (e.g. Finnish) to highly irregular or deep orthographies (e.g. English). In comparison to shallow orthographies, reading accuracy develops slower in deeper orthographies (Ziegler & Goswami, 2005). The psycholinguistic 'grain size' theory (Goswami, 2002) proposes a variation in the chunking of written language as a strategy to adapt to the orthographic properties of the target orthography. Finnish for instance only requires small grain size (single grapheme to phoneme mapping is enough to decode all words) while English requires the use of different grain sizes (phoneme, rime, onset, word). For this reason, children need more time to learn to fluently read and write in English than in Finnish. Another factor in the early reading acquisition process is the syllable structure which depends on the phonotactic characteristics of a language. The latter determine the number of legal syllables which can differ drastically across languages. We can assume that a less complex syllable structure, particularly at the syllable onset, offers an advantage for early reading acquisition, as it reduces the 'granularity' problem (Ziegler & Goswami, 2005), and thus the number of correspondences at the syllabic level that needs to be automatized. The way in which the complexity of syllable structure in the spoken language determines reading acquisition, depends on the transparency. Syllabic complexity has only a minor influence on early reading acquisition in a transparent script. If the orthography is opaque, (concerning especially the rimes in English) the granularity problem is an obstacle for early acquisition. Behaviourally, readers of languages with more complex syllable structures are on par in terms of reading accuracy compared to languages with simpler syllable structures, but they are usually slower in pseudoword reading (Seymour, Aro & Erskine, 2003).

2.1 Properties of Dutch

Historically, Latin script was used to represent the sounds of Dutch dialects. Since no one to one correspondence could be found for some sounds - especially for the large number of Dutch vowels, digraphs were used instead (e.g. ⟨ae⟩ or ⟨oi⟩). The current standardized Dutch orthography was established as official spelling in 1954 (Reitsma & Verhoeven, 1990). In Dutch, as in all alphabetical languages, the graphemes correspond to phonemes (distinctive sounds). Dutch orthography makes use

[1] This document is intended as supplementary material for the article *Glatz, T., Tops, W., Borleffs, E., Richardson, U., Maurits, N., Desoete., A., & Maassen, B. A. M. (2018). Dynamic assessment of the effectiveness of digital game-based literacy training in beginning readers. PeerJ.*

of the 26 letters of the roman alphabet to represent around 35 different phonemes. The four graphemes ⟨x⟩, ⟨c⟩, ⟨q⟩ and ⟨y⟩ only occur in words of Latin origin, but apart from some additional exceptions in the form of e.g. ⟨b⟩, ⟨d⟩, ⟨g⟩, consonant graphemes have an almost one to one correspondence to phonemes. In addition, complex graphemes (e.g. ⟨ee⟩, ⟨aa⟩, or ⟨ie⟩) and consonant clusters (e.g. ⟨dr⟩ or ⟨tw⟩) are quite common in high frequent words (Aro & Wimmer, 2003), for instance in numbers twee ('two'), drie ('three') or twaalf ('twelve').

The translation conventions used by alphabetical writing systems go both ways: from grapheme to phoneme when reading, but also from phoneme to grapheme when spelling. In Dutch there are several spelling principles (Reitsma & Verhoeven, 1990). Firstly, according to the phonological principle a word is spelled as perceived like in *vis* (/vɪs/ 'fish') to realize transparent one to one correspondences between the graphemes and phonemes of the word. This is the easiest approach to spelling, but it does not allow to spell out irregular words. Secondly, there is the morphosyntactic rule of uniformity, which states that words that derive from the same root should be spelled the same way, even if they are pronounced differently (e.g. due to final devoicing: *hand* (/ɦɑnt/, 'hand') vs. *handen* (/'ɦɑndə(n)/, 'hands')). Furthermore, there is the rule of analogy which targets homophones, which are pairs of words that sound the same (e.g. /'ɣroːtə/) but should be distinguishable in their written form (e.g. *grootte* ('size') vs. *grote* ('big')). Additionally, there is a principle of etymology which suggests that loan words retain their original spelling (e.g. *chauffeur* (/ʃo.ˈføːr/, 'driver'). The latter three principles introduce additional layers of complexity, thus making the Dutch orthography more irregular. As it was previously suggested that children take slightly longer to master reading and spelling in more irregular writing systems, such delays should also apply to Dutch (Seymour et al., 2003).

2.2 Dutch in comparison to other European orthographies

In a work classifying 13 European orthographies by syllabic complexity and orthographic depth (Seymour, et al., 2003), Dutch was classified as having a complex syllable structure and medium orthographic depth. Sitting in the middle of the continuum between languages with deep and shallow orthographies, Dutch is not as transparent as Finnish, Spanish or Italian but still more regular than for instance English or French. In another work comparing properties of English, French, German, and Dutch, the latter was characterized as having the highest proportion of rime and consonant neighbour words, but the lowest number of onset-vowel neighbours (Ziegler & Goswami, 2005). These structural classifications are also supported by experimental evidence. As expected for a language with a complex syllable structure, Dutch first graders showed an exaggerated lexicality effect with pseudowords being read less accurately and slower in comparison to their European peers (Seymour et al., 2003). Overall reading speed was also noticeably slower, with Dutch children showing the slowest median reading time among seven European languages at the end of the first grade but catch up with comparable languages in that regard towards the end of 2nd grade (Aro & Wimmer, 2003; Coenen, von Bon, & Schreuder, 1997). Summing up, from previous research we can get the impression that even though Dutch children are trailing their peers from other European languages in terms of reading speed, by the end of 1st grade Dutch children can decode basic pseudo-words with a high accuracy. Considering that Dutch has a complex syllable structure, and a medium orthographic depth with some one-to-many correspondences, current teaching methods seem to be quite successful, achieving high reading accuracy early on.

2.3 Current teaching methods

There are different theoretical views on how to teach to read. Most commonly phonics instruction (Duncan et al., 2013), i.e. teaching the smallest units first, is thought to be most effective for English (Hulme, Hatcher, Nation, Brown, Adams, & Stuart 2002; Seymour & Duncan, 1997). Steps in this process are i) to form a representation of the units of written language, ii) to attach them to their corresponding sound (letter knowledge) and iii) to automate the processing of these connections. Only then it makes sense to broadly apply the alphabetical principle and to combine items into syllables and words (Richardson & Lyytinen, 2014). Given that English has a very complex syllable structure, there are also competing views which suggest that learning rime families is more useful in teaching to read English (Goswami & Bryant, 1990; Kyle et al., 2013). Transparent orthographies such as Finnish can be mastered by exclusive teaching (and learning) of grapheme to phoneme correspondences. Once children know how to sound out individual letters, they can decode any word they encounter. From then on it becomes a matter of speeding up this process and reaching the state of whole word reading. In less transparent languages like Dutch or English, the exclusive teaching of grapheme to phoneme correspondences is not sufficient. In the case of English it may even be counter-productive. Teaching a child that the letter ⟨a⟩ is pronounced as /ɑː/ may not be very effective because there are four different pronunciations of ⟨a⟩ in English. In less transparent languages a bigger role in the process of becoming a fluent reader is attributed to phonological awareness (PA) and rapid automatized naming (RAN), which are both precursors of fluent reading. These cross-linguistic differences are also reflected in the onset age of formal reading instruction, which is earlier in deep languages (five to six years of age in the UK and The Netherlands) and later in more transparent languages (seven years of age in Finland). While full maturation of reading skills continues throughout elementary school (Vaessen & Blomert, 2010), at around seven years of age the foundation of literacy development is considered to be completed.

In the Netherlands children usually enter elementary school at the age of 4, but they don't get any formal reading education in the first two years. However, there are some language games which are played and the basics of written language (e.g. reading from left to right, and from top to bottom), as well as the spelling of one's own name are introduced (Broekhof, 2006). Formal reading instruction then starts in the 3rd school-year (which corresponds to 1st grade in most other countries) at an average age of 6 years and 5 months. Veilig Leren Lezen (VLL; Mommers, Verhoeven, & Van der Linden, 1990) is one of the most common teaching methods used in the Netherlands. It is a word-based approach using minimal pairs to demonstrate the correspondences between written and spoken language. VLL uses a multi-modal principle that trains visual, auditory and articulatory skills in isolation and in combination. The first words are embedded into a (reading) story, supported by pictures. Children have to do tasks in a workbook such as discriminating words and matching them to pictures, or tasks for letter/word copying or filling in missing graphemes with the help of foldable structureerblaadjes ('structure sheets') which divide words into phonemes like *m/aa/n* ('m|oo|n'). Often continuant phonemes are used at the beginning (not stop consonants), because they can illustrate the word prolonged (mmmm|aaaaaa|nnnn). The first half of VLL is covered within the first 4-5 months of school, which should give all children the ability to decode regular monosyllabic words, an aim which we shall take over for GG-NL below as well! Using this teaching method children should advance quickly from decoding on a letter by letter basis to bigger clusters of reoccurring graphemes. Overall, VLL teaches word analysis skills, grapheme-phoneme correspondence and blending procedures (Reitsma & Verhoeven, 1990). In comparison to other Dutch teaching methods VLL fares well. In a study in which

two groups were compared, one learning with VLL and the other with Letterstad (Kooreman & Franken 1976), the children who were taught with VLL showed a more coherent learning curve (smaller group SD) and after ~20 weeks all children could read short regular words, while with Letterstad learning curves were less consistent (Bus, 1990; for further teaching methods also see Kanselaar, Vermeulen, & Kok, 1998).

3 The GraphoGame method

In recent years, digital game-based learning (DGBL) gained attention for literacy acquisition and intervention research due to a number of attractive characteristics. Arguably most important is the multimodal nature of learning (Potocki, Ecalle, & Magnan, 2013) and the ability to adapt to each players' special needs. Due to their playful nature, DGBL may further be more motivating for struggling readers compared to books (Ryan & Deci, 2000). They can also be installed on a smartphone, tablet or computer, allowing learning sessions outside of the classroom or away from home. As a side effect, compared to a single longer reading session at school, they enable researchers to deliver more frequent, yet shorter sessions, which is thought to maintain higher levels of concentration and interest and yield better learning outcome (Richardson & Lyytinen, 2014). DGBL may therefore be used in a wide range of settings, for instance training pre-school children, complementing the classroom curriculum, or provide extracurricular reading instruction or practice for poor readers. This can even be taken one step further by using such a tool for nominating children in need of additional tuition, while at the same time minimizing the time effort for the school staff. Apart from differences in training goals there may also be a division by economic region. In developed countries, the focus of the game is usually on remediation, early detection, and additional exposure to print. Whereas in developing countries, DGBL can provide reading instruction to children which may otherwise not be available due to social, political or geographical factors (Richardson & Lyytinen, 2014). Finally, such games offer advanced behavioural measures compared to traditional classroom teaching or pencil and paper testing. They collect data on confusability counts of certain sounds, the time needed to learn reading direction (both in: Kujala, Richardson & Lyytinen, 2010), a classification of committed errors, and of course response times (Gros, 2007; this work). Complementing such qualitative benefits for data generation, there is also a quantitative side: with DGBL much more data can be collected from each individual, and from much bigger groups. In combination, data gathered during DGBL may have a diagnostic value without being a reading assessment per se.

One great example for a literacy DGBL tool which combines all these features is GraphoGame (GG). GraphoGame offers a child friendly computerized learning environment which was developed at the University of Jyväskylä in Finland (for review, see Richardson & Lyytinen, 2014). In the very transparent Finish orthography the letter knowledge (LK) at the end of kindergarten is the most consistent predictor for later reading failure (Puolakanaho, et al., 2007), so the game started off as a simple tool to train this skill (Lyytinen, Ronimus, Alanko, Poikkeus, & Taanila 2007). To enable the use of GraphoGame for less transparent languages, the game evolved to also train PA skills such as blending, spelling and rhyming. Ultimately, it can now be used to train pre-literacy skills such as LK and PA, as well as reading fluency itself (e.g. Huemer, Landerl, Aro, & Lyytinen, 2008) and has been implemented in a wide range of languages. Considering the empirical evidence for long-term development of reading fluency, GraphoGame proved to work well for Finnish (Saine, Lerkkanen, Ahonen, Tolvanen, & Lyytinen, 2010; 2011), but there is less clear evidence from studies in

orthographies such as Polish (Kamykowska, Haman, Latvala, Richardson, & Lyytinen), Greek (Ktisti, 2015), German in Austria (Huemer et al., 2008) and Switzerland (Brem et al., 2010), Chilean Spanish (Rosas, Escobar, Ramírez, Meneses, & Guajardo, 2017), UK English (Kyle, Kujala, Richardson, Lyytinen, & Goswami, 2013), French (Ruiz, Lassault, Sprenger-Charolles, Richardson, Lyytinen, & Ziegler, 2017), Nyanja in Zambia (Ojanen, Kujala, Richardson, & Lyytinen, 2013, Ojanen et al., 2015), Bahasa in Indonesia (Borleffs, Glatz, Daulay, Richardson, Zwarts, & Maassen, 2017), and ultimately Dutch (Blomert & Willems, 2010).

It's well worth re-considering one of the traits of digital game-based learning (DGBL) tools like GraphoGame which make them so appealing for research: They are very flexible tools that can be adapted to work for different languages, age groups, training goals, feedback types and instruction methods ranging from a very explicit to very implicit instruction. However, exactly this flexibility makes it difficult to compare such studies to one another, because there is not one single version of GraphoGame that is translated to new languages, but rather for each study a new version is made and tailored to a specific use case and population like described above. Comparing across different DGBL tools may even be more challenging because of the different game and content design philosophies. Thus, one of the biggest obstacles for comparing studies, and for evaluating whether the observed differences in effectiveness can be attributed to cross-linguistic or rather methodological differences, remains a lack of transparency about the actual training content and tasks. To improve on this situation, the remainder of the present work makes a first step by sharing the specific use case, the approach to stimulus selection and level sequence design, and a detailed presentation of the resulting features of, and tasks within, GG-NL.

The sole previous attempt for adapting GraphoGame to Dutch was not successful but gave valuable insights for the development of the current version (Blomert & Willems, 2010). Being one of the few studies that trained children at familial risk for dyslexia (FR) before entering school during the last year of kindergarten, their game covered the reading materials from the entire first grade in around 80 levels. However, the difficulty increased rapidly, presenting minimal pairs, e.g. *rak* ('rack') vs. *ran* ('ran'), in level four, and complex graphemes (⟨ui⟩ ⟨eu⟩ ⟨ij⟩ ⟨ie⟩ and ⟨ou⟩) already in level nine, both potentially seen within the very first playing session[2]. Ultimately, the authors found that most FR children were training resistors, which indicates that their version of the game was not suitable for their use case. There are different perspectives to take on this: i) the content was too difficult for the developmental state of the high-risk sample, ii) the content was fine, but the training too early, or iii) the game did not give explicit enough instructions to cater for the needs of their high-risk population.

Based on these insights, we decided to create a version primarily aimed at first graders (six to seven years of age) and secondarily for slightly older children who had to repeat first grade, or who remained illiterate even after extensive instruction. This way we could assume that all children who play the game get concurrent formal reading instruction at school, which would allow us to make the game rather implicit, without too much explicit instruction. We also wanted to make the content less complex than in the previous version, meaning a level sequence with a gentler increase of difficulty, as well as not training material which children do not come across during the first months at school (e.g. overly complex multi-letter diphthongs like ⟨eeuw⟩ or ⟨oei⟩. The training goal was in alignment with the reading curriculum goal to enable children the decoding of regular monosyllabic words, which Dutch children usually achieve after half a year of reading instruction at school. Consequently, the

[2]This information is not part of the original publication nor publicly available but was provided upon personal communication with the creators of the game.

game should initially focus on improving accuracy so that progression through the game content should only be possible if predefined accuracy levels are reached. Only once children proved to maintain high levels of accuracy for a given subset of content, the game should also put a focus on speed and make use of timed levels so that for decent readers who move through the content with ease both accuracy and speed become a criterion to proceed.

To control the training environment and assure that children get equal opportunity to play 10-15 minutes on a daily basis, we also envisioned the game to be used at schools, embedded into the curriculum by the respective classroom teachers. This means that the game should complement formal reading instruction and that the content and sequence of GG-NL should to some extend mirror and complement what is happening in class. This should not imply that we merely aimed to computerized the books used for teaching, but that the content and ordering of the teaching material from elementary schools was one of many factors for the development of GG-NL. In summary, GG-NL is not meant to replace or imitate classroom instruction, as it does not cover important parts of reading acquisition, such as proper instructions, (hand-) writing, reading aloud, nor text reading. It is mostly intended as a tool to provide additional exposure to relevant reading materials (at phoneme and single word level). Research from the pre-digital era suggests that struggeling readers in fifth grade read up to 12 times less compared to average readers (Anderson, Wilson, & Fielding, 1988). Increased exposure may thus be especially important for poor performers, for whom an initially mild delay may further manifest due to lack of exposure to print. At the same time, we can investigate why sheer exposure works for some, but not for all poor performers. The resulting core functions of our envisioned game for the present work are therefore i) ensuring letter sound knowledge, ii) enhancing letter-to-speech-sound processing and in particular its automation iii) training PA skills at the level of blending and spelling, and iv) providing extended exposure to print. Before we go into the content, design and features of our reading game, we take a quick look at using GraphoGame for teaching of arithmetic skills.

3.1 Mathematics DGBL

Being a DGBL tool, the content within GraphoGame is not necessarily limited to reading material but can also cover other educational domains like mathematics. When investigating the effectiveness of GraphoGame with a randomized controlled trial (RCT), it is crucial to also have an appropriate control condition (All et al., 2014). To disentangle the motivational effects of gaming per se, it is imperative to include both a passive group as well as another active gaming group that plays a game without the literacy training. In many ways, a math version of GraphoGame can be considered a perfect active control condition as the training environment and tasks are comparable and only the type of content differs. Several previous studies investigated computerized arithmetic training around the onset of formal instruction at school. As these works inspired the design of the math game which was created within the scope of the present research, they are briefly presented below.

Wilson and colleagues (Wilson, Revkin, Cohen, Cohen, & Dehaene, 2006) designed the Number Race (NR). Within their study they trained 22 French children in an age range of 7 - 10 years and found increased speed for subitizing and enhanced subtraction accuracy after training. There was no change in the speed of object counting for four or more objects though. It is important to note that there was no control group in this study. Subsequently, Räsänen and colleagues (Räsänen, Salminen, Wilson, Aunio, & Dehaene, 2009) conducted a study with 60 Finnish preschool children aged 6.5 years comparing the effectiveness of NR to a version of GraphoGame with math content. Incidentally, the paper does not report on specific training content of the GraphoGame math version that was used.

But it can be inferred that at least number knowledge (NK), counting and magnitude comparison were part of the training. The study featured a three-week computerized training of five times 15 minutes per week for a total of three to four hours of game exposure. In a number comparison task, children who played GraphoGame Math improved over untrained controls, while the NR children did not improve significantly. Training with the NR game did show significant gains in response times for big differences between numbers though (e.g. numbers two vs. eight), but not for small differences (e.g. numbers four vs. six).

Further support for the effectiveness of computerized math interventions comes from a study conducted with normal developing children and children at risk of dyscalculia (based on low early numeracy) in kindergarten (5.8 years of age) in Belgium (Desoete & Praet, 2013; Praet & Desoete, 2014). During a five-week training phase these children played nine sessions of 25 minutes each, totaling four hours of exposure. Children were assigned to a game that trained either counting objects, comparing amounts, or to a control group without training. The training was implemented as a kind of preventive class, where children got feedback on their skills. There was no change on a number line estimation task but overall arithmetic skills improved. There was a persistent effect of gaming 6 months after the training and the counting game lead to bigger improvements. In conclusion, a computerized counting intervention at kindergarten age seems to be effective and have a long-term effect on mathematical development.

For our study, the math game was intended as an active control condition and was created from the ground up for this purpose. It was built upon the same framework as the GraphoGame reading game. Thus, featuring a range of identical reactive/interactive mini-games with varying graphics and task demands but in this case the level content consisted of number/digit knowledge, counting, comparison of numbers and amounts, sorting of adjacent or nonadjacent numbers in ascending or descending order, as well as simple addition and subtraction with numbers up to 20. Because of its role as active control condition, its effectiveness was not evaluated in detail and is beyond the scope of this dissertation. The math game is nevertheless an interesting project by itself, because at first look, the phenomenon of dyscalculia seems to mirror the complex of developmental dyslexia in the domain of numbers, amounts and calculations. The prevalence numbers are similar, with around 4 to 7% of children showing a selective inability in arithmetic while other cognitive functions are typically developed (Landerl, Fussenegger, Moll, & Willburger, 2009). In addition, the latest Programme for International Student Assessment (PISA, OECD, 2014) indicates that 14.8% of 15 year-olds in the Netherlands are not sufficiently proficient in mathematics, with 3.8% of these being unable to do any tasks involving numbers. The underlying cause for these problems differs between individuals. It may arise from difficulties in controlling working memory, from visuo-spatial working memory or a deficit in quantity representation (Dowker, 2005). Language and communication impairments frequently co-occur with dyscalculia and, unsurprisingly, the failure to become proficient in mathematics poses a similar danger for future education and emotional wellbeing as dyslexia does. A detailed description of math teaching methods is beyond the scope of this dissertation, and there is a wealth of research available for further background information (e.g. Baker, Gersten, & Lee, 2002).

4 The Dutch GraphoGame

4.1 Game mechanics

Upon starting the game, children are greeted by a pelican, which pops up throughout the game to give instructions and explain the tasks. In the first session children have to pick a character which serves as their login together with their first name (Figure 1). They are also reminded to always wear headphones when playing the game. Once inside the game, children can move their avatar around a labyrinth consisting of connected squares to find a way to a door which leads to a subsequent labyrinth. Along the way, they can encounter one of many mini games which offer training, empty squares, or items which can be used to customize the player's avatar (e.g. hats, funny glasses, or shoes). The collectable items offer additional opportunities to read as they come with properties like style or armor (see Figure 2). Upon entering a level (the different types of mini games will be detailed below) children need to solve a sequence of tasks. By default, each target is repeated three times within a level, so a level with five targets will contain 15 trials and take around one minute on average to complete. In practice, this allows for 6 to 10 mini games per playing session of 10 to 15 minutes. Most levels start easy, only showing one target and one distractor on the screen, but the number of distractors increases by one after a correct response and decreases by one after a wrong response. To successfully pass a level, 80% of trials need to be answered correctly (e.g. 12 out of 15 trials). However, an additional constraint requires that each target was at least once correctly responded to (i.e. being 100% correct on four out of the five targets to reach the 12 out of 15 is not sufficient, because it shows that the child still needs to learn the remaining target). There is positive reinforcement implemented into the game such that after a wrong response all written distractors disappear and only the target remains on the screen. In addition, the child will also hear the target again and is required to click on the sole remaining (correct) item on the screen. This is supposed to strengthen the speech-text associations and enhance learning, but also to implicitly explain what the goal of the task is, respectively was.

4.2 Game modules

The game features a wide range of game types (or themes) which even when the training content is kept constant contain different challenges in themselves. Here, we present an overview of the used game modules and highlight their specific features.

Standard game

There are a couple of level themes which behave identically even though their visual appearance differ. Within the game these modules are called Trial, Flow and Star game (Figures 3 through 5). Here, we group them together and entitle them standard game. This is the most prototypical form of the game where a target is presented auditorily and several bubbles are presented on the screen containing the correct target among one to six distractors. By default, these levels do not have a time limit (but one can be set) and the responses choices are fixed in their position on the screen and don't move around.

Balloon and pirate game

These levels start off with a big number of bubbles or objects which are reduced as the level proceeds towards the end (Figures 6 & 7). Noticeably, not all bubbles contain distractors from the start, but are increasing or decreasing in number according to the accuracy of the child on a trial by trial basis.

Furthermore, the bubbles with targets are slightly moving around, which makes them more difficult to read.

Fishing game

In the fishing game (Figure 8 on page 15), the words are projected onto fish in a lake and the player can catch them with a fishing tackle. The task is visually more demanding than the previous game modules because the fish are swimming around and the words thus move horizontally from left to right or right to left over the screen.

Ladder game

This game consists of a range of ladders and children control a ghost which goes up or down the ladder depending on whether a response is correct or incorrect (Figure 9 on page 16). Once they made it to the top, there is a bell which they can ring. The special feature of this game module is that there are at most 2-3 ladders per platform, which reduces the maximum number of distractors to one or two at a time, even when a child is always correct. This makes this module ideal to start teaching new associations or using it for more difficult content.

Frog game

The frog game involves a frog jumping from one screen to the next, and on each screen, there are multiple targets hidden among a big number of distractors of the same type (Figure 10 on page 16). When contrasting visually similar targets such as m/n or oe/ou this game module has a very high visual task load and is probably the only part of the game which is even challenging for adults. As a funny element for children, the frog catches the selected items with its tongue and swallows them. After a short pause the frog will spit out any wrong selections into the pool of distractors again.

Duel and race game

These two game modules have a built in time limit to promote faster responses. A too slow response is considered an error, so these levels are best used in later stages of the game with content where an accuracy close to 100% can be expected. In the duel game the player will see an opponent who slowly raises a water pistol to splash the player if he did not respond fast enough (Figure 11 on page 17). If the player responds faster though, he can splash the opponent. Similarly, in the race game there are race cars on the track which slowly move out of view (Figure 12 on page 17). The player needs to respond before they disappear out of sight.

Quiz game

The quiz game presents nine words on the screen and asks the player to either select real words among pseudowords, or to select a group of words according to some criterion (e.g. all animals) among other words or pseudowords (Figure 13 on page 18). In theory, children must read through all alternatives to decide which ones are correct. There are versions with and without time limit implemented in the game. If a predefined time is set, then new words come up until the time is up, otherwise there is a fixed number of targets which need to be found.

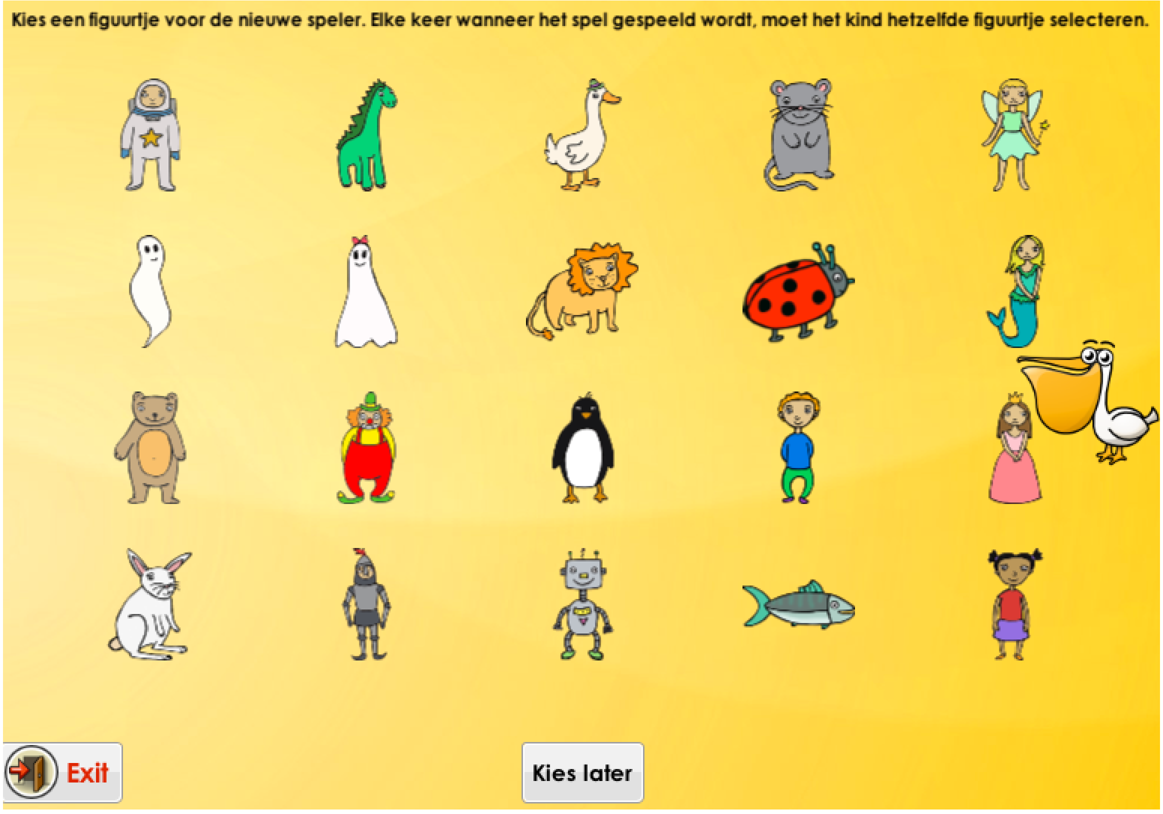

Figure 1. The character selector. Players have to chose a character as a login token and are guided through the game by a pelican (right).

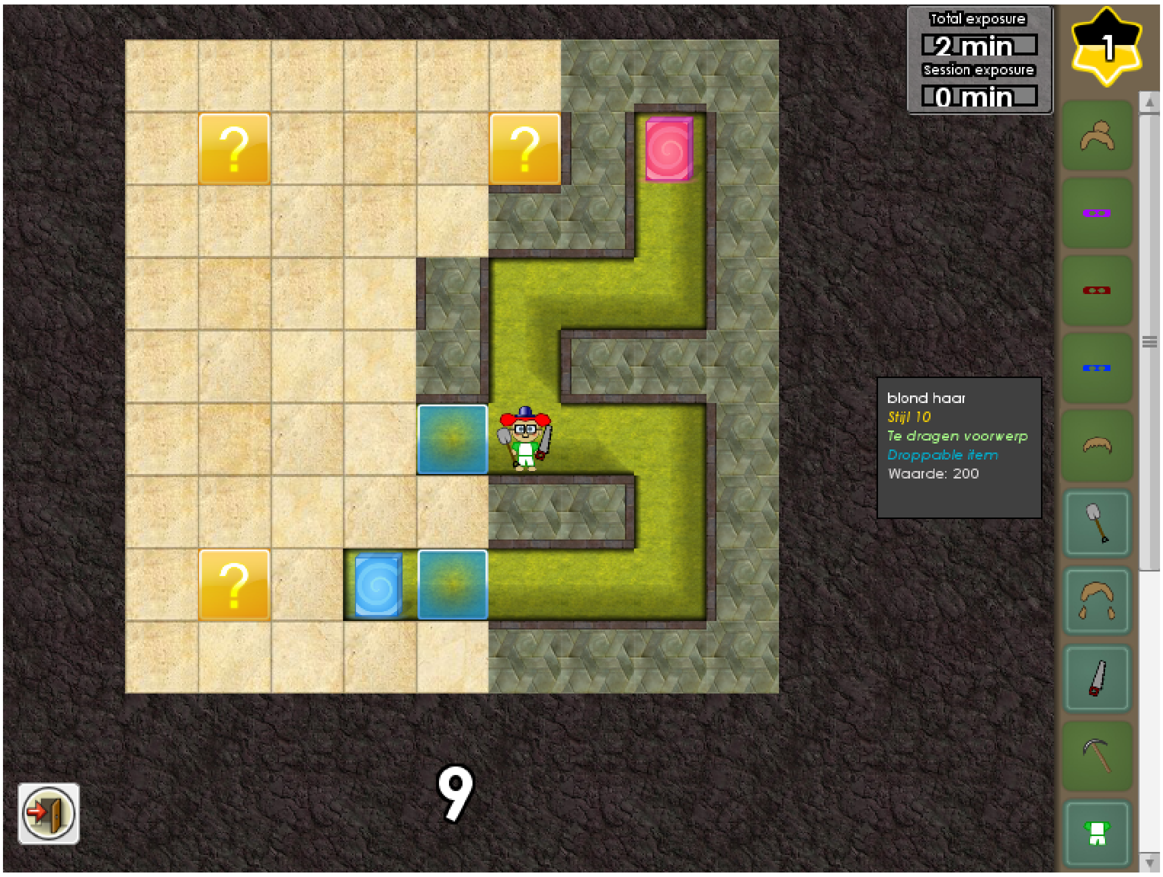

Figure 2. The randomly generated labyrinth map with inventory (right), experience and exposure (top right).

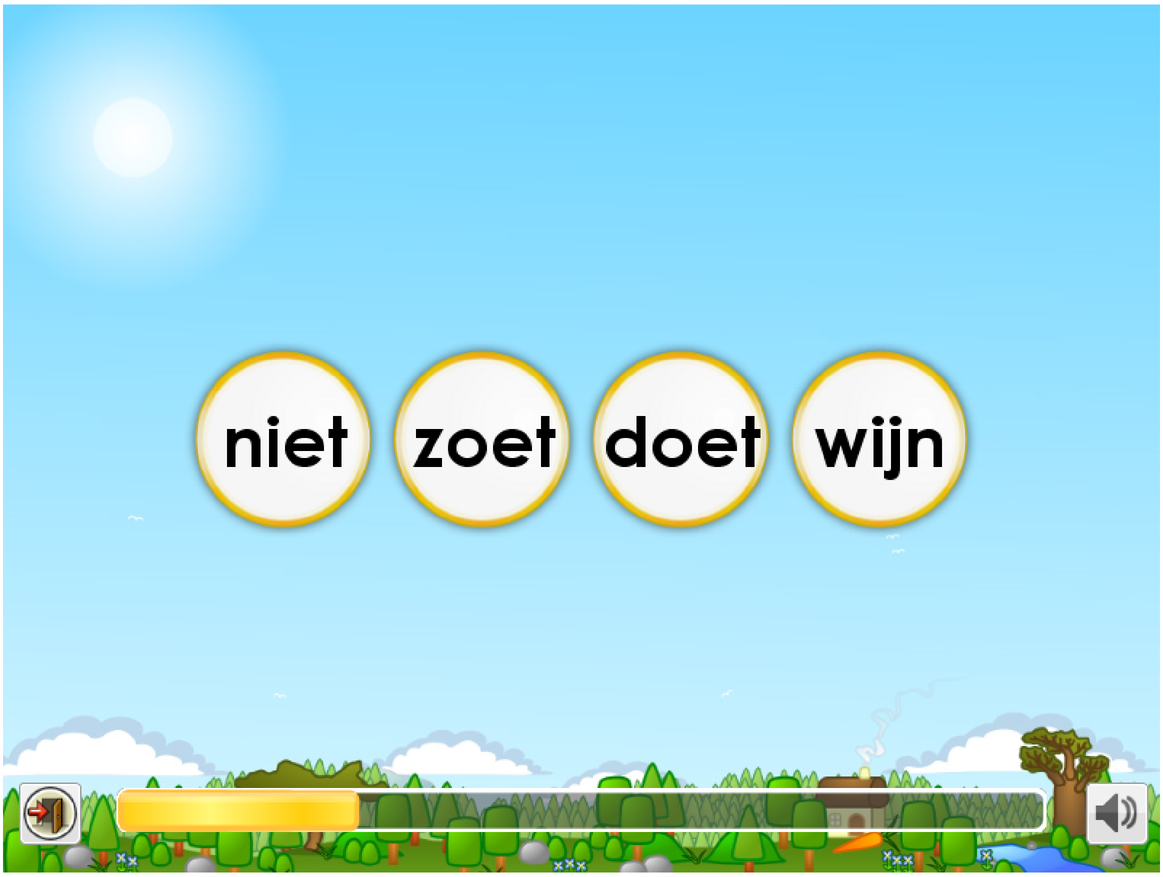

Figure 3. The trial game.

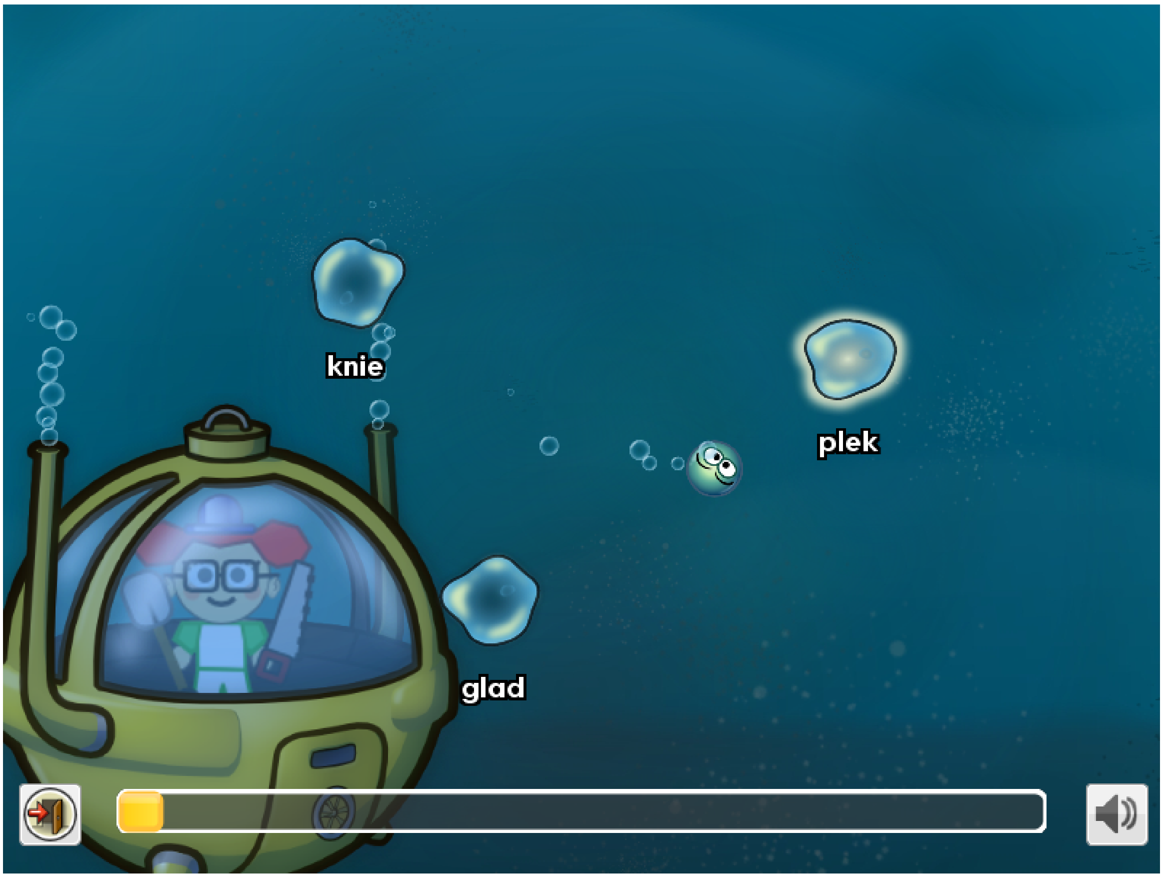

Figure 4. The flow game.

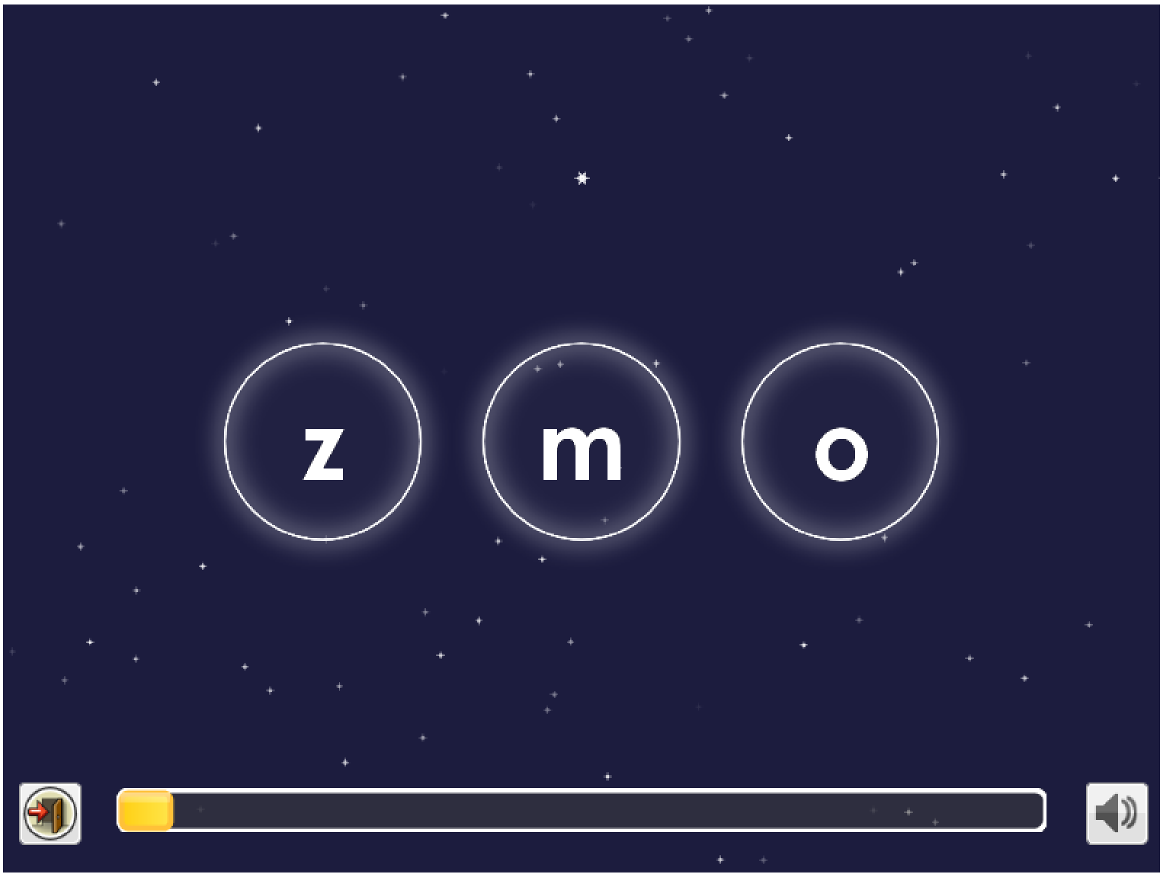

Figure 5. The star game.

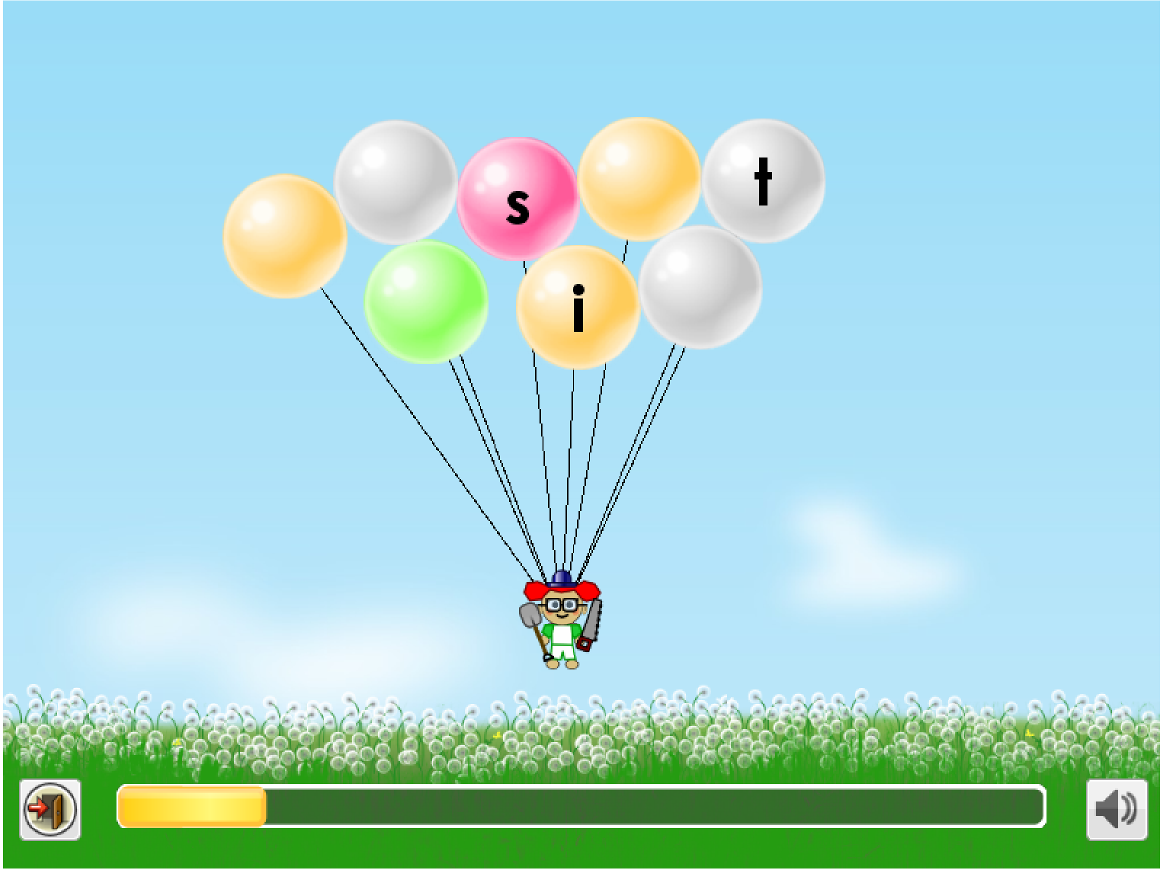

Figure 6. The balloon game. Balloons burst upon correct selections until the avatar lands on the ground.

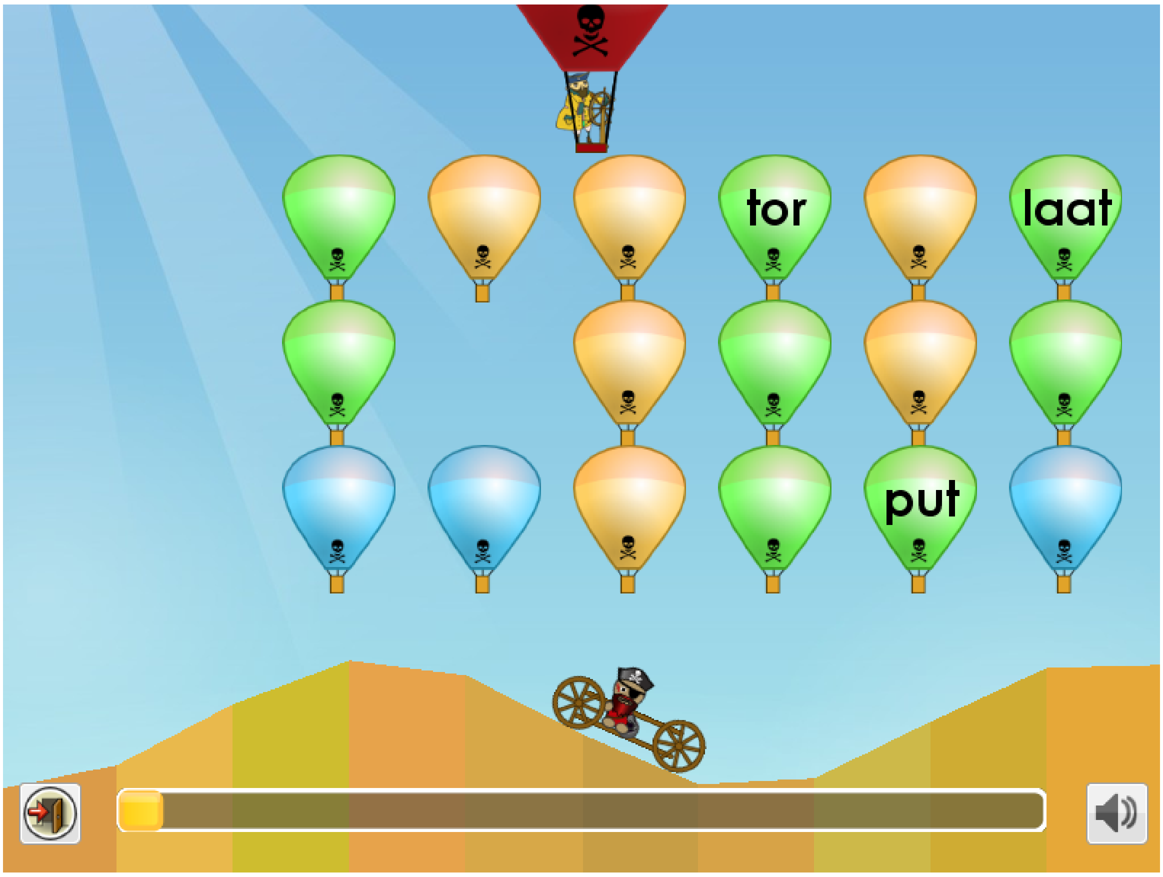

Figure 7. The pirate game. Balloons move left and right, and burst after a correct response.

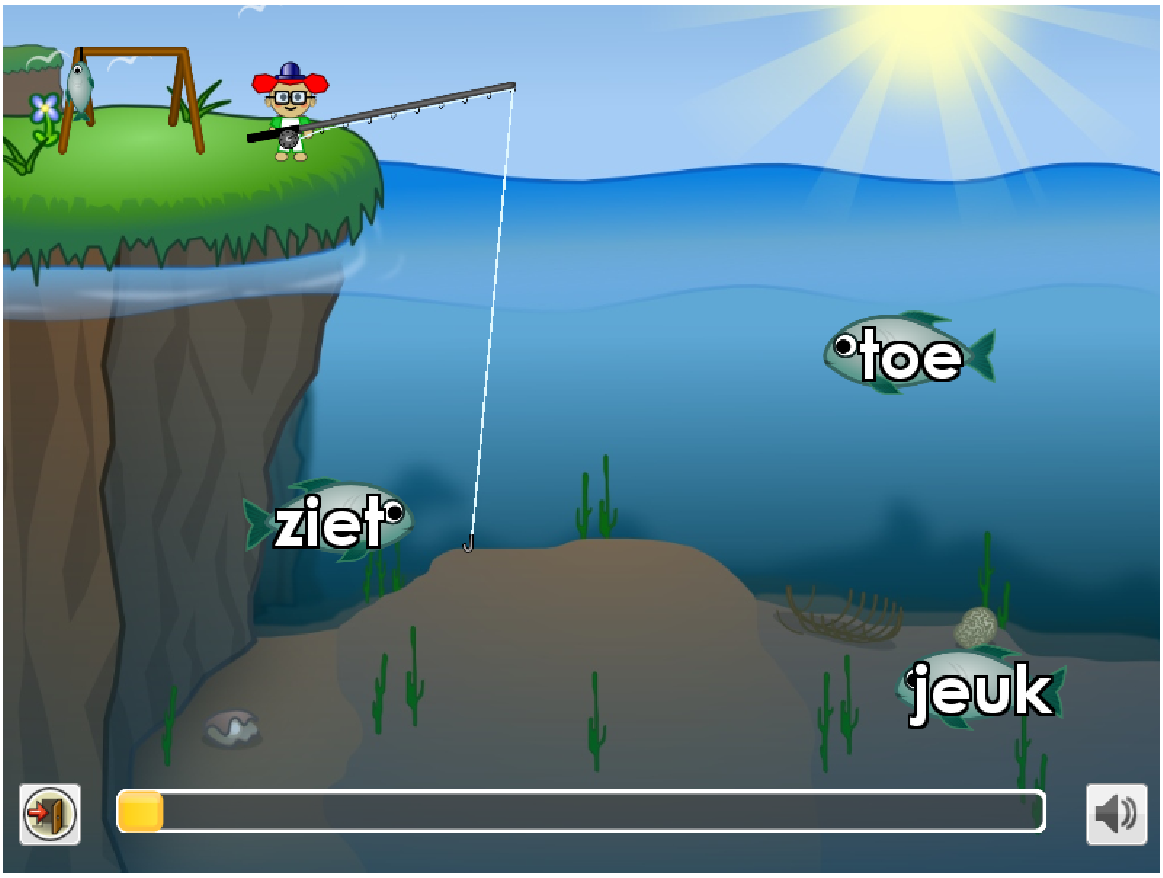

Figure 8. The fishing game. The fish with the words swim left and right.

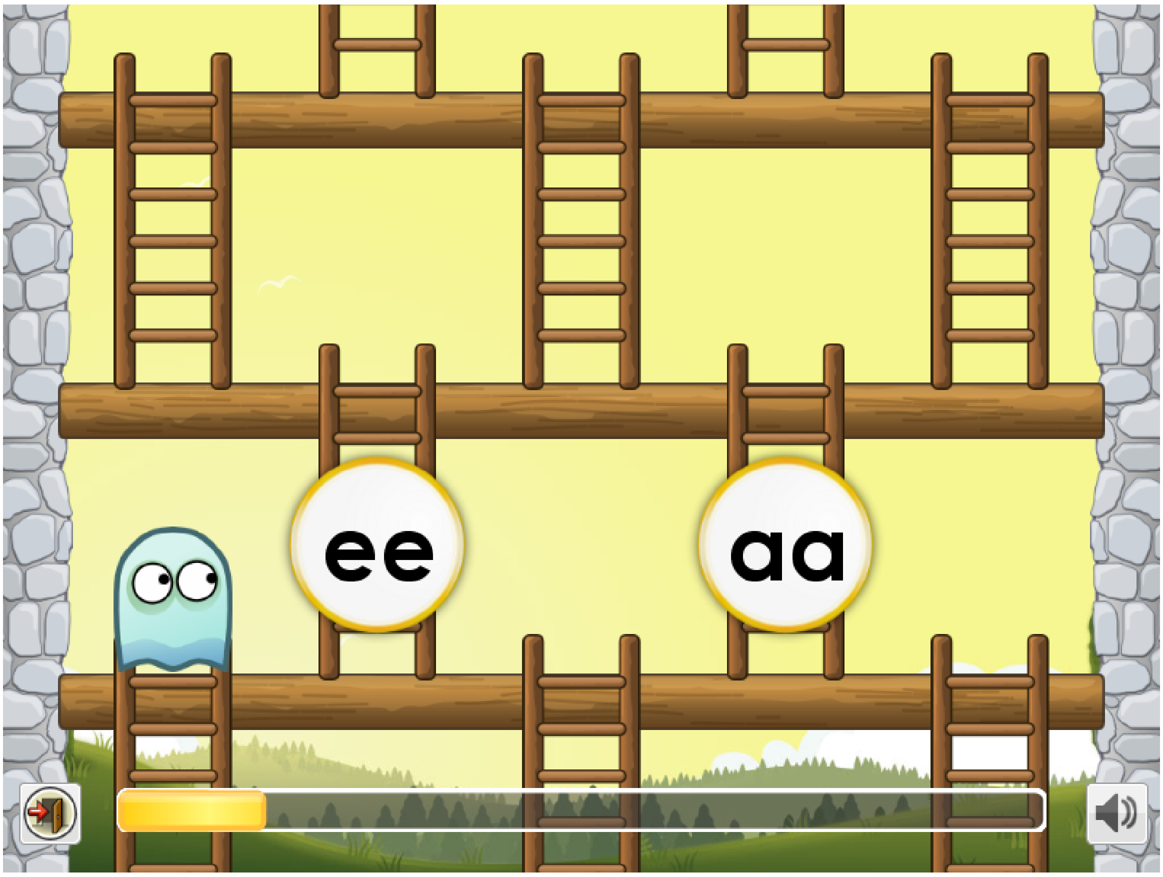

Figure 9. The ladder game. The ghost moves one platform up upon correct response, and one platform down upon a wrong response.

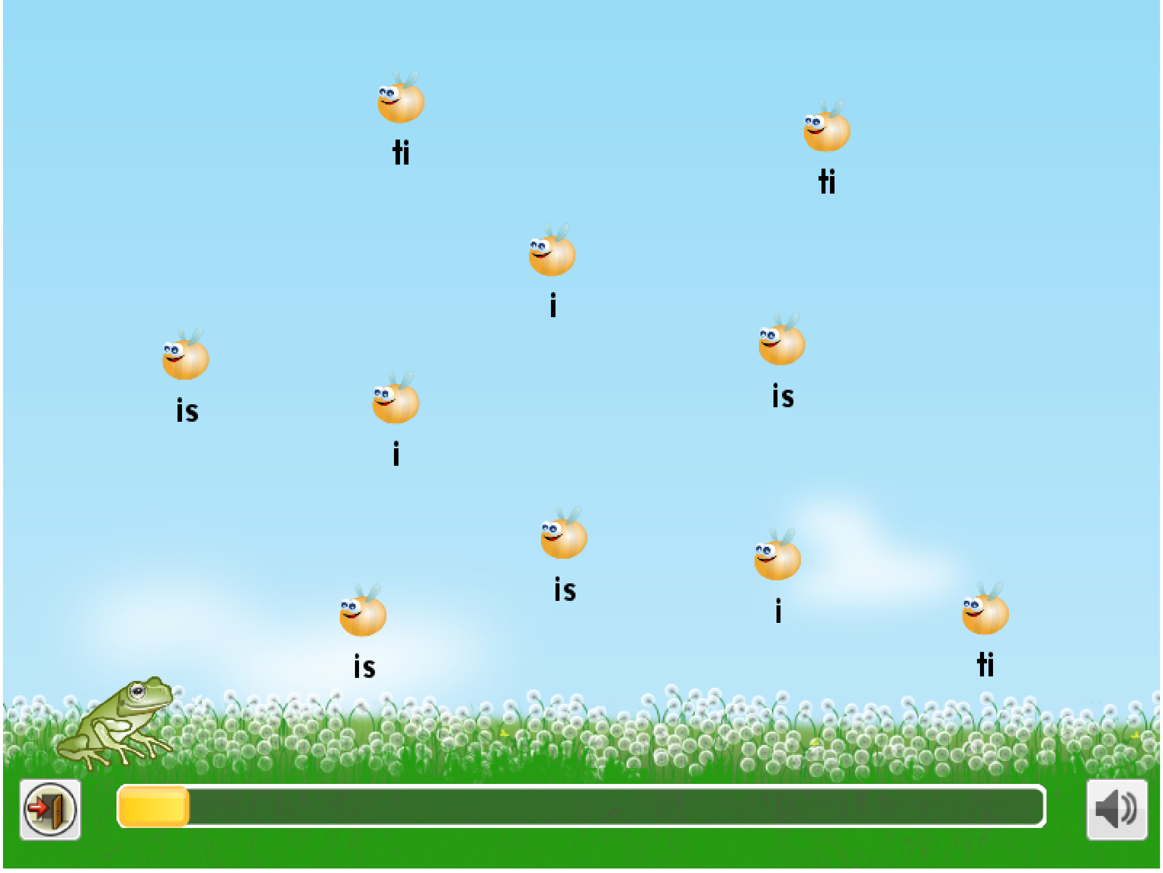

Figure 10. The frog game. The tongue of the frog eats the selected targets, and spits any incorrect selection back out into the pool of distractors.

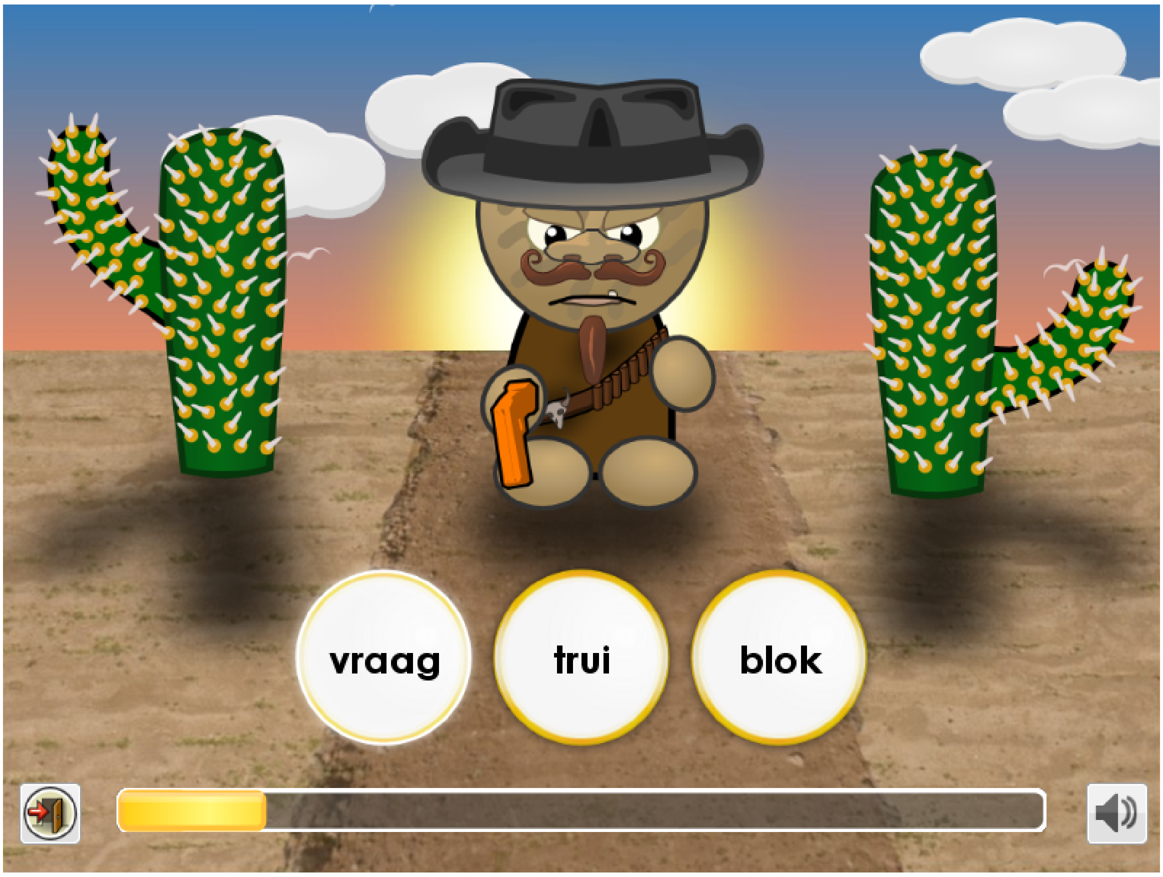

Figure 11. The duel game. The enemy slowly raises its water pistol to splash the player, a fast and correct response will instead splash the enemy.

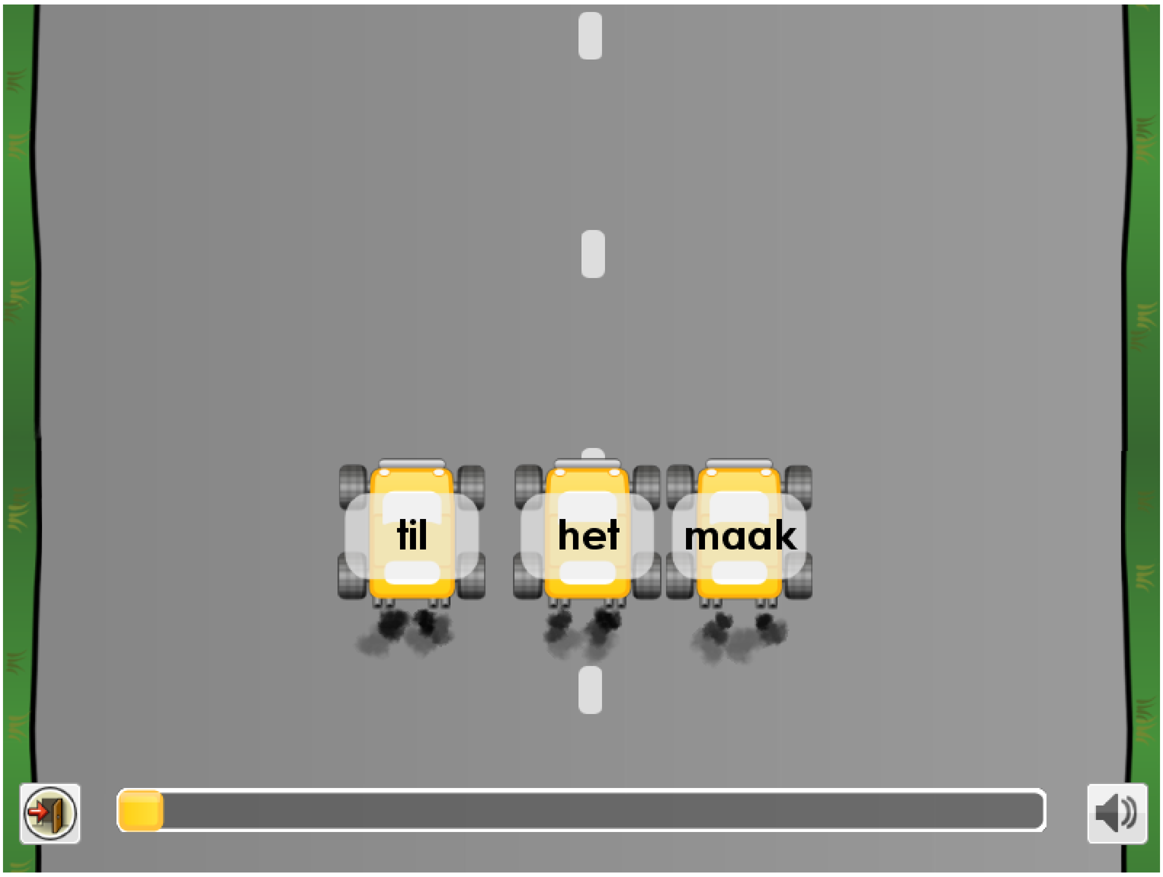

Figure 12. The race game. The cars slowly move up the road until they are out of sight.

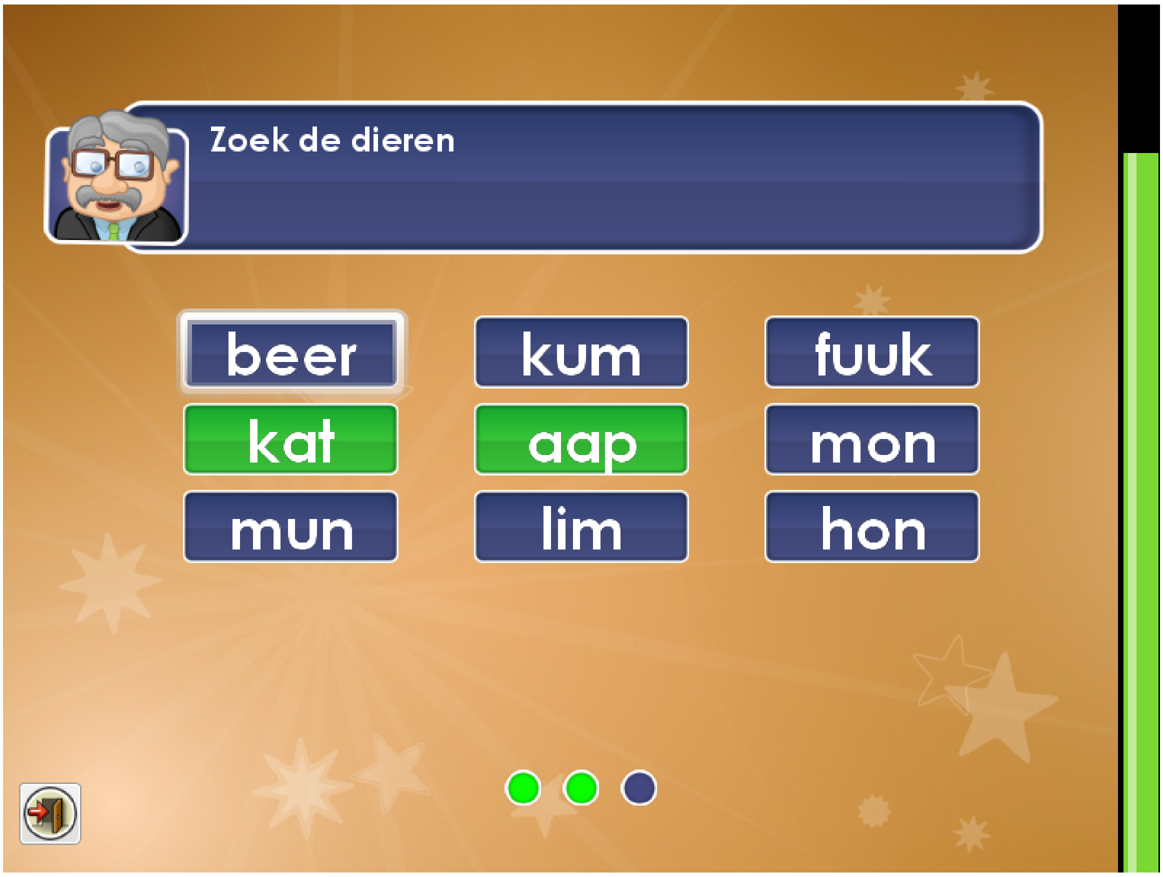

Figure 13. The quiz game. Regenerating quiz game with a bar indicating the remaining time on the right hand side.

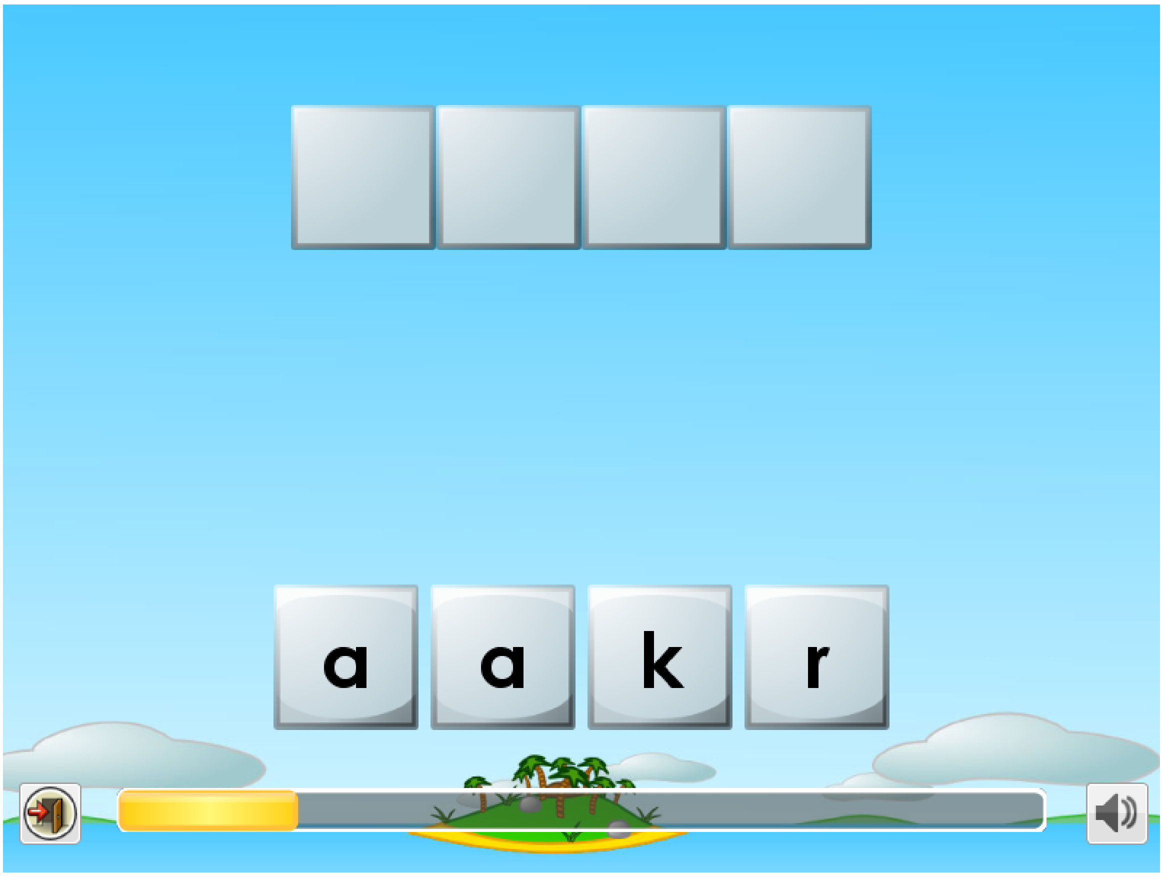

Figure 14. The word forming game. In this case the target is raak ('hit').

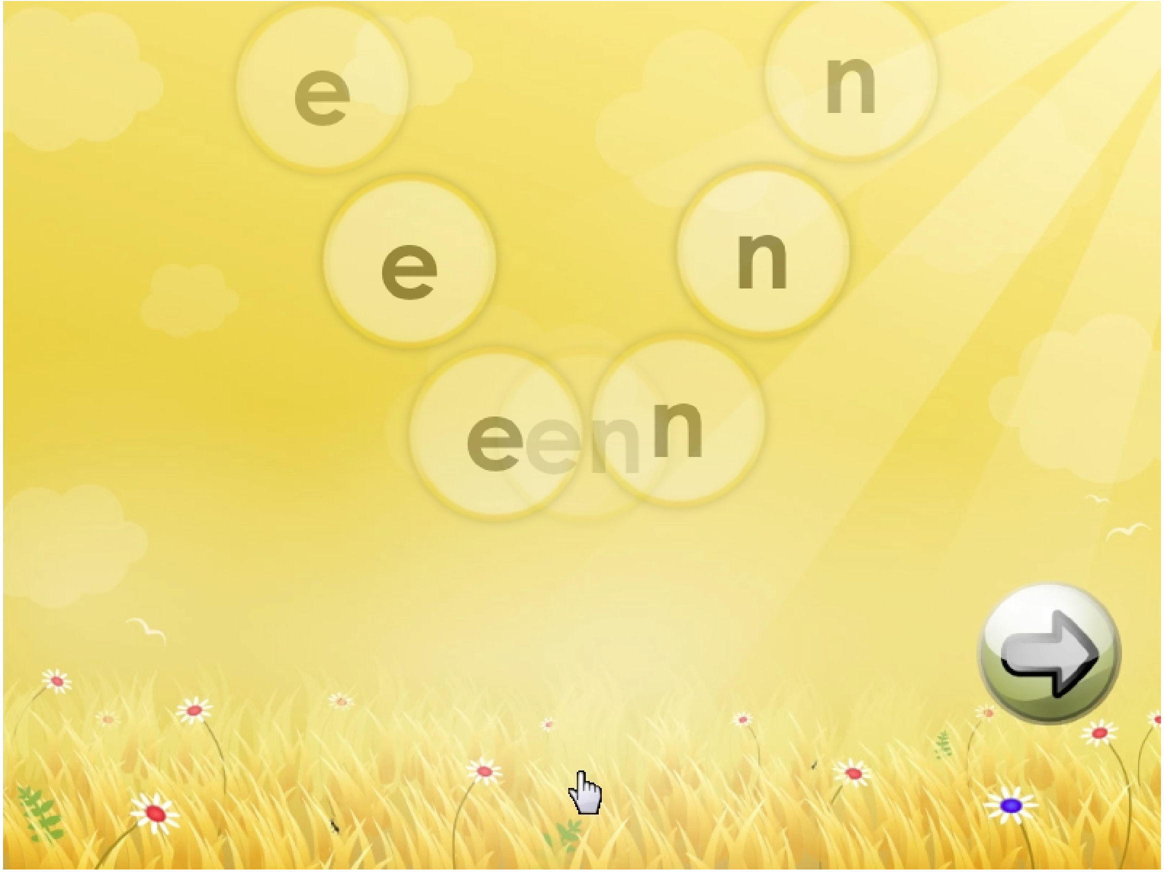

Figure 15. The blending demo showing a stepwise convergence of graphemes (in this case *e* and *n* into *en*). This image consists of four overlaid screenshots.

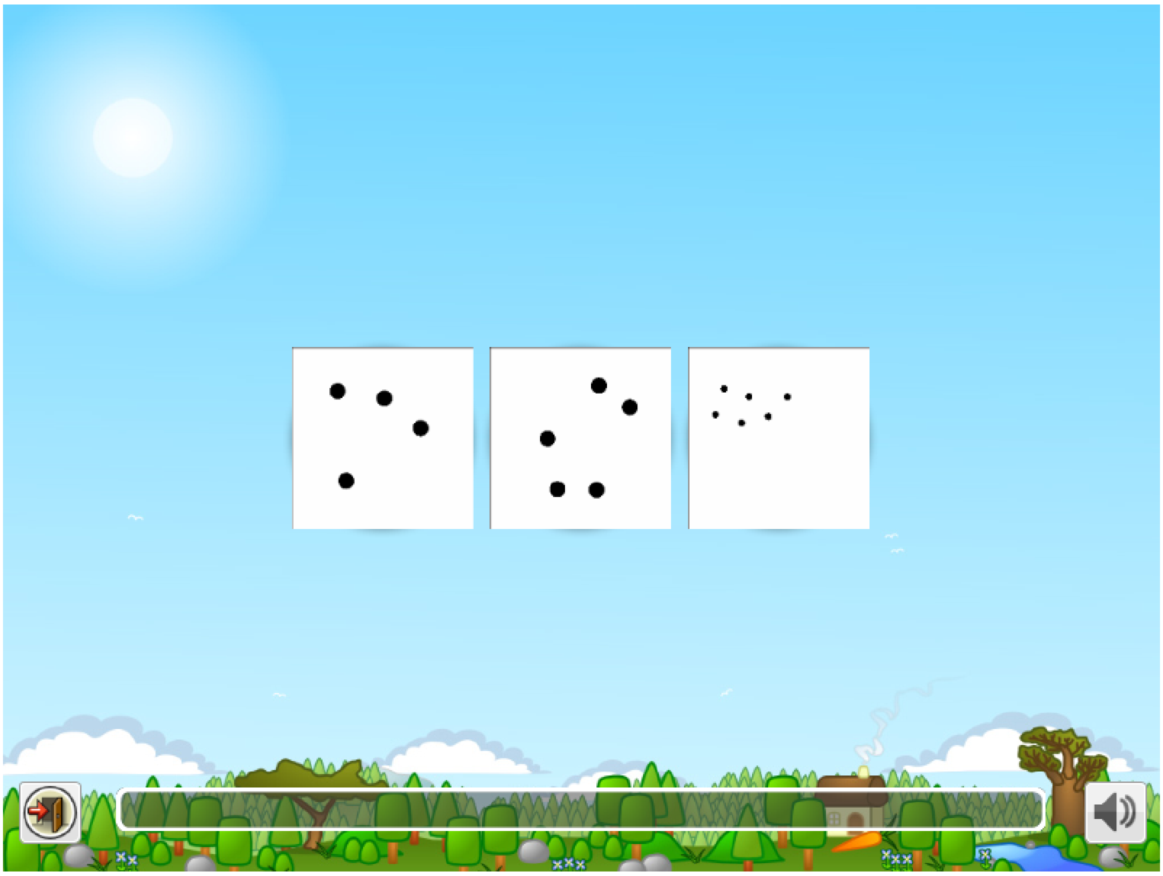

Figure 16. The counting task. The player has to select the image with five dots.

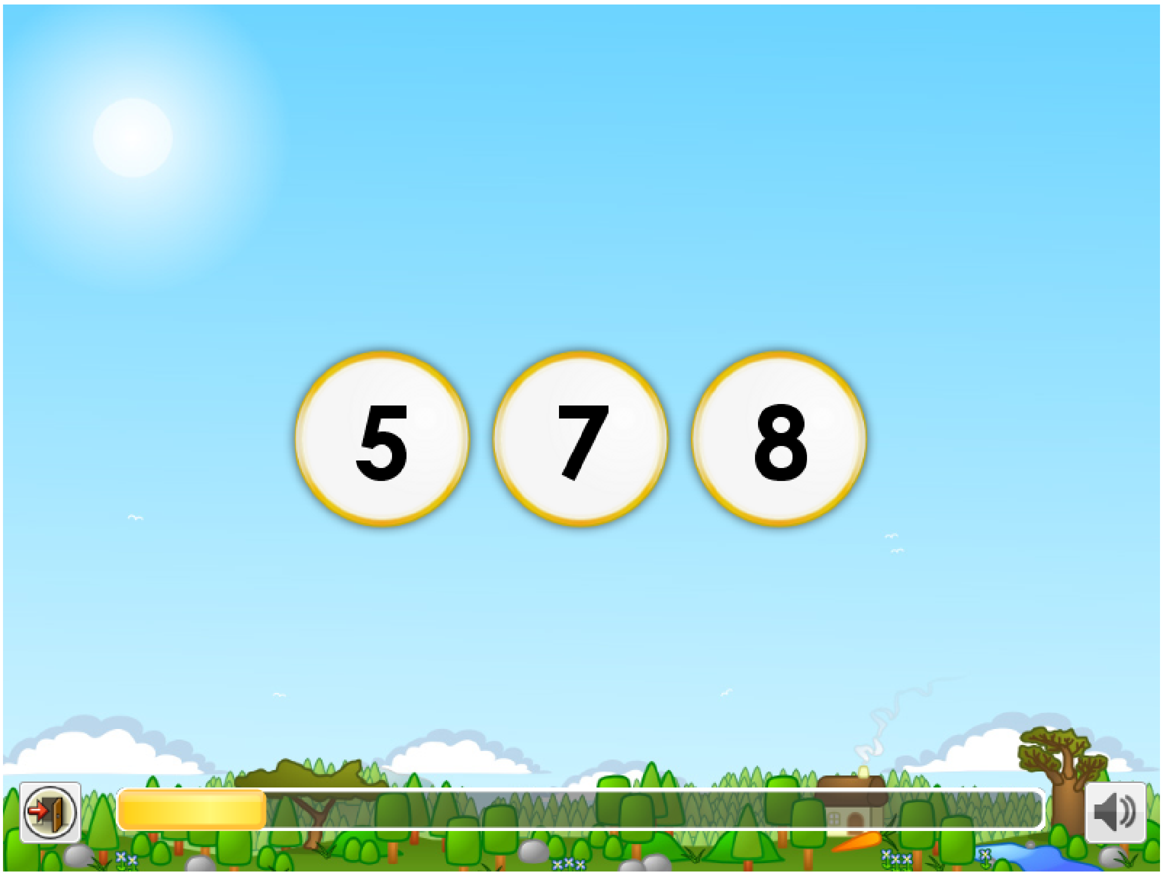

Figure 17. The number knowledge and neighboring number task. Select the number five, or the number which comes after seven or before six.

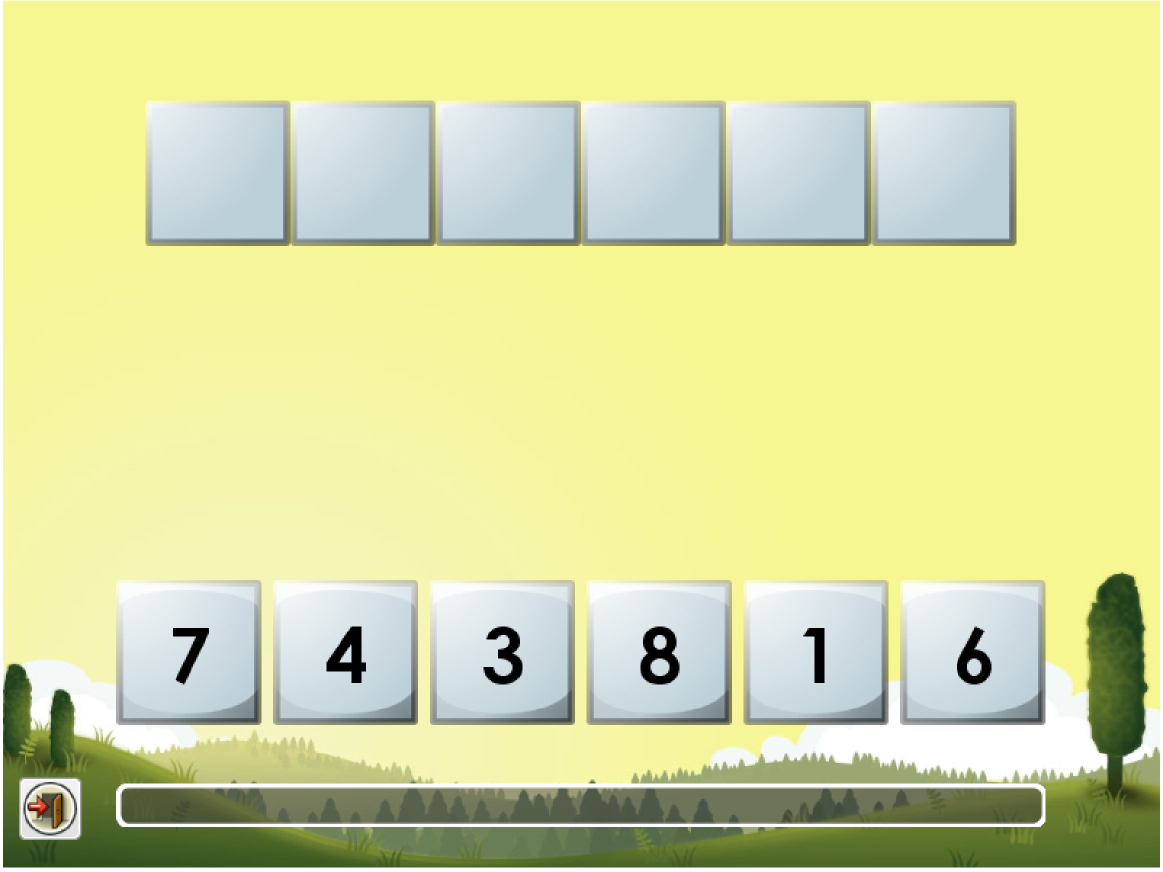

Figure 18. The number sorting task. Sort from low to high.

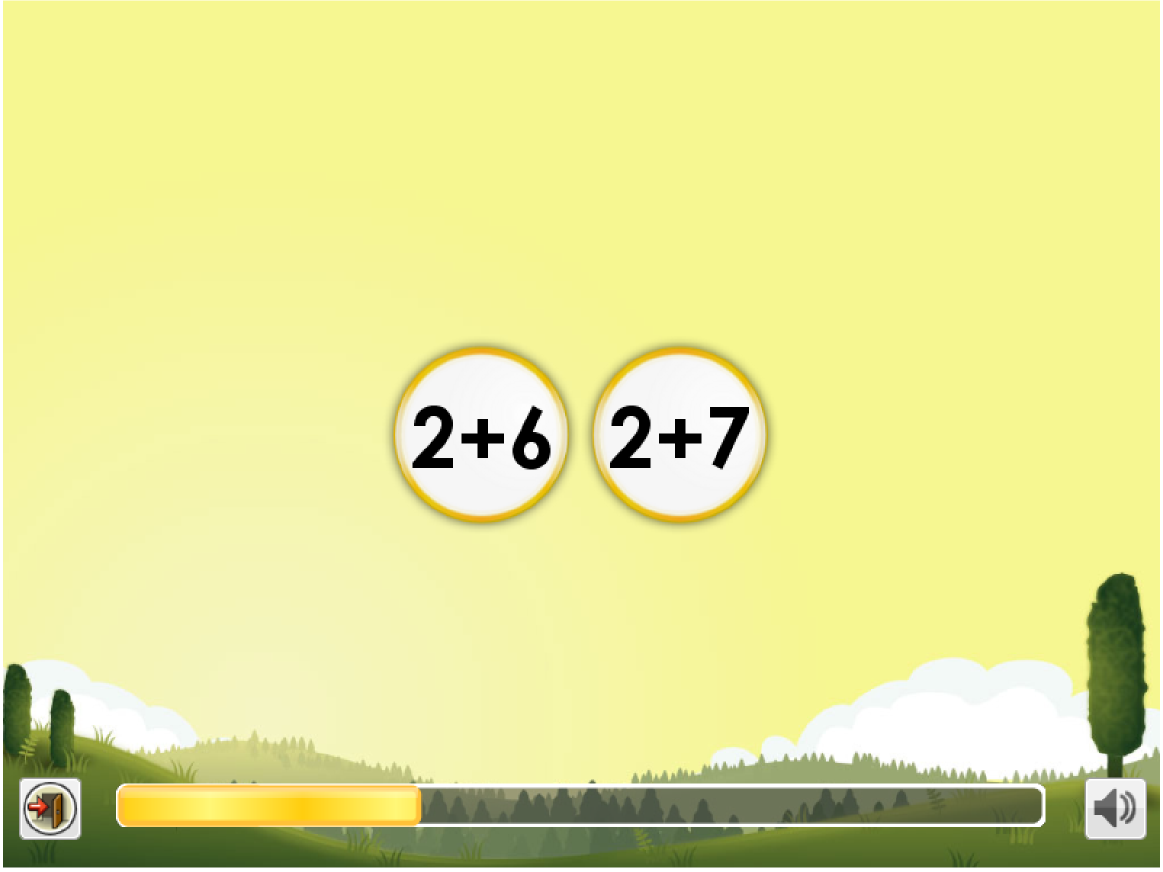

Figure 19. The addition task. Select the bubble that equals eight.

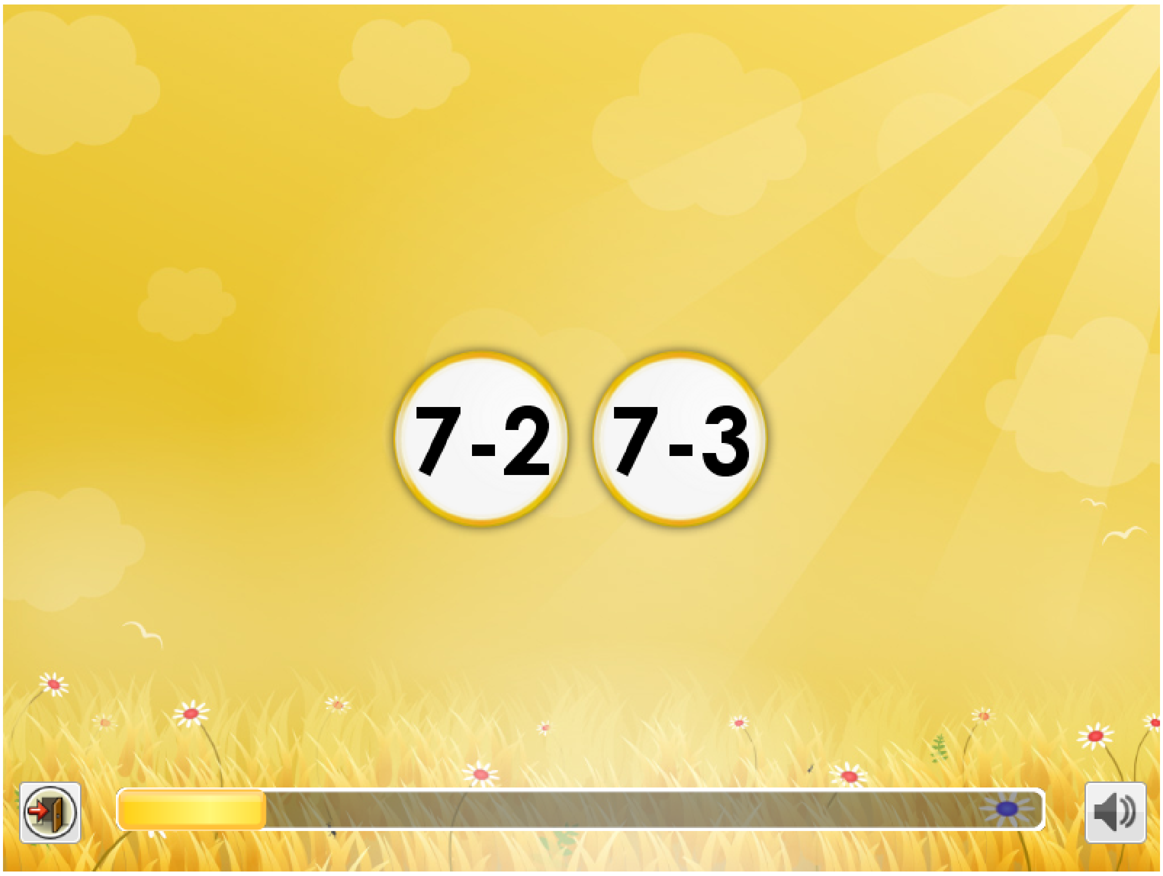

Figure 20. The subtraction task. Select the bubble that equals five.

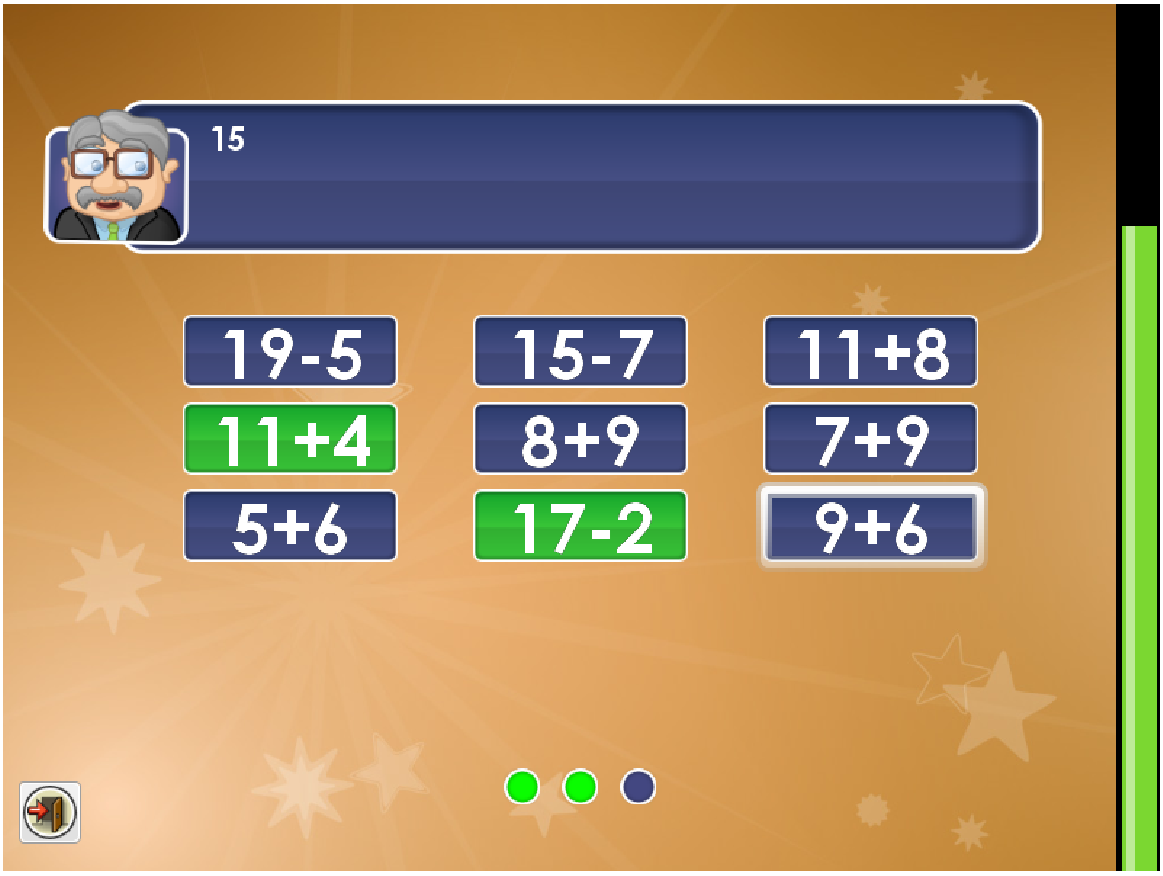

Figure 21. The quiz addition and subtraction task. The player has to select the sums which equal 15?

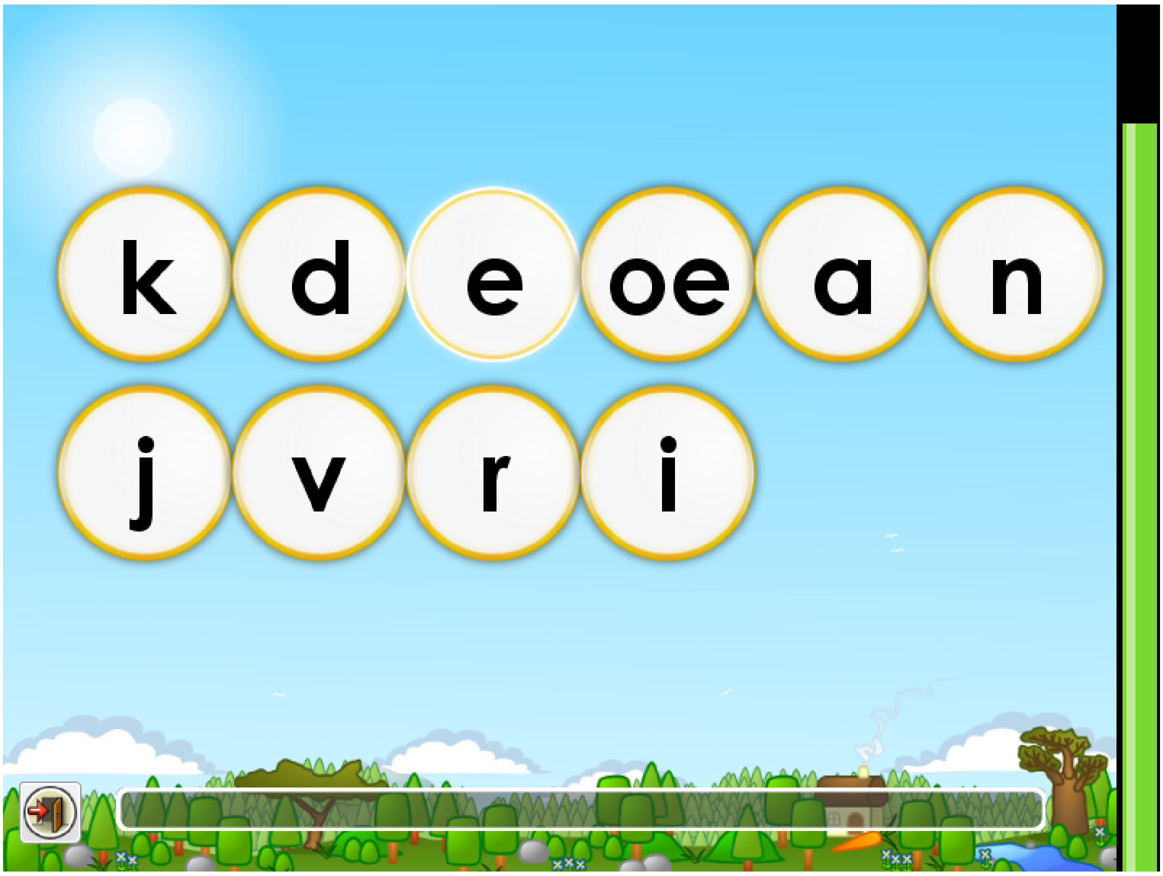

Figure 22. The easy letter-sound identification assessment. The bar on the right indicates the remaining time within the assessment.

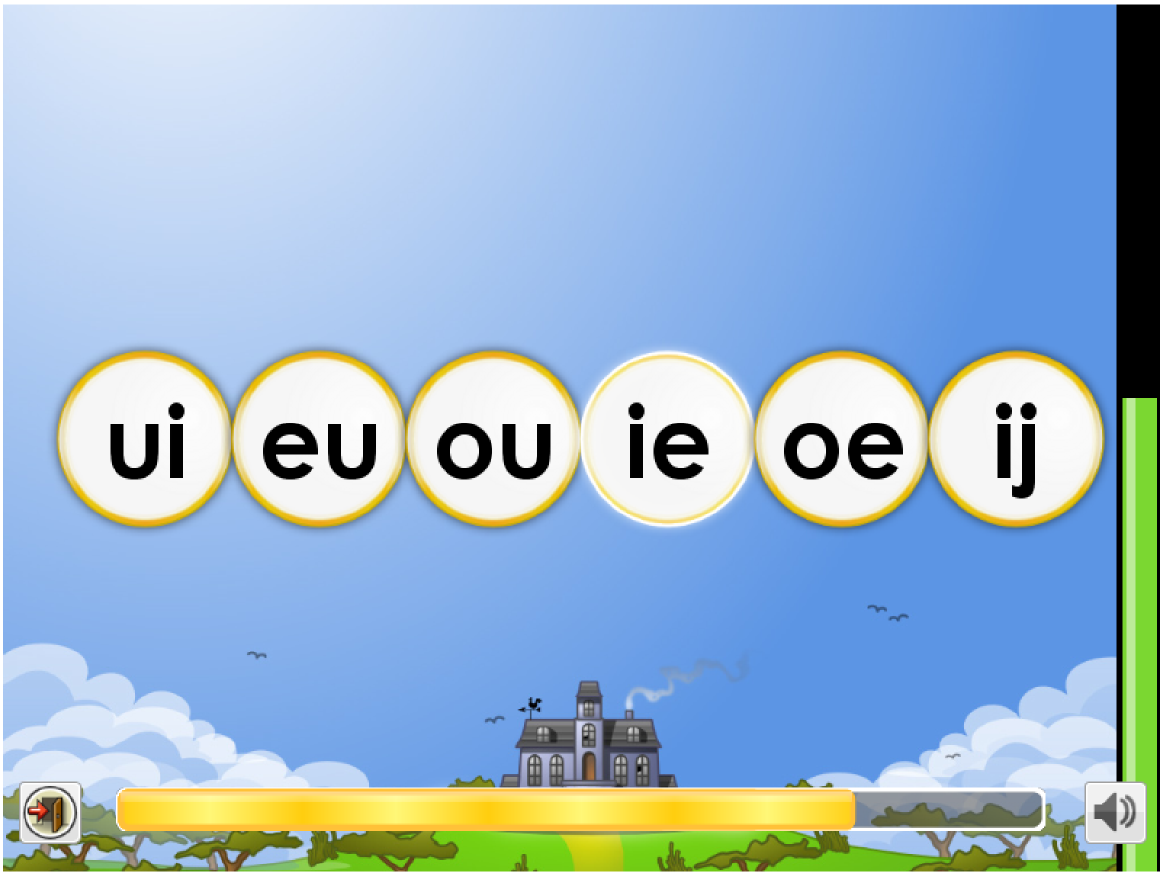

Figure 23. The difficult letter-sound identification assessment.

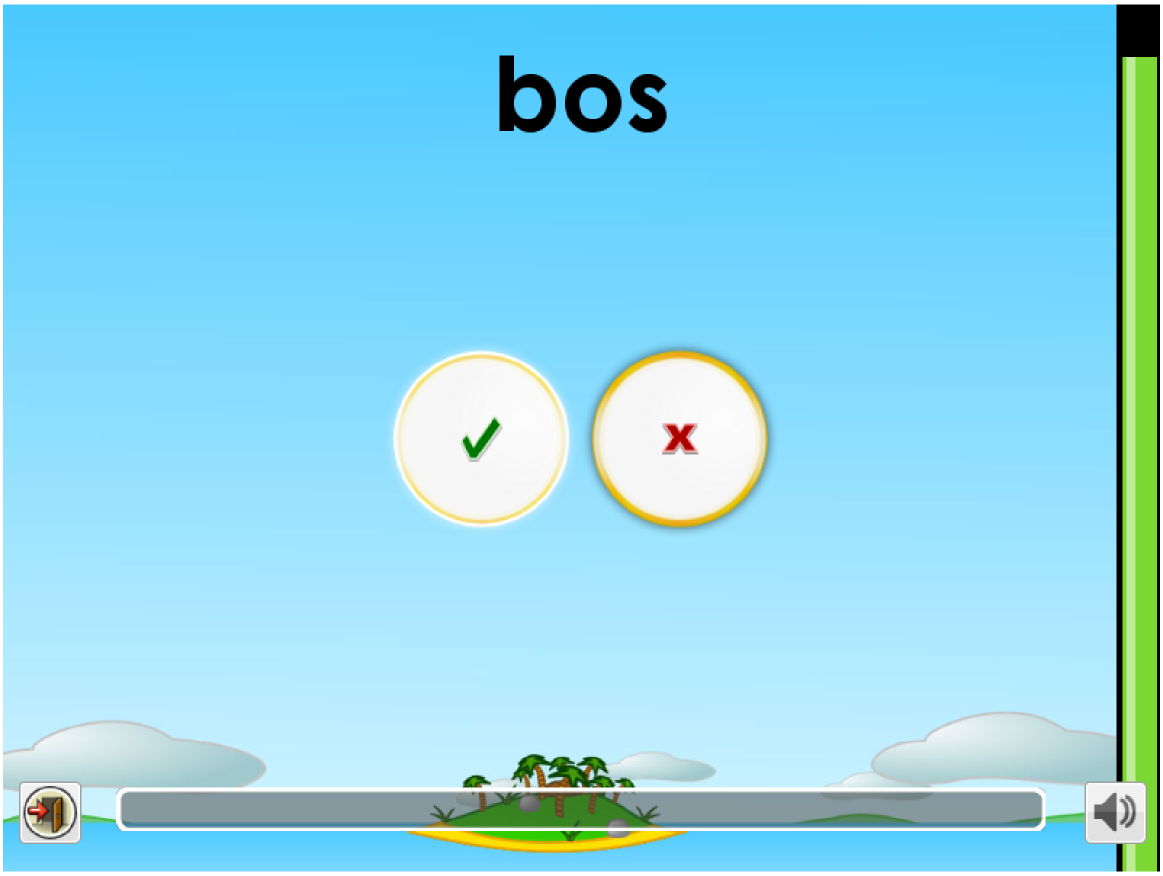

Figure 24. The written lexical decision assessment.

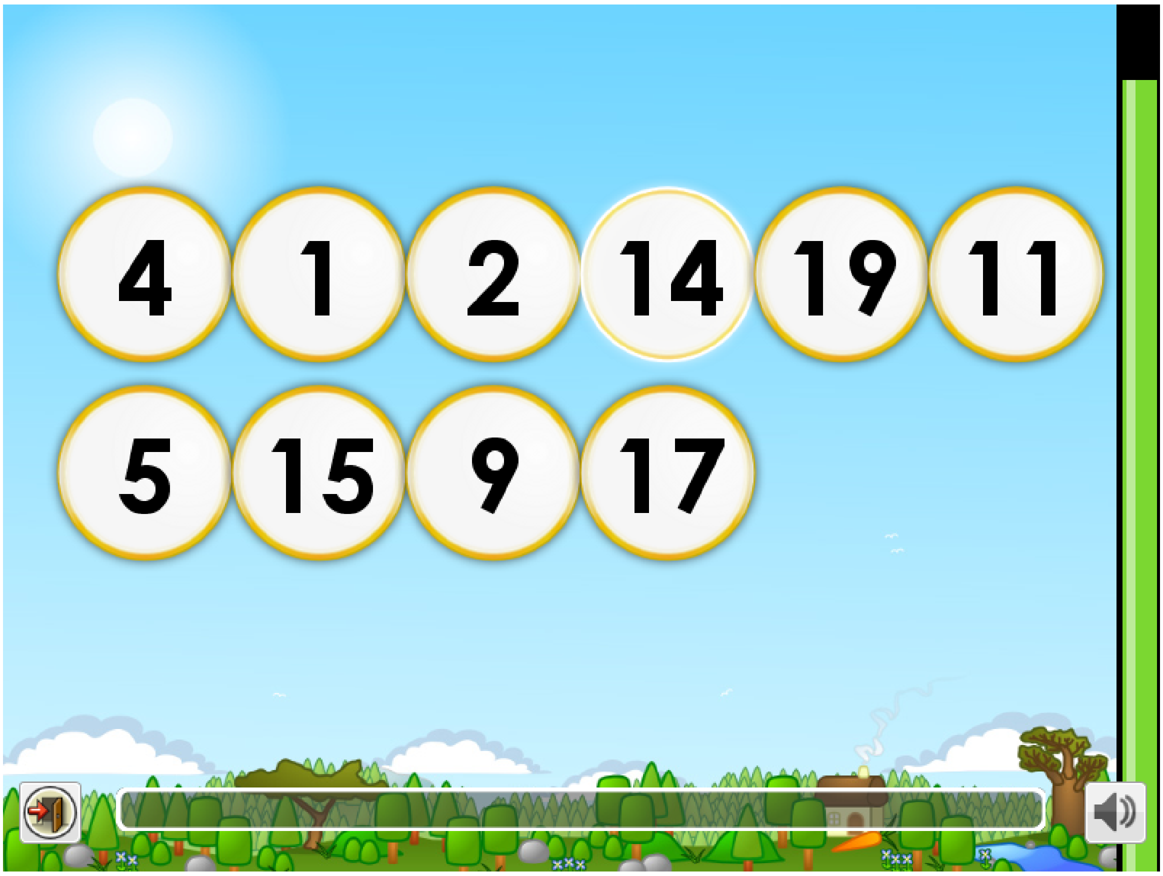

Figure 25. The number knowledge assessment.

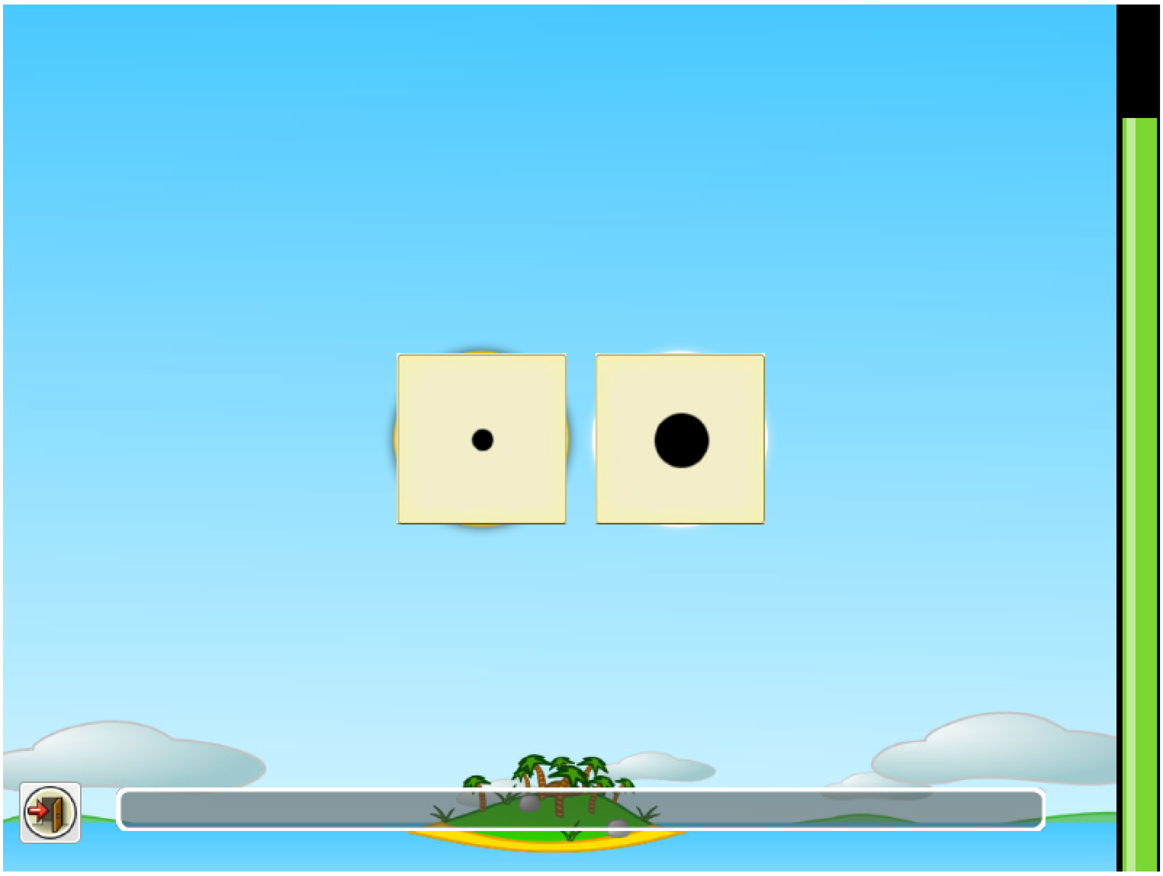

Figure 26. The biggest dot assessment.

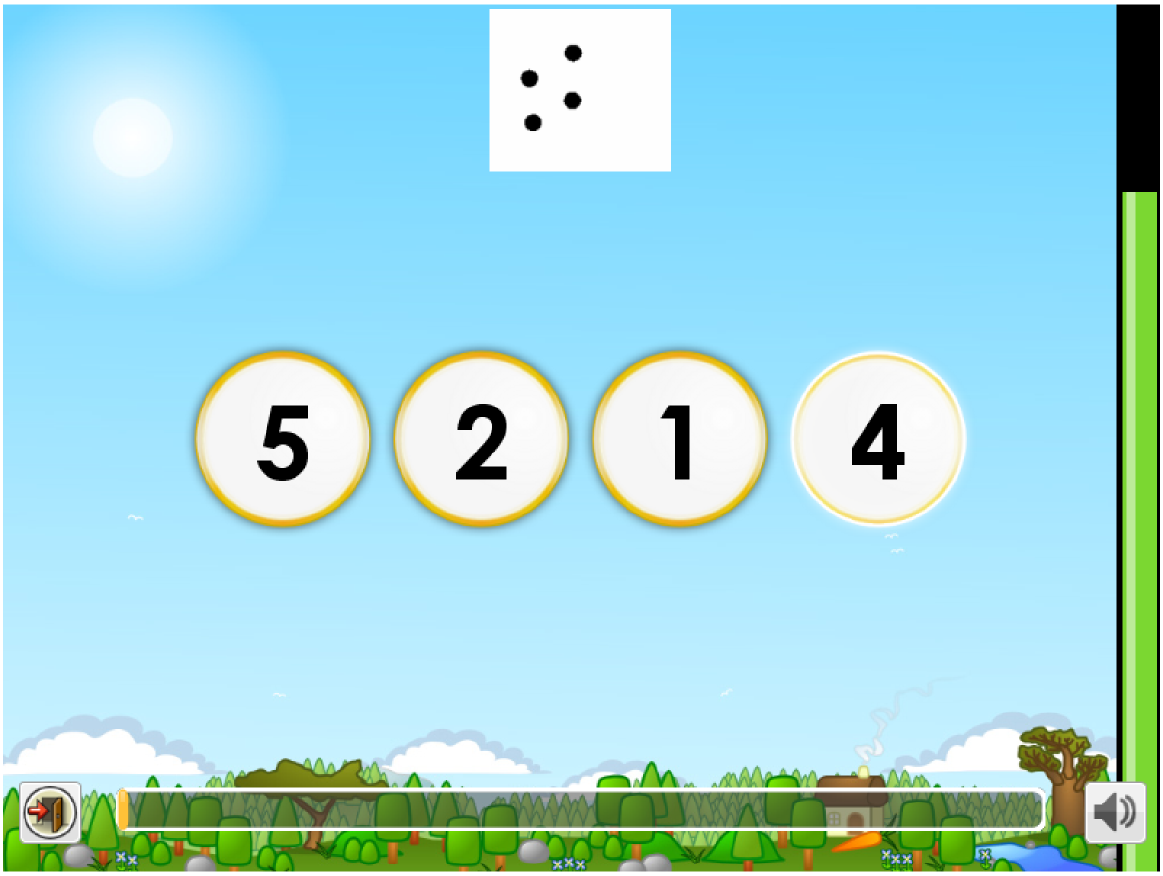

Figure 27. The counting assessment.

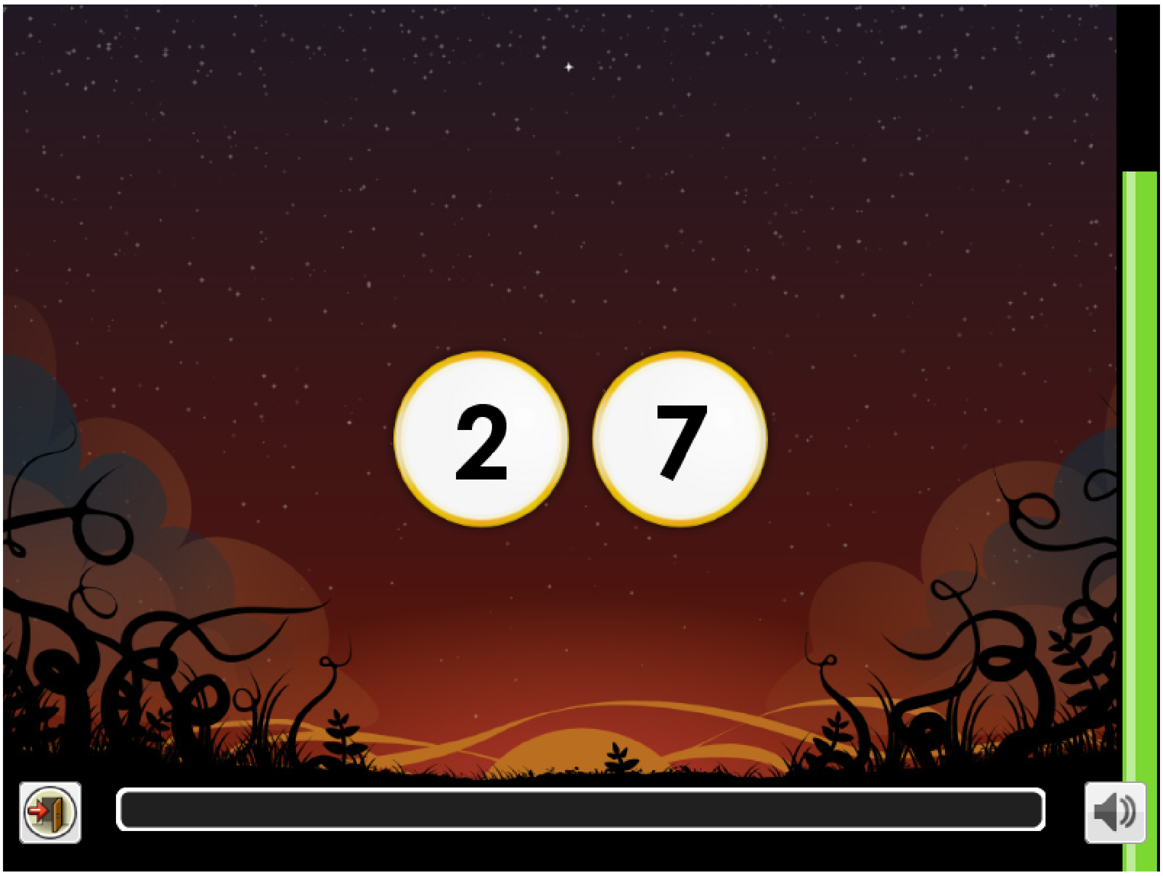

Figure 28. The bigger number assessment (single digit).

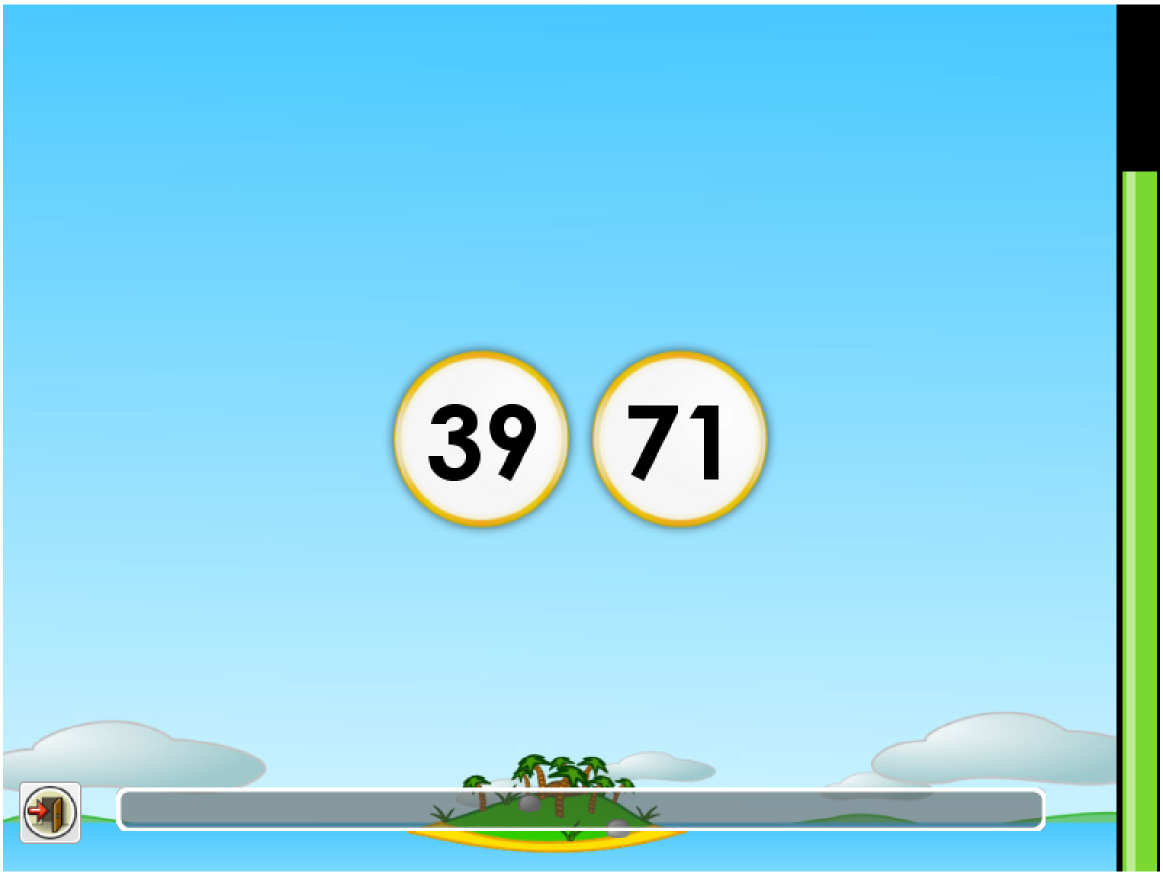

Figure 29. The bigger number assessment (double digit).

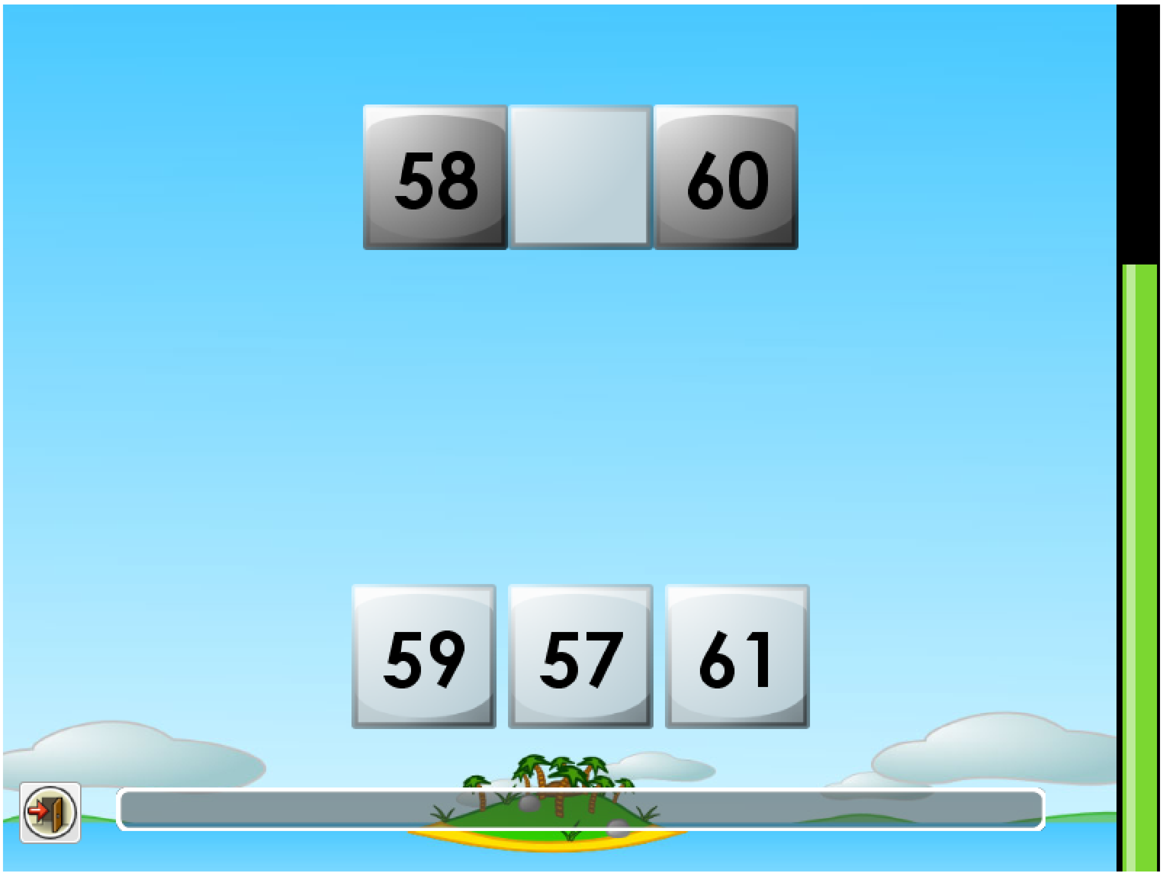

Figure 30. The missing number assessment.

Word forming game

The word forming game is a task where children need to click on boxes in the right order to create the correct sequence of what they heard (Figure 14 on page 18). Boxes can contain anything from graphemes, over syllables to words and also numbers to make up words or sentences or an ascending or descending row of numbers.

4.3 Gaming tasks

Based on these game modules, the reading and the math games contain several possible training tasks which are presented below.

Letter knowledge

In this task children hear a phoneme and must select the corresponding grapheme on the screen (e.g. Figures 5, 6 & 9). This task can be implemented in most game modules presented above, e.g. the Trial, Balloon or Ladder game. The following instruction is given to the child during the first occurrences of this task: *In dit spel hoor je de klanken die bij de verschillende letters horen. Luister goed en kies de letter die past bij de klank die je net hebt gehoord.* ('In this game you'll hear the sounds which belong to the different letters. Listen carefully and choose the letter that belongs to the sound you just heard.').

Reading

Reading can be considered the natural extension of the letter knowledge tasks within the game, showing words instead of letters (e.g. Figures 3, 4 & 7). Once children know letters sufficiently well they are combined into syllables and words which need to be read. All game modules can accommodate this task, and especially the quiz module is well suited to promote reading fluency at later stages of the game. There are no specific prompts associated with this generic task.

Spelling

During the spelling task children hear a word and need to spell it with graphemes (Figure 14 on page 18). Spelling can be done on a grapheme level to spell syllables or words, on a syllable level to spell words, or even on a word level to build sentences. The instruction given to the child is: *Probeer nu het woord zo goed mogelijk te spellen, door in de juiste volgorde op de vakjes te klikken.* ('Try to spell the word the best you can by clicking on the boxes in the right order.'). The only game module which can implement spelling is word building (see above).

Blending (demo)

This is not specifically a task which can be played, but the first levels that present CV, VC and CVC syllables always include a demo of blending two or three graphemes into sublexical units or monosyllabic words (Figure 15 on page 19). Children first hear single phonemes and see the corresponding graphemes on screen (e.g. ⟨e⟩ and ⟨n⟩), and then the visual and auditory space between the two units is reduced in three steps to ultimately blend the two phonemes into a syllable (in this case *en*). This can be considered the only explicit form of instruction which is currently used within the Dutch game version. The instructions given to the child were: *Nu gaan we twee/drie klanken combineren. Ik zal je laten zien hoe dat moet.* ('Now we will combine two/three sounds. I'll show you how it's done.').

Number knowledge

Children hear a spoken number and must pick the corresponding written number (made up by one or two digits) on the screen (Figure 17 on page 20). The following instruction was given to the children: *In dit spel, hoor je steeds een cijfer. Luister goed, en kies het cijfer op het scherm dat past bij het cijfer dat je net gehoord hebt.* ('In this game, you will always hear a number. Listen carefully, and choose the number on the screen, which you just heard.')

Adjacent numbers

In this task, children are asked which number comes before or after another given number (Figure 17 on page 20). The task looks identical to the number knowledge task above and may seem counter-intuitive at first. For the question "Which number comes after 5?" the correct response is 6 and not the 5 which was just heard. The instructions for the children were *Welk cijfer komt voor/na 5?* ('Which number comes before/after 5?').

Counting dots

The child hears a spoken number and needs to respond by counting the dots in three provided pictures and choosing the picture which has the correct number of dots (Figure 16 on page 19). The counting goes up to 17 dots in the training levels but is limited to 6 dots in the assessment (see below). The given instruction was: *Tel de stippen, en kies het bijbehorende getal!* ('Count the dots, and choose the corresponding number').

Sorting

Similar to the spelling task in the reading game, the boxes with numbers of the word building module have to be sorted either in ascending or descending order (Figure 18 on page 20). In early levels this is exclusively done with neighbouring numbers (e.g. 3, 4 & 5), but in later stages of the game also non-adjacent numbers (e.g. 7, 9, 12 & 18) are used. The following instructions were given to the child: *Probeer de blokken met cijfers van laag tot hoog te ordenen! Probeer de blokken met cijfers van hoog tot laag te ordenen!* ('Try to sort the blocks with numbers from low to high! Try to sort the blocks with numbers from high to low!').

Addition and subtraction

Basic addition and subtraction is added to the game, but in a rather unconventional way, as it is limited by the current game engine. Children hear the result of an addition or subtraction (e.g. eight) and see two or three formulas on the screen - one of which will have a result of 8 (see Figures 19 & 20). In theory, children need to do the addition for each single formula to be able to pick the correct response. Note that within a level the operations are not mixed, they are either all additions, or all subtractions. The instructions used for this type of task were: *Laten we cijfers gaan optellen/aftrekken. Welke som heeft als uitkomst 5?* ('Let's add/subtract some numbers. Which sum has a result of 5?'). Furthermore, addition is also implemented with the quiz game, where a total of 9 formulas are on the screen at once, and 3 of them result in a number displayed on top of the screen (see Figure 21).

4.4 In-game assessments

To get more sensitive measures for the evaluation of game-based learning outcomes, we implemented parts of our assessment battery into the game itself. These special assessment levels appear the first time the game is played and can be triggered automatically and manually at later stages of the gameplay. In contrast to regular training levels, these assessment levels have strict time limits of 45 seconds up to 3 minutes, are not adaptive and thus have a fixed order and/or number of distractors. In addition, they are always passed successfully and don't have to be repeated, and they give no feedback on accuracy of a response. Apart from conventional raw scores for number of items correct and average time per correct item, all these levels provide per item accuracy and response time measures, which allow for single trial analyses. To also be able to evaluate the effects of playing the reading game, we implemented a range of assessments into the game which tap both, reading related skills, as well as arithmetic knowledge. These purpose built levels are one of the novelties of GG-NL used for the study presented in this work.

4.4.1 Literacy assessments

Easy letter knowledge

In this task children hear a phoneme and must select the corresponding grapheme on the screen (Figure 22 on page 22). The following instructions were given to the child: 1) *Laten we zien welke letters je kent!* ('Let's see which letters you know!'), and 2) *Laten we zien welke andere letters jij kent!* ('Let's see which other letters you know!'). A total of 32 phoneme-grapheme associations are tested. For two practical reasons this task had to be divided into several levels: i) the version of the game which we used is not suited to display that many targets/distractors on the screen at once, especially considering that some children play on school laptops with very small (10") screens, and ii) as we were also interested in response time measures we did not want to have one very long and tiring level, but preferred several shorter ones. Ultimately, this assessment consists of three levels with 10 or 11 targets and a time limit of one minute each. Consequently, the distribution of stimuli across these levels has a big impact because some targets may be very easy, unless there is a specific distractor present. As an example, the highly confusable targets ⟨m⟩ and ⟨n⟩, which consciously ended up in different levels are not distractors for one another, and may thus artificially boost the "knowledge" of these letters. It is important to note that all 9 or 10 distractors are visible at all times, giving a very low chance of randomly selecting the correct answer. The distribution of targets across the three levels is described in Table 1.

Difficult letter knowledge

As a consequence of some of the limitations of the easy version of the letter-sound identification task, where some highly confusable target and distractor combinations may not have been presented together, we implemented an additional level where these highly confusable pairs are contrasted (Figure 23 on page 23). This task was identical to the easy version in that the child hears a phoneme and must pick the corresponding grapheme on the screen. Children heard the following instructions: *Ken jij deze letters?* ('Do you know these letters?'). Compared to the easy version, the number of distractors were reduced to five, so that six items were presented on the screen at all times. This task consisted of one level with 10 trials and a time limit of one minute. A list of all trials can be seen in Table 2.

Written lexical decision

The desire to get an additional reading fluency measure from the game itself with per item accuracy and response times lead to the design of a written lexical decision assessment. This task presents a mixed written sequence of 16 words and 16 pseudo words presented one by one on a screen, which should either be accepted as a real word or rejected as a pseudo word by clicking on a green checkbox or a red cross, respectively (Figure 24 on page 23). Because of this rather difficult, and in comparison, widely different game type, the first two trials are training items describing how this assessment works. The following instructions were used to explain the task to the children: *In dit spel zie je woorden op het scherm die echte woorden, of onzinwoorden zijn. Druk op de groene knop als het een echt woord is, of de rode knop als het geen word is. Bijvoorbeeld, het eerste woord is bos. Een bos bestaat uit bomen, dus het is een echt woord. Dan druk je op de groene knop. Het volgende woord is 'kos', dit is geen echt woord, dus druk je op de rode knop.* ('In this game, you can see words on the screen which are real words, or non-sense words. Click on the green button if it is a real word, or the red button if it is not a word. Let's take forest for example. A forest consists of trees, so it is a real word. In this case you press the green button. The next word is kos, this is not a real word and you press the red button.'). To investigate whether stimulus specific learning effects or generalized performance enhancements occur, the 16 words are divided into eight which are part of the GraphoGame training material and are potentially trained items, while the other half is not present in the game, nor in any other behavioural assessment or EEG paradigm. The 32 targets are split up into two levels with 16 trials and a time limit of three minutes each. As this is the longest in-game assessment and the performance in this task is very low at the start of first grade, it only appears at the manually or automatically triggered post-test in the game. Table 3 shows the distribution of words and pseudo words across the two levels.

4.4.2 Arithmetic assessments

Number knowledge

Analog to the letter sound identification task, children hear a spoken number and must pick the corresponding written number (made up by one or two digits) on the screen (Figure 25 on page 24). The provided instructions were 1) L*aten we nu zien welke cijfers je kent!* ('Let's see which numbers you know!'), or 2) *Laten we zien welke andere cijfers je kent!* ('Let's see which other numbers you know!'). We tested the knowledge of the numbers 0-20. Similar to written lexical decision assessment, to keep the time duration short and not crowd the screen too much, we split this assessment across two levels with 10 to 11 targets and a one minute time limit each. The distribution of targets across the two levels can be seen in Table 4.

Biggest dot

This and the following four assessments were inspired by subtests of the Nederlandse Dyscalculie Screener (NDS, Eng.: Dutch Dyscalculia Screener; Milikowski & Vermeire, 2013) and adapted for GG. In this task children see two dots of different sizes in the center of the screen in quick succession and must click as fast as possible on the one that is bigger (Figure 26 on page 24). Children heard the instruction *Kies de grootste stip!* ('Choose the biggest dot!'). This is not only an indicator of visual magnitude processing, but due to the low task demand also a great measure of how well children can

handle the computer mouse or touch screen to give responses. This level has a time limit of 45 seconds which never allows to respond to all 120 pairs of dots.

Counting dots

This task presents an image with dots at the top of the screen (Figure 27 on page 25). Children need to count the dots and pick the corresponding number out of four candidates displayed in the center of the screen. The following instruction was used to introduce the task: *Tel de stippen en kies het bijbehorende getal!* ('Count the dots and choose the corresponding number!'). The dots in the image appeared scattered in different shapes and sizes so that no two images for the same number looked alike. The number of dots presented visually ranged from two to six and distractors from one to seven. This level has a time limit of 45 seconds which never allows to respond to all 110 images with dots.

Bigger number - single digit

To compare amounts, this task displays two single digit numbers on the screen and the child needs to pick the bigger number (Figure 28 on page 25). The provided instructions were: *Kies het grootste getal! Probeer zo snel mogelijk te drukken!* ('Choose the biggest number! Try to respond as fast as you can!'). This level has a time limit of 45 seconds which never allows to respond to all 120 pairs of single digit numbers.

Bigger number - double digit

In line with the previous level this task contrasted two numbers with the same instructions as before. The only difference was that these were double digit numbers, only containing numbers which are not trained in the math game (i.e. bigger than 20), which makes this a suitable task to measure learning transfer or generalization effects (Figure 29 on page 26). This level has a time limit of 45 seconds which never allows to respond to all 120 pairs of double digit numbers.

Fill in missing number

In this task children see a row of three sequential adjacent numbers where the middle number is missing and needs to be selected from three alternatives displayed at the bottom (Figure 30 on page 26). The following instructions were given to the children: *In dit spel zie je blokken met een cijfer erin. Jij mag proberen de blokken met cijfers in de juiste volgorde te zetten.* ('In this game, you see blocks with numbers. You may try to put the blocks in the right order!'). Only numbers which are not trained in the math game (i.e. bigger than 20) are assessed. This level has a time limit of 45 seconds which never allows to respond to all 54 rows of numbers.

4.4.3 Assessment order and time limits

In total, the maximum (summed) time limit for the in-game assessments are 9:45 minutes for the pre-test and 15:45 minutes for the post-test including the written lexical decision task. While children may be done before the time limit in certain assessments, they usually spend some seconds on the labyrinth map in between, putting the length of the assessments in the range of 10 to 20 minutes. Within our experimental studies we made an effort to complete the entire assessment battery within the first and the last playing session to avoid splitting this across several days of gaming. Furthermore,

Table 1
Content of the easy letter knowledge assessment.

Level	Trials
1	d, e, r, i, n, k, a, v, oe, j
2	b, g, p, w, s, aa, z, ui, oo, ee, u
3	l, ij, h, ie, uu, f, eu, o, t, m, ou

Table 2
Content of the difficult letter knowledge assessment.

Trial	Target	Distractors
1	v	f u h n e
2	z	s m e r f
3	p	b t d w h
4	d	b p k f g
5	g	b d k h p
6	j	l s i r d
7	u	n v o w e
8	m	n l r w t
9	ij	eu ou oe ui ie
10	ou	eu oe ij ui ie

the sequential order of the assessments was not as described above, but mixed. We provide an outline of the actual order and time limits in Table 5.

4.5 Training material

The content for the reading game was determined based on the most common current teaching method in the Netherlands (Veilig leren lezen, Eng.: Learning to read safely; Mommers, Verhoeven, & van der Linden, 1990) as well as on a vocabulary achievement list of six-year old children (Schaerlaekens, Kohnstamm, Lajaegere, & Vries, 1999). This gave a list of over 1000 potential training target words. Around 650 items were ultimately selected for the training, ranging from simple and complex graphemes (e.g., ⟨n⟩, ⟨r⟩, ⟨ui⟩), over CV/VC syllables either representing separate words or occurring as parts in existing words (e.g. vi / is), to monosyllabic words with CVC structure (e.g., vis, Eng.: 'fish') or

Table 3
Content of the written lexical decision assessment.

Level	Trials
1	bos, kos, zoon, neus, ak, pe, al kels, as, dag, neul, hat, jas, ag, daf, zoof
2	toed, vis, hond, ke, kerk, zeup, te, mat, zeep, kem, hem, ves, de, tijd, jal, hend

Table 4
Content of the number knowledge assessment.

Level	Trials
1	1, 2, 9, 17, 11, 4, 19, 15, 14, 5
2	20, 3, 8, 16, 13, 0, 10, 6, 12, 18, 7

Table 5
Order and duration of in-game assessments.

Assessment	Session	Time limit
Easy Letter Sound Identification 1	Pre & Post	60
Number/Digit Knowledge 1	Pre & Post	60
Bigger Number - single digit	Pre & Post	45
Written Lexical Decision 1	Post	180
Easy Letter Sound Identification 2	Pre & Post	60
Counting Dots	Pre & Post	45
Easy Letter Sound Identification 3	Pre & Post	60
Number/Digit Knowledge 2	Pre & Post	60
Biggest Dot	Pre & Post	45
Bigger Number - double digit	Pre & Post	45
Written Lexical Decision 2	Post	180
Difficult Letter Sound Identification	Pre & Post	60
Fill in missing number	Pre & Post	45

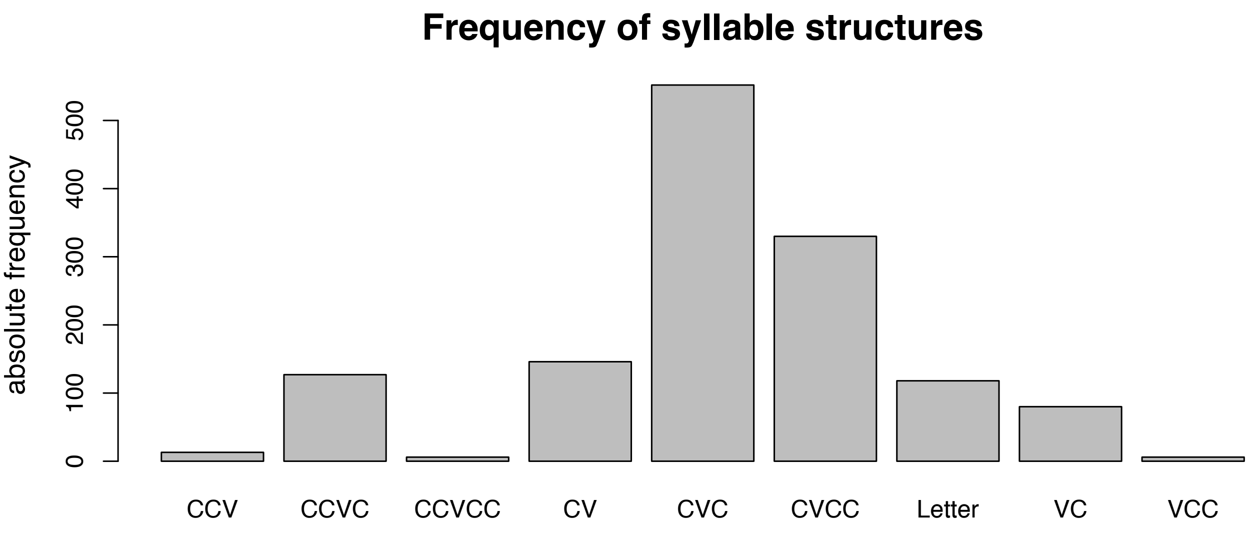

Figure 31. Frequency distribution of syllable structures of all training targets.

Table 6
Training content in alphabetical order.

Graphemes (N=32): a, aa, b, d, e, ee, eu, f, g, h, i, ie, ij, j, k, l, m, n, o, oe, oo, ou, p, r, s, t, u, ui, uu, v, w, z.

Words (N=323): aan, aap, aar, aas, al, baan, baas, bak, bakt, bank, been, beer, beest, bek, ben, bent, berg, bes, bij, bijt, bloem, blok, boe, boef, boen, bok, bom, boom, boon, boor, boos, boot, dan, daar, dag, dak, dan, dat, de, die, dik, dit, doe, doel, doen, doet, door, dorp, dus, duw, een, eens, eet, en, er, fee, fijn, film, fout, gaat, gans, gat, geel, geen, glad, goed, haak, haar, haas, hak, hakt, hals, heb, heel, heet, heg, helm, hem, het, hier, hij, hoe, hoek, hoofd, hoor, hoort, hou, hut, ik, in, is, je, jeuk, jij, jou, kaal, kaars, kaas, kan, kapt, kat, keer, kees, kerk, kers, kies, kijk, kijkt, kip, knie, knoop, koen, kok, kom, komt, kook, kookt, koop, kort, kun, kus, laars, laat, lam, lat, leeg, leuk, lief, lieg, ligt, lijn, loop, loopt, maak, maakt, maar, maat, mam, man, mand, me, mee, meer, mees, meet, mek, mens, mep, mest, met, mij, mijn, mis, moe, moet, mos, muts, muur, naam, naar, nat, nee, neem, neemt, nest, net, niet, noem, nog, nou, nu, of, om, ook, op, paar, pakt, park, pas, past, peen, peer, pen, pet, pier, pijn, pijp, pik, pikt, pink, pit, plat, plek, pols, poot, pot, poes, pret, prijs, put, raak, raap, raar, rek, ren, rent, reuk, riet, rij, rijk, rijst, rik, rit, roep, roept, roer, rook, room, ruik, rust, sip, sis, sla, slak, slee, snel, soep, som, sop, spin, spoor, staan, staat, stad, stier, teen, tel, tien, tijd, tikt, til, tip, toe, toon, tor, tot, traan, trui, uil, uit, uur, uw, vaak, vaar, van, veel, ver, vest, vier, vies, vijf, vis, vla, vlag, vlek, voer, vol, voor, vos, vraag, waar, warm, was, wat, we, web, weeg, week, weer, weet, weg, wel, wie, wiet, wijn, wijs, wijst, wil, win, wint, wip, wit, wolf, woon, woont, woord, worm, zalf, ze, zeg, zegt, zes, zet, zie, ziek, zien, ziet, zij, zijn, zit, zo, zoek, zoekt, zoem, zoemt, zoen, zoet, zoon, zout, zuur, zwaan, zwaar, zwart.

Sublexical units and pseudowords (N=285): ak, am, baa, baf, bap, bapt, bees, belm, bem, beus, biem, bijs, bim, birk, blaan, bluup, bor, bum, daap, dakt, darp, dast, dest, det, diel, dijf, din, domt, doog, doon, dug, duis, dust, ek, faa, feek, feu, fie, foe, foo, fook, fou, fui, fuim, fuin, fuu, fuuk, gaak, gaam, gan, gar, gee, geem, gem, germ, glen, gles, gluin, goo, goom, goun, guu, guum, hie, hies, hig, hijk, hoel, hoept, hon, hook, hoot, huin, hus, ief, ijf, ijt, it, jaam, jal, jeem, jek, jeug, jeut, jik, joo, joug, jui, juu, kaam, kag, ke, keekt, keem, kel, kelm, ker, kes, ket, kie, kiet, knees, knes, kui, kuirs, kum, kupt, kur, laap, laapt, larg, lee, leet, lent, leug, lie, lij, lim, lit, loe, lork, lort, luip, lut, mas, meef, meek, mel, miel, mijst, mil, mim, mit, mon, mookt, moon, moont, muif, muk, mun, ne, ner, nin, nit, noeg, noep, nof, noomt, nost, not, nui, nuist, ol, oons, oop, oos, ouf, ouk, oum, oz, paam, paat, pag, pank, peem, peets, pem, pijst, pir, plaas, pukt, purk, puu, raal, raan, rakt, ran, re, rer, res, rie, ries, riets, rijl, rin, roen, rom, sa, see, sek, sep, ses, seu, seuk, si, sie, sij, sin, sloek, snom, snur, souk, spen, spoer, stijt, stuin, sui, suim, sum, suu, taar, tan, tar, teeg, teem, tek, tekt, telk, tep, tet, ti, tie, tijp, treg, trijl, trup, ub, uf, uik, uim, uip, ul, uud, uug, uul, uun, uup, uus, vank, veef, veg, ves, vij, voem, vrook, vruuk, vus, waaf, wap, warg, weem, weep, werg, wes, weuns, wiem, wilf, woe, wog, woo, wop, wos, wot, wuik, wuil, wul, wut, wuu, wuur, zaap, zaas, zarm, zer, zest, zeu, zief, zijmt, zo, zop, zos, zour, zuikt, zunk, zut, zwijs.

Table 7
Training content in sequential order. C = Consonant, V = Vowel.

Stream	Levels	Structure	Content
1	9	C<br>V<br>CV<br>VC	n d k r<br>e<br>de ke ne re<br>en ek er
2	5	C<br>V<br>CV<br>VC	s t<br>a i<br>sa si ti<br>is it
3	6	C<br>V<br>CV<br>VC	h m v z<br>o<br>zo<br>om
4	6	C<br>V<br>CV<br>VC	b f l<br>aa ee u<br>baa faa lee<br>uf ul
5	8	C<br>V<br>CV<br>VC<br>CVC	r k n<br>e i<br>re<br>ak ek en ik in<br>dan dik rek ren rik
6	7	C<br>V<br>CV<br>VC	g j p w<br>oo uu<br>juu puu woo<br>ag oop uug uup
7	12	CV<br>VC<br>CVC	ik in<br>of<br>bom boom dak dan dat dik dit geel heel kan ker leeg nat ner rer rik rit sis tar weeg
8	34	CV<br>VC<br>CVC	je me nee nu we ze<br>aan aar aas al een eet ook op uur uw<br>aap baan baas bak been beer bek ben bes bok boon boor boot bos bus daan daar door dus duw gaat gat geen haak haar hak heb heet hem het hok hoor kaal kaas keer kees kip kok kom kook koop kun kus laat lam lat loop maak maar maat mam meer mees meet mek mep met mis mos muur naar neem net nog paar pas peen peer pen pet pik pit poot pot put raak raap raar rook saar sip som sop teen tel til tip toon tor tot vaak vaar van veel ver vis vol voor vos waar was wat weer weet wel wil win wip wit woon zeg zes zet zit zoon zuur
9	14	V<br>CV<br>VC<br>CVC	eu ie ij ou ui<br>feu fie foe fou fui hie jui kie kui lie lij mui nui rie seu sie sij soe sou sui tie vij woe zeu<br>ief ijk ijt ouf ouk oul oum uik uim uip<br>leeg lieg muur net niet zes ziek zon zoon zus zuur
10	23	CV<br>VC<br>CVC	bij boe die doe hij hoe hou jij jou mij moe nou poe rij toe wie zie zij<br>uil uit<br>bijt boef boen doel doen doet duis fijn fout goed goun hier hijk hoek huin jeuk jeut jij joug kies kiet kijk koen leug leuk lieg lief lijn luip miel miet mijn moet muif niet noeg noem pijn pijp reuk ries riet rijk roen roep roer ruik soep suim tiel tien tijd tijn tijp vier vies vijf voem voer wiem wiet wijn wijs wuik wuil zief zien ziep ziet zijn zijp zoek zoem zoet zon zour zout
11	6	CCV<br>CCVC<br>CVCC	sla trui vla<br>bloem blok glad knie plat plek pret prijs slak slee snel spin staan staat traan vlaag vlag vlek<br>bakt beest hakt kijkt mest nest past pikt pink
12	20	CVCC	bank bapt bent berg darp domt eens film gans germ hals helm hoept hoofd hoort kaars kapt kerk kers komt kookt kort kupt laapt laars larg lent ligt loopt lort maakt mens mookt moont muts neemt noomt oons pakt park peets pols rent riets rijst roept rust tekt telk tikt vest warg werg weuns wijst wilf wint wolf woont word worm zalf zarm zegt zijmt zoekt zoemt zwart
13-17	75		Repetition of streams 10, 11, 12, 11, 12

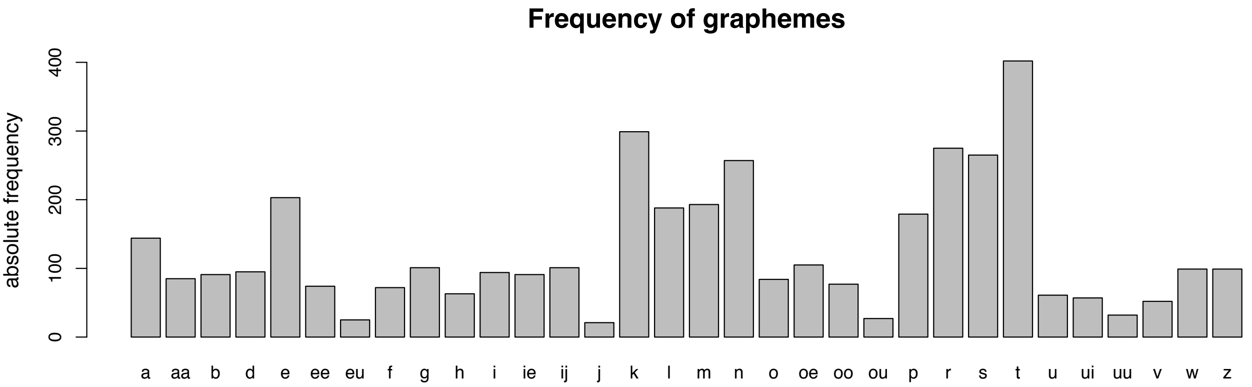

Figure 32. Frequency distribution of graphemes used across the entire training content.

targets with CCVC or CVCC consonant clusters (e.g., prijs, Eng.: 'price'; zwart, Eng.: 'black'). Here, we only consider lower case letters and did not intend to train upper case letters. Neither did we include all existing graphemes in Dutch, but excluded a few infrequent complex graphemes, which are not usually taught at the beginning of first grade (⟨sch⟩, ⟨ch⟩, ⟨ng⟩, ⟨au⟩, ⟨ei⟩), as well as complex diphthongs such as ⟨aai⟩, ⟨auw⟩, ⟨eeuw⟩, ⟨ieuw⟩, ⟨oei⟩, and ⟨ouw⟩.

For a limited number of target words, we also created phonotactically legal pseudo words as minimal pairs using the software Wuggy (Keuleers, & Brysbaert, 2010). Note that not all pseudo words are used as (auditory) targets but are mostly used as (visual) distractors. See Table 6 on page 34 for an alphabetical list of trained items and Table 7 on the previous page for their order of introduction. In addition, many syllables trained inside the game can be considered pseudowords, but they are mostly productive sublexical units which are part of existing words like ke or ker in *kerk* ('church') or *kers* ('cherry'). A more fine-grained impression of the characteristics of the game content can be found in the frequency distribution plots below (Figures 31 through 33). These only consider the auditory target but none of the distractors. A look at the syllable structure (Figure 31 on page 33) shows that single letters/graphemes are only a rather small portion of the game content comparable to the occurrence of CV and VC syllable training. The vast majority of content comes in the form of CVC or CVCC words and pseudowords. The combined frequency distribution of graphemes in isolation or as part of words (Figure 32) shows that the most frequent graphemes are the consonants ⟨k⟩ ⟨l⟩ ⟨m⟩ ⟨p⟩ ⟨r⟩ ⟨s⟩ ⟨t⟩ while graphemes such as ⟨j⟩ or ⟨ou⟩ occur rather rarely. Ultimately, we can split up the grapheme frequency by occurrence within the syllable structure while at the same time considering consonant clusters (Figure 33).

All auditory stimuli were recorded from five female students enrolled in the linguistics or speech and language pathology programs at the University of Groningen. The recordings were done in a sound attenuated chamber in an audio recordings studio of the university. Each speaker produced around 20 realizations of each target phoneme, and around five for syllables, words, and pseudo words. Subsequently, the same speakers made a pre-selection of the most prototypical realizations, reducing the number of candidates from up to 100 to a manageable range of 8-10 realizations. To reduce the remaining candidates even further, a perception experiment was set up in Praat (Boersma & Weenick, 2014). Another five students who were native speakers of Dutch and were not involved in previous stages, listened to pairs of phonemes and had to indicate which was more prototypical. Only the most prototypical items were used in the game, yielding one to four different spoken realizations per target.

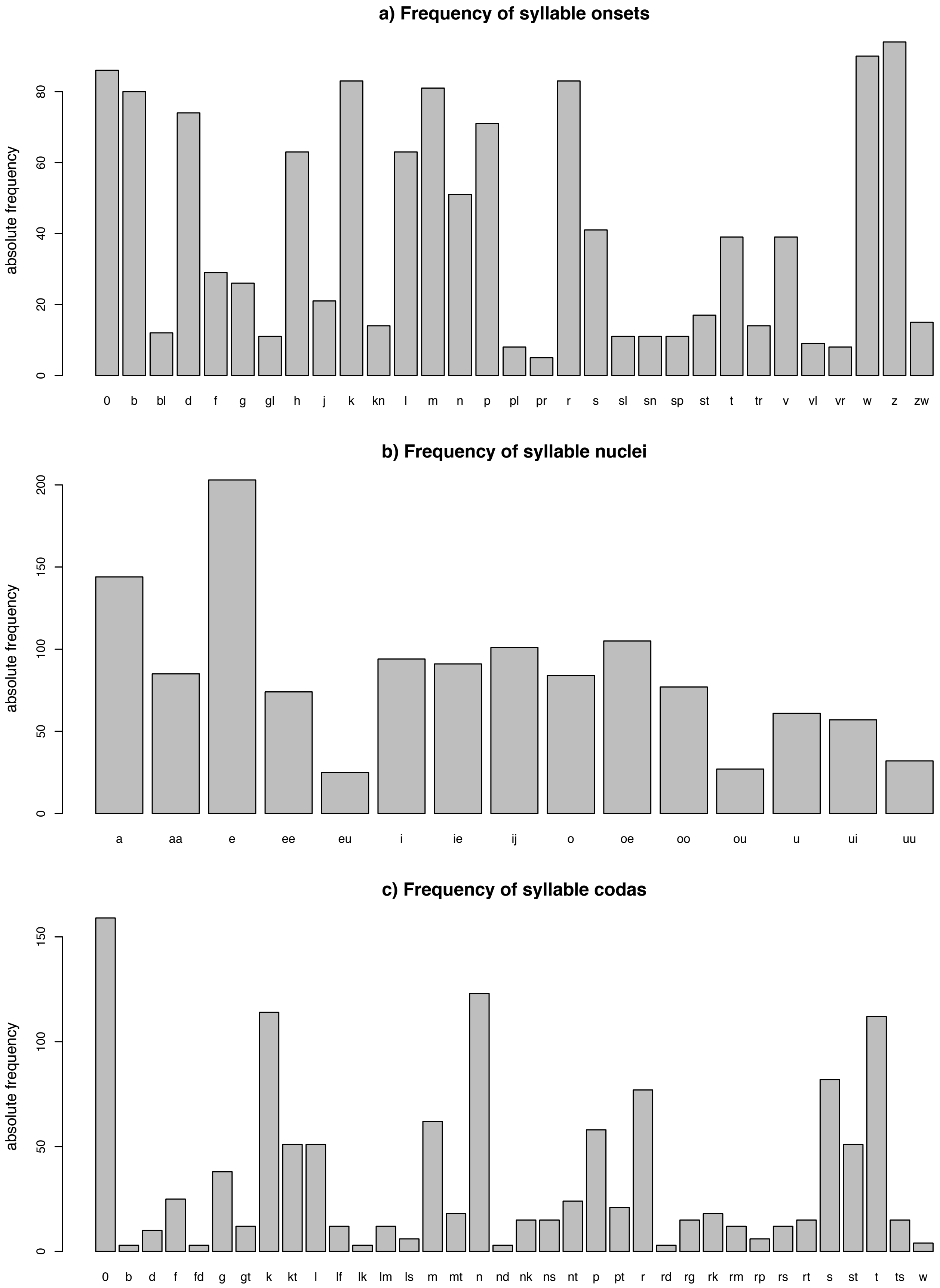

Figure 33. Frequency distribution of syllable onsets (a), nuclei (b), and codas (c) across the entire training content. Zero (0) refers to an empty onset or coda.

4.6 Level design

After creating a first theoretically motivated sequence through the available content, it went through several iterations of piloting with around 30 children enrolled in regular and special primary education. After daily playing for one week the game logs where checked to identify points within the sequence that were too difficult, and children were additionally asked if their recent experience of the game was boring or difficult. The content was accordingly rearranged several times until a sequence which was not too difficult, yet motivating enough to allow most children to play daily for at least five weeks, was established. The final sequence features 150 unique levels distributed across 12 so called 'streams', which are groups of levels that share a specific content or training goal (see Table 6 on page 34 for a full overview of all streams and their content). For instance, there are streams for the introduction of graphemes (e.g. streams 1, 2 & 5 for simple graphemes and stream 9 for complex graphemes), for reading of transparent CVC words (stream 8), or dedicated to reading of words with consonant clusters (streams 11 & 12). To extend the playing period for good readers, the levels of streams 10 through 12 were duplicated at the end to increase the total number of available levels to 265.

The philosophy behind the use of streams is that the game should start with a small inventory of highly frequent and transparent graphemes (3-5) which are auditorily and visually hardly confusable to facilitate the start for the children who are struggling with reading acquisition (Richardson & Lyytinen, 2014). Each of the initial streams starts with the introduction of a small set of graphemes (e.g. ⟨n d e r k⟩), first teaching grapheme-phoneme correspondences in isolation (⟨n⟩ /n/), and subsequently building up larger lexical (e.g.: en, de) and productive sublexical units (e.g.: ne, ke). Over time, the blending of phonemes goes beyond CV and VC and syllabic complexity increases to CVC from stream 5 onwards. Within each stream there are between 5 to 34 separate levels which train a mix of the tasks presented previously (Section 4.3). To make the most out of every set of items, we used the full range of game modules (or mini games) mentioned above (Section 4.2). The individual peculiarities of the different mini-games were also quite important to consider when deciding which game module should be used at what point of the game with what type of content. For instance, when introducing new targets, we chose a level with less distractors (e.g. the ladder game) and avoided moving text (e.g. within the fish game) and/or multiple targets/distractors (e.g. within the frog game). Because text movement or many distractors may be especially hard for struggling readers, these latter games were preferably used for material which children have already proven to know. Within the level sequence, such mini-games with a time limit gain more prominence from stream 10 onwards.

References

All, A., Castellar, E. P. N., & Van Looy, J. (2014). Measuring Effectiveness in Digital Game-Based Learning: A Methodological Review. *International Journal of Serious Games, 1*(2).

Anderson, R. C., Wilson, P. T., & Fielding, L. G. (1988). Growth in reading and how children spend their time outside of school. *Reading research quarterly,* 285-303.

Aro, M., & Wimmer, H. (2003). Learning to read: English in comparison to six more regular orthographies. *Applied psycholinguistics, 24*(4), 621-635.

Baker, S., Gersten, R., & Lee, D. S. (2002). A synthesis of empirical research on teaching mathematics to low-achieving students. *The Elementary School Journal, 103*(1), 51-73.

Blomert, L., & Willems, G. (2010). Is there a causal link from a phonological awareness deficit to reading failure in children at familial risk for dyslexia?. *Dyslexia, 16*(4), 300-317. doi: 10.1002/dys.405

Boersma, P., & Weenink, D. (2015). Praat [Computer Software]. Retrieved from: http://www.praat.org

Borleffs, E., Glatz, T. K., Daulay, D. A., Richardson, U., Zwarts, F., & Maassen, B. A. (2017). GraphoGame SI: the development of a technology-enhanced literacy learning tool for Standard Indonesian. *European Journal of Psychology of Education*, 1-19.

Brem, S., Bach, S., Kucian, K., Kujala, J. V., Guttorm, T. K., Martin, E., ... & Richardson, U. (2010). Brain sensitivity to print emerges when children learn letter–speech sound correspondences. *Proceedings of the National Academy of Sciences, 107*(17), 7939-7944. doi: 10.1073/pnas.0904402107

Broekhof, K. (2006). Preschool education in the Netherlands. Sardes Educational Service, SARDES, Utrecht.

Bus, A. G., (1990). Early reading acquisition. In P. Reitsma, & T. L. Verhoeven (Eds.), *Acquisition of reading in Dutch* (pp. 14-28). Walter de Gruyter.

Coenen, M. J. W. L., Van Bon, W. H. J., & Schreuder, R. (1997). Reading and spelling in Dutch first and second graders: do they use an orthographic strategy?. In *Cross-language studies of learning to read and spell* (pp. 249-269). Springer, Dordrecht.

Desoete, A., & Praet, M. (2013). Inclusive mathematics education: the value of a computerized look-ahead approach in kindergarten: a randomized controlled study. *Transylvanian Journal of Psychology, 14*, 103-119.

Dowker, A. (2005). Individual differences in arithmetic: Implications for psychology, neuroscience and education. Psychology Press.

Duncan, L. G., Castro, S. L., Defior, S., Seymour, P. H., Baillie, S., Leybaert, J., ... & Lund, R. (2013). Phonological development in relation to native language and literacy: Variations on a theme in six alphabetic orthographies. *Cognition, 127*(3), 398-419.

Gros, B. (2007). Digital games in education: The design of games-based learning environments. *Journal of research on technology in education, 40*(1), 23-38.

Goswami, U. (2002). Phonology, reading development, and dyslexia: A cross-linguistic perspective. *Annals of Dyslexia, 52*(1), 139-163.

Goswami, U., & Bryant, P. (1990). Essays in developmental psychology series. Phonological skills and learning to read. Hillsdale, NJ, US.

Huemer, S., Landerl, K., Aro, M., & Lyytinen, H. (2008). Training reading fluency among poor readers of German: Many ways to the goal. *Annals of Dyslexia, 58*(2), 115-137.

Hulme, C., Hatcher, P. J., Nation, K., Brown, A., Adams, J., & Stuart, G. (2002). Phoneme awareness is a better predictor of early reading skill than onset-rime awareness. *Journal of experimental child psychology, 82*(1), 2-28.

Kamykowska, J., Haman, E., Latvala, J. M., Richardson, U., & Lyytinen, H. (2014). Developmental changes of early reading skills in six-year-old Polish children and GraphoGame as a computer-based intervention to support them. *L1-Educational Studies in Language and Literature, 13*, 1-17. doi: 10.17239/L1ESLL-2013.01.05

Kanselaar, G., Vermeulen, C. J. A. J., & Kok, W. A. M. (1998). Voorspellen van leesuitval en achterblijvende leesontwikkeling: een onderzoek naar de predictieve validiteit van het leerrendementqoutient voor technisch lezen (LRQ) aan het einde van groep drie in de basisschool.

Keuleers, E., & Brysbaert, M. (2010). Wuggy: A multilingual pseudoword generator. *Behavior research methods, 42*(3), 627-633.

Kooreman, H. J., & Franken, M. I. (1976). Letterstad: transparantpapier. Wolters-Noordhoff.

Ktisti, C. (2015). Computer-based remediation for reading difficulties in a consistent orthography: comparing the effects of two theory-driven programs (Doctoral dissertation). Retrieved from the library of the University of Cyprus.

Kujala, J. V., Richardson, U., & Lyytinen, H. (2010). Estimation and visualization of confusability matrices from adaptive measurement data. Journal of *Mathematical Psychology, 54*(1), 196-207.

Kyle, F., Kujala, J., Richardson, U., Lyytinen, H., & Goswami, U. (2013). Assessing the effectiveness of two theoretically motivated computer-assisted reading interventions in the United Kingdom: GG Rime and GG Phoneme. *Reading Research Quarterly, 48*(1), 61-76.

Landerl, K., Fussenegger, B., Moll, K., & Willburger, E. (2009). Dyslexia and dyscalculia: Two learning disorders with different cognitive profiles. *Journal of experimental child psychology, 103*(3), 309-324.

Lyytinen, H., Ronimus, M., Alanko, A., Poikkeus, A. M., & Taanila, M. (2007). Early identification of dyslexia and the use of computer game-based practice to support reading acquisition. *Nordic Psychology, 59*(2), 109-126. ISO 690

Milikowski, M., and Vermeire, S. (2013). Nederlandse Dyscalculie Screener (NDS). Handleiding en Verantwoording, Amsterdam: Boom Testuitgevers.

Mommers, M. J. C., Verhoeven, L., & Van der Linden, S. (1990). Veilig leren lezen. Zwijsen, Tilburg.

OECD (2014). PISA 2012 Results: What Students Know and Can Do - Student Performance in Mathematics, Reading and Science. Volume I, Revised edition. OECD Publishing. http://dx.doi.org/10.1787/9789264201118-en

Ojanen, E., Kujala, J., Richardson, U., & Lyytinen, H. (2013). Technology-enhanced literacy learning in Zambia: Observations from a multilingual literacy environment. *Insights on Learning Disabilities: From Prevailing Theories to Validated Practices, 10*(2), 103-127.

Ojanen, E., Ronimus, M., Ahonen, T., Chansa-Kabali, T., February, P., Jere-Folotiya, J., ... & Puhakka, S. (2015). GraphoGame–a catalyst for multi-level promotion of literacy in diverse contexts. *Frontiers in psychology, 6*, 671. doi: 10.3389/fpsyg.2015.00671

Praet, M., & Desoete, A. (2014). Enhancing young children's arithmetic skills through non-intensive, computerised kindergarten interventions: A randomised controlled study. *Teaching and Teacher Education, 39*, 56-65.

Puolakanaho, A., Ahonen, T., Aro, M., Eklund, K., Leppänen, P. H., Poikkeus, A. M., ... & Lyytinen, H. (2007). Very early phonological and language skills: estimating individual risk of reading disability. *Journal of Child Psychology and Psychiatry, 48*(9), 923-931.

Reitsma, P., & Verhoeven, L. T. (Eds.). (1990). Acquisition of reading in Dutch (Vol. 9). Walter de Gruyter.

Richardson, U., & Lyytinen, H. (2014). The GraphoGame method: the theoretical and methodological background of the technology-enhanced learning environment for learning to read. *Human Technology: An Interdisciplinary Journal on Humans in ICT Environments, 10*(1), 39-60. doi: 10.17011/ht/urn 201405281859

Potocki, A., Ecalle, J., & Magnan, A. (2013). Effects of computer-assisted comprehension training in less skilled comprehenders in second grade: A one-year follow-up study. *Computers & Education, 63*, 131-140.

Räsänen, P., Salminen, J., Wilson, A. J., Aunio, P., & Dehaene, S. (2009). Computer-assisted intervention for children with low numeracy skills. *Cognitive Development, 24*(4), 450-472.

Rosas, R., Escobar, J. P., Ramírez, M. P., Meneses, A., & Guajardo, A. (2017). Impact of a computer-based intervention in Chilean children at risk of manifesting reading difficulties/Impacto de una intervención basada en ordenador en niños chilenos con riesgo de manifestar dificultades lectoras. *Infancia y Aprendizaje, 40*(1), 158-188.

Ruiz, J. P., Lassault, J., Sprenger-Charolles, L., Richardson, U., Lyytinen, H., & Ziegler, J. C. (2017). GraphoGame: un outil numerique pour enfant en difficultes d'apprentissage de la lecture. *ANAE Approche Neuropsychologique des Apprentissages chez l'Enfant, 148*, 333-343.

Ryan, R. M., & Deci, E. L. (2000). Intrinsic and extrinsic motivations: Classic definitions and new directions. *Contemporary educational psychology, 25*(1), 54-67.

Saine, N. L., Lerkkanen, M. K., Ahonen, T., Tolvanen, A., & Lyytinen, H. (2010). Predicting word-level reading fluency outcomes in three contrastive groups: Remedial and computer-assisted remedial reading intervention, and mainstream instruction. *Learning and Individual differences, 20*(5), 402-414. doi: 10.1016/j.lindif.2010.06.004

Saine, N. L., Lerkkanen, M. K., Ahonen, T., Tolvanen, A., & Lyytinen, H. (2011). Computer-assisted remedial reading intervention for school beginners at risk for reading disability. *Child Development, 82*(3), 1013-1028. doi: 10.1111/j.1467-8624.2011.01580.x

Schaerlaekens, A. M., Kohnstamm, G. A., Lejaegere, M., & Vries, A. K. (1999). Streeflijst woordenschat voor zesjarigen: gebaseerd op nieuw onderzoek in Nederland en België. Swets & Zeitlinger.

Seymour, P. H., Aro, M., Erskine, J. M., & collaboration with COST Action A8 network. (2003). Foundation literacy acquisition in European orthographies. *British Journal of psychology, 94*(2), 143-174.

Seymour, P. H., & Duncan, L. G. (1997). Small versus large unit theories of reading acquisition. *Dyslexia, 3*(3), 125-134.

Vaessen, A., & Blomert, L. (2010). Long-term cognitive dynamics of fluent reading development. *Journal of experimental child psychology, 105*(3), 213-231. ISO 690

Wilson, A. J., Revkin, S. K., Cohen, D., Cohen, L., & Dehaene, S. (2006). An open trial assessment of" The Number Race", an adaptive computer game for remediation of dyscalculia. *Behavioral and brain functions, 2*(1), 20.

Ziegler, J. C., & Goswami, U. (2005). Reading acquisition, developmental dyslexia, and skilled reading across languages: a psycholinguistic grain size theory. *Psychological bulletin, 131*(1), 3.
