# Supplementary material for: Dynamic assessment of the effectiveness of digital game-based literacy training in beginning readers: a cluster randomised controlled trial
Source: PeerJ. 2023 Jul 31;11:e15499. doi: 10.7717/peerj.15499 (PMC10399564; doi:10.7717/peerj.15499)
Supplement: Supplemental Information 4 — Output of the R markdown file. [file peerj-11-15499-s004.html]

Analysis script and documentation for Dynamic assessment of the effectiveness of digital game-based literacy training in beginning readers


Code 

- Show All Code
- Hide All Code

# Analysis script and documentation for *Dynamic assessment of the effectiveness of digital game-based literacy training in beginning readers*

#### Toivo Glatz1,2,3, Wim Tops1, Elisabeth Borleffs1,2, Ulla Richardson4, Natasha Maurits2,5, Annemie Desoete6, & Ben Maassen1,2,7;1Centre for Language and Cognition (CLCG),University of Groningen, The Netherlands, 2Behavioural and Cognitive Neuroscience (BCN), University of Groningen, The Netherlands, 3Charité – Universitätsmedizin Berlin, corporate member of Freie Universität Berlin and Humboldt-Universität zu Berlin, Institute of Public Health, 4Centre for Applied Language Studies, University of Jyväskylä, Finland, 5Department of Neurology, University of Groningen, the Netherlands, 6Department of Developmental Disorders, Ghent University, Belgium 7Department of Neuroscience, University Medical Center Groningen, University of Groningen, The Netherlands

#### Created on: June 21, 2021 - 10:45:18

# 1 Documentation & Analyses

## 1.1 Abstract

In this paper, we report on a study evaluating the effectiveness of a digital game-based learning (DGBL) tool for beginning readers of Dutch, employing active (math game) and passive (no game) control conditions. This classroom-level randomized control trial included 247 first graders from 16 classrooms in the Netherlands and the Dutch-speaking part of Belgium. The intervention consisted of 10 to 15 minutes of daily playing during school time for a period of 4 to 7 weeks. Our outcome measures included reading fluency, as well as purpose built in-game proficiency levels to measure written lexical decision and letter speech sound association. After an average of 28 playing sessions, the literacy game improved letter knowledge at a scale generalizable for all children in the classroom compared to the other two conditions. In addition to a small classroom wide benefit in terms of reading fluency, we furthermore discovered that children who scored high on phonological awareness prior to training were more fluent readers after extensive exposure to the reading game. This study is among the first to exploit game generated data for the evaluation of DGBL for literacy interventions.

Second revision submitted to **PeerJ** in May 2021.

## 1.2 R packages

The following R version and packages were used to generate this markdown:

```
sessionInfo()
```

```
## R version 4.0.4 (2021-02-15)
## Platform: x86_64-apple-darwin17.0 (64-bit)
## Running under: macOS Catalina 10.15.7
## 
## Matrix products: default
## BLAS:   /Library/Frameworks/R.framework/Versions/4.0/Resources/lib/libRblas.dylib
## LAPACK: /Library/Frameworks/R.framework/Versions/4.0/Resources/lib/libRlapack.dylib
## 
## locale:
## [1] en_US.UTF-8/en_US.UTF-8/en_US.UTF-8/C/en_US.UTF-8/en_US.UTF-8
## 
## attached base packages:
## [1] stats     graphics  grDevices utils     datasets  methods   base     
## 
## other attached packages:
##  [1] MuMIn_1.43.17     arsenal_3.6.2     Hmisc_4.5-0       ggplot2_3.3.3     Formula_1.2-4     survival_3.2-11   lattice_0.20-41   boot_1.3-26       psych_2.1.3       itsadug_2.4      
## [11] plotfunctions_1.4 mgcv_1.8-35       nlme_3.1-152      data.table_1.14.0 effects_4.2-0     carData_3.0-4     lmerTest_3.1-3    lme4_1.1-26       Matrix_1.3-2     
## 
## loaded via a namespace (and not attached):
##  [1] splines_4.0.4       tmvnsim_1.0-2       assertthat_0.2.1    statmod_1.4.35      stats4_4.0.4        latticeExtra_0.6-29 yaml_2.2.1          numDeriv_2016.8-1.1 pillar_1.6.0       
## [10] backports_1.2.1     glue_1.4.2          digest_0.6.27       RColorBrewer_1.1-2  checkmate_2.0.0     minqa_1.2.4         colorspace_2.0-1    htmltools_0.5.1.1   survey_4.0         
## [19] pkgconfig_2.0.3     purrr_0.3.4         scales_1.1.1        jpeg_0.1-8.1        tibble_3.1.1        htmlTable_2.1.0     generics_0.1.0      ellipsis_0.3.2      withr_2.4.2        
## [28] nnet_7.3-15         mnormt_2.0.2        magrittr_2.0.1      crayon_1.4.1        evaluate_0.14       fansi_0.4.2         MASS_7.3-54         foreign_0.8-81      tools_4.0.4        
## [37] mitools_2.4         lifecycle_1.0.0     stringr_1.4.0       munsell_0.5.0       cluster_2.1.0       compiler_4.0.4      rlang_0.4.11        grid_4.0.4          nloptr_1.2.2.2     
## [46] rstudioapi_0.13     htmlwidgets_1.5.3   base64enc_0.1-3     rmarkdown_2.8       gtable_0.3.0        DBI_1.1.1           R6_2.5.0            gridExtra_2.3       knitr_1.33         
## [55] dplyr_1.0.6         utf8_1.2.1          insight_0.14.0      stringi_1.5.3       parallel_4.0.4      Rcpp_1.0.6          vctrs_0.3.8         rpart_4.1-15        png_0.1-7          
## [64] tidyselect_1.1.1    xfun_0.22
```

## 1.3 Dataframes

Here we present the complete raw data of 286 children who initially consented to participate and who were subsequently allocated to a treatment condition via block randomization. Below are the reasons for exclusion, as stated in flowchart (Figure 1). **ADD** = attention deficit disorder, **ADHD** = attention deficit hyperactivity disorder, **age** = age above 8 years, **consentGAM** = parents only gave consent to play the game, but not to carry out psychometric testing at school, **DCD** = developmental coordination disorder, **no** = 247 participants included in the subsequent analyses, **noT2** = lost to follow up, **repeat** = repeated first grade, **sessions** = played less than 20 gaming sessions.

```
summary(BehavDat$Exclude)
```

```
##        ADD       ADHD        age consentGAM        DCD         no       noT2     repeat   sessions 
##          1          3          1          4          1        247          8          2         19
```

### 1.3.1 Offline assessments

Dataframe **BehavDat** contains the raw data based on questionnaires (e.g. familial risk for dyslexia) and psychometric test (e.g. CELF-IV-NL PA). Variable names and their descriptions are provided below.

#### 1.3.1.1 Variable Names

The dataset consists of 286 rows and 40 columns with the following column names:

```
str(BehavDat)
```

```
## 'data.frame':    286 obs. of  40 variables:
##  $ Subj          : Factor w/ 286 levels "1","2","3","4",..: 1 2 3 4 5 6 7 8 9 10 ...
##  $ School        : Factor w/ 8 levels "1","2","3","4",..: 1 1 1 1 1 1 1 1 1 1 ...
##  $ Class         : Factor w/ 16 levels "1","2","3","4",..: 11 11 11 11 11 11 11 11 11 11 ...
##  $ T1AgeF        : num  6.49 6.18 6.06 6.42 6.82 ...
##  $ Gender        : Factor w/ 2 levels "female","male": 2 2 2 2 2 1 1 2 2 2 ...
##  $ Exclude       : Factor w/ 9 levels "ADD","ADHD","age",..: 6 6 6 7 6 6 6 6 6 6 ...
##  $ Cond          : Factor w/ 3 levels "Math","Passive",..: 1 1 1 1 1 1 1 1 1 1 ...
##  $ HandScore     : num  0.1847 0.0154 0.5232 0.3539 0.0154 ...
##  $ FamRisk       : Factor w/ 2 levels "no","yes": 1 1 1 1 2 1 1 1 1 1 ...
##  $ Lang          : Factor w/ 2 levels "mono","multi": 1 1 1 1 1 1 1 1 1 1 ...
##  $ SON_1st       : num  0.075 -1.959 -0.739 -2.366 -0.739 ...
##  $ hoursPlayed   : num  2.73 3.35 3.01 3.23 3.16 ...
##  $ levelsPlayed  : int  332 386 304 374 423 345 230 391 265 422 ...
##  $ sessionsPlayed: int  25 26 25 24 26 27 27 26 25 26 ...
##  $ MaxLevel      : int  114 116 136 136 85 82 132 113 95 122 ...
##  $ itemsSeen     : int  5975 6849 5915 7119 7283 6413 4140 7010 5370 7581 ...
##  $ responsesGiven: int  2406 2867 2370 2930 3197 2668 1559 2911 2020 3262 ...
##  $ T2EMTaCor     : num  11 9 9 NA 6 12 11 16 15 10 ...
##  $ T2EMTbCor     : num  9 15 10 NA 4 12 6 15 21 11 ...
##  $ T1CF_Total    : int  6 10 31 11 19 25 29 31 26 28 ...
##  $ T1CF_TotalPc  : num  5 9 50 16 16 37 50 50 37 50 ...
##  $ T2CF_Total    : int  29 13 31 NA 29 29 37 35 22 33 ...
##  $ T2CF_TotalPc  : num  37 16 50 NA 16 37 63 75 25 50 ...
##  $ T1PF_Total    : int  30 23 33 22 29 30 30 24 27 34 ...
##  $ T1PF_TotalPc  : num  37.5 12.5 55 10 32.5 37.5 37.5 12.5 22.5 55 ...
##  $ T2PF_Total    : int  34 30 31 NA 34 36 35 33 32 36 ...
##  $ T2PF_TotalPc  : num  55 37.5 42.5 NA 47.5 75 62.5 55 47.5 75 ...
##  $ T1RANcT       : int  73 105 70 80 73 100 65 81 104 57 ...
##  $ T1RANoT       : int  88 88 61 95 117 88 78 89 95 72 ...
##  $ T2RANcT       : int  56 62 50 NA 78 62 62 76 64 46 ...
##  $ T2RANoT       : int  71 80 44 NA 85 82 80 91 72 80 ...
##  $ T2.LetKen     : int  NA NA NA NA NA NA 27 NA NA NA ...
##  $ T2.LexDec     : int  NA NA NA NA NA NA 17 NA NA NA ...
##  $ T1.LetKen     : int  5 0 15 6 8 3 5 6 4 8 ...
##  $ Country       : Factor w/ 2 levels "B","NL": 1 1 1 1 1 1 1 1 1 1 ...
##  $ EMT           : num  10 12 9.5 NA 5 12 8.5 15.5 18 10.5 ...
##  $ T1CF_TotalZ   : num  -1.971 -1.566 0.556 -1.465 -0.657 ...
##  $ T1PF_TotalZ   : num  -0.148 -1.391 0.384 -1.568 -0.326 ...
##  $ T1.LetKenZ    : num  -1.0362 -1.5995 0.0905 -0.9235 -0.6982 ...
##  $ LK            : int  NA NA NA NA NA NA 22 NA NA NA ...
```

```
summary(BehavDat)
```

```
##       Subj         School       Class         T1AgeF         Gender          Exclude         Cond       HandScore       FamRisk      Lang        SON_1st         hoursPlayed     levelsPlayed   
##  1      :  1   6      :71   4      : 31   Min.   :5.460   female:123   no        :247   Math   :117   Min.   :-3.2006   no :241   mono :250   Min.   :-2.3655   Min.   :1.161   Min.   :  87.0  
##  2      :  1   4      :50   1      : 27   1st Qu.:5.989   male  :163   sessions  : 19   Passive: 58   1st Qu.: 0.1847   yes: 45   multi: 36   1st Qu.:-0.7385   1st Qu.:2.620   1st Qu.: 210.8  
##  3      :  1   5      :37   10     : 25   Median :6.296                noT2      :  8   Read   :111   Median : 0.3539                         Median : 0.0750   Median :3.243   Median : 280.0  
##  4      :  1   3      :32   9      : 24   Mean   :6.271                consentGAM:  4                 Mean   : 0.0000                         Mean   : 0.0000   Mean   :3.323   Mean   : 308.8  
##  5      :  1   7      :31   8      : 22   3rd Qu.:6.500                ADHD      :  3                 3rd Qu.: 0.5232                         3rd Qu.: 0.4818   3rd Qu.:4.146   3rd Qu.: 363.8  
##  6      :  1   1      :28   2      : 19   Max.   :8.047                repeat    :  2                 Max.   : 0.5232                         Max.   : 4.9561   Max.   :5.236   Max.   :1026.0  
##  (Other):280   (Other):37   (Other):138                                (Other)   :  3                                                         NA's   :4         NA's   :58      NA's   :58      
##  sessionsPlayed     MaxLevel        itemsSeen     responsesGiven    T2EMTaCor       T2EMTbCor       T1CF_Total     T1CF_TotalPc     T2CF_Total    T2CF_TotalPc     T1PF_Total     T1PF_TotalPc   
##  Min.   :11.00   Min.   : 24.00   Min.   : 2010   Min.   :  628   Min.   : 0.00   Min.   : 0.00   Min.   : 0.00   Min.   : 0.10   Min.   : 2.0   Min.   : 0.10   Min.   : 0.00   Min.   :  0.00  
##  1st Qu.:24.00   1st Qu.: 89.25   1st Qu.: 5987   1st Qu.: 2079   1st Qu.: 8.00   1st Qu.: 7.00   1st Qu.:18.25   1st Qu.:25.00   1st Qu.:28.0   1st Qu.:37.00   1st Qu.:28.00   1st Qu.: 32.50  
##  Median :26.00   Median :132.00   Median : 8448   Median : 2850   Median :13.00   Median :13.00   Median :27.00   Median :37.00   Median :34.0   Median :63.00   Median :31.00   Median : 52.50  
##  Mean   :26.17   Mean   :126.37   Mean   : 9431   Mean   : 3113   Mean   :15.29   Mean   :14.53   Mean   :25.50   Mean   :44.41   Mean   :32.5   Mean   :58.24   Mean   :30.84   Mean   : 51.93  
##  3rd Qu.:30.00   3rd Qu.:150.00   3rd Qu.:12516   3rd Qu.: 3586   3rd Qu.:19.00   3rd Qu.:18.25   3rd Qu.:33.00   3rd Qu.:63.00   3rd Qu.:38.0   3rd Qu.:75.00   3rd Qu.:35.00   3rd Qu.: 75.00  
##  Max.   :37.00   Max.   :265.00   Max.   :22782   Max.   :10877   Max.   :45.00   Max.   :45.00   Max.   :44.00   Max.   :99.00   Max.   :44.0   Max.   :99.00   Max.   :40.00   Max.   :100.00  
##  NA's   :58      NA's   :58       NA's   :58      NA's   :58      NA's   :14      NA's   :14      NA's   :8       NA's   :8       NA's   :14     NA's   :14      NA's   :5       NA's   :5       
##    T2PF_Total    T2PF_TotalPc       T1RANcT          T1RANoT          T2RANcT          T2RANoT         T2.LetKen       T2.LexDec       T1.LetKen    Country       EMT          T1CF_TotalZ     
##  Min.   :13.0   Min.   :  0.00   Min.   : 39.00   Min.   : 40.00   Min.   : 34.00   Min.   : 39.00   Min.   : 3.00   Min.   : 8.00   Min.   : 0.0   B :191   Min.   : 0.000   Min.   :-2.5770  
##  1st Qu.:31.0   1st Qu.: 42.50   1st Qu.: 56.00   1st Qu.: 63.00   1st Qu.: 47.00   1st Qu.: 57.00   1st Qu.:19.00   1st Qu.:18.00   1st Qu.: 7.0   NL: 95   1st Qu.: 8.375   1st Qu.:-0.7327  
##  Median :34.0   Median : 55.00   Median : 65.00   Median : 72.00   Median : 55.00   Median : 66.00   Median :25.00   Median :21.00   Median :13.0            Median :12.500   Median : 0.1516  
##  Mean   :33.7   Mean   : 61.77   Mean   : 67.78   Mean   : 75.82   Mean   : 56.52   Mean   : 69.65   Mean   :23.42   Mean   :20.74   Mean   :14.2            Mean   :14.923   Mean   : 0.0000  
##  3rd Qu.:37.0   3rd Qu.: 90.00   3rd Qu.: 78.00   3rd Qu.: 85.50   3rd Qu.: 63.00   3rd Qu.: 79.00   3rd Qu.:29.00   3rd Qu.:24.00   3rd Qu.:23.0            3rd Qu.:18.500   3rd Qu.: 0.7579  
##  Max.   :40.0   Max.   :100.00   Max.   :122.00   Max.   :180.00   Max.   :103.00   Max.   :240.00   Max.   :32.00   Max.   :30.00   Max.   :30.0            Max.   :45.000   Max.   : 1.8696  
##  NA's   :13     NA's   :13       NA's   :5        NA's   :7        NA's   :13       NA's   :16       NA's   :79      NA's   :81      NA's   :1               NA's   :14       NA's   :8        
##   T1PF_TotalZ         T1.LetKenZ            LK         
##  Min.   :-5.47241   Min.   :-1.5995   Min.   :-16.000  
##  1st Qu.:-0.50335   1st Qu.:-0.8108   1st Qu.:  3.000  
##  Median : 0.02905   Median :-0.1348   Median :  7.000  
##  Mean   : 0.00000   Mean   : 0.0000   Mean   :  7.908  
##  3rd Qu.: 0.73892   3rd Qu.: 0.9919   3rd Qu.: 13.000  
##  Max.   : 1.62625   Max.   : 1.7806   Max.   : 27.000  
##  NA's   :5          NA's   :1         NA's   :80
```

#### 1.3.1.2 Variable Description

- Subj - unique subject ID
- School - unique school ID
- Class - unique classroom ID
- Country - country: Netherlands (NL) or Belgium (B)
- T1AgeF - fractional age in years at pretest (T1)
- Gender - gender (male/female)
- Exclude - exclusion criteria (e.g. ADHD or not enough sessions). Participants with Exclude==“no” are used for analyses
- Cond - gaming condition (Passive, Math, Read)
- HandScore - handedness score based on average of 11 questions (z-transformed)
- FamRisk - self reported familal risk for dyslexia
- Lang - language spoken at home (monolingual/multilingual)
- SON\_1st - analogies and categories subtest of SON-R 6-40 IQ test (z-transformed)
- hoursPlayed - time on task (read & math only)
- levelsPlayed - number of played levels (read & math only)
- sessionsPlayed - number of played sessions (read & math only)
- MaxLevel - highest level that was played (read & math only)
- MaxLevelS - highest level that was played (as above, but z-transformed)
- itemsSeen - total number of items seen during gameplay (read & math only)
- responsesGiven - number of responses give during gameplay (read & math only)
- T1CF\_Total - CELF IV NL: total test score at T1 (raw scores 0-45)
- T1CF\_TotalPc - CELF IV NL: total test score at T1 (percentile 0-100)
- T2CF\_Total - CELF IV NL: total test score at T2 (raw scores 0-45)
- T2CF\_TotalPc - CELF IV NL: total test score at T2 (percentiles 0-100)
- T1PF\_Total - PREOF PA: total test score at T1 (raw scores 0-10)
- T1PF\_TotalPc - PREOF PA: total test score at T1 (percentiles 0-100)
- T2PF\_Total - PREOF PA: total test score at T2 (raw scores 0-10)
- T2PF\_TotalPc - PREOF PA:total test score at T2 (percentiles 0-100)
- T1RANcT - Rapid Automatized Naming of colours at T1 (time in seconds)
- T1RANoT - Rapid Automatized Naming of objects at T1 (time in seconds)
- T2RANcT - Rapid Automatized Naming of colours at T2 (time in seconds)
- T2RANoT - Rapid Automatized Naming of objects at T2 (time in seconds)
- T2.LetKen - Computerized letter speech sound identification at T2 (raw scores 0-32)
- T1.LetKen - Computerized letter speech sound identification at T1 (raw scores 0-32)
- T2.LexDec - Computerized written lexical decision at T2 (raw scores 0-32)
- EMT - One minute reading fluency scores at T2 (raw scores in correctly read words per minute)

#### 1.3.1.3 Reliability of offline measures

##### 1.3.1.3.1 Reading fluency

```
readingdata <- droplevels(subset(BehavDat,EMT<100))
readingdata <- subset(readingdata, select=c(T2EMTaCor, T2EMTbCor))
(r = cor(readingdata$T2EMTaCor,readingdata$T2EMTbCor)) # 0.93 correlation between two lists
```

```
## [1] 0.9281803
```

```
((2 * r) / (1 + r)) # 0.96 split half reliability with Spearman Brown correction
```

```
## [1] 0.9627526
```

```
psych::alpha(readingdata) # 0.96 based on 272 children
```

```
## 
## Reliability analysis   
## Call: psych::alpha(x = readingdata)
## 
##   raw_alpha std.alpha G6(smc) average_r S/N    ase mean sd median_r
##       0.96      0.96    0.93      0.93  26 0.0046   15 10     0.93
## 
##  lower alpha upper     95% confidence boundaries
## 0.95 0.96 0.97 
## 
##  Reliability if an item is dropped:
##           raw_alpha std.alpha G6(smc) average_r S/N alpha se var.r med.r
## T2EMTaCor      1.00      0.93    0.86      0.93  13       NA     0  0.93
## T2EMTbCor      0.86      0.93    0.86      0.93  13       NA     0  0.93
## 
##  Item statistics 
##             n raw.r std.r r.cor r.drop mean   sd
## T2EMTaCor 272  0.98  0.98  0.95   0.93   15 10.5
## T2EMTbCor 272  0.98  0.98  0.95   0.93   15  9.8
```

### 1.3.2 Online assessments

Dataframe **inGameDat** contains the raw data from the in-game assessments that took place during the very first and the very last gaming session.

#### 1.3.2.1 Variable Names

The dataset consists of 25134 rows and 57 columns with the following additional variables:

```
str(inGameDat[1:18])
```

```
## 'data.frame':    25134 obs. of  18 variables:
##  $ idLevel            : int  3984112 3984112 3984112 3984112 4140137 4140137 4140137 4140137 4140137 4140137 ...
##  $ stream             : Factor w/ 2 levels "T1","T2": 1 1 1 1 2 2 2 2 2 2 ...
##  $ lvl                : Factor w/ 3 levels "LetKenDifficult",..: 1 1 1 1 2 2 2 2 2 2 ...
##  $ selectionsCounts   : num  10 10 10 10 10 10 10 10 10 10 ...
##  $ selectionsCorrect  : num  1 1 1 1 10 10 10 10 10 10 ...
##  $ selectionsIncorrect: num  9 9 9 9 0 0 0 0 0 0 ...
##  $ idTrial            : int  21196635 21196632 21196633 21196634 22792572 22792573 22792574 22792575 22792576 22792577 ...
##  $ result             : Factor w/ 2 levels "Correct","null": 2 2 2 2 1 1 1 1 1 1 ...
##  $ itemCountOnScreen  : int  6 6 6 6 10 10 10 10 10 10 ...
##  $ correctResponse    : Factor w/ 33 levels "a","aa","b","correct",..: 5 31 33 24 5 6 25 12 19 16 ...
##  $ playerResponse     : Factor w/ 34 levels "a","aa","b","correct",..: 16 19 6 5 5 6 25 12 19 16 ...
##  $ correctSelections  : Factor w/ 2 levels "0","1": 1 1 1 1 2 2 2 2 2 2 ...
##  $ incorrectSelections: num  1 1 1 1 0 0 0 0 0 0 ...
##  $ itemsOnScreen      : chr  "k;d;g;p;f;b" "h;n;u;e;v;f" "e;s;r;f;m;z" "p;d;h;b;t;w" ...
##  $ trialRTs           : num  3.591 1.467 0.284 4.676 1.962 ...
##  $ TrialNum           : int  4 1 2 3 1 2 3 4 5 6 ...
##  $ prevRT             : num  4.676 NA 1.467 0.284 NA ...
##  $ Subj               : Factor w/ 286 levels "1","2","3","4",..: 70 70 70 70 70 70 70 70 70 70 ...
```

```
summary(inGameDat[1:18])
```

```
##     idLevel        stream                  lvl        selectionsCounts selectionsCorrect selectionsIncorrect    idTrial             result      itemCountOnScreen correctResponse playerResponse 
##  Min.   :3977791   T1:10562   LetKenDifficult: 4635   Min.   : 1.00    Min.   : 0.000    Min.   : 0.00       Min.   :21131607   Correct:15953   Min.   : 3.000    correct: 6444   correct: 4270  
##  1st Qu.:3985919   T2:14572   LetKenEasy     :14055   1st Qu.:10.00    1st Qu.: 5.000    1st Qu.: 2.00       1st Qu.:21210963   null   : 9181   1st Qu.: 3.000    g      :  949   wrong  : 2174  
##  Median :4082142              LexDec         : 6444   Median :11.00    Median : 8.000    Median : 4.00       Median :22172822                   Median :10.000    p      :  949   z      :  826  
##  Mean   :4076320                                      Mean   :11.52    Mean   : 7.425    Mean   : 4.09       Mean   :22130302                   Mean   : 7.853    d      :  938   g      :  794  
##  3rd Qu.:4117278                                      3rd Qu.:16.00    3rd Qu.:10.000    3rd Qu.: 6.00       3rd Qu.:22541745                   3rd Qu.:11.000    z      :  933   ij     :  737  
##  Max.   :4188568                                      Max.   :16.00    Max.   :16.000    Max.   :11.00       Max.   :23299834                   Max.   :11.000    ij     :  927   d      :  735  
##                                                                                                                                                                   (Other):13994   (Other):15598  
##  correctSelections incorrectSelections itemsOnScreen         trialRTs         TrialNum          prevRT            Subj      
##  0: 9150           Min.   :0.000       Length:25134       Min.   : 0.001   Min.   : 1.000   Min.   : 0.001   30     :  116  
##  1:15984           1st Qu.:0.000       Class :character   1st Qu.: 1.816   1st Qu.: 3.000   1st Qu.: 1.806   40     :  116  
##                    Median :0.000       Mode  :character   Median : 2.901   Median : 6.000   Median : 2.867   48     :  116  
##                    Mean   :0.364                          Mean   : 3.735   Mean   : 6.227   Mean   : 3.649   54     :  116  
##                    3rd Qu.:1.000                          3rd Qu.: 4.569   3rd Qu.: 9.000   3rd Qu.: 4.504   62     :  116  
##                    Max.   :1.000                          Max.   :44.408   Max.   :16.000   Max.   :44.408   76     :  116  
##                                                                                             NA's   :2407     (Other):24438
```

#### 1.3.2.2 Variable Description

- idLevel - ingame level ID
- stream - pre- or post test measure (T1/T2)
- lvl - ingame level name
- selectionsCounts - number of targets/distractors selected in a given level ID
- selectionsCorrect - number of correctly selected targets in a given level ID
- selectionsIncorrect - number of mistakenly selected distractors in a given level ID
- idTirla - ingame trial ID
- result - outcome of a single trial/click (correct/null[=incorrect])
- itemCountOnScreen - sum of target+distractors displayed on screen in a givent rial
- correctResponse - the correct response to the task the child has to solve
- playerResponse - the actual response to the task by the child
- correctSelections - number of correctly selected targets in a given trial ID
- incorrectSelections - number of mistakenly selected distractors in a given trial ID
- itemsOnScreen - character representation of the order of graphemes on screen. Written lexical decision task additional encodes order of response buttons (correct/wrong)
- trialRTs - response time of a given trial ID (in seconds)
- TrialNum - sequential number of a given trial ID within a given level ID
- prevRT - response time to the previous trial ID

#### 1.3.2.3 Reliability of online assessments

This section computes the reliability for the letter speech sound identification (LSSI) and written lexical decision (WLD) tasks based on all available data at pre-test. We present Cronbach’s alpha as a measure for internal consistency, as well as split-half reliability. Furthermore, we present values for item based reliability and aggregated by level. Although Henson (2001) suggested that speeded tests violate the assumption of measuring a single underlying construct, we can consider speed as part of the construct of interest (GPC automation and reading fluency).

##### 1.3.2.3.1 LSSI aggregated by level

```
# Letter speech sound identification. Aggregated by level
# Establish level variable to sort each trial into the level it belongs to
inGameDat$LSSI[inGameDat$itemsOnScreen=="k;d;e;oe;a;n;j;v;r;i"] <- "LK1"
inGameDat$LSSI[inGameDat$itemsOnScreen=="s;ee;b;aa;p;oo;u;w;g;ui;z"] <- "LK2"
inGameDat$LSSI[inGameDat$itemsOnScreen=="uu;m;l;f;h;t;ou;ie;ij;o;eu"] <- "LK3"
inGameDat$LSSI[inGameDat$lvl=="LetKenDifficult"] <- "LK4"
inGameDat$LSSI <- as.factor(inGameDat$LSSI)
inGameDat$correctSelections <- as.integer(inGameDat$correctSelections) - 1
# We have isolated cases of double pre-test assessments. Here we filter those out and only take the first.
assessments <- aggregate(correctSelections~idLevel+Subj+LSSI,subset(inGameDat,stream=="T1"), FUN=sum)
firstAssessed <- aggregate(idLevel~Subj+LSSI, assessments,FUN=min)
assessment <- subset(assessments, idLevel %in% firstAssessed$idLevel)

# internal consistency with cronbach's alpha
LSSI <- reshape(assessment, idvar = "Subj", timevar = "LSSI", direction = "wide")[,c(3,5,7,9)]
LSSIn <- LSSI[complete.cases(LSSI),]
#a = (4 / (4 - 1)) * (1 - (var(LSSIn$correctSelections.LK1) + var(LSSIn$correctSelections.LK2) + var(LSSIn$correctSelections.LK3)+ var(LSSIn$correctSelections.LK4)) / var (LSSIn$correctSelections.LK1  +LSSIn$correctSelections.LK2 + LSSIn$correctSelections.LK3 + LSSIn$correctSelections.LK4)) # compute alpha from formula

score_e <- rowMeans(LSSIn[, c(TRUE, FALSE)],na.rm=T)  # with even items
score_o <- rowMeans(LSSIn[, c(FALSE, TRUE)],na.rm=T)  # with odd items
r <- cor(score_e, score_o)
(2 * r) / (1 + r) # 0.72 split half reliability with Spearman Brown correction
```

```
## [1] 0.9078268
```

```
psych::alpha(LSSI[complete.cases(LSSI),]) # 0.68 Cronbach's alpha
```

```
## 
## Reliability analysis   
## Call: psych::alpha(x = LSSI[complete.cases(LSSI), ])
## 
##   raw_alpha std.alpha G6(smc) average_r S/N    ase mean  sd median_r
##        0.9       0.9    0.88       0.7 9.3 0.0096  4.8 2.7     0.69
## 
##  lower alpha upper     95% confidence boundaries
## 0.88 0.9 0.92 
## 
##  Reliability if an item is dropped:
##                       raw_alpha std.alpha G6(smc) average_r S/N alpha se   var.r med.r
## correctSelections.LK1      0.87      0.87    0.82      0.70 6.9    0.013 0.00033  0.70
## correctSelections.LK2      0.86      0.86    0.81      0.68 6.3    0.015 0.00027  0.68
## correctSelections.LK3      0.87      0.88    0.83      0.70 7.1    0.013 0.00201  0.70
## correctSelections.LK4      0.88      0.89    0.84      0.72 7.7    0.012 0.00080  0.72
## 
##  Item statistics 
##                         n raw.r std.r r.cor r.drop mean  sd
## correctSelections.LK1 270  0.89  0.88  0.83   0.79  5.3 3.5
## correctSelections.LK2 270  0.90  0.90  0.86   0.82  5.4 3.3
## correctSelections.LK3 270  0.87  0.88  0.82   0.78  4.0 2.9
## correctSelections.LK4 270  0.85  0.86  0.79   0.75  4.6 2.8
```

---

##### 1.3.2.3.2 LSSI by item

```
# Letter speech sound identification. Single trial data
itemanalysis <- subset(inGameDat, idLevel %in% firstAssessed$idLevel & stream=="T1")

items <- aggregate(correctSelections~correctResponse+Subj,itemanalysis,FUN=sum)
item <- reshape(items, idvar = "Subj", timevar = "correctResponse", direction = "wide")[,-1]

score_e <- rowMeans(item[, c(TRUE, FALSE)],na.rm=T)  # with even items
score_o <- rowMeans(item[, c(FALSE, TRUE)],na.rm=T)  # with odd items
r <- cor(score_e, score_o)
(2 * r) / (1 + r) # 0.87 split half reliability with Spearman Brown correction
```

```
## [1] 0.9180898
```

```
psych::alpha(item) # 0.9 Cronbach's alpha
```

```
## 
## Reliability analysis   
## Call: psych::alpha(x = item)
## 
##   raw_alpha std.alpha G6(smc) average_r S/N    ase mean   sd median_r
##       0.93      0.94    0.95      0.31  14 0.0054 0.63 0.34     0.31
## 
##  lower alpha upper     95% confidence boundaries
## 0.92 0.93 0.94 
## 
##  Reliability if an item is dropped:
##                      raw_alpha std.alpha G6(smc) average_r S/N alpha se var.r med.r
## correctSelections.aa      0.93      0.93    0.94      0.31  14   0.0056 0.012  0.30
## correctSelections.b       0.93      0.93    0.94      0.31  14   0.0056 0.012  0.30
## correctSelections.d       0.93      0.93    0.94      0.31  14   0.0056 0.012  0.30
## correctSelections.e       0.93      0.93    0.94      0.30  14   0.0057 0.011  0.30
## correctSelections.ee      0.93      0.93    0.94      0.31  14   0.0056 0.012  0.30
## correctSelections.eu      0.93      0.93    0.94      0.31  14   0.0055 0.012  0.31
## correctSelections.f       0.93      0.93    0.94      0.31  14   0.0055 0.012  0.31
## correctSelections.g       0.93      0.93    0.94      0.30  13   0.0058 0.011  0.30
## correctSelections.h       0.93      0.93    0.94      0.31  14   0.0056 0.012  0.30
## correctSelections.i       0.93      0.93    0.94      0.31  14   0.0055 0.012  0.31
## correctSelections.ie      0.93      0.93    0.94      0.32  14   0.0054 0.012  0.31
## correctSelections.ij      0.93      0.93    0.94      0.31  14   0.0056 0.012  0.30
## correctSelections.j       0.93      0.93    0.94      0.30  13   0.0058 0.011  0.30
## correctSelections.k       0.93      0.93    0.94      0.31  14   0.0055 0.012  0.31
## correctSelections.l       0.93      0.93    0.94      0.32  14   0.0054 0.012  0.32
## correctSelections.m       0.93      0.93    0.94      0.31  14   0.0054 0.012  0.31
## correctSelections.n       0.93      0.93    0.94      0.31  14   0.0055 0.012  0.31
## correctSelections.o       0.93      0.93    0.95      0.32  14   0.0054 0.012  0.32
## correctSelections.oo      0.93      0.93    0.94      0.31  14   0.0055 0.012  0.31
## correctSelections.ou      0.93      0.93    0.94      0.31  14   0.0054 0.012  0.31
## correctSelections.p       0.93      0.93    0.94      0.32  14   0.0053 0.012  0.32
## correctSelections.r       0.93      0.93    0.94      0.31  14   0.0056 0.012  0.30
## correctSelections.s       0.93      0.94    0.95      0.32  14   0.0054 0.012  0.32
## correctSelections.t       0.93      0.93    0.94      0.30  13   0.0057 0.011  0.30
## correctSelections.u       0.93      0.93    0.94      0.31  14   0.0056 0.012  0.30
## correctSelections.ui      0.93      0.93    0.94      0.31  14   0.0056 0.012  0.30
## correctSelections.uu      0.93      0.94    0.95      0.32  15   0.0054 0.011  0.32
## correctSelections.v       0.93      0.93    0.94      0.31  14   0.0057 0.012  0.30
## correctSelections.w       0.93      0.93    0.94      0.31  14   0.0056 0.012  0.31
## correctSelections.z       0.93      0.93    0.94      0.31  14   0.0056 0.013  0.30
## correctSelections.a       0.93      0.93    0.94      0.31  14   0.0055 0.012  0.31
## correctSelections.oe      0.93      0.93    0.94      0.31  14   0.0055 0.012  0.31
## 
##  Item statistics 
##                        n raw.r std.r r.cor r.drop mean   sd
## correctSelections.aa 265  0.64  0.64  0.63   0.60 0.58 0.49
## correctSelections.b  281  0.62  0.63  0.62   0.60 0.57 0.50
## correctSelections.d  283  0.65  0.62  0.61   0.59 0.89 0.77
## correctSelections.e  270  0.74  0.74  0.74   0.71 0.47 0.50
## correctSelections.ee 190  0.68  0.69  0.69   0.66 0.54 0.50
## correctSelections.eu 247  0.46  0.48  0.45   0.44 0.21 0.41
## correctSelections.f  259  0.52  0.55  0.53   0.50 0.36 0.48
## correctSelections.g  282  0.77  0.75  0.75   0.74 0.88 0.83
## correctSelections.h  280  0.65  0.65  0.63   0.62 0.40 0.49
## correctSelections.i  250  0.47  0.48  0.46   0.43 0.68 0.47
## correctSelections.ie 279  0.44  0.45  0.43   0.41 0.27 0.44
## correctSelections.ij 281  0.65  0.62  0.61   0.60 0.77 0.80
## correctSelections.j  274  0.76  0.76  0.76   0.74 0.91 0.83
## correctSelections.k  227  0.46  0.48  0.46   0.43 0.81 0.39
## correctSelections.l  282  0.44  0.45  0.42   0.39 0.57 0.50
## correctSelections.m  262  0.49  0.49  0.46   0.44 1.00 0.78
## correctSelections.n  242  0.61  0.60  0.59   0.57 0.67 0.47
## correctSelections.o  235  0.39  0.42  0.39   0.36 0.71 0.45
## correctSelections.oo 217  0.55  0.55  0.54   0.51 0.74 0.44
## correctSelections.ou 244  0.50  0.48  0.46   0.45 0.45 0.67
## correctSelections.p  282  0.47  0.44  0.41   0.40 0.83 0.82
## correctSelections.r  260  0.62  0.62  0.61   0.58 0.71 0.46
## correctSelections.s  273  0.38  0.41  0.38   0.35 0.84 0.36
## correctSelections.t  213  0.73  0.75  0.75   0.73 0.50 0.50
## correctSelections.u  265  0.64  0.63  0.62   0.61 0.70 0.72
## correctSelections.ui 239  0.64  0.63  0.62   0.59 0.54 0.50
## correctSelections.uu 274  0.32  0.33  0.29   0.28 0.23 0.42
## correctSelections.v  284  0.70  0.68  0.67   0.65 1.00 0.82
## correctSelections.w  275  0.63  0.64  0.63   0.61 0.37 0.48
## correctSelections.z  282  0.64  0.61  0.59   0.58 1.18 0.81
## correctSelections.a  215  0.58  0.60  0.58   0.55 0.59 0.49
## correctSelections.oe 179  0.56  0.56  0.54   0.52 0.64 0.48
## 
## Non missing response frequency for each item
##                         0    1    2 miss
## correctSelections.aa 0.42 0.58 0.00 0.07
## correctSelections.b  0.43 0.57 0.00 0.01
## correctSelections.d  0.36 0.40 0.25 0.01
## correctSelections.e  0.53 0.47 0.00 0.05
## correctSelections.ee 0.46 0.54 0.00 0.33
## correctSelections.eu 0.79 0.21 0.00 0.13
## correctSelections.f  0.64 0.36 0.00 0.09
## correctSelections.g  0.41 0.29 0.29 0.01
## correctSelections.h  0.60 0.40 0.00 0.02
## correctSelections.i  0.32 0.68 0.00 0.12
## correctSelections.ie 0.73 0.27 0.00 0.02
## correctSelections.ij 0.46 0.31 0.23 0.01
## correctSelections.j  0.40 0.30 0.30 0.04
## correctSelections.k  0.19 0.81 0.00 0.20
## correctSelections.l  0.43 0.57 0.00 0.01
## correctSelections.m  0.30 0.39 0.31 0.08
## correctSelections.n  0.33 0.67 0.00 0.15
## correctSelections.o  0.29 0.71 0.00 0.18
## correctSelections.oo 0.26 0.74 0.00 0.24
## correctSelections.ou 0.66 0.24 0.10 0.14
## correctSelections.p  0.43 0.31 0.26 0.01
## correctSelections.r  0.29 0.71 0.00 0.09
## correctSelections.s  0.16 0.84 0.00 0.04
## correctSelections.t  0.50 0.50 0.00 0.25
## correctSelections.u  0.45 0.39 0.15 0.07
## correctSelections.ui 0.46 0.54 0.00 0.16
## correctSelections.uu 0.77 0.23 0.00 0.04
## correctSelections.v  0.33 0.33 0.33 0.00
## correctSelections.w  0.63 0.37 0.00 0.04
## correctSelections.z  0.25 0.32 0.43 0.01
## correctSelections.a  0.41 0.59 0.00 0.25
## correctSelections.oe 0.36 0.64 0.00 0.37
```

---

##### 1.3.2.3.3 Written Lexical Decision aggregated by level

```
# We have to subset data and remove bos and kos
WLDreliab <- droplevels(subset(inGameDat, lvl=="LexDec"))
WLDreliab <- subset(WLDreliab, itemsOnScreen != "bos;correct;wrong" & itemsOnScreen !="kos;wrong;correct")

# Check how many unique levelIDs per subject
WLDlevels <- aggregate(idLevel~Subj,WLDreliab,FUN=function(x) NROW(unique(x))) # only one subject has 3 times this level #StM105 - solution: remove trial 3991226

WLDreliab <- subset(WLDreliab, idLevel != "3991226") # only 2 levels per person!
# now split up in min and max level per person
WLDlevel <- aggregate(idLevel~Subj,WLDreliab,FUN=min)
WLDlevel$max <- aggregate(idLevel~Subj,WLDreliab,FUN=max)$idLevel
WLDlevel$diff <- WLDlevel$max - WLDlevel$idLevel
WLDinclude <- droplevels(subset(WLDlevel, diff>0)$Subj)

WLDrelia <- droplevels(subset(WLDreliab, Subj %in% WLDinclude))
WLDassess <- aggregate(correctSelections~Subj+idLevel,WLDrelia,FUN=sum)

WLDassess$lvl <- "2"
WLDassess$lvl[WLDassess$idLevel %in% WLDlevel$idLevel] <- "1"
WLDassess$lvl <- as.factor(WLDassess$lvl)
WLDassess$idLevel <- NULL
WLD <- reshape(WLDassess, idvar = "Subj", timevar = "lvl", direction = "wide")[,-1]
r <- cor(WLD$correctSelections.1,WLD$correctSelections.2)
((2 * r) / (1 + r)) # 0.65 split-half reliability with Spearman Brown correction
```

```
## [1] 0.6394148
```

```
psych::alpha(WLD) # 0.65 Cronbach's alpha
```

```
## 
## Reliability analysis   
## Call: psych::alpha(x = WLD)
## 
##   raw_alpha std.alpha G6(smc) average_r S/N   ase mean sd median_r
##       0.64      0.64    0.47      0.47 1.8 0.051  9.6  2     0.47
## 
##  lower alpha upper     95% confidence boundaries
## 0.54 0.64 0.74 
## 
##  Reliability if an item is dropped:
##                     raw_alpha std.alpha G6(smc) average_r  S/N alpha se var.r med.r
## correctSelections.1      0.42      0.47    0.22      0.47 0.89       NA     0  0.47
## correctSelections.2      0.53      0.47    0.22      0.47 0.89       NA     0  0.47
## 
##  Item statistics 
##                       n raw.r std.r r.cor r.drop mean  sd
## correctSelections.1 199  0.84  0.86  0.59   0.47  8.8 2.2
## correctSelections.2 199  0.88  0.86  0.59   0.47 10.4 2.5
```

---

##### 1.3.2.3.4 Written Lexical Decision by item

```
items <- aggregate(correctSelections~itemsOnScreen+Subj,WLDreliab,FUN=sum)
item <- reshape(items, idvar = "Subj", timevar = "itemsOnScreen", direction = "wide")[,-1]

score_e <- rowMeans(item[, c(TRUE, FALSE)],na.rm=T)  # with even items
score_o <- rowMeans(item[, c(FALSE, TRUE)],na.rm=T)  # with odd items
r <- cor(score_e, score_o)
(2 * r) / (1 + r) # 0.65 split half reliability with Spearman Brown correction
```

```
## [1] 0.6522993
```

```
psych::alpha(item,check.keys=TRUE) # Cronbach's alpha # 0.68
```

```
## 
## Reliability analysis   
## Call: psych::alpha(x = item, check.keys = TRUE)
## 
##   raw_alpha std.alpha G6(smc) average_r S/N   ase mean   sd median_r
##       0.68      0.68    0.74     0.066 2.1 0.031 0.64 0.14    0.067
## 
##  lower alpha upper     95% confidence boundaries
## 0.62 0.68 0.74 
## 
##  Reliability if an item is dropped:
##                                       raw_alpha std.alpha G6(smc) average_r S/N alpha se  var.r med.r
## correctSelections.ag;wrong;correct         0.68      0.68    0.74     0.067 2.1    0.031 0.0103 0.067
## correctSelections.ak;wrong;correct         0.67      0.67    0.73     0.064 2.0    0.033 0.0097 0.065
## correctSelections.al;correct;wrong         0.68      0.68    0.74     0.067 2.1    0.031 0.0101 0.068
## correctSelections.as;correct;wrong-        0.68      0.67    0.74     0.066 2.1    0.032 0.0100 0.067
## correctSelections.bem;wrong;correct        0.67      0.66    0.73     0.064 2.0    0.033 0.0100 0.065
## correctSelections.daf;wrong;correct        0.67      0.67    0.74     0.065 2.0    0.032 0.0101 0.066
## correctSelections.dag;correct;wrong        0.68      0.67    0.74     0.066 2.1    0.032 0.0100 0.066
## correctSelections.de;correct;wrong         0.68      0.67    0.74     0.066 2.1    0.032 0.0100 0.068
## correctSelections.hat;wrong;correct-       0.69      0.69    0.75     0.070 2.2    0.030 0.0099 0.072
## correctSelections.hem;correct;wrong-       0.69      0.69    0.75     0.071 2.2    0.030 0.0096 0.073
## correctSelections.hend;wrong;correct       0.67      0.67    0.74     0.065 2.0    0.032 0.0099 0.066
## correctSelections.hond;correct;wrong       0.69      0.68    0.74     0.069 2.2    0.030 0.0095 0.068
## correctSelections.jal;wrong;correct        0.66      0.66    0.73     0.063 1.9    0.033 0.0098 0.065
## correctSelections.jas;correct;wrong        0.68      0.68    0.74     0.067 2.1    0.031 0.0101 0.068
## correctSelections.ke;wrong;correct         0.67      0.66    0.73     0.063 1.9    0.033 0.0099 0.062
## correctSelections.kels;wrong;correct       0.67      0.67    0.73     0.065 2.0    0.032 0.0098 0.067
## correctSelections.kerk;correct;wrong       0.68      0.68    0.74     0.068 2.1    0.031 0.0097 0.067
## correctSelections.mat;correct;wrong        0.68      0.68    0.74     0.069 2.1    0.031 0.0101 0.068
## correctSelections.neul;wrong;correct       0.66      0.66    0.73     0.062 1.9    0.033 0.0100 0.062
## correctSelections.neus;correct;wrong       0.68      0.67    0.73     0.066 2.0    0.032 0.0098 0.067
## correctSelections.pe;wrong;correct         0.67      0.66    0.73     0.062 1.9    0.033 0.0098 0.062
## correctSelections.te;correct;wrong-        0.68      0.68    0.74     0.068 2.1    0.031 0.0100 0.068
## correctSelections.tijd;correct;wrong-      0.69      0.69    0.75     0.071 2.2    0.030 0.0095 0.072
## correctSelections.toed;wrong;correct       0.67      0.66    0.73     0.063 2.0    0.033 0.0097 0.065
## correctSelections.ves;wrong;correct        0.66      0.65    0.72     0.061 1.9    0.034 0.0098 0.062
## correctSelections.vis;correct;wrong        0.68      0.67    0.74     0.066 2.1    0.032 0.0098 0.068
## correctSelections.zeep;correct;wrong       0.68      0.67    0.74     0.067 2.1    0.032 0.0098 0.068
## correctSelections.zeup;wrong;correct       0.65      0.65    0.72     0.060 1.9    0.034 0.0096 0.061
## correctSelections.zoof;wrong;correct       0.67      0.66    0.73     0.064 2.0    0.033 0.0101 0.063
## correctSelections.zoon;correct;wrong       0.69      0.69    0.75     0.070 2.2    0.031 0.0099 0.069
## 
##  Item statistics 
##                                         n raw.r std.r    r.cor   r.drop mean   sd
## correctSelections.ag;wrong;correct    204 0.278 0.256  0.19277  0.15690 0.58 0.49
## correctSelections.ak;wrong;correct    204 0.398 0.389  0.36422  0.29914 0.74 0.44
## correctSelections.al;correct;wrong    204 0.270 0.260  0.21142  0.15394 0.48 0.50
## correctSelections.as;correct;wrong-   204 0.298 0.288  0.23713  0.19484 0.53 0.50
## correctSelections.bem;wrong;correct   197 0.405 0.392  0.35904  0.29989 0.55 0.50
## correctSelections.daf;wrong;correct   202 0.362 0.362  0.31545  0.26339 0.54 0.50
## correctSelections.dag;correct;wrong   203 0.283 0.292  0.24021  0.18496 0.82 0.38
## correctSelections.de;correct;wrong    199 0.271 0.300  0.25321  0.18152 0.81 0.39
## correctSelections.hat;wrong;correct-  203 0.117 0.113  0.02773 -0.00043 0.67 0.47
## correctSelections.hem;correct;wrong-  199 0.084 0.080 -0.00066 -0.01901 0.27 0.44
## correctSelections.hend;wrong;correct  199 0.349 0.332  0.28882  0.23706 0.49 0.50
## correctSelections.hond;correct;wrong  199 0.158 0.156  0.09002  0.04020 0.58 0.50
## correctSelections.jal;wrong;correct   199 0.479 0.452  0.43828  0.37906 0.49 0.50
## correctSelections.jas;correct;wrong   204 0.241 0.248  0.19339  0.14561 0.80 0.40
## correctSelections.ke;wrong;correct    199 0.442 0.441  0.41885  0.34877 0.72 0.45
## correctSelections.kels;wrong;correct  204 0.391 0.358  0.32933  0.28195 0.59 0.49
## correctSelections.kerk;correct;wrong  199 0.233 0.240  0.19247  0.12589 0.71 0.46
## correctSelections.mat;correct;wrong   199 0.152 0.188  0.11550  0.07572 0.88 0.32
## correctSelections.neul;wrong;correct  204 0.490 0.481  0.46748  0.39163 0.55 0.50
## correctSelections.neus;correct;wrong  204 0.313 0.318  0.28570  0.20894 0.71 0.46
## correctSelections.pe;wrong;correct    204 0.448 0.474  0.45946  0.38127 0.81 0.39
## correctSelections.te;correct;wrong-   199 0.234 0.234  0.17766  0.12518 0.68 0.47
## correctSelections.tijd;correct;wrong- 199 0.102 0.087  0.00346 -0.00971 0.34 0.48
## correctSelections.toed;wrong;correct  198 0.446 0.433  0.41241  0.34237 0.54 0.50
## correctSelections.ves;wrong;correct   199 0.529 0.516  0.51673  0.43206 0.52 0.50
## correctSelections.vis;correct;wrong   198 0.236 0.286  0.24482  0.17923 0.93 0.25
## correctSelections.zeep;correct;wrong  199 0.244 0.279  0.24111  0.17393 0.89 0.31
## correctSelections.zeup;wrong;correct  199 0.568 0.557  0.56551  0.48380 0.59 0.49
## correctSelections.zoof;wrong;correct  202 0.413 0.401  0.36866  0.30714 0.70 0.46
## correctSelections.zoon;correct;wrong  205 0.128 0.132  0.05033  0.02824 0.76 0.43
## 
## Non missing response frequency for each item
##                                         0    1 miss
## correctSelections.ag;wrong;correct   0.42 0.58 0.00
## correctSelections.ak;wrong;correct   0.26 0.74 0.00
## correctSelections.al;correct;wrong   0.52 0.48 0.00
## correctSelections.as;correct;wrong   0.53 0.47 0.00
## correctSelections.bem;wrong;correct  0.45 0.55 0.04
## correctSelections.daf;wrong;correct  0.46 0.54 0.01
## correctSelections.dag;correct;wrong  0.18 0.82 0.01
## correctSelections.de;correct;wrong   0.19 0.81 0.03
## correctSelections.hat;wrong;correct  0.67 0.33 0.01
## correctSelections.hem;correct;wrong  0.27 0.73 0.03
## correctSelections.hend;wrong;correct 0.51 0.49 0.03
## correctSelections.hond;correct;wrong 0.42 0.58 0.03
## correctSelections.jal;wrong;correct  0.51 0.49 0.03
## correctSelections.jas;correct;wrong  0.20 0.80 0.00
## correctSelections.ke;wrong;correct   0.28 0.72 0.03
## correctSelections.kels;wrong;correct 0.41 0.59 0.00
## correctSelections.kerk;correct;wrong 0.29 0.71 0.03
## correctSelections.mat;correct;wrong  0.12 0.88 0.03
## correctSelections.neul;wrong;correct 0.45 0.55 0.00
## correctSelections.neus;correct;wrong 0.29 0.71 0.00
## correctSelections.pe;wrong;correct   0.19 0.81 0.00
## correctSelections.te;correct;wrong   0.68 0.32 0.03
## correctSelections.tijd;correct;wrong 0.34 0.66 0.03
## correctSelections.toed;wrong;correct 0.46 0.54 0.03
## correctSelections.ves;wrong;correct  0.48 0.52 0.03
## correctSelections.vis;correct;wrong  0.07 0.93 0.03
## correctSelections.zeep;correct;wrong 0.11 0.89 0.03
## correctSelections.zeup;wrong;correct 0.41 0.59 0.03
## correctSelections.zoof;wrong;correct 0.30 0.70 0.01
## correctSelections.zoon;correct;wrong 0.24 0.76 0.00
```

## 1.4 Analyses

### 1.4.1 Descriptives

Descriptives for the 247 children included in the analyses by Condition, Country, Gender and Familal Risk.

#### 1.4.1.1 Tables by Condition, Country, Gender and status of familial risk

```
TableDat <- droplevels(subset(BehavDat, Exclude == "no"))

tab <- tableby(Cond ~ EMT + FamRisk + Country + T1AgeF + HandScore + Gender + Lang + SON_1st + hoursPlayed + levelsPlayed + sessionsPlayed + MaxLevel + itemsSeen + responsesGiven + T1CF_TotalPc + T1PF_TotalPc + T1.LetKen + T1RANcT + T1RANoT, data = TableDat, numeric.stats=c("meansd","medianrange"))
summary(tab, text=TRUE, digits=2, test=F)
```

```
## 
## 
## |                  |        Math (N=104)         |    Passive (N=52)     |         Read (N=91)          |        Total (N=247)        |
## |:-----------------|:---------------------------:|:---------------------:|:----------------------------:|:---------------------------:|
## |EMT               |                             |                       |                              |                             |
## |-  Mean (SD)      |        17.34 (11.34)        |     10.05 (4.59)      |         15.43 (9.30)         |        15.08 (9.85)         |
## |-  Median (Range) |     14.75 (1.00, 45.00)     |  10.50 (2.50, 22.00)  |     13.50 (1.00, 45.00)      |     12.50 (1.00, 45.00)     |
## |FamRisk           |                             |                       |                              |                             |
## |-  no             |         86 (82.7%)          |      45 (86.5%)       |          75 (82.4%)          |         206 (83.4%)         |
## |-  yes            |         18 (17.3%)          |       7 (13.5%)       |          16 (17.6%)          |         41 (16.6%)          |
## |Country           |                             |                       |                              |                             |
## |-  B              |         56 (53.8%)          |      52 (100.0%)      |          53 (58.2%)          |         161 (65.2%)         |
## |-  NL             |         48 (46.2%)          |       0 (0.0%)        |          38 (41.8%)          |         86 (34.8%)          |
## |T1AgeF            |                             |                       |                              |                             |
## |-  Mean (SD)      |         6.28 (0.31)         |      6.20 (0.30)      |         6.23 (0.34)          |         6.25 (0.32)         |
## |-  Median (Range) |      6.32 (5.55, 7.07)      |   6.17 (5.68, 6.80)   |      6.23 (5.46, 7.39)       |      6.26 (5.46, 7.39)      |
## |HandScore         |                             |                       |                              |                             |
## |-  Mean (SD)      |         0.09 (0.94)         |     -0.27 (1.27)      |         -0.01 (0.96)         |        -0.02 (1.03)         |
## |-  Median (Range) |     0.35 (-3.20, 0.52)      |  0.35 (-3.20, 0.52)   |      0.35 (-3.20, 0.52)      |     0.35 (-3.20, 0.52)      |
## |Gender            |                             |                       |                              |                             |
## |-  female         |         44 (42.3%)          |      21 (40.4%)       |          47 (51.6%)          |         112 (45.3%)         |
## |-  male           |         60 (57.7%)          |      31 (59.6%)       |          44 (48.4%)          |         135 (54.7%)         |
## |Lang              |                             |                       |                              |                             |
## |-  mono           |         89 (85.6%)          |      46 (88.5%)       |          87 (95.6%)          |         222 (89.9%)         |
## |-  multi          |         15 (14.4%)          |       6 (11.5%)       |           4 (4.4%)           |         25 (10.1%)          |
## |SON_1st           |                             |                       |                              |                             |
## |-  Mean (SD)      |         0.11 (0.96)         |     -0.29 (0.99)      |         0.17 (1.00)          |         0.05 (0.99)         |
## |-  Median (Range) |     0.08 (-2.37, 3.33)      |  -0.54 (-2.37, 1.70)  |      0.08 (-2.37, 4.96)      |     0.08 (-2.37, 4.96)      |
## |hoursPlayed       |                             |                       |                              |                             |
## |-  Mean (SD)      |         3.38 (0.89)         |          NA           |         3.59 (0.84)          |         3.48 (0.87)         |
## |-  Median (Range) |      3.14 (1.96, 5.24)      |          NA           |      3.61 (1.68, 5.23)       |      3.40 (1.68, 5.24)      |
## |levelsPlayed      |                             |                       |                              |                             |
## |-  Mean (SD)      |       401.81 (153.29)       |          NA           |        235.14 (61.40)        |       324.03 (145.51)       |
## |-  Median (Range) |  358.50 (132.00, 1026.00)   |          NA           |   232.00 (101.00, 377.00)    |  289.00 (101.00, 1026.00)   |
## |sessionsPlayed    |                             |                       |                              |                             |
## |-  Mean (SD)      |        26.74 (3.96)         |          NA           |         27.93 (4.94)         |        27.30 (4.47)         |
## |-  Median (Range) |    26.00 (18.00, 35.00)     |          NA           |     27.00 (16.00, 37.00)     |    26.00 (16.00, 37.00)     |
## |MaxLevel          |                             |                       |                              |                             |
## |-  Mean (SD)      |       136.42 (27.86)        |          NA           |        125.19 (59.63)        |       131.18 (45.75)        |
## |-  Median (Range) |   136.00 (39.00, 178.00)    |          NA           |    109.00 (38.00, 265.00)    |   135.00 (38.00, 265.00)    |
## |itemsSeen         |                             |                       |                              |                             |
## |-  Mean (SD)      |      8857.08 (4167.68)      |          NA           |      11036.49 (3918.98)      |      9874.14 (4187.51)      |
## |-  Median (Range) | 7666.00 (2827.00, 22782.00) |          NA           | 10838.00 (2983.00, 20113.00) | 8778.00 (2827.00, 22782.00) |
## |responsesGiven    |                             |                       |                              |                             |
## |-  Mean (SD)      |      3817.48 (2040.07)      |          NA           |       2671.35 (810.57)       |      3282.62 (1686.15)      |
## |-  Median (Range) | 3249.00 (1019.00, 10877.00) |          NA           |  2626.00 (948.00, 4522.00)   | 2943.00 (948.00, 10877.00)  |
## |T1CF_TotalPc      |                             |                       |                              |                             |
## |-  Mean (SD)      |        48.40 (24.21)        |     41.14 (23.91)     |        47.41 (25.05)         |        46.53 (24.52)        |
## |-  Median (Range) |     50.00 (1.00, 91.00)     |  37.00 (1.00, 95.00)  |     37.00 (5.00, 99.00)      |     50.00 (1.00, 99.00)     |
## |T1PF_TotalPc      |                             |                       |                              |                             |
## |-  Mean (SD)      |        54.52 (29.52)        |     49.52 (25.94)     |        56.07 (27.95)         |        54.04 (28.22)        |
## |-  Median (Range) |    55.00 (0.00, 100.00)     |  47.50 (0.00, 97.50)  |     55.00 (0.00, 100.00)     |    55.00 (0.00, 100.00)     |
## |T1.LetKen         |                             |                       |                              |                             |
## |-  Mean (SD)      |        16.35 (9.48)         |     11.27 (5.30)      |         14.08 (9.53)         |        14.46 (8.97)         |
## |-  Median (Range) |     15.50 (0.00, 30.00)     |  10.00 (0.00, 25.00)  |     13.00 (0.00, 30.00)      |     13.50 (0.00, 30.00)     |
## |T1RANcT           |                             |                       |                              |                             |
## |-  Mean (SD)      |        63.32 (14.50)        |     70.88 (15.92)     |        68.46 (17.23)         |        66.81 (16.09)        |
## |-  Median (Range) |    61.50 (40.00, 107.00)    | 67.50 (48.00, 115.00) |    66.00 (39.00, 122.00)     |    65.00 (39.00, 122.00)    |
## |T1RANoT           |                             |                       |                              |                             |
## |-  Mean (SD)      |        73.55 (16.51)        |     75.56 (17.49)     |        75.05 (21.26)         |        74.53 (18.55)        |
## |-  Median (Range) |    70.00 (40.00, 126.00)    | 76.50 (46.00, 127.00) |    72.00 (43.00, 180.00)     |    72.00 (40.00, 180.00)    |
```

```
tab <- tableby(Country ~ EMT + FamRisk + Cond + T1AgeF + HandScore + Gender + Lang + SON_1st + hoursPlayed + levelsPlayed + sessionsPlayed + MaxLevel + itemsSeen + responsesGiven + T1CF_TotalPc + T1PF_TotalPc + T1.LetKen + T1RANcT + T1RANoT, data = TableDat, numeric.stats=c("meansd","medianrange"))
summary(tab, text=TRUE, digits=2, test=F)
```

```
## 
## 
## |                  |          B (N=161)           |          NL (N=86)          |        Total (N=247)        |
## |:-----------------|:----------------------------:|:---------------------------:|:---------------------------:|
## |EMT               |                              |                             |                             |
## |-  Mean (SD)      |         11.58 (6.83)         |        21.80 (11.23)        |        15.08 (9.85)         |
## |-  Median (Range) |     10.50 (1.00, 41.50)      |     18.00 (3.50, 45.00)     |     12.50 (1.00, 45.00)     |
## |FamRisk           |                              |                             |                             |
## |-  no             |         137 (85.1%)          |         69 (80.2%)          |         206 (83.4%)         |
## |-  yes            |          24 (14.9%)          |         17 (19.8%)          |         41 (16.6%)          |
## |Cond              |                              |                             |                             |
## |-  Math           |          56 (34.8%)          |         48 (55.8%)          |         104 (42.1%)         |
## |-  Passive        |          52 (32.3%)          |          0 (0.0%)           |         52 (21.1%)          |
## |-  Read           |          53 (32.9%)          |         38 (44.2%)          |         91 (36.8%)          |
## |T1AgeF            |                              |                             |                             |
## |-  Mean (SD)      |         6.26 (0.33)          |         6.23 (0.31)         |         6.25 (0.32)         |
## |-  Median (Range) |      6.26 (5.68, 7.39)       |      6.26 (5.46, 6.83)      |      6.26 (5.46, 7.39)      |
## |HandScore         |                              |                             |                             |
## |-  Mean (SD)      |         -0.05 (1.03)         |         0.03 (1.03)         |        -0.02 (1.03)         |
## |-  Median (Range) |      0.35 (-3.20, 0.52)      |     0.35 (-3.20, 0.52)      |     0.35 (-3.20, 0.52)      |
## |Gender            |                              |                             |                             |
## |-  female         |          66 (41.0%)          |         46 (53.5%)          |         112 (45.3%)         |
## |-  male           |          95 (59.0%)          |         40 (46.5%)          |         135 (54.7%)         |
## |Lang              |                              |                             |                             |
## |-  mono           |         150 (93.2%)          |         72 (83.7%)          |         222 (89.9%)         |
## |-  multi          |          11 (6.8%)           |         14 (16.3%)          |         25 (10.1%)          |
## |SON_1st           |                              |                             |                             |
## |-  Mean (SD)      |         -0.17 (0.97)         |         0.44 (0.93)         |         0.05 (0.99)         |
## |-  Median (Range) |     -0.33 (-2.37, 3.33)      |     0.48 (-1.55, 4.96)      |     0.08 (-2.37, 4.96)      |
## |hoursPlayed       |                              |                             |                             |
## |-  Mean (SD)      |         3.90 (0.78)          |         2.94 (0.66)         |         3.48 (0.87)         |
## |-  Median (Range) |      4.07 (1.97, 5.24)       |      2.93 (1.68, 4.95)      |      3.40 (1.68, 5.24)      |
## |levelsPlayed      |                              |                             |                             |
## |-  Mean (SD)      |       355.40 (174.26)        |       284.27 (82.96)        |       324.03 (145.51)       |
## |-  Median (Range) |   309.00 (119.00, 1026.00)   |   280.00 (101.00, 607.00)   |  289.00 (101.00, 1026.00)   |
## |sessionsPlayed    |                              |                             |                             |
## |-  Mean (SD)      |         27.78 (5.21)         |        26.69 (3.23)         |        27.30 (4.47)         |
## |-  Median (Range) |     26.00 (16.00, 37.00)     |    27.00 (20.00, 34.00)     |    26.00 (16.00, 37.00)     |
## |MaxLevel          |                              |                             |                             |
## |-  Mean (SD)      |        119.65 (43.14)        |       145.79 (45.02)        |       131.18 (45.75)        |
## |-  Median (Range) |    128.00 (38.00, 265.00)    |   136.00 (62.00, 265.00)    |   135.00 (38.00, 265.00)    |
## |itemsSeen         |                              |                             |                             |
## |-  Mean (SD)      |      10518.28 (4097.98)      |      9057.73 (4180.63)      |      9874.14 (4187.51)      |
## |-  Median (Range) | 10010.00 (2827.00, 22782.00) | 7844.00 (3304.00, 20113.00) | 8778.00 (2827.00, 22782.00) |
## |responsesGiven    |                              |                             |                             |
## |-  Mean (SD)      |      3695.00 (2001.38)       |      2759.95 (951.37)       |      3282.62 (1686.15)      |
## |-  Median (Range) |  3090.00 (948.00, 10877.00)  |  2686.00 (999.00, 6984.00)  | 2943.00 (948.00, 10877.00)  |
## |T1CF_TotalPc      |                              |                             |                             |
## |-  Mean (SD)      |        38.31 (22.56)         |        61.55 (20.63)        |        46.53 (24.52)        |
## |-  Median (Range) |     37.00 (1.00, 95.00)      |     63.00 (9.00, 99.00)     |     50.00 (1.00, 99.00)     |
## |T1PF_TotalPc      |                              |                             |                             |
## |-  Mean (SD)      |        46.46 (26.89)         |        68.23 (25.11)        |        54.04 (28.22)        |
## |-  Median (Range) |     42.50 (0.00, 100.00)     |    75.00 (7.50, 100.00)     |    55.00 (0.00, 100.00)     |
## |T1.LetKen         |                              |                             |                             |
## |-  Mean (SD)      |         9.59 (6.27)          |        23.50 (5.60)         |        14.46 (8.97)         |
## |-  Median (Range) |      8.00 (0.00, 26.00)      |     25.00 (2.00, 30.00)     |     13.50 (0.00, 30.00)     |
## |T1RANcT           |                              |                             |                             |
## |-  Mean (SD)      |        71.05 (15.97)         |        58.86 (13.09)        |        66.81 (16.09)        |
## |-  Median (Range) |    69.00 (40.00, 122.00)     |    57.50 (39.00, 102.00)    |    65.00 (39.00, 122.00)    |
## |T1RANoT           |                              |                             |                             |
## |-  Mean (SD)      |        77.86 (19.83)         |        68.35 (14.00)        |        74.53 (18.55)        |
## |-  Median (Range) |    76.00 (46.00, 180.00)     |    67.00 (40.00, 126.00)    |    72.00 (40.00, 180.00)    |
```

```
tab <- tableby(Gender ~ EMT + FamRisk + Cond + T1AgeF + HandScore + Country + Lang + SON_1st + hoursPlayed + levelsPlayed + sessionsPlayed + MaxLevel + itemsSeen + responsesGiven + T1CF_TotalPc + T1PF_TotalPc + T1.LetKen + T1RANcT + T1RANoT, data = TableDat, numeric.stats=c("meansd","medianrange"))
summary(tab, text=TRUE, digits=2, test=F)
```

```
## 
## 
## |                  |       female (N=112)        |        male (N=135)         |        Total (N=247)        |
## |:-----------------|:---------------------------:|:---------------------------:|:---------------------------:|
## |EMT               |                             |                             |                             |
## |-  Mean (SD)      |        17.00 (10.60)        |        13.52 (8.94)         |        15.08 (9.85)         |
## |-  Median (Range) |     14.75 (1.50, 45.00)     |     11.50 (1.00, 45.00)     |     12.50 (1.00, 45.00)     |
## |FamRisk           |                             |                             |                             |
## |-  no             |         92 (82.1%)          |         114 (84.4%)         |         206 (83.4%)         |
## |-  yes            |         20 (17.9%)          |         21 (15.6%)          |         41 (16.6%)          |
## |Cond              |                             |                             |                             |
## |-  Math           |         44 (39.3%)          |         60 (44.4%)          |         104 (42.1%)         |
## |-  Passive        |         21 (18.8%)          |         31 (23.0%)          |         52 (21.1%)          |
## |-  Read           |         47 (42.0%)          |         44 (32.6%)          |         91 (36.8%)          |
## |T1AgeF            |                             |                             |                             |
## |-  Mean (SD)      |         6.22 (0.33)         |         6.26 (0.31)         |         6.25 (0.32)         |
## |-  Median (Range) |      6.22 (5.46, 7.39)      |      6.30 (5.55, 7.26)      |      6.26 (5.46, 7.39)      |
## |HandScore         |                             |                             |                             |
## |-  Mean (SD)      |         0.05 (0.97)         |        -0.08 (1.08)         |        -0.02 (1.03)         |
## |-  Median (Range) |     0.35 (-3.20, 0.52)      |     0.35 (-3.20, 0.52)      |     0.35 (-3.20, 0.52)      |
## |Country           |                             |                             |                             |
## |-  B              |         66 (58.9%)          |         95 (70.4%)          |         161 (65.2%)         |
## |-  NL             |         46 (41.1%)          |         40 (29.6%)          |         86 (34.8%)          |
## |Lang              |                             |                             |                             |
## |-  mono           |         99 (88.4%)          |         123 (91.1%)         |         222 (89.9%)         |
## |-  multi          |         13 (11.6%)          |          12 (8.9%)          |         25 (10.1%)          |
## |SON_1st           |                             |                             |                             |
## |-  Mean (SD)      |         0.19 (0.98)         |        -0.08 (0.99)         |         0.05 (0.99)         |
## |-  Median (Range) |     0.08 (-2.37, 4.96)      |     0.08 (-2.37, 2.52)      |     0.08 (-2.37, 4.96)      |
## |hoursPlayed       |                             |                             |                             |
## |-  Mean (SD)      |         3.50 (0.90)         |         3.46 (0.85)         |         3.48 (0.87)         |
## |-  Median (Range) |      3.40 (1.68, 5.24)      |      3.37 (1.97, 5.18)      |      3.40 (1.68, 5.24)      |
## |levelsPlayed      |                             |                             |                             |
## |-  Mean (SD)      |       302.49 (120.42)       |       342.88 (162.61)       |       324.03 (145.51)       |
## |-  Median (Range) |   275.00 (101.00, 694.00)   |  310.50 (119.00, 1026.00)   |  289.00 (101.00, 1026.00)   |
## |sessionsPlayed    |                             |                             |                             |
## |-  Mean (SD)      |        27.40 (4.48)         |        27.21 (4.48)         |        27.30 (4.47)         |
## |-  Median (Range) |    27.00 (16.00, 37.00)     |    26.00 (19.00, 37.00)     |    26.00 (16.00, 37.00)     |
## |MaxLevel          |                             |                             |                             |
## |-  Mean (SD)      |       133.13 (48.22)        |       129.47 (43.65)        |       131.18 (45.75)        |
## |-  Median (Range) |   135.00 (38.00, 265.00)    |   135.50 (41.00, 265.00)    |   135.00 (38.00, 265.00)    |
## |itemsSeen         |                             |                             |                             |
## |-  Mean (SD)      |      9722.09 (4018.76)      |     10007.18 (4344.79)      |      9874.14 (4187.51)      |
## |-  Median (Range) | 9286.00 (2827.00, 18528.00) | 8688.50 (2983.00, 22782.00) | 8778.00 (2827.00, 22782.00) |
## |responsesGiven    |                             |                             |                             |
## |-  Mean (SD)      |      3059.71 (1416.44)      |      3477.66 (1876.05)      |      3282.62 (1686.15)      |
## |-  Median (Range) |  2859.00 (999.00, 8746.00)  | 3074.00 (948.00, 10877.00)  | 2943.00 (948.00, 10877.00)  |
## |T1CF_TotalPc      |                             |                             |                             |
## |-  Mean (SD)      |        52.00 (24.11)        |        42.02 (24.03)        |        46.53 (24.52)        |
## |-  Median (Range) |     50.00 (5.00, 99.00)     |     37.00 (1.00, 95.00)     |     50.00 (1.00, 99.00)     |
## |T1PF_TotalPc      |                             |                             |                             |
## |-  Mean (SD)      |        60.83 (28.01)        |        48.41 (27.23)        |        54.04 (28.22)        |
## |-  Median (Range) |    57.50 (2.50, 100.00)     |    47.50 (0.00, 100.00)     |    55.00 (0.00, 100.00)     |
## |T1.LetKen         |                             |                             |                             |
## |-  Mean (SD)      |        16.84 (9.24)         |        12.50 (8.28)         |        14.46 (8.97)         |
## |-  Median (Range) |     18.00 (1.00, 30.00)     |     10.00 (0.00, 30.00)     |     13.50 (0.00, 30.00)     |
## |T1RANcT           |                             |                             |                             |
## |-  Mean (SD)      |        64.37 (15.01)        |        68.83 (16.72)        |        66.81 (16.09)        |
## |-  Median (Range) |    63.00 (39.00, 115.00)    |    66.00 (40.00, 122.00)    |    65.00 (39.00, 122.00)    |
## |T1RANoT           |                             |                             |                             |
## |-  Mean (SD)      |        71.28 (17.44)        |        77.21 (19.06)        |        74.53 (18.55)        |
## |-  Median (Range) |    69.00 (43.00, 127.00)    |    76.00 (40.00, 180.00)    |    72.00 (40.00, 180.00)    |
```

```
tab <- tableby(FamRisk ~ EMT + Country + Cond + T1AgeF + HandScore + Gender + Lang + SON_1st + hoursPlayed + levelsPlayed + sessionsPlayed + MaxLevel + itemsSeen + responsesGiven + T1CF_TotalPc + T1PF_TotalPc + T1.LetKen + T1RANcT + T1RANoT, data = TableDat, numeric.stats=c("meansd","medianrange"))
summary(tab, text=TRUE, digits=2, test=F)
```

```
## 
## 
## |                  |         no (N=206)          |         yes (N=41)          |        Total (N=247)        |
## |:-----------------|:---------------------------:|:---------------------------:|:---------------------------:|
## |EMT               |                             |                             |                             |
## |-  Mean (SD)      |        15.15 (9.68)         |        14.73 (10.80)        |        15.08 (9.85)         |
## |-  Median (Range) |     12.50 (1.00, 45.00)     |     12.50 (1.00, 45.00)     |     12.50 (1.00, 45.00)     |
## |Country           |                             |                             |                             |
## |-  B              |         137 (66.5%)         |         24 (58.5%)          |         161 (65.2%)         |
## |-  NL             |         69 (33.5%)          |         17 (41.5%)          |         86 (34.8%)          |
## |Cond              |                             |                             |                             |
## |-  Math           |         86 (41.7%)          |         18 (43.9%)          |         104 (42.1%)         |
## |-  Passive        |         45 (21.8%)          |          7 (17.1%)          |         52 (21.1%)          |
## |-  Read           |         75 (36.4%)          |         16 (39.0%)          |         91 (36.8%)          |
## |T1AgeF            |                             |                             |                             |
## |-  Mean (SD)      |         6.25 (0.32)         |         6.23 (0.34)         |         6.25 (0.32)         |
## |-  Median (Range) |      6.28 (5.46, 7.26)      |      6.22 (5.76, 7.39)      |      6.26 (5.46, 7.39)      |
## |HandScore         |                             |                             |                             |
## |-  Mean (SD)      |        -0.02 (1.05)         |        -0.03 (0.94)         |        -0.02 (1.03)         |
## |-  Median (Range) |     0.35 (-3.20, 0.52)      |     0.35 (-3.20, 0.52)      |     0.35 (-3.20, 0.52)      |
## |Gender            |                             |                             |                             |
## |-  female         |         92 (44.7%)          |         20 (48.8%)          |         112 (45.3%)         |
## |-  male           |         114 (55.3%)         |         21 (51.2%)          |         135 (54.7%)         |
## |Lang              |                             |                             |                             |
## |-  mono           |         186 (90.3%)         |         36 (87.8%)          |         222 (89.9%)         |
## |-  multi          |          20 (9.7%)          |          5 (12.2%)          |         25 (10.1%)          |
## |SON_1st           |                             |                             |                             |
## |-  Mean (SD)      |         0.03 (0.97)         |         0.12 (1.11)         |         0.05 (0.99)         |
## |-  Median (Range) |     0.08 (-2.37, 4.96)      |     0.08 (-2.37, 3.33)      |     0.08 (-2.37, 4.96)      |
## |hoursPlayed       |                             |                             |                             |
## |-  Mean (SD)      |         3.49 (0.89)         |         3.42 (0.77)         |         3.48 (0.87)         |
## |-  Median (Range) |      3.40 (1.68, 5.24)      |      3.38 (1.97, 4.94)      |      3.40 (1.68, 5.24)      |
## |levelsPlayed      |                             |                             |                             |
## |-  Mean (SD)      |       324.78 (146.11)       |       320.50 (144.78)       |       324.03 (145.51)       |
## |-  Median (Range) |  287.00 (101.00, 1026.00)   |   314.50 (119.00, 722.00)   |  289.00 (101.00, 1026.00)   |
## |sessionsPlayed    |                             |                             |                             |
## |-  Mean (SD)      |        27.48 (4.64)         |        26.44 (3.52)         |        27.30 (4.47)         |
## |-  Median (Range) |    27.00 (16.00, 37.00)     |    25.00 (22.00, 35.00)     |    26.00 (16.00, 37.00)     |
## |MaxLevel          |                             |                             |                             |
## |-  Mean (SD)      |       132.84 (44.52)        |       123.32 (51.18)        |       131.18 (45.75)        |
## |-  Median (Range) |   135.00 (38.00, 265.00)    |   135.50 (41.00, 265.00)    |   135.00 (38.00, 265.00)    |
## |itemsSeen         |                             |                             |                             |
## |-  Mean (SD)      |      9868.60 (4065.96)      |      9900.35 (4788.53)      |      9874.14 (4187.51)      |
## |-  Median (Range) | 8842.00 (2827.00, 21452.00) | 8409.00 (2983.00, 22782.00) | 8778.00 (2827.00, 22782.00) |
## |responsesGiven    |                             |                             |                             |
## |-  Mean (SD)      |      3263.51 (1595.57)      |      3373.12 (2088.11)      |      3282.62 (1686.15)      |
## |-  Median (Range) |  2969.00 (999.00, 9826.00)  | 2853.00 (948.00, 10877.00)  | 2943.00 (948.00, 10877.00)  |
## |T1CF_TotalPc      |                             |                             |                             |
## |-  Mean (SD)      |        47.31 (24.28)        |        42.71 (25.68)        |        46.53 (24.52)        |
## |-  Median (Range) |     50.00 (1.00, 99.00)     |     37.00 (1.00, 91.00)     |     50.00 (1.00, 99.00)     |
## |T1PF_TotalPc      |                             |                             |                             |
## |-  Mean (SD)      |        54.61 (27.93)        |        51.16 (29.79)        |        54.04 (28.22)        |
## |-  Median (Range) |    55.00 (0.00, 100.00)     |    47.50 (0.00, 100.00)     |    55.00 (0.00, 100.00)     |
## |T1.LetKen         |                             |                             |                             |
## |-  Mean (SD)      |        14.32 (8.95)         |        15.15 (9.19)         |        14.46 (8.97)         |
## |-  Median (Range) |     13.00 (0.00, 30.00)     |     14.00 (1.00, 30.00)     |     13.50 (0.00, 30.00)     |
## |T1RANcT           |                             |                             |                             |
## |-  Mean (SD)      |        66.00 (16.50)        |        70.83 (13.31)        |        66.81 (16.09)        |
## |-  Median (Range) |    63.00 (39.00, 122.00)    |    70.00 (43.00, 106.00)    |    65.00 (39.00, 122.00)    |
## |T1RANoT           |                             |                             |                             |
## |-  Mean (SD)      |        73.26 (18.08)        |        80.88 (19.75)        |        74.53 (18.55)        |
## |-  Median (Range) |    71.00 (40.00, 180.00)    |    75.00 (56.00, 127.00)    |    72.00 (40.00, 180.00)    |
```

```
TableDat$CountrCond <- interaction(TableDat$Country, TableDat$Cond)
tab <- tableby(CountrCond ~ EMT + FamRisk + T1AgeF + HandScore + Gender + Lang + SON_1st + hoursPlayed + levelsPlayed + sessionsPlayed + MaxLevel + itemsSeen + responsesGiven + T1CF_TotalPc + T1PF_TotalPc + T1.LetKen + T1RANcT + T1RANoT, data = TableDat, numeric.stats=c("meansd","medianrange"))
summary(tab, text=TRUE, digits=2, test=F)
```

```
## 
## 
## |                  |        B.Math (N=56)        |       NL.Math (N=48)        |   B.Passive (N=52)    |        B.Read (N=53)         |        NL.Read (N=38)        |        Total (N=247)        |
## |:-----------------|:---------------------------:|:---------------------------:|:---------------------:|:----------------------------:|:----------------------------:|:---------------------------:|
## |EMT               |                             |                             |                       |                              |                              |                             |
## |-  Mean (SD)      |        12.02 (7.79)         |        23.83 (11.67)        |     10.05 (4.59)      |         12.62 (7.43)         |        19.34 (10.29)         |        15.08 (9.85)         |
## |-  Median (Range) |     10.00 (1.00, 41.50)     |     19.50 (3.50, 45.00)     |  10.50 (2.50, 22.00)  |     12.50 (1.00, 32.50)      |     16.50 (8.00, 45.00)      |     12.50 (1.00, 45.00)     |
## |FamRisk           |                             |                             |                       |                              |                              |                             |
## |-  no             |         49 (87.5%)          |         37 (77.1%)          |      45 (86.5%)       |          43 (81.1%)          |          32 (84.2%)          |         206 (83.4%)         |
## |-  yes            |          7 (12.5%)          |         11 (22.9%)          |       7 (13.5%)       |          10 (18.9%)          |          6 (15.8%)           |         41 (16.6%)          |
## |T1AgeF            |                             |                             |                       |                              |                              |                             |
## |-  Mean (SD)      |         6.31 (0.31)         |         6.25 (0.32)         |      6.20 (0.30)      |         6.26 (0.37)          |         6.19 (0.29)          |         6.25 (0.32)         |
## |-  Median (Range) |      6.31 (5.70, 7.07)      |      6.32 (5.55, 6.83)      |   6.17 (5.68, 6.80)   |      6.25 (5.71, 7.39)       |      6.20 (5.46, 6.69)       |      6.26 (5.46, 7.39)      |
## |HandScore         |                             |                             |                       |                              |                              |                             |
## |-  Mean (SD)      |         0.07 (1.00)         |         0.12 (0.87)         |     -0.27 (1.27)      |         0.05 (0.76)          |         -0.10 (1.20)         |        -0.02 (1.03)         |
## |-  Median (Range) |     0.35 (-3.20, 0.52)      |     0.35 (-3.20, 0.52)      |  0.35 (-3.20, 0.52)   |      0.35 (-3.20, 0.52)      |      0.44 (-3.20, 0.52)      |     0.35 (-3.20, 0.52)      |
## |Gender            |                             |                             |                       |                              |                              |                             |
## |-  female         |         21 (37.5%)          |         23 (47.9%)          |      21 (40.4%)       |          24 (45.3%)          |          23 (60.5%)          |         112 (45.3%)         |
## |-  male           |         35 (62.5%)          |         25 (52.1%)          |      31 (59.6%)       |          29 (54.7%)          |          15 (39.5%)          |         135 (54.7%)         |
## |Lang              |                             |                             |                       |                              |                              |                             |
## |-  mono           |         53 (94.6%)          |         36 (75.0%)          |      46 (88.5%)       |          51 (96.2%)          |          36 (94.7%)          |         222 (89.9%)         |
## |-  multi          |          3 (5.4%)           |         12 (25.0%)          |       6 (11.5%)       |           2 (3.8%)           |           2 (5.3%)           |         25 (10.1%)          |
## |SON_1st           |                             |                             |                       |                              |                              |                             |
## |-  Mean (SD)      |        -0.15 (1.02)         |         0.41 (0.81)         |     -0.29 (0.99)      |         -0.06 (0.88)         |         0.48 (1.07)          |         0.05 (0.99)         |
## |-  Median (Range) |     0.08 (-2.37, 3.33)      |     0.48 (-1.55, 2.52)      |  -0.54 (-2.37, 1.70)  |      0.08 (-2.37, 1.30)      |      0.48 (-1.55, 4.96)      |     0.08 (-2.37, 4.96)      |
## |hoursPlayed       |                             |                             |                       |                              |                              |                             |
## |-  Mean (SD)      |         3.96 (0.76)         |         2.70 (0.43)         |          NA           |         3.83 (0.80)          |         3.26 (0.77)          |         3.48 (0.87)         |
## |-  Median (Range) |      4.17 (2.12, 5.24)      |      2.72 (1.96, 3.80)      |          NA           |      3.98 (1.97, 5.23)       |      3.24 (1.68, 4.95)       |      3.40 (1.68, 5.24)      |
## |levelsPlayed      |                             |                             |                       |                              |                              |                             |
## |-  Mean (SD)      |       473.43 (165.94)       |       318.25 (77.64)        |          NA           |        230.70 (55.57)        |        241.34 (69.02)        |       324.03 (145.51)       |
## |-  Median (Range) |  460.00 (132.00, 1026.00)   |   317.50 (191.00, 607.00)   |          NA           |   228.00 (119.00, 369.00)    |   237.50 (101.00, 377.00)    |  289.00 (101.00, 1026.00)   |
## |sessionsPlayed    |                             |                             |                       |                              |                              |                             |
## |-  Mean (SD)      |        27.82 (4.82)         |        25.48 (2.02)         |          NA           |         27.74 (5.64)         |         28.21 (3.81)         |        27.30 (4.47)         |
## |-  Median (Range) |    27.00 (18.00, 35.00)     |    25.00 (20.00, 30.00)     |          NA           |     26.00 (16.00, 37.00)     |     29.00 (20.00, 34.00)     |    26.00 (16.00, 37.00)     |
## |MaxLevel          |                             |                             |                       |                              |                              |                             |
## |-  Mean (SD)      |       136.84 (31.04)        |       135.94 (23.95)        |          NA           |        101.49 (46.82)        |        158.24 (60.35)        |       131.18 (45.75)        |
## |-  Median (Range) |   137.50 (39.00, 178.00)    |   135.50 (62.00, 178.00)    |          NA           |    87.00 (38.00, 265.00)     |    164.50 (71.00, 265.00)    |   135.00 (38.00, 265.00)    |
## |itemsSeen         |                             |                             |                       |                              |                              |                             |
## |-  Mean (SD)      |     10747.04 (4563.03)      |      6652.12 (2116.28)      |          NA           |      10276.57 (3569.36)      |      12096.39 (4179.95)      |      9874.14 (4187.51)      |
## |-  Median (Range) | 9805.50 (2827.00, 22782.00) | 6722.50 (3304.00, 15832.00) |          NA           | 10145.00 (2983.00, 18124.00) | 11825.50 (4191.00, 20113.00) | 8778.00 (2827.00, 22782.00) |
## |responsesGiven    |                             |                             |                       |                              |                              |                             |
## |-  Mean (SD)      |      4729.16 (2259.21)      |      2753.85 (1001.08)      |          NA           |       2602.30 (742.90)       |       2767.66 (897.87)       |      3282.62 (1686.15)      |
## |-  Median (Range) | 4261.00 (1019.00, 10877.00) | 2668.50 (1197.00, 6984.00)  |          NA           |  2603.00 (948.00, 4072.00)   |  2767.00 (999.00, 4522.00)   | 2943.00 (948.00, 10877.00)  |
## |T1CF_TotalPc      |                             |                             |                       |                              |                              |                             |
## |-  Mean (SD)      |        38.22 (23.68)        |        59.85 (19.35)        |     41.14 (23.91)     |        35.74 (20.05)         |        63.68 (22.22)         |        46.53 (24.52)        |
## |-  Median (Range) |     37.00 (1.00, 91.00)     |     63.00 (9.00, 91.00)     |  37.00 (1.00, 95.00)  |     37.00 (5.00, 84.00)      |     56.50 (25.00, 99.00)     |     50.00 (1.00, 99.00)     |
## |T1PF_TotalPc      |                             |                             |                       |                              |                              |                             |
## |-  Mean (SD)      |        45.67 (29.28)        |        64.84 (26.55)        |     49.52 (25.94)     |        44.29 (25.37)         |        72.50 (22.80)         |        54.04 (28.22)        |
## |-  Median (Range) |    45.00 (0.00, 100.00)     |    66.25 (7.50, 100.00)     |  47.50 (0.00, 97.50)  |     42.50 (0.00, 97.50)      |    75.00 (22.50, 100.00)     |    55.00 (0.00, 100.00)     |
## |T1.LetKen         |                             |                             |                       |                              |                              |                             |
## |-  Mean (SD)      |         9.36 (6.42)         |        24.50 (4.74)         |     11.27 (5.30)      |         8.23 (6.70)          |         22.24 (6.36)         |        14.46 (8.97)         |
## |-  Median (Range) |     8.00 (0.00, 26.00)      |    26.00 (13.00, 30.00)     |  10.00 (0.00, 25.00)  |      6.00 (0.00, 25.00)      |     24.00 (2.00, 30.00)      |     13.50 (0.00, 30.00)     |
## |T1RANcT           |                             |                             |                       |                              |                              |                             |
## |-  Mean (SD)      |        67.50 (15.37)        |        58.44 (11.80)        |     70.88 (15.92)     |        74.96 (16.02)         |        59.39 (14.71)         |        66.81 (16.09)        |
## |-  Median (Range) |    67.00 (40.00, 107.00)    |    57.50 (41.00, 96.00)     | 67.50 (48.00, 115.00) |    70.00 (49.00, 122.00)     |    57.50 (39.00, 102.00)     |    65.00 (39.00, 122.00)    |
## |T1RANoT           |                             |                             |                       |                              |                              |                             |
## |-  Mean (SD)      |        76.25 (17.33)        |        70.46 (15.11)        |     75.56 (17.49)     |        81.77 (23.82)         |        65.68 (12.13)         |        74.53 (18.55)        |
## |-  Median (Range) |    72.00 (50.00, 120.00)    |    68.00 (40.00, 126.00)    | 76.50 (46.00, 127.00) |    76.00 (49.00, 180.00)     |     65.50 (43.00, 90.00)     |    72.00 (40.00, 180.00)    |
```

```
TableDat$CountrFamRisk <- interaction(TableDat$Country, TableDat$FamRisk)
tab <- tableby(CountrFamRisk ~ EMT + Cond + T1AgeF + HandScore + Gender + Lang + SON_1st + hoursPlayed + levelsPlayed + sessionsPlayed + MaxLevel + itemsSeen + responsesGiven + T1CF_TotalPc + T1PF_TotalPc + T1.LetKen + T1RANcT + T1RANoT, data = TableDat, numeric.stats=c("meansd","medianrange"))
summary(tab, text=TRUE, digits=2, test=F)
```

```
## 
## 
## |                  |         B.no (N=137)         |        NL.no (N=69)         |         B.yes (N=24)         |        NL.yes (N=17)        |        Total (N=247)        |
## |:-----------------|:----------------------------:|:---------------------------:|:----------------------------:|:---------------------------:|:---------------------------:|
## |EMT               |                              |                             |                              |                             |                             |
## |-  Mean (SD)      |         11.83 (6.98)         |        21.96 (10.87)        |         10.17 (5.88)         |        21.18 (12.90)        |        15.08 (9.85)         |
## |-  Median (Range) |     10.50 (1.00, 41.50)      |     18.00 (8.00, 45.00)     |      9.50 (1.00, 21.00)      |     17.50 (3.50, 45.00)     |     12.50 (1.00, 45.00)     |
## |Cond              |                              |                             |                              |                             |                             |
## |-  Math           |          49 (35.8%)          |         37 (53.6%)          |          7 (29.2%)           |         11 (64.7%)          |         104 (42.1%)         |
## |-  Passive        |          45 (32.8%)          |          0 (0.0%)           |          7 (29.2%)           |          0 (0.0%)           |         52 (21.1%)          |
## |-  Read           |          43 (31.4%)          |         32 (46.4%)          |          10 (41.7%)          |          6 (35.3%)          |         91 (36.8%)          |
## |T1AgeF            |                              |                             |                              |                             |                             |
## |-  Mean (SD)      |         6.26 (0.31)          |         6.23 (0.33)         |         6.25 (0.40)          |         6.20 (0.23)         |         6.25 (0.32)         |
## |-  Median (Range) |      6.29 (5.68, 7.26)       |      6.27 (5.46, 6.83)      |      6.22 (5.76, 7.39)       |      6.22 (5.82, 6.56)      |      6.26 (5.46, 7.39)      |
## |HandScore         |                              |                             |                              |                             |                             |
## |-  Mean (SD)      |         -0.04 (1.04)         |         0.01 (1.07)         |         -0.11 (1.00)         |         0.08 (0.88)         |        -0.02 (1.03)         |
## |-  Median (Range) |      0.35 (-3.20, 0.52)      |     0.52 (-3.20, 0.52)      |      0.35 (-3.03, 0.52)      |     0.35 (-3.20, 0.52)      |     0.35 (-3.20, 0.52)      |
## |Gender            |                              |                             |                              |                             |                             |
## |-  female         |          55 (40.1%)          |         37 (53.6%)          |          11 (45.8%)          |          9 (52.9%)          |         112 (45.3%)         |
## |-  male           |          82 (59.9%)          |         32 (46.4%)          |          13 (54.2%)          |          8 (47.1%)          |         135 (54.7%)         |
## |Lang              |                              |                             |                              |                             |                             |
## |-  mono           |         128 (93.4%)          |         58 (84.1%)          |          22 (91.7%)          |         14 (82.4%)          |         222 (89.9%)         |
## |-  multi          |           9 (6.6%)           |         11 (15.9%)          |           2 (8.3%)           |          3 (17.6%)          |         25 (10.1%)          |
## |SON_1st           |                              |                             |                              |                             |                             |
## |-  Mean (SD)      |         -0.16 (0.92)         |         0.41 (0.97)         |         -0.20 (1.23)         |         0.58 (0.71)         |         0.05 (0.99)         |
## |-  Median (Range) |     -0.33 (-2.37, 1.70)      |     0.48 (-1.55, 4.96)      |      0.08 (-2.37, 3.33)      |     0.48 (-0.33, 2.52)      |     0.08 (-2.37, 4.96)      |
## |hoursPlayed       |                              |                             |                              |                             |                             |
## |-  Mean (SD)      |         3.91 (0.80)          |         2.92 (0.67)         |         3.81 (0.67)          |         3.02 (0.66)         |         3.48 (0.87)         |
## |-  Median (Range) |      4.11 (2.12, 5.24)       |      2.94 (1.68, 4.95)      |      3.89 (1.97, 4.68)       |      2.82 (2.23, 4.94)      |      3.40 (1.68, 5.24)      |
## |levelsPlayed      |                              |                             |                              |                             |                             |
## |-  Mean (SD)      |       356.64 (172.20)        |       282.29 (85.66)        |       348.71 (190.40)        |       292.29 (72.74)        |       324.03 (145.51)       |
## |-  Median (Range) |   314.00 (132.00, 1026.00)   |   275.00 (101.00, 607.00)   |   282.00 (119.00, 722.00)    |   316.00 (152.00, 414.00)   |  289.00 (101.00, 1026.00)   |
## |sessionsPlayed    |                              |                             |                              |                             |                             |
## |-  Mean (SD)      |         27.90 (5.38)         |        26.91 (3.36)         |         27.12 (4.26)         |        25.76 (2.54)         |        27.30 (4.47)         |
## |-  Median (Range) |     26.00 (16.00, 37.00)     |    27.00 (20.00, 34.00)     |     26.00 (22.00, 35.00)     |    25.00 (23.00, 33.00)     |    26.00 (16.00, 37.00)     |
## |MaxLevel          |                              |                             |                              |                             |                             |
## |-  Mean (SD)      |        122.01 (40.98)        |       147.28 (45.24)        |        106.88 (52.98)        |       139.76 (44.97)        |       131.18 (45.75)        |
## |-  Median (Range) |    130.50 (38.00, 241.00)    |   136.00 (71.00, 265.00)    |    94.00 (41.00, 265.00)     |   136.00 (62.00, 241.00)    |   135.00 (38.00, 265.00)    |
## |itemsSeen         |                              |                             |                              |                             |                             |
## |-  Mean (SD)      |      10390.43 (3860.55)      |      9172.83 (4253.74)      |      11210.12 (5290.32)      |      8590.59 (3957.31)      |      9874.14 (4187.51)      |
## |-  Median (Range) | 10018.50 (2827.00, 21452.00) | 8025.00 (3304.00, 20113.00) | 10010.00 (2983.00, 22782.00) | 7664.00 (5069.00, 18594.00) | 8778.00 (2827.00, 22782.00) |
## |responsesGiven    |                              |                             |                              |                             |                             |
## |-  Mean (SD)      |      3638.86 (1840.26)       |      2763.04 (1006.70)      |      3998.82 (2767.65)       |      2747.41 (707.86)       |      3282.62 (1686.15)      |
## |-  Median (Range) |  3106.50 (1019.00, 9826.00)  |  2842.00 (999.00, 6984.00)  |  3090.00 (948.00, 10877.00)  | 2485.00 (1882.00, 4212.00)  | 2943.00 (948.00, 10877.00)  |
## |T1CF_TotalPc      |                              |                             |                              |                             |                             |
## |-  Mean (SD)      |        39.02 (22.14)         |        63.30 (19.91)        |        34.42 (24.90)         |        54.41 (22.54)        |        46.53 (24.52)        |
## |-  Median (Range) |     37.00 (1.00, 95.00)      |    63.00 (16.00, 99.00)     |     31.00 (1.00, 84.00)      |     50.00 (9.00, 91.00)     |     50.00 (1.00, 99.00)     |
## |T1PF_TotalPc      |                              |                             |                              |                             |                             |
## |-  Mean (SD)      |        47.41 (26.62)         |        68.91 (24.97)        |        41.04 (28.38)         |        65.44 (26.28)        |        54.04 (28.22)        |
## |-  Median (Range) |     47.50 (0.00, 100.00)     |    75.00 (7.50, 100.00)     |     32.50 (0.00, 97.50)      |    75.00 (20.00, 100.00)    |    55.00 (0.00, 100.00)     |
## |T1.LetKen         |                              |                             |                              |                             |                             |
## |-  Mean (SD)      |         9.72 (6.40)          |        23.38 (5.78)         |         8.88 (5.57)          |        24.00 (4.92)         |        14.46 (8.97)         |
## |-  Median (Range) |      8.00 (0.00, 26.00)      |     25.00 (2.00, 30.00)     |      7.50 (1.00, 24.00)      |    25.00 (15.00, 30.00)     |     13.50 (0.00, 30.00)     |
## |T1RANcT           |                              |                             |                              |                             |                             |
## |-  Mean (SD)      |        70.80 (16.33)         |        56.48 (12.19)        |        72.46 (13.92)         |        68.53 (12.45)        |        66.81 (16.09)        |
## |-  Median (Range) |    68.00 (40.00, 122.00)     |    56.00 (39.00, 102.00)    |    72.00 (43.00, 106.00)     |    68.00 (45.00, 96.00)     |    65.00 (39.00, 122.00)    |
## |T1RANoT           |                              |                             |                              |                             |                             |
## |-  Mean (SD)      |        76.78 (19.42)         |        66.33 (12.59)        |        83.96 (21.46)         |        76.53 (16.70)        |        74.53 (18.55)        |
## |-  Median (Range) |    76.00 (46.00, 180.00)     |    66.00 (40.00, 94.00)     |    76.50 (58.00, 127.00)     |    72.00 (56.00, 126.00)    |    72.00 (40.00, 180.00)    |
```

---

#### 1.4.1.2 Normality at T1

None of the variables were normally distributed at T1.

```
datComb <- droplevels(subset(BehavDat, Exclude=="no"))
shapiro.test(datComb$T1AgeF)
```

```
## 
##  Shapiro-Wilk normality test
## 
## data:  datComb$T1AgeF
## W = 0.97958, p-value = 0.001267
```

```
shapiro.test(datComb$T1.LetKen)
```

```
## 
##  Shapiro-Wilk normality test
## 
## data:  datComb$T1.LetKen
## W = 0.93044, p-value = 2.314e-09
```

```
shapiro.test(datComb$T1CF_TotalPc)
```

```
## 
##  Shapiro-Wilk normality test
## 
## data:  datComb$T1CF_TotalPc
## W = 0.95943, p-value = 2.34e-06
```

```
shapiro.test(datComb$T1PF_TotalPc)
```

```
## 
##  Shapiro-Wilk normality test
## 
## data:  datComb$T1PF_TotalPc
## W = 0.95598, p-value = 7.716e-07
```

```
shapiro.test(datComb$T1RANoT)
```

```
## 
##  Shapiro-Wilk normality test
## 
## data:  datComb$T1RANoT
## W = 0.92143, p-value = 4.04e-10
```

```
shapiro.test(datComb$T1RANcT)
```

```
## 
##  Shapiro-Wilk normality test
## 
## data:  datComb$T1RANcT
## W = 0.95561, p-value = 6.996e-07
```

```
shapiro.test(datComb$SON_1st)
```

```
## 
##  Shapiro-Wilk normality test
## 
## data:  datComb$SON_1st
## W = 0.95947, p-value = 1.978e-06
```

---

#### 1.4.1.3 Differences by country and condition

Chi-squared/Fisher’s exact test to compare counts of Gender, Multilingualism, Handedness and familial risk of dyslexia in the combined sample. No significant differences were observed, although there is a trend towards more multilinguals in the math condition.

##### 1.4.1.3.1 Chi-square test Gender x Condition

```
chisq.test(matrix(c(21,31,44,60,47,44),ncol=3,dimnames=list(c("female","male"), c("Passive","Math","Read"))))
```

```
## 
##  Pearson's Chi-squared test
## 
## data:  matrix(c(21, 31, 44, 60, 47, 44), ncol = 3, dimnames = list(c("female",     "male"), c("Passive", "Math", "Read")))
## X-squared = 2.3623, df = 2, p-value = 0.3069
```

##### 1.4.1.3.2 Fisher’s exact test Multilingualism x Condition

```
fisher.test(matrix(c(6,46,15,89,4,87),ncol=3,dimnames=list(c("multilingual","monolingual"), c("Passive","Math","Read"))))
```

```
## 
##  Fisher's Exact Test for Count Data
## 
## data:  matrix(c(6, 46, 15, 89, 4, 87), ncol = 3, dimnames = list(c("multilingual", "monolingual"), c("Passive", "Math", "Read")))
## p-value = 0.05332
## alternative hypothesis: two.sided
```

##### 1.4.1.3.3 Chi-square test Handedness x Condition

```
chisq.test(matrix(c(9,43,8,96,10,81),ncol=3,dimnames=list(c("lefthander","righthander"), c("Passive","Math","Read"))))
```

```
## 
##  Pearson's Chi-squared test
## 
## data:  matrix(c(9, 43, 8, 96, 10, 81), ncol = 3, dimnames = list(c("lefthander",     "righthander"), c("Passive", "Math", "Read")))
## X-squared = 3.2924, df = 2, p-value = 0.1928
```

##### 1.4.1.3.4 Chi-square test Familial Risk x Condition

```
chisq.test(matrix(c(7,45,18,86,16,75), ncol=3, dimnames=list(c("familial risk", "no familial risk"), c("Passive","Math","Read"))))
```

```
## 
##  Pearson's Chi-squared test
## 
## data:  matrix(c(7, 45, 18, 86, 16, 75), ncol = 3, dimnames = list(c("familial risk",     "no familial risk"), c("Passive", "Math", "Read")))
## X-squared = 0.47105, df = 2, p-value = 0.7902
```

---

##### 1.4.1.3.5 ANOVA for variables on interval scales

ANOVAs testing for a Condition x Country interaction for variables Age, LK, CELF PA, PROEF PA, RAN objects, RAN colours, and abstract reasoning. We found main effect of country for LK, CELF PA, PROEF PA, RAN objects, and RAN colours. In addition, there were main effects of condition for LK and RAN colours. For LK all groups differed from knowing most letters (math) via passive to least letters (read). Further, the math group was faster in RAN colours than both other groups. We further observed a Condition x Country interaction for RAN objects in that the Math group did not differ between the two countries, but the Dutch reading group outperformed their Belgian reading group peers.

```
boxplot(T1AgeF~Cond+Country,data=datComb, main="Age at T1", ylab="Age in years")
```

```
summary(aov(T1AgeF~Cond*Country,data=datComb))
```

```
##               Df Sum Sq Mean Sq F value Pr(>F)
## Cond           2  0.278 0.13892   1.362  0.258
## Country        1  0.174 0.17399   1.706  0.193
## Cond:Country   1  0.001 0.00134   0.013  0.909
## Residuals    242 24.683 0.10199
```

```
boxplot(T1.LetKen~Cond+Country,data=datComb, main="LK at T1", ylab="Letter knowledge")
```

```
summary(aov(T1.LetKen~Cond*Country,data=datComb))
```

```
##               Df Sum Sq Mean Sq F value   Pr(>F)    
## Cond           2    901     450  12.686 5.78e-06 ***
## Country        1  10256   10256 288.837  < 2e-16 ***
## Cond:Country   1     15      15   0.431    0.512    
## Residuals    241   8557      36                     
## ---
## Signif. codes:  0 '***' 0.001 '**' 0.01 '*' 0.05 '.' 0.1 ' ' 1
## 1 observation deleted due to missingness
```

```
TukeyHSD(aov(T1.LetKen~Cond*Country,data=datComb))
```

```
##   Tukey multiple comparisons of means
##     95% family-wise confidence level
## 
## Fit: aov(formula = T1.LetKen ~ Cond * Country, data = datComb)
## 
## $Cond
##                   diff        lwr       upr     p adj
## Passive-Math -5.071644 -7.4738881 -2.669400 0.0000036
## Read-Math    -2.269231 -4.2863565 -0.252105 0.0230612
## Read-Passive  2.802413  0.3443593  5.260467 0.0208848
## 
## $Country
##          diff      lwr      upr p adj
## NL-B 12.54066 10.97121 14.11012     0
## 
## $`Cond:Country`
##                           diff       lwr       upr     p adj
## Passive:B-Math:B      1.917367 -1.396032  5.230765 0.5580253
## Read:B-Math:B        -1.130728 -4.411244  2.149788 0.9207562
## Math:NL-Math:B       15.142857 11.775704 18.510011 0.0000000
## Passive:NL-Math:B           NA        NA        NA        NA
## Read:NL-Math:B       12.879699  9.281883 16.477516 0.0000000
## Read:B-Passive:B     -3.048095 -6.405892  0.309703 0.0992899
## Math:NL-Passive:B    13.225490  9.782999 16.667981 0.0000000
## Passive:NL-Passive:B        NA        NA        NA        NA
## Read:NL-Passive:B    10.962332  7.293913 14.630752 0.0000000
## Math:NL-Read:B       16.273585 12.862732 19.684438 0.0000000
## Passive:NL-Read:B           NA        NA        NA        NA
## Read:NL-Read:B       14.010427 10.371680 17.649174 0.0000000
## Passive:NL-Math:NL          NA        NA        NA        NA
## Read:NL-Math:NL      -2.263158 -5.980202  1.453886 0.5007696
## Read:NL-Passive:NL          NA        NA        NA        NA
```

```
boxplot(T1CF_TotalPc~Cond+Country,data=datComb, main="CELF PA at T1", ylab="CELF PA (percentile)")
```

```
summary(aov(T1CF_TotalPc~Cond*Country,data=datComb))
```

```
##               Df Sum Sq Mean Sq F value   Pr(>F)    
## Cond           2   1880     940   1.954    0.144    
## Country        1  28707   28707  59.676 3.11e-13 ***
## Cond:Country   1    472     472   0.981    0.323    
## Residuals    238 114488     481                     
## ---
## Signif. codes:  0 '***' 0.001 '**' 0.01 '*' 0.05 '.' 0.1 ' ' 1
## 4 observations deleted due to missingness
```

```
boxplot(T1PF_TotalPc~Cond+Country,data=datComb, main="PREOF PA at T1", ylab="PROEF PA (percentile)")
```

```
summary(aov(T1PF_TotalPc~Cond*Country,data=datComb))
```

```
##               Df Sum Sq Mean Sq F value   Pr(>F)    
## Cond           2   1462     731   1.058    0.349    
## Country        1  26139   26139  37.812 3.19e-09 ***
## Cond:Country   1    973     973   1.407    0.237    
## Residuals    242 167291     691                     
## ---
## Signif. codes:  0 '***' 0.001 '**' 0.01 '*' 0.05 '.' 0.1 ' ' 1
```

```
boxplot(T1RANoT~Cond+Country,data=datComb, main="RAN Objects at T1", ylab="Time in seconds")
```

```
summary(aov(T1RANoT~Cond*Country,data=datComb))
```

```
##               Df Sum Sq Mean Sq F value   Pr(>F)    
## Cond           2    178      89   0.277    0.758    
## Country        1   5332    5332  16.582 6.32e-05 ***
## Cond:Country   1   1258    1258   3.913    0.049 *  
## Residuals    241  77495     322                     
## ---
## Signif. codes:  0 '***' 0.001 '**' 0.01 '*' 0.05 '.' 0.1 ' ' 1
## 1 observation deleted due to missingness
```

```
TukeyHSD(aov(T1RANoT~Cond*Country,data=datComb))
```

```
##   Tukey multiple comparisons of means
##     95% family-wise confidence level
## 
## Fit: aov(formula = T1RANoT ~ Cond * Country, data = datComb)
## 
## $Cond
##                    diff       lwr      upr     p adj
## Passive-Math  2.0042942 -5.189691 9.198280 0.7885541
## Read-Math     1.5015470 -4.582402 7.585496 0.8298643
## Read-Passive -0.5027473 -7.854140 6.848646 0.9857635
## 
## $Country
##           diff       lwr       upr    p adj
## NL-B -9.022104 -13.74513 -4.299079 0.000211
## 
## $`Cond:Country`
##                             diff        lwr        upr     p adj
## Passive:B-Math:B      -0.6968531 -10.661014  9.2673075 0.9999545
## Read:B-Math:B          5.5190395  -4.396685 15.4347639 0.6000329
## Math:NL-Math:B        -5.7962121 -15.971542  4.3791178 0.5752397
## Passive:NL-Math:B             NA         NA         NA        NA
## Read:NL-Math:B       -10.5703349 -21.437098  0.2964277 0.0617692
## Read:B-Passive:B       6.2158926  -3.839219 16.2710041 0.4832867
## Math:NL-Passive:B     -5.0993590 -15.410567  5.2118493 0.7144224
## Passive:NL-Passive:B          NA         NA         NA        NA
## Read:NL-Passive:B     -9.8734818 -20.867581  1.1206170 0.1062016
## Math:NL-Read:B       -11.3152516 -21.579661 -1.0508418 0.0212809
## Passive:NL-Read:B             NA         NA         NA        NA
## Read:NL-Read:B       -16.0893744 -27.039594 -5.1391552 0.0004894
## Passive:NL-Math:NL            NA         NA         NA        NA
## Read:NL-Math:NL       -4.7741228 -15.959964  6.4117186 0.8237344
## Read:NL-Passive:NL            NA         NA         NA        NA
```

```
boxplot(T1RANcT~Cond+Country,data=datComb, main="RAN Colours at T1", ylab="Time in seconds")
```

```
summary(aov(T1RANcT~Cond*Country,data=datComb))
```

```
##               Df Sum Sq Mean Sq F value   Pr(>F)    
## Cond           2   2380    1190   5.352  0.00532 ** 
## Country        1   6982    6982  31.397 5.71e-08 ***
## Cond:Country   1    505     505   2.269  0.13331    
## Residuals    242  53814     222                     
## ---
## Signif. codes:  0 '***' 0.001 '**' 0.01 '*' 0.05 '.' 0.1 ' ' 1
```

```
TukeyHSD(aov(T1RANcT~Cond*Country,data=datComb))
```

```
##   Tukey multiple comparisons of means
##     95% family-wise confidence level
## 
## Fit: aov(formula = T1RANcT ~ Cond * Country, data = datComb)
## 
## $Cond
##                   diff         lwr       upr     p adj
## Passive-Math  7.567308  1.59461955 13.539996 0.0086730
## Read-Math     5.144231  0.09638797 10.192074 0.0446632
## Read-Passive -2.423077 -8.53631814  3.690164 0.6188702
## 
## $Country
##           diff       lwr       upr p adj
## NL-B -10.32471 -14.24802 -6.401408 5e-07
## 
## $`Cond:Country`
##                             diff         lwr        upr     p adj
## Passive:B-Math:B       3.3846154  -4.8652450 11.6344758 0.8467470
## Read:B-Math:B          7.4622642  -0.7471413 15.6716696 0.0984482
## Math:NL-Math:B        -9.0625000 -17.4887132 -0.6362868 0.0268696
## Passive:NL-Math:B             NA          NA         NA        NA
## Read:NL-Math:B        -8.1052632 -17.1087041  0.8981778 0.1046278
## Read:B-Passive:B       4.0776488  -4.2838754 12.4391729 0.7264811
## Math:NL-Passive:B    -12.4471154 -21.0216018 -3.8726290 0.0006003
## Passive:NL-Passive:B          NA          NA         NA        NA
## Read:NL-Passive:B    -11.4898785 -20.6322358 -2.3475212 0.0049453
## Math:NL-Read:B       -16.5247642 -25.0603344 -7.9891939 0.0000011
## Passive:NL-Read:B             NA          NA         NA        NA
## Read:NL-Read:B       -15.5675273 -24.6733957 -6.4616589 0.0000245
## Passive:NL-Math:NL            NA          NA         NA        NA
## Read:NL-Math:NL        0.9572368  -8.3445678 10.2590415 0.9996944
## Read:NL-Passive:NL            NA          NA         NA        NA
```

```
boxplot(SON_1st~Cond+Country,data=datComb, main="Abstract reasoning at T1", ylab ="Abstract reasoning (z-score)")
```

```
summary(aov(SON_1st~Cond*Country,data=datComb))
```

```
##               Df Sum Sq Mean Sq F value  Pr(>F)    
## Cond           2   7.71   3.857   4.226  0.0157 *  
## Country        1  14.36  14.363  15.735 9.6e-05 ***
## Cond:Country   1   0.00   0.004   0.004  0.9469    
## Residuals    242 220.91   0.913                    
## ---
## Signif. codes:  0 '***' 0.001 '**' 0.01 '*' 0.05 '.' 0.1 ' ' 1
```

### 1.4.2 ANALYSIS 1 - Belgian sample

This is the analysis for the Belgium sample (N = 161) which features passive (non-playing) and active (math) control conditions.

#### 1.4.2.1 Descriptives

Here, we show results of Chi-squared/Fisher’s exact test for Gender, Lang, Handedness and FR in the Belgian sample. The sample is balanced in that regard.

##### 1.4.2.1.1 Chi-square test Gender x Condition

```
chisq.test(matrix(c(21,31,21,35,24,29),ncol=3,dimnames=list(c("female","male"), c("Passive","Math","Read"))))
```

```
## 
##  Pearson's Chi-squared test
## 
## data:  matrix(c(21, 31, 21, 35, 24, 29), ncol = 3, dimnames = list(c("female",     "male"), c("Passive", "Math", "Read")))
## X-squared = 0.69368, df = 2, p-value = 0.7069
```

##### 1.4.2.1.2 Fisher’s exact test Multilingualism x Condition

```
fisher.test(matrix(c(6,46,3,53,2,51),ncol=3,dimnames=list(c("multilingual","monolingual"), c("Passive","Math","Read"))))
```

```
## 
##  Fisher's Exact Test for Count Data
## 
## data:  matrix(c(6, 46, 3, 53, 2, 51), ncol = 3, dimnames = list(c("multilingual", "monolingual"), c("Passive", "Math", "Read")))
## p-value = 0.2808
## alternative hypothesis: two.sided
```

##### 1.4.2.1.3 Fisher’s exact test Handedness x Condition

```
fisher.test(matrix(c(9,43,5,51,4,49),ncol=3,dimnames=list(c("lefthander","righthander"), c("Passive","Math","Read"))))
```

```
## 
##  Fisher's Exact Test for Count Data
## 
## data:  matrix(c(9, 43, 5, 51, 4, 49), ncol = 3, dimnames = list(c("lefthander", "righthander"), c("Passive", "Math", "Read")))
## p-value = 0.2677
## alternative hypothesis: two.sided
```

##### 1.4.2.1.4 Chi-square test Familial Risk x Condition

```
chisq.test(matrix(c(7,45,7,49,10,43),ncol=3,dimnames=list(c("namilial risk","no familial risk"), c("Passive","Math","Read"))))
```

```
## 
##  Pearson's Chi-squared test
## 
## data:  matrix(c(7, 45, 7, 49, 10, 43), ncol = 3, dimnames = list(c("namilial risk",     "no familial risk"), c("Passive", "Math", "Read")))
## X-squared = 0.99695, df = 2, p-value = 0.6075
```

---

ANOVAs checking group differences at T1 revealed effects of condition for LK and RANcT. Subsequeny Tukey HSD tests revealed that the reading group knew less letters than the passive group, and that the math group was faster at RAN colours than the reading group. In both cases the math and passive groups did not differ from one another.

```
summary(aov(T1AgeF~Cond,data=datB))
```

```
##              Df Sum Sq Mean Sq F value Pr(>F)
## Cond          2  0.322  0.1612    1.53   0.22
## Residuals   158 16.639  0.1053
```

```
summary(aov(T1.LetKen~Cond,data=datB))
```

```
##              Df Sum Sq Mean Sq F value Pr(>F)  
## Cond          2    246  123.15    3.22 0.0426 *
## Residuals   157   6004   38.24                 
## ---
## Signif. codes:  0 '***' 0.001 '**' 0.01 '*' 0.05 '.' 0.1 ' ' 1
## 1 observation deleted due to missingness
```

```
TukeyHSD(aov(T1.LetKen~Cond,data=datB))
```

```
##   Tukey multiple comparisons of means
##     95% family-wise confidence level
## 
## Fit: aov(formula = T1.LetKen ~ Cond, data = datB)
## 
## $Cond
##                   diff        lwr       upr     p adj
## Passive-Math  1.917367 -0.9149175  4.749651 0.2478947
## Read-Math    -1.130728 -3.9349044  1.673449 0.6069370
## Read-Passive -3.048095 -5.9183315 -0.177858 0.0345137
```

```
summary(aov(T1CF_TotalPc~Cond,data=datB))
```

```
##              Df Sum Sq Mean Sq F value Pr(>F)
## Cond          2    752   376.0   0.736   0.48
## Residuals   154  78630   510.6               
## 4 observations deleted due to missingness
```

```
summary(aov(T1PF_TotalPc~Cond,data=datB))
```

```
##              Df Sum Sq Mean Sq F value Pr(>F)
## Cond          2    771   385.3    0.53   0.59
## Residuals   158 114924   727.4
```

```
summary(aov(T1RANoT~Cond,data=datB))
```

```
##              Df Sum Sq Mean Sq F value Pr(>F)
## Cond          2   1229   614.6   1.574  0.211
## Residuals   157  61319   390.6               
## 1 observation deleted due to missingness
```

```
summary(aov(T1RANcT~Cond,data=datB))
```

```
##              Df Sum Sq Mean Sq F value Pr(>F)  
## Cond          2   1518   759.2   3.055 0.0499 *
## Residuals   158  39265   248.5                 
## ---
## Signif. codes:  0 '***' 0.001 '**' 0.01 '*' 0.05 '.' 0.1 ' ' 1
```

```
TukeyHSD(aov(T1RANcT~Cond,data=datB))
```

```
##   Tukey multiple comparisons of means
##     95% family-wise confidence level
## 
## Fit: aov(formula = T1RANcT ~ Cond, data = datB)
## 
## $Cond
##                  diff        lwr      upr     p adj
## Passive-Math 3.384615 -3.7984242 10.56765 0.5063501
## Read-Math    7.462264  0.3144481 14.61008 0.0385081
## Read-Passive 4.077649 -3.2026149 11.35791 0.3833327
```

```
summary(aov(SON_1st~Cond,data=datB))
```

```
##              Df Sum Sq Mean Sq F value Pr(>F)
## Cond          2    1.6  0.8008   0.799  0.452
## Residuals   158  158.4  1.0025
```

A summary of the Belgian data with number of observations, means, range and standard deviation per variable and condition.

```
tab <- tableby(Gender ~ EMT + Cond + T1AgeF + HandScore + FamRisk + Lang + SON_1st + hoursPlayed + levelsPlayed + sessionsPlayed + MaxLevel + itemsSeen + responsesGiven + T1CF_Total + T1PF_Total + T1.LetKen + T1RANcT + T1RANoT, data = datB, numeric.stats=c("meansd","medianrange"))
summary(tab, text=TRUE, digits=2, test=F)
```

```
## 
## 
## |                  |        female (N=66)        |         male (N=95)          |        Total (N=161)         |
## |:-----------------|:---------------------------:|:----------------------------:|:----------------------------:|
## |EMT               |                             |                              |                              |
## |-  Mean (SD)      |         0.14 (1.05)         |         -0.10 (0.95)         |         -0.00 (1.00)         |
## |-  Median (Range) |     0.06 (-1.48, 3.57)      |     -0.23 (-1.55, 4.38)      |     -0.16 (-1.55, 4.38)      |
## |Cond              |                             |                              |                              |
## |-  Math           |         21 (31.8%)          |          35 (36.8%)          |          56 (34.8%)          |
## |-  Passive        |         21 (31.8%)          |          31 (32.6%)          |          52 (32.3%)          |
## |-  Read           |         24 (36.4%)          |          29 (30.5%)          |          53 (32.9%)          |
## |T1AgeF            |                             |                              |                              |
## |-  Mean (SD)      |         6.23 (0.34)         |         6.28 (0.31)          |         6.26 (0.33)          |
## |-  Median (Range) |      6.22 (5.70, 7.39)      |      6.31 (5.68, 7.26)       |      6.26 (5.68, 7.39)       |
## |HandScore         |                             |                              |                              |
## |-  Mean (SD)      |         0.14 (0.83)         |         -0.10 (1.10)         |         0.00 (1.00)          |
## |-  Median (Range) |     0.39 (-2.88, 0.55)      |      0.39 (-3.05, 0.55)      |      0.39 (-3.05, 0.55)      |
## |FamRisk           |                             |                              |                              |
## |-  no             |         55 (83.3%)          |          82 (86.3%)          |         137 (85.1%)          |
## |-  yes            |         11 (16.7%)          |          13 (13.7%)          |          24 (14.9%)          |
## |Lang              |                             |                              |                              |
## |-  mono           |         62 (93.9%)          |          88 (92.6%)          |         150 (93.2%)          |
## |-  multi          |          4 (6.1%)           |           7 (7.4%)           |          11 (6.8%)           |
## |SON_1st           |                             |                              |                              |
## |-  Mean (SD)      |         0.23 (1.01)         |         -0.16 (0.96)         |         -0.00 (1.00)         |
## |-  Median (Range) |     0.25 (-2.28, 3.62)      |     -0.17 (-2.28, 1.93)      |     -0.17 (-2.28, 3.62)      |
## |hoursPlayed       |                             |                              |                              |
## |-  Mean (SD)      |         4.06 (0.75)         |         3.78 (0.78)          |         3.90 (0.78)          |
## |-  Median (Range) |      4.15 (2.12, 5.24)      |      3.99 (1.97, 5.18)       |      4.07 (1.97, 5.24)       |
## |levelsPlayed      |                             |                              |                              |
## |-  Mean (SD)      |       324.18 (140.67)       |       377.36 (192.48)        |       355.40 (174.26)        |
## |-  Median (Range) |   287.00 (132.00, 694.00)   |   323.50 (119.00, 1026.00)   |   309.00 (119.00, 1026.00)   |
## |sessionsPlayed    |                             |                              |                              |
## |-  Mean (SD)      |        28.13 (5.56)         |         27.53 (4.98)         |         27.78 (5.21)         |
## |-  Median (Range) |    27.00 (16.00, 37.00)     |     26.00 (19.00, 37.00)     |     26.00 (16.00, 37.00)     |
## |MaxLevel          |                             |                              |                              |
## |-  Mean (SD)      |       121.33 (49.05)        |        118.47 (38.82)        |        119.65 (43.14)        |
## |-  Median (Range) |   132.00 (38.00, 265.00)    |    122.50 (41.00, 201.00)    |    128.00 (38.00, 265.00)    |
## |itemsSeen         |                             |                              |                              |
## |-  Mean (SD)      |     10238.60 (3739.57)      |      10714.92 (4350.49)      |      10518.28 (4097.98)      |
## |-  Median (Range) | 9859.00 (2827.00, 18528.00) | 10349.00 (2983.00, 22782.00) | 10010.00 (2827.00, 22782.00) |
## |responsesGiven    |                             |                              |                              |
## |-  Mean (SD)      |      3397.51 (1647.90)      |      3904.17 (2205.07)       |      3695.00 (2001.38)       |
## |-  Median (Range) | 3039.00 (1019.00, 8746.00)  |  3147.00 (948.00, 10877.00)  |  3090.00 (948.00, 10877.00)  |
## |T1CF_Total        |                             |                              |                              |
## |-  Mean (SD)      |        24.72 (8.23)         |         21.75 (9.72)         |         22.96 (9.23)         |
## |-  Median (Range) |     26.00 (9.00, 40.00)     |     22.00 (1.00, 42.00)      |     24.00 (1.00, 42.00)      |
## |T1PF_Total        |                             |                              |                              |
## |-  Mean (SD)      |        31.11 (4.98)         |         28.87 (5.81)         |         29.79 (5.58)         |
## |-  Median (Range) |    31.50 (18.00, 40.00)     |     29.00 (0.00, 39.00)      |     30.00 (0.00, 40.00)      |
## |T1.LetKen         |                             |                              |                              |
## |-  Mean (SD)      |        10.92 (6.97)         |         8.68 (5.60)          |         9.59 (6.27)          |
## |-  Median (Range) |     10.00 (1.00, 26.00)     |      8.00 (0.00, 25.00)      |      8.00 (0.00, 26.00)      |
## |T1RANcT           |                             |                              |                              |
## |-  Mean (SD)      |        69.48 (15.16)        |        72.14 (16.49)         |        71.05 (15.97)         |
## |-  Median (Range) |    66.50 (42.00, 115.00)    |    70.00 (40.00, 122.00)     |    69.00 (40.00, 122.00)     |
## |T1RANoT           |                             |                              |                              |
## |-  Mean (SD)      |        75.08 (18.77)        |        79.76 (20.41)         |        77.86 (19.83)         |
## |-  Median (Range) |    72.00 (46.00, 127.00)    |    77.00 (48.00, 180.00)     |    76.00 (46.00, 180.00)     |
```

```
tab <- tableby(FamRisk ~ EMT + Cond + T1AgeF + HandScore + Gender + Lang + SON_1st + hoursPlayed + levelsPlayed + sessionsPlayed + MaxLevel + itemsSeen + responsesGiven + T1CF_Total + T1PF_Total + T1.LetKen + T1RANcT + T1RANoT, data = datB, numeric.stats=c("meansd","medianrange"))
summary(tab, text=TRUE, digits=2, test=F)
```

```
## 
## 
## |                  |          no (N=137)          |          yes (N=24)          |        Total (N=161)         |
## |:-----------------|:----------------------------:|:----------------------------:|:----------------------------:|
## |EMT               |                              |                              |                              |
## |-  Mean (SD)      |         0.04 (1.02)          |         -0.21 (0.86)         |         -0.00 (1.00)         |
## |-  Median (Range) |     -0.16 (-1.55, 4.38)      |     -0.30 (-1.55, 1.38)      |     -0.16 (-1.55, 4.38)      |
## |Cond              |                              |                              |                              |
## |-  Math           |          49 (35.8%)          |          7 (29.2%)           |          56 (34.8%)          |
## |-  Passive        |          45 (32.8%)          |          7 (29.2%)           |          52 (32.3%)          |
## |-  Read           |          43 (31.4%)          |          10 (41.7%)          |          53 (32.9%)          |
## |T1AgeF            |                              |                              |                              |
## |-  Mean (SD)      |         6.26 (0.31)          |         6.25 (0.40)          |         6.26 (0.33)          |
## |-  Median (Range) |      6.29 (5.68, 7.26)       |      6.22 (5.76, 7.39)       |      6.26 (5.68, 7.39)       |
## |HandScore         |                              |                              |                              |
## |-  Mean (SD)      |         0.01 (1.01)          |         -0.06 (0.96)         |         0.00 (1.00)          |
## |-  Median (Range) |      0.39 (-3.05, 0.55)      |      0.39 (-2.88, 0.55)      |      0.39 (-3.05, 0.55)      |
## |Gender            |                              |                              |                              |
## |-  female         |          55 (40.1%)          |          11 (45.8%)          |          66 (41.0%)          |
## |-  male           |          82 (59.9%)          |          13 (54.2%)          |          95 (59.0%)          |
## |Lang              |                              |                              |                              |
## |-  mono           |         128 (93.4%)          |          22 (91.7%)          |         150 (93.2%)          |
## |-  multi          |           9 (6.6%)           |           2 (8.3%)           |          11 (6.8%)           |
## |SON_1st           |                              |                              |                              |
## |-  Mean (SD)      |         0.01 (0.95)          |         -0.03 (1.28)         |         -0.00 (1.00)         |
## |-  Median (Range) |     -0.17 (-2.28, 1.93)      |      0.25 (-2.28, 3.62)      |     -0.17 (-2.28, 3.62)      |
## |hoursPlayed       |                              |                              |                              |
## |-  Mean (SD)      |         3.91 (0.80)          |         3.81 (0.67)          |         3.90 (0.78)          |
## |-  Median (Range) |      4.11 (2.12, 5.24)       |      3.89 (1.97, 4.68)       |      4.07 (1.97, 5.24)       |
## |levelsPlayed      |                              |                              |                              |
## |-  Mean (SD)      |       356.64 (172.20)        |       348.71 (190.40)        |       355.40 (174.26)        |
## |-  Median (Range) |   314.00 (132.00, 1026.00)   |   282.00 (119.00, 722.00)    |   309.00 (119.00, 1026.00)   |
## |sessionsPlayed    |                              |                              |                              |
## |-  Mean (SD)      |         27.90 (5.38)         |         27.12 (4.26)         |         27.78 (5.21)         |
## |-  Median (Range) |     26.00 (16.00, 37.00)     |     26.00 (22.00, 35.00)     |     26.00 (16.00, 37.00)     |
## |MaxLevel          |                              |                              |                              |
## |-  Mean (SD)      |        122.01 (40.98)        |        106.88 (52.98)        |        119.65 (43.14)        |
## |-  Median (Range) |    130.50 (38.00, 241.00)    |    94.00 (41.00, 265.00)     |    128.00 (38.00, 265.00)    |
## |itemsSeen         |                              |                              |                              |
## |-  Mean (SD)      |      10390.43 (3860.55)      |      11210.12 (5290.32)      |      10518.28 (4097.98)      |
## |-  Median (Range) | 10018.50 (2827.00, 21452.00) | 10010.00 (2983.00, 22782.00) | 10010.00 (2827.00, 22782.00) |
## |responsesGiven    |                              |                              |                              |
## |-  Mean (SD)      |      3638.86 (1840.26)       |      3998.82 (2767.65)       |      3695.00 (2001.38)       |
## |-  Median (Range) |  3106.50 (1019.00, 9826.00)  |  3090.00 (948.00, 10877.00)  |  3090.00 (948.00, 10877.00)  |
## |T1CF_Total        |                              |                              |                              |
## |-  Mean (SD)      |         23.34 (9.02)         |        20.88 (10.31)         |         22.96 (9.23)         |
## |-  Median (Range) |     24.00 (1.00, 42.00)      |     20.00 (1.00, 39.00)      |     24.00 (1.00, 42.00)      |
## |T1PF_Total        |                              |                              |                              |
## |-  Mean (SD)      |         30.00 (5.49)         |         28.58 (6.01)         |         29.79 (5.58)         |
## |-  Median (Range) |     30.00 (0.00, 40.00)      |     29.00 (11.00, 37.00)     |     30.00 (0.00, 40.00)      |
## |T1.LetKen         |                              |                              |                              |
## |-  Mean (SD)      |         9.72 (6.40)          |         8.88 (5.57)          |         9.59 (6.27)          |
## |-  Median (Range) |      8.00 (0.00, 26.00)      |      7.50 (1.00, 24.00)      |      8.00 (0.00, 26.00)      |
## |T1RANcT           |                              |                              |                              |
## |-  Mean (SD)      |        70.80 (16.33)         |        72.46 (13.92)         |        71.05 (15.97)         |
## |-  Median (Range) |    68.00 (40.00, 122.00)     |    72.00 (43.00, 106.00)     |    69.00 (40.00, 122.00)     |
## |T1RANoT           |                              |                              |                              |
## |-  Mean (SD)      |        76.78 (19.42)         |        83.96 (21.46)         |        77.86 (19.83)         |
## |-  Median (Range) |    76.00 (46.00, 180.00)     |    76.50 (58.00, 127.00)     |    76.00 (46.00, 180.00)     |
```

```
tab <- tableby(Cond ~ EMT + FamRisk + T1AgeF + HandScore + Gender + Lang + SON_1st + hoursPlayed + levelsPlayed + sessionsPlayed + MaxLevel + itemsSeen + responsesGiven + T1CF_Total + T1PF_Total + T1.LetKen + T1RANcT + T1RANoT, data = datB, numeric.stats=c("meansd","medianrange"))
summary(tab, text=TRUE, digits=2, test=F)
```

```
## 
## 
## |                  |         Math (N=56)         |    Passive (N=52)     |         Read (N=53)          |        Total (N=161)         |
## |:-----------------|:---------------------------:|:---------------------:|:----------------------------:|:----------------------------:|
## |EMT               |                             |                       |                              |                              |
## |-  Mean (SD)      |         0.06 (1.14)         |     -0.22 (0.67)      |         0.15 (1.09)          |         -0.00 (1.00)         |
## |-  Median (Range) |     -0.23 (-1.55, 4.38)     |  -0.16 (-1.33, 1.53)  |      0.13 (-1.55, 3.06)      |     -0.16 (-1.55, 4.38)      |
## |FamRisk           |                             |                       |                              |                              |
## |-  no             |         49 (87.5%)          |      45 (86.5%)       |          43 (81.1%)          |         137 (85.1%)          |
## |-  yes            |          7 (12.5%)          |       7 (13.5%)       |          10 (18.9%)          |          24 (14.9%)          |
## |T1AgeF            |                             |                       |                              |                              |
## |-  Mean (SD)      |         6.31 (0.31)         |      6.20 (0.30)      |         6.26 (0.37)          |         6.26 (0.33)          |
## |-  Median (Range) |      6.31 (5.70, 7.07)      |   6.17 (5.68, 6.80)   |      6.25 (5.71, 7.39)       |      6.26 (5.68, 7.39)       |
## |HandScore         |                             |                       |                              |                              |
## |-  Mean (SD)      |         0.11 (0.97)         |     -0.21 (1.23)      |         0.09 (0.73)          |         0.00 (1.00)          |
## |-  Median (Range) |     0.39 (-3.05, 0.55)      |  0.39 (-3.05, 0.55)   |      0.39 (-3.05, 0.55)      |      0.39 (-3.05, 0.55)      |
## |Gender            |                             |                       |                              |                              |
## |-  female         |         21 (37.5%)          |      21 (40.4%)       |          24 (45.3%)          |          66 (41.0%)          |
## |-  male           |         35 (62.5%)          |      31 (59.6%)       |          29 (54.7%)          |          95 (59.0%)          |
## |Lang              |                             |                       |                              |                              |
## |-  mono           |         53 (94.6%)          |      46 (88.5%)       |          51 (96.2%)          |         150 (93.2%)          |
## |-  multi          |          3 (5.4%)           |       6 (11.5%)       |           2 (3.8%)           |          11 (6.8%)           |
## |SON_1st           |                             |                       |                              |                              |
## |-  Mean (SD)      |         0.02 (1.06)         |     -0.13 (1.03)      |         0.11 (0.91)          |         -0.00 (1.00)         |
## |-  Median (Range) |     0.25 (-2.28, 3.62)      |  -0.38 (-2.28, 1.93)  |      0.25 (-2.28, 1.51)      |     -0.17 (-2.28, 3.62)      |
## |hoursPlayed       |                             |                       |                              |                              |
## |-  Mean (SD)      |         3.96 (0.76)         |          NA           |         3.83 (0.80)          |         3.90 (0.78)          |
## |-  Median (Range) |      4.17 (2.12, 5.24)      |          NA           |      3.98 (1.97, 5.23)       |      4.07 (1.97, 5.24)       |
## |levelsPlayed      |                             |                       |                              |                              |
## |-  Mean (SD)      |       473.43 (165.94)       |          NA           |        230.70 (55.57)        |       355.40 (174.26)        |
## |-  Median (Range) |  460.00 (132.00, 1026.00)   |          NA           |   228.00 (119.00, 369.00)    |   309.00 (119.00, 1026.00)   |
## |sessionsPlayed    |                             |                       |                              |                              |
## |-  Mean (SD)      |        27.82 (4.82)         |          NA           |         27.74 (5.64)         |         27.78 (5.21)         |
## |-  Median (Range) |    27.00 (18.00, 35.00)     |          NA           |     26.00 (16.00, 37.00)     |     26.00 (16.00, 37.00)     |
## |MaxLevel          |                             |                       |                              |                              |
## |-  Mean (SD)      |       136.84 (31.04)        |          NA           |        101.49 (46.82)        |        119.65 (43.14)        |
## |-  Median (Range) |   137.50 (39.00, 178.00)    |          NA           |    87.00 (38.00, 265.00)     |    128.00 (38.00, 265.00)    |
## |itemsSeen         |                             |                       |                              |                              |
## |-  Mean (SD)      |     10747.04 (4563.03)      |          NA           |      10276.57 (3569.36)      |      10518.28 (4097.98)      |
## |-  Median (Range) | 9805.50 (2827.00, 22782.00) |          NA           | 10145.00 (2983.00, 18124.00) | 10010.00 (2827.00, 22782.00) |
## |responsesGiven    |                             |                       |                              |                              |
## |-  Mean (SD)      |      4729.16 (2259.21)      |          NA           |       2602.30 (742.90)       |      3695.00 (2001.38)       |
## |-  Median (Range) | 4261.00 (1019.00, 10877.00) |          NA           |  2603.00 (948.00, 4072.00)   |  3090.00 (948.00, 10877.00)  |
## |T1CF_Total        |                             |                       |                              |                              |
## |-  Mean (SD)      |        23.44 (9.64)         |     23.38 (9.84)      |         22.08 (8.27)         |         22.96 (9.23)         |
## |-  Median (Range) |     26.00 (1.00, 40.00)     |  24.50 (1.00, 42.00)  |     23.00 (2.00, 39.00)      |     24.00 (1.00, 42.00)      |
## |T1PF_Total        |                             |                       |                              |                              |
## |-  Mean (SD)      |        29.93 (5.66)         |     30.21 (5.33)      |         29.23 (5.79)         |         29.79 (5.58)         |
## |-  Median (Range) |    30.00 (16.00, 40.00)     | 31.00 (11.00, 38.00)  |     30.00 (0.00, 37.00)      |     30.00 (0.00, 40.00)      |
## |T1.LetKen         |                             |                       |                              |                              |
## |-  Mean (SD)      |         9.36 (6.42)         |     11.27 (5.30)      |         8.23 (6.70)          |         9.59 (6.27)          |
## |-  Median (Range) |     8.00 (0.00, 26.00)      |  10.00 (0.00, 25.00)  |      6.00 (0.00, 25.00)      |      8.00 (0.00, 26.00)      |
## |T1RANcT           |                             |                       |                              |                              |
## |-  Mean (SD)      |        67.50 (15.37)        |     70.88 (15.92)     |        74.96 (16.02)         |        71.05 (15.97)         |
## |-  Median (Range) |    67.00 (40.00, 107.00)    | 67.50 (48.00, 115.00) |    70.00 (49.00, 122.00)     |    69.00 (40.00, 122.00)     |
## |T1RANoT           |                             |                       |                              |                              |
## |-  Mean (SD)      |        76.25 (17.33)        |     75.56 (17.49)     |        81.77 (23.82)         |        77.86 (19.83)         |
## |-  Median (Range) |    72.00 (50.00, 120.00)    | 76.50 (46.00, 127.00) |    76.00 (49.00, 180.00)     |    76.00 (46.00, 180.00)     |
```

#### 1.4.2.2 Correlations

Here, we show the correlation matrices of the main outcome variables and covariates at T1 and T2: EMT (one minute reading), CF\_Total (CELF PA raw scores), PF\_Total (PROEF PA raw scores), RAN (rapid automatized naming) of objects (o) or colors (c), as well as LK (in-game letter knowledge) and WLD (written lexical decision). The asterisk indicates significance at p < .05

```
corB <- subset(datB,select=c(EMT, T1CF_Total,T2CF_Total, T1PF_Total,T2PF_Total, T1RANcT, T2RANcT, T1RANoT, T2RANoT, T1.LetKen, T2.LetKen, T2.LexDec))
names(corB) <- c("T2EMT","T1CF","T2CF","T1PF","T2PF","T1RANc","T2RANc","T1RANo","T2RANo","T1LK","T2LK","T2WLD")
corBP <- subset(datB,Cond=="Passive",select=c(EMT, T1CF_Total,T2CF_Total, T1PF_Total,T2PF_Total, T1RANcT, T2RANcT, T1RANoT, T2RANoT, T1.LetKen, T2.LetKen, T2.LexDec))
names(corBP) <- c("T2EMT","T1CF","T2CF","T1PF","T2PF","T1RANc","T2RANc","T1RANo","T2RANo","T1LK","T2LK","T2WLD")
corBM <- subset(datB,Cond=="Math",select=c(EMT, T1CF_Total,T2CF_Total, T1PF_Total,T2PF_Total, T1RANcT, T2RANcT, T1RANoT, T2RANoT, T1.LetKen, T2.LetKen, T2.LexDec))
names(corBM) <- c("T2EMT","T1CF","T2CF","T1PF","T2PF","T1RANc","T2RANc","T1RANo","T2RANo","T1LK","T2LK","T2WLD")
corBR <- subset(datB,Cond=="Read",select=c(EMT, T1CF_Total,T2CF_Total, T1PF_Total,T2PF_Total, T1RANcT, T2RANcT, T1RANoT, T2RANoT, T1.LetKen, T2.LetKen, T2.LexDec))
names(corBR) <- c("T2EMT","T1CF","T2CF","T1PF","T2PF","T1RANc","T2RANc","T1RANo","T2RANo","T1LK","T2LK","T2WLD")
```

##### 1.4.2.2.1 Entire Belgian sample

```
##         T2EMT   T1CF   T2CF   T1PF   T2PF T1RANc T2RANc T1RANo T2RANo   T1LK   T2LK
## T2EMT                                                                              
## T1CF    0.49*                                                                      
## T2CF    0.33*  0.64*                                                               
## T1PF    0.18*  0.53*  0.57*                                                        
## T2PF    0.15   0.36*  0.65*  0.57*                                                 
## T1RANc -0.36* -0.35* -0.29* -0.28* -0.22*                                          
## T2RANc -0.38* -0.29* -0.23* -0.24* -0.19*  0.62*                                   
## T1RANo -0.31* -0.35* -0.28* -0.40* -0.25*  0.56*  0.54*                            
## T2RANo -0.34* -0.34* -0.32* -0.38* -0.25*  0.53*  0.65*  0.72*                     
## T1LK    0.32*  0.46*  0.29*  0.27*  0.15  -0.31* -0.33* -0.28* -0.29*              
## T2LK    0.23*  0.36*  0.32*  0.21*  0.17  -0.17  -0.31* -0.21* -0.29*  0.37*       
## T2WLD   0.37*  0.49*  0.33*  0.23*  0.15  -0.22* -0.22* -0.14  -0.31*  0.40*  0.55*
```

---

##### 1.4.2.2.2 Belgian Passive group

```
##         T2EMT   T1CF   T2CF   T1PF   T2PF T1RANc T2RANc T1RANo T2RANo   T1LK   T2LK
## T2EMT                                                                              
## T1CF    0.58*                                                                      
## T2CF    0.43*  0.71*                                                               
## T1PF    0.30*  0.68*  0.68*                                                        
## T2PF    0.35*  0.53*  0.75*  0.66*                                                 
## T1RANc -0.34* -0.27  -0.11  -0.25  -0.18                                           
## T2RANc -0.39* -0.20  -0.09  -0.18  -0.14   0.53*                                   
## T1RANo -0.34* -0.31* -0.22  -0.29* -0.25   0.69*  0.56*                            
## T2RANo -0.41* -0.32* -0.18  -0.23  -0.18   0.51*  0.76*  0.66*                     
## T1LK    0.31*  0.56*  0.40*  0.50*  0.26  -0.22  -0.32* -0.41* -0.36*              
## T2LK    0.40*  0.42*  0.43*  0.21   0.22  -0.18  -0.30* -0.28* -0.30*  0.44*       
## T2WLD   0.37*  0.55*  0.42*  0.43*  0.33* -0.21  -0.18  -0.15  -0.29*  0.39*  0.51*
```

---

##### 1.4.2.2.3 Belgian Math group

```
##         T2EMT   T1CF   T2CF   T1PF   T2PF T1RANc T2RANc T1RANo T2RANo   T1LK   T2LK
## T2EMT                                                                              
## T1CF    0.50*                                                                      
## T2CF    0.41*  0.68*                                                               
## T1PF    0.32*  0.60*  0.60*                                                        
## T2PF    0.25   0.24   0.52*  0.59*                                                 
## T1RANc -0.46* -0.36* -0.46* -0.29* -0.05                                           
## T2RANc -0.52* -0.47* -0.40* -0.24  -0.05   0.67*                                   
## T1RANo -0.41* -0.36* -0.28* -0.36* -0.04   0.49*  0.59*                            
## T2RANo -0.45* -0.41* -0.43* -0.31* -0.03   0.58*  0.57*  0.66*                     
## T1LK    0.39*  0.53*  0.38*  0.31*  0.11  -0.34* -0.41* -0.26  -0.34*              
## T2LK   -0.21   0.19   0.09  -0.05  -0.09   0.02  -0.23   0.29  -0.05   0.30        
## T2WLD   0.31   0.42   0.26  -0.07  -0.04  -0.39  -0.43*  0.16  -0.46*  0.25   0.57*
```

---

##### 1.4.2.2.4 Belgian Read group

```
##         T2EMT   T1CF   T2CF   T1PF   T2PF T1RANc T2RANc T1RANo T2RANo   T1LK   T2LK
## T2EMT                                                                              
## T1CF    0.52*                                                                      
## T2CF    0.19   0.50*                                                               
## T1PF   -0.01   0.30*  0.45*                                                        
## T2PF   -0.05   0.27   0.66*  0.46*                                                 
## T1RANc -0.34* -0.40* -0.31* -0.27  -0.34*                                          
## T2RANc -0.29* -0.21  -0.22  -0.28* -0.32*  0.66*                                   
## T1RANo -0.29* -0.39* -0.39* -0.49* -0.39*  0.51*  0.49*                            
## T2RANo -0.26  -0.25  -0.41* -0.56* -0.54*  0.50*  0.63*  0.79*                     
## T1LK    0.37*  0.32*  0.18   0.06   0.10  -0.33* -0.29* -0.19  -0.16               
## T2LK    0.46*  0.49*  0.40*  0.37*  0.34  -0.35* -0.44* -0.40* -0.48*  0.43*       
## T2WLD   0.49*  0.51*  0.28   0.18   0.07  -0.17  -0.16  -0.28  -0.21   0.59*  0.61*
```

#### 1.4.2.3 Reading Fluency

Single word reading fluency at T2 as measures by two custom lists with a time limit of one minute each.

```
## quartz_off_screen 
##                 2
```

```
## Linear mixed model fit by REML. t-tests use Satterthwaite's method ['lmerModLmerTest']
## Formula: EMT ~ log(T1RANcT) + T1CF_TotalZ + T1.LetKenZ + Cond + (1 | School)
##    Data: datBemt.2
## 
## REML criterion at convergence: 369.1
## 
## Scaled residuals: 
##     Min      1Q  Median      3Q     Max 
## -1.9137 -0.6467 -0.1702  0.4866  4.5527 
## 
## Random effects:
##  Groups   Name        Variance Std.Dev.
##  School   (Intercept) 0.08639  0.2939  
##  Residual             0.62233  0.7889  
## Number of obs: 150, groups:  School, 5
## 
## Fixed effects:
##               Estimate Std. Error        df t value Pr(>|t|)    
## (Intercept)    4.79126    1.39908 143.91640   3.425 0.000802 ***
## log(T1RANcT)  -1.13122    0.33013 143.01952  -3.427 0.000798 ***
## T1CF_TotalZ    0.26393    0.07987 143.58942   3.305 0.001201 ** 
## T1.LetKenZ     0.20405    0.07889 143.41895   2.587 0.010687 *  
## CondPassive   -0.27338    0.16820 141.78417  -1.625 0.106311    
## CondRead       0.27244    0.15977 141.49981   1.705 0.090351 .  
## ---
## Signif. codes:  0 '***' 0.001 '**' 0.01 '*' 0.05 '.' 0.1 ' ' 1
## 
## Correlation of Fixed Effects:
##             (Intr) l(T1RA T1CF_T T1.LKZ CndPss
## lg(T1RANcT) -0.992                            
## T1CF_TotalZ -0.235  0.230                     
## T1.LetKenZ  -0.140  0.147 -0.455              
## CondPassive  0.058 -0.117  0.038 -0.144       
## CondRead     0.116 -0.171  0.004 -0.002  0.452
```

```
## Explained variance
```

```
##             Class   Family     Link  Marginal Conditional      AIC
## 1 lmerModLmerTest gaussian identity 0.3014201   0.3865766 371.9283
```

```
## Sample size by condition
```

```
##    Math Passive    Read 
##      52      48      50
```

```
## Trimmed observations:
```

```
## [1] 7
```

```
## Trimmed in percent:
```

```
## [1] 4.347826
```

```
## `geom_smooth()` using formula 'y ~ x'
## `geom_smooth()` using formula 'y ~ x'
## `geom_smooth()` using formula 'y ~ x'
```

```
## `stat_bin()` using `bins = 30`. Pick better value with `binwidth`.
```

```
## `geom_smooth()` using formula 'y ~ x'
```

```
## Model summary for comparison Passive vs Read
```

```
## Linear mixed model fit by REML. t-tests use Satterthwaite's method ['lmerModLmerTest']
## Formula: EMT ~ log(T1RANcT) + T1CF_TotalZ + T1.LetKenZ + Cond + (1 | School)
##    Data: datBemt.2
## 
## REML criterion at convergence: 369.1
## 
## Scaled residuals: 
##     Min      1Q  Median      3Q     Max 
## -1.9137 -0.6467 -0.1702  0.4866  4.5527 
## 
## Random effects:
##  Groups   Name        Variance Std.Dev.
##  School   (Intercept) 0.08639  0.2939  
##  Residual             0.62233  0.7889  
## Number of obs: 150, groups:  School, 5
## 
## Fixed effects:
##               Estimate Std. Error        df t value Pr(>|t|)    
## (Intercept)    4.51788    1.41880 143.99618   3.184 0.001779 ** 
## log(T1RANcT)  -1.13122    0.33013 143.01951  -3.427 0.000798 ***
## T1CF_TotalZ    0.26393    0.07987 143.58942   3.305 0.001201 ** 
## T1.LetKenZ     0.20405    0.07889 143.41895   2.587 0.010687 *  
## CondMath       0.27338    0.16820 141.78417   1.625 0.106311    
## CondRead       0.54582    0.17178 141.96222   3.177 0.001823 ** 
## ---
## Signif. codes:  0 '***' 0.001 '**' 0.01 '*' 0.05 '.' 0.1 ' ' 1
## 
## Correlation of Fixed Effects:
##             (Intr) l(T1RA T1CF_T T1.LKZ CndMth
## lg(T1RANcT) -0.992                            
## T1CF_TotalZ -0.228  0.230                     
## T1.LetKenZ  -0.155  0.147 -0.455              
## CondMath    -0.176  0.117 -0.038  0.144       
## CondRead    -0.015 -0.045 -0.033  0.139  0.558
```

```
## Computing bootstrap confidence intervals ...
```

```
## 
## 102 message(s): boundary (singular) fit: see ?isSingular
```

```
##                    2.5 %     97.5 %
## .sig01        0.00000000  0.5604682
## .sigma        0.69568260  0.8750023
## (Intercept)   1.89213982  7.5270843
## log(T1RANcT) -1.76792106 -0.4506568
## T1CF_TotalZ   0.10769399  0.4363178
## T1.LetKenZ    0.04361214  0.3570556
## CondPassive  -0.59817112  0.0651892
## CondRead     -0.04605775  0.6038840
```

```
## Computing bootstrap confidence intervals ...
```

```
## 
## 84 message(s): boundary (singular) fit: see ?isSingular
```

```
##                    2.5 %     97.5 %
## .sig01        0.00000000  0.5612927
## .sigma        0.69123477  0.8786516
## (Intercept)   1.76481853  7.4670811
## log(T1RANcT) -1.83266666 -0.4714886
## T1CF_TotalZ   0.11412112  0.4225151
## T1.LetKenZ    0.05033333  0.3555478
## CondMath     -0.06478391  0.6242061
## CondRead      0.20327189  0.8639525
```

#### 1.4.2.4 PA

Phonological awareness at T2 as measured by the CELF-IV-NL and the PROEF.

##### 1.4.2.4.1 CELF-IV-NL Phonological Awareness

```
## quartz_off_screen 
##                 2
```

```
## Linear mixed model fit by REML. t-tests use Satterthwaite's method ['lmerModLmerTest']
## Formula: T2CF_TotalZ ~ SON_1st + T1PF_TotalZ + T1CF_TotalZ + Cond + (1 +      T1PF_TotalZ | School)
##    Data: datBcf.2
## Control: lmerControl(optimizer = "bobyqa", optCtrl = list(maxfun = 50000))
## 
## REML criterion at convergence: 314.7
## 
## Scaled residuals: 
##      Min       1Q   Median       3Q      Max 
## -3.00062 -0.59917  0.05576  0.76240  2.21515 
## 
## Random effects:
##  Groups   Name        Variance Std.Dev. Corr 
##  School   (Intercept) 0.05801  0.2409        
##           T1PF_TotalZ 0.05490  0.2343   -0.89
##  Residual             0.39822  0.6310        
## Number of obs: 152, groups:  School, 5
## 
## Fixed effects:
##              Estimate Std. Error        df t value Pr(>|t|)    
## (Intercept)   0.03990    0.14486   5.83354   0.275  0.79246    
## SON_1st       0.16690    0.06305 123.44667   2.647  0.00918 ** 
## T1PF_TotalZ   0.31602    0.12853   4.39290   2.459  0.06418 .  
## T1CF_TotalZ   0.43985    0.06831 141.91567   6.439 1.74e-09 ***
## CondPassive  -0.31207    0.13210 131.24033  -2.362  0.01963 *  
## CondRead     -0.10431    0.12541 141.89643  -0.832  0.40698    
## ---
## Signif. codes:  0 '***' 0.001 '**' 0.01 '*' 0.05 '.' 0.1 ' ' 1
## 
## Correlation of Fixed Effects:
##             (Intr) SON_1s T1PF_T T1CF_T CndPss
## SON_1st      0.016                            
## T1PF_TotalZ -0.574 -0.140                     
## T1CF_TotalZ -0.037 -0.361 -0.195              
## CondPassive -0.455  0.051 -0.024  0.004       
## CondRead    -0.429 -0.114  0.015  0.094  0.459
```

```
## Explained variance
```

```
## boundary (singular) fit: see ?isSingular
```

```
##             Class   Family     Link  Marginal Conditional      AIC
## 1 lmerModLmerTest gaussian identity 0.5420864   0.6406924 316.5835
```

```
## Sample size by condition
```

```
##    Math Passive    Read 
##      52      48      52
```

```
## Trimmed observations:
```

```
## [1] 5
```

```
## Trimmed in percent:
```

```
## [1] 3.10559
```

```
## `geom_smooth()` using formula 'y ~ x'
```

```
## `geom_smooth()` using formula 'y ~ x'
## `geom_smooth()` using formula 'y ~ x'
```

```
## `stat_bin()` using `bins = 30`. Pick better value with `binwidth`.
```

```
## `geom_smooth()` using formula 'y ~ x'
```

```
## Model summary for comparison Passive vs Read
```

```
## Linear mixed model fit by REML. t-tests use Satterthwaite's method ['lmerModLmerTest']
## Formula: T2CF_TotalZ ~ SON_1st + T1PF_TotalZ + T1CF_TotalZ + Cond + (1 +      T1PF_TotalZ | School)
##    Data: datBcf.2
## Control: lmerControl(optimizer = "bobyqa", optCtrl = list(maxfun = 50000))
## 
## REML criterion at convergence: 314.7
## 
## Scaled residuals: 
##      Min       1Q   Median       3Q      Max 
## -3.00062 -0.59917  0.05576  0.76240  2.21515 
## 
## Random effects:
##  Groups   Name        Variance Std.Dev. Corr 
##  School   (Intercept) 0.05801  0.2409        
##           T1PF_TotalZ 0.05490  0.2343   -0.89
##  Residual             0.39822  0.6310        
## Number of obs: 152, groups:  School, 5
## 
## Fixed effects:
##              Estimate Std. Error        df t value Pr(>|t|)    
## (Intercept)  -0.27217    0.14495   6.33391  -1.878  0.10693    
## SON_1st       0.16690    0.06305 123.44666   2.647  0.00918 ** 
## T1PF_TotalZ   0.31602    0.12853   4.39290   2.459  0.06418 .  
## T1CF_TotalZ   0.43985    0.06831 141.91566   6.439 1.74e-09 ***
## CondMath      0.31207    0.13210 131.24033   2.362  0.01963 *  
## CondRead      0.20776    0.13399 131.06617   1.551  0.12342    
## ---
## Signif. codes:  0 '***' 0.001 '**' 0.01 '*' 0.05 '.' 0.1 ' ' 1
## 
## Correlation of Fixed Effects:
##             (Intr) SON_1s T1PF_T T1CF_T CndMth
## SON_1st      0.062                            
## T1PF_TotalZ -0.596 -0.140                     
## T1CF_TotalZ -0.033 -0.361 -0.195              
## CondMath    -0.456 -0.051  0.024 -0.004       
## CondRead    -0.460 -0.157  0.038  0.084  0.556
```

```
## Computing bootstrap confidence intervals ...
```

```
## 
## 711 message(s): boundary (singular) fit: see ?isSingular
## 36 warning(s): NA values in sdcor matrix converted to 0
```

```
##                    2.5 %      97.5 %
## .sig01       0.000000000  0.45369374
## .sig02      -1.000000000  1.00000000
## .sig03       0.005644197  0.44941338
## .sigma       0.554702518  0.69969505
## (Intercept) -0.239423581  0.32319262
## SON_1st      0.044165615  0.29130676
## T1PF_TotalZ  0.060609227  0.57339020
## T1CF_TotalZ  0.298514714  0.57251358
## CondPassive -0.563894302 -0.04320932
## CondRead    -0.344376944  0.14333415
```

```
## Computing bootstrap confidence intervals ...
```

```
## 
## 693 message(s): boundary (singular) fit: see ?isSingular
## 33 warning(s): NA values in sdcor matrix converted to 0
```

```
##                    2.5 %      97.5 %
## .sig01       0.000000000 0.461607244
## .sig02      -1.000000000 1.000000000
## .sig03       0.007060223 0.454370049
## .sigma       0.550815859 0.706469612
## (Intercept) -0.562552031 0.003501017
## SON_1st      0.033879098 0.292656778
## T1PF_TotalZ  0.080863485 0.564718194
## T1CF_TotalZ  0.304782446 0.588459296
## CondMath     0.047141815 0.572305155
## CondRead    -0.063046567 0.473183876
```

##### 1.4.2.4.2 PROEF Phonological Awareness

```
## quartz_off_screen 
##                 2
```

```
## Linear mixed model fit by REML. t-tests use Satterthwaite's method ['lmerModLmerTest']
## Formula: T2PF_TotalZ ~ T1CF_TotalPc + Cond + FamRisk + T1PF_TotalZ + (1 +      T1PF_TotalZ | School)
##    Data: datBpf.2
## Control: lmerControl(optimizer = "bobyqa", optCtrl = list(maxfun = 50000))
## 
## REML criterion at convergence: 350.1
## 
## Scaled residuals: 
##     Min      1Q  Median      3Q     Max 
## -3.6718 -0.4615 -0.0136  0.5862  2.4022 
## 
## Random effects:
##  Groups   Name        Variance Std.Dev. Corr
##  School   (Intercept) 0.05968  0.2443       
##           T1PF_TotalZ 0.11580  0.3403   0.34
##  Residual             0.49597  0.7043       
## Number of obs: 150, groups:  School, 5
## 
## Fixed effects:
##                Estimate Std. Error         df t value Pr(>|t|)  
## (Intercept)   -0.066667   0.197777  17.695726  -0.337   0.7400  
## T1CF_TotalPc   0.006442   0.003029 142.806595   2.127   0.0352 *
## CondPassive   -0.210174   0.150662 139.097203  -1.395   0.1652  
## CondRead      -0.199270   0.140761 138.159598  -1.416   0.1591  
## FamRiskyes    -0.234602   0.175181 140.521442  -1.339   0.1827  
## T1PF_TotalZ    0.405484   0.174501   4.158058   2.324   0.0783 .
## ---
## Signif. codes:  0 '***' 0.001 '**' 0.01 '*' 0.05 '.' 0.1 ' ' 1
## 
## Correlation of Fixed Effects:
##             (Intr) T1CF_T CndPss CondRd FmRsky
## T1CF_TotlPc -0.618                            
## CondPassive -0.362 -0.011                     
## CondRead    -0.351  0.018  0.453              
## FamRiskyes  -0.174  0.066  0.004 -0.070       
## T1PF_TotalZ  0.288 -0.199 -0.042 -0.013  0.037
```

```
## Explained variance
```

```
##             Class   Family     Link  Marginal Conditional      AIC
## 1 lmerModLmerTest gaussian identity 0.2854256    0.472845 350.0313
```

```
## Sample size by condition
```

```
##    Math Passive    Read 
##      52      47      51
```

```
## Trimmed observations:
```

```
## [1] 7
```

```
## Trimmed in percent:
```

```
## [1] 4.347826
```

```
## `geom_smooth()` using formula 'y ~ x'
## `geom_smooth()` using formula 'y ~ x'
## `geom_smooth()` using formula 'y ~ x'
```

```
## `stat_bin()` using `bins = 30`. Pick better value with `binwidth`.
```

```
## `geom_smooth()` using formula 'y ~ x'
```

```
## Model summary for comparison Passive vs Read
```

```
## Linear mixed model fit by REML. t-tests use Satterthwaite's method ['lmerModLmerTest']
## Formula: T2PF_TotalZ ~ T1CF_TotalPc + Cond + FamRisk + T1PF_TotalZ + (1 +      T1PF_TotalZ | School)
##    Data: datBpf.2
## Control: lmerControl(optimizer = "bobyqa", optCtrl = list(maxfun = 50000))
## 
## REML criterion at convergence: 350.1
## 
## Scaled residuals: 
##     Min      1Q  Median      3Q     Max 
## -3.6718 -0.4615 -0.0136  0.5862  2.4022 
## 
## Random effects:
##  Groups   Name        Variance Std.Dev. Corr
##  School   (Intercept) 0.05968  0.2443       
##           T1PF_TotalZ 0.11580  0.3403   0.34
##  Residual             0.49597  0.7043       
## Number of obs: 150, groups:  School, 5
## 
## Fixed effects:
##                Estimate Std. Error         df t value Pr(>|t|)  
## (Intercept)   -0.276841   0.200536  20.678106  -1.381   0.1822  
## T1CF_TotalPc   0.006442   0.003029 142.806595   2.127   0.0352 *
## CondMath       0.210174   0.150662 139.097199   1.395   0.1652  
## CondRead       0.010904   0.152675 136.232318   0.071   0.9432  
## FamRiskyes    -0.234602   0.175181 140.521441  -1.339   0.1827  
## T1PF_TotalZ    0.405484   0.174501   4.158060   2.324   0.0783 .
## ---
## Signif. codes:  0 '***' 0.001 '**' 0.01 '*' 0.05 '.' 0.1 ' ' 1
## 
## Correlation of Fixed Effects:
##             (Intr) T1CF_T CndMth CondRd FmRsky
## T1CF_TotlPc -0.618                            
## CondMath    -0.394  0.011                     
## CondRead    -0.394  0.027  0.569              
## FamRiskyes  -0.169  0.066 -0.004 -0.068       
## T1PF_TotalZ  0.253 -0.199  0.042  0.029  0.037
```

```
## Computing bootstrap confidence intervals ...
```

```
## 
## 433 message(s): boundary (singular) fit: see ?isSingular
## 43 warning(s): NA values in sdcor matrix converted to 0
```

```
##                      2.5 %     97.5 %
## .sig01        0.0000000000 0.48269234
## .sig02       -1.0000000000 1.00000000
## .sig03        0.0199435963 0.60276719
## .sigma        0.6154907740 0.78432781
## (Intercept)  -0.4776526349 0.34247497
## T1CF_TotalPc  0.0003218559 0.01250006
## CondPassive  -0.5259308555 0.08974560
## CondRead     -0.4833103421 0.08202462
## FamRiskyes   -0.5754436678 0.10933238
## T1PF_TotalZ   0.0752537922 0.74123204
```

```
## Computing bootstrap confidence intervals ...
```

```
## 
## 480 message(s): boundary (singular) fit: see ?isSingular
## 44 warning(s): NA values in sdcor matrix converted to 0
```

```
##                      2.5 %     97.5 %
## .sig01        0.0000000000 0.45937205
## .sig02       -1.0000000000 1.00000000
## .sig03        0.0262184040 0.64838874
## .sigma        0.6197543955 0.78553786
## (Intercept)  -0.6785631756 0.12283451
## T1CF_TotalPc  0.0004110071 0.01231995
## CondMath     -0.0871827590 0.52842200
## CondRead     -0.2985500670 0.30758592
## FamRiskyes   -0.6167136423 0.09877992
## T1PF_TotalZ   0.0518092141 0.75605138
```

#### 1.4.2.5 RAN

Rapid Automatized Naming of objects and colours at T2. Both of these models are problematic as they show a high multicollinearity of the Cond\*PreTest interaction - indicating that there is not much change from pre to post which can be attributed to condition.

##### 1.4.2.5.1 RAN colours

```
## 
## Call:
## lm(formula = T2RANcT ~ Cond * T1RANcT, data = datBrC.2)
## 
## Residuals:
##      Min       1Q   Median       3Q      Max 
## -16.5929  -5.7267   0.3601   4.6996  19.2800 
## 
## Coefficients:
##                     Estimate Std. Error t value Pr(>|t|)    
## (Intercept)         22.48504    4.89891   4.590 9.58e-06 ***
## CondPassive          1.27480    7.54872   0.169    0.866    
## CondRead            -5.44453    7.35907  -0.740    0.461    
## T1RANcT              0.48943    0.07087   6.906 1.48e-10 ***
## CondPassive:T1RANcT -0.01589    0.10812  -0.147    0.883    
## CondRead:T1RANcT     0.05551    0.10063   0.552    0.582    
## ---
## Signif. codes:  0 '***' 0.001 '**' 0.01 '*' 0.05 '.' 0.1 ' ' 1
## 
## Residual standard error: 8.069 on 144 degrees of freedom
## Multiple R-squared:  0.4964, Adjusted R-squared:  0.4789 
## F-statistic: 28.39 on 5 and 144 DF,  p-value: < 2.2e-16
```

```
## `geom_smooth()` using formula 'y ~ x'
## `geom_smooth()` using formula 'y ~ x'
## `geom_smooth()` using formula 'y ~ x'
```

```
## `stat_bin()` using `bins = 30`. Pick better value with `binwidth`.
```

```
##                           2.5 %     97.5 %
## (Intercept)          12.8019680 32.1681040
## CondPassive         -13.6458088 16.1954113
## CondRead            -19.9902946  9.1012287
## T1RANcT               0.3493538  0.6295094
## CondPassive:T1RANcT  -0.2295942  0.1978137
## CondRead:T1RANcT     -0.1433869  0.2544055
```

##### 1.4.2.5.2 RAN objects

```
## 
## Call:
## lm(formula = T2RANcT ~ Cond * T1RANcT, data = datBrO.2)
## 
## Residuals:
##      Min       1Q   Median       3Q      Max 
## -23.5222  -6.3315   0.3091   5.4736  24.1091 
## 
## Coefficients:
##                     Estimate Std. Error t value Pr(>|t|)    
## (Intercept)         23.45397    5.94122   3.948 0.000126 ***
## CondPassive          5.50818    8.92403   0.617 0.538108    
## CondRead            -8.99419    9.16830  -0.981 0.328317    
## T1RANcT              0.47995    0.08758   5.480 1.98e-07 ***
## CondPassive:T1RANcT -0.09689    0.12980  -0.746 0.456647    
## CondRead:T1RANcT     0.12044    0.12743   0.945 0.346249    
## ---
## Signif. codes:  0 '***' 0.001 '**' 0.01 '*' 0.05 '.' 0.1 ' ' 1
## 
## Residual standard error: 9.099 on 137 degrees of freedom
##   (1 observation deleted due to missingness)
## Multiple R-squared:  0.4051, Adjusted R-squared:  0.3834 
## F-statistic: 18.66 on 5 and 137 DF,  p-value: 4.148e-14
```

```
## `geom_smooth()` using formula 'y ~ x'
## `geom_smooth()` using formula 'y ~ x'
## `geom_smooth()` using formula 'y ~ x'
```

```
## `stat_bin()` using `bins = 30`. Pick better value with `binwidth`.
```

```
##                           2.5 %     97.5 %
## (Intercept)          11.7056169 35.2023281
## CondPassive         -12.1384819 23.1548350
## CondRead            -27.1238727  9.1354980
## T1RANcT               0.3067638  0.6531395
## CondPassive:T1RANcT  -0.3535560  0.1597709
## CondRead:T1RANcT     -0.1315465  0.3724298
```

#### 1.4.2.6 In-Game

Apart from the offline pencil and paper tests in 4.1.3 and 4.1.4 we also did in-game assessments in form of letter-speech-sound-identification (LSSI) and written lexical decision (WLD). Both of these feature seperate analyses for accuracy and response times.

##### 1.4.2.6.1 LSSI Accuracy

```
accLKB <-glmer(correctSelections ~ lvl + trialRTs + T1CF_TotalZ + stream * Cond + (1+trialRTs|correctResponse) + (0+T1CF_TotalZ|correctResponse) + (1|Subj), family=binomial, data=LKaccB, glmerControl(optimizer="bobyqa",optCtrl=list(maxfun=50000) ))
LKaccB.2 = LKaccB[abs(scale(resid(accLKB))) < 2 , ]
accLKB.2 <-glmer(correctSelections ~ lvl + trialRTs + T1CF_TotalZ + stream * Cond + (1+trialRTs|correctResponse) + (0+T1CF_TotalZ|correctResponse) + (1|Subj), family=binomial, data=LKaccB.2, glmerControl(optimizer="bobyqa",optCtrl=list(maxfun=50000) ))
LKaccB.2$Cond <- relevel(LKaccB.2$Cond,ref="Passive") # refit the model to get passive as baseline for plotting
accLKB.3 <-glmer(correctSelections ~ lvl + trialRTs + T1CF_TotalZ + stream * Cond + (1+trialRTs|correctResponse) + (0+T1CF_TotalZ|correctResponse) + (1|Subj), family=binomial, data=LKaccB.2, glmerControl(optimizer="bobyqa",optCtrl=list(maxfun=50000) ))
plot(effect("stream*Cond",accLKB.3), ylab="probability of correct response", main="Letter Knowledge Accuracy",
     multiline=TRUE, ci.style="bars", xlab="Session",lines=list(col=c("black"),lty=c(1,2,3)),x.var="stream",
     lattice=list(key.args=list(title="Condition",x=.05, y=.95,cex=1.2,cex.title=1.2)))
```

```
pdf("Ch3_B_LSSIacc.pdf", width=6,height=4)
plot(effect("stream*Cond",accLKB.3), ylab="probability of correct response", main="",
     multiline=TRUE, ci.style="bars", xlab="Session",lines=list(col=c("black"),lty=c(1,2,3)),x.var="stream",
     lattice=list(key.args=list(title="Condition",x=.05, y=.95,cex=1.2,cex.title=1.2)))
dev.off()
```

```
## quartz_off_screen 
##                 2
```

```
summary(accLKB.2)
```

```
## Generalized linear mixed model fit by maximum likelihood (Laplace Approximation) ['glmerMod']
##  Family: binomial  ( logit )
## Formula: correctSelections ~ lvl + trialRTs + T1CF_TotalZ + stream * Cond +      (1 + trialRTs | correctResponse) + (0 + T1CF_TotalZ | correctResponse) +      (1 | Subj)
##    Data: LKaccB.2
## Control: glmerControl(optimizer = "bobyqa", optCtrl = list(maxfun = 50000))
## 
##      AIC      BIC   logLik deviance df.resid 
##   7061.5   7157.0  -3516.8   7033.5     6742 
## 
## Scaled residuals: 
##     Min      1Q  Median      3Q     Max 
## -7.5020 -0.6442  0.2467  0.5983  6.5378 
## 
## Random effects:
##  Groups            Name        Variance Std.Dev. Corr 
##  Subj              (Intercept) 0.534012 0.73076       
##  correctResponse   T1CF_TotalZ 0.028902 0.17001       
##  correctResponse.1 (Intercept) 1.476235 1.21500       
##                    trialRTs    0.002834 0.05324  -0.80
## Number of obs: 6756, groups:  Subj, 101; correctResponse, 32
## 
## Fixed effects:
##                      Estimate Std. Error z value Pr(>|z|)    
## (Intercept)          -0.32991    0.29537  -1.117  0.26403    
## lvlLetKenEasy         0.16466    0.08721   1.888  0.05902 .  
## trialRTs             -0.07127    0.01357  -5.252 1.51e-07 ***
## T1CF_TotalZ           0.69394    0.08991   7.719 1.18e-14 ***
## streamT2              1.77871    0.14021  12.686  < 2e-16 ***
## CondPassive           0.52582    0.22209   2.368  0.01790 *  
## CondRead              0.02034    0.24006   0.085  0.93246    
## streamT2:CondPassive -0.49154    0.16298  -3.016  0.00256 ** 
## streamT2:CondRead     0.33767    0.17975   1.878  0.06031 .  
## ---
## Signif. codes:  0 '***' 0.001 '**' 0.01 '*' 0.05 '.' 0.1 ' ' 1
## 
## Correlation of Fixed Effects:
##             (Intr) lvlLKE trlRTs T1CF_T strmT2 CndPss CondRd sT2:CP
## lvlLetKnEsy -0.227                                                 
## trialRTs    -0.499 -0.068                                          
## T1CF_TotalZ  0.010  0.004 -0.018                                   
## streamT2    -0.218 -0.033  0.049  0.047                            
## CondPassive -0.519 -0.005 -0.004  0.058  0.289                     
## CondRead    -0.482  0.004 -0.002  0.113  0.265  0.649              
## strmT2:CndP  0.182  0.025 -0.023 -0.020 -0.845 -0.343 -0.227       
## strmT2:CndR  0.168  0.008 -0.023 -0.002 -0.758 -0.221 -0.355  0.654
```

```
writeLines('Explained variance')
```

```
## Explained variance
```

```
rsquared.glmm(list(accLKB.2))
```

```
##      Class   Family  Link Marginal Conditional      AIC
## 1 glmerMod binomial logit 0.206102   0.4748549 7061.514
```

```
writeLines('Sample size by condition')
```

```
## Sample size by condition
```

```
summary(aggregate(Cond~Subj,data=accLKB.2@frame,FUN=unique)$Cond)
```

```
##    Math Passive    Read 
##      21      47      33
```

```
writeLines('Trimmed observations:')
```

```
## Trimmed observations:
```

```
nrow(LKaccB[abs(scale(resid(accLKB))) > 2 , ])
```

```
## [1] 132
```

```
writeLines('Trimmed in percent:')
```

```
## Trimmed in percent:
```

```
(1-nrow(LKaccB[abs(scale(resid(accLKB))) < 2 , ]) / nrow(LKaccB)) * 100
```

```
## [1] 1.844606
```

```
(accLKB.2bs <- confint(accLKB.2, method = "boot", nsim = simulations, level = 0.95, parallel = bootparallel, ncpus = bootthreads))
```

```
##                            2.5 %      97.5 %
## .sig01                0.58851243  0.85120574
## .sig02                0.03246917  0.25017349
## .sig03                0.89060569  1.54219520
## .sig04               -1.00000000 -0.46767888
## .sig05                0.02427070  0.07600698
## (Intercept)          -0.88411879  0.32543645
## lvlLetKenEasy        -0.01145247  0.33758540
## trialRTs             -0.09758046 -0.04390026
## T1CF_TotalZ           0.51717649  0.88225595
## streamT2              1.51287569  2.06082365
## CondPassive           0.07702440  0.95293074
## CondRead             -0.44700271  0.48952336
## streamT2:CondPassive -0.83244064 -0.18793447
## streamT2:CondRead    -0.01930842  0.67953986
```

```
summary(accLKB.3)
```

```
## Generalized linear mixed model fit by maximum likelihood (Laplace Approximation) ['glmerMod']
##  Family: binomial  ( logit )
## Formula: correctSelections ~ lvl + trialRTs + T1CF_TotalZ + stream * Cond +      (1 + trialRTs | correctResponse) + (0 + T1CF_TotalZ | correctResponse) +      (1 | Subj)
##    Data: LKaccB.2
## Control: glmerControl(optimizer = "bobyqa", optCtrl = list(maxfun = 50000))
## 
##      AIC      BIC   logLik deviance df.resid 
##   7061.5   7157.0  -3516.8   7033.5     6742 
## 
## Scaled residuals: 
##     Min      1Q  Median      3Q     Max 
## -7.5020 -0.6442  0.2467  0.5983  6.5378 
## 
## Random effects:
##  Groups            Name        Variance Std.Dev. Corr 
##  Subj              (Intercept) 0.534014 0.73076       
##  correctResponse   T1CF_TotalZ 0.028901 0.17000       
##  correctResponse.1 (Intercept) 1.476270 1.21502       
##                    trialRTs    0.002834 0.05324  -0.80
## Number of obs: 6756, groups:  Subj, 101; correctResponse, 32
## 
## Fixed effects:
##                   Estimate Std. Error z value Pr(>|z|)    
## (Intercept)        0.19591    0.26160   0.749  0.45392    
## lvlLetKenEasy      0.16466    0.08721   1.888  0.05902 .  
## trialRTs          -0.07127    0.01357  -5.252 1.51e-07 ***
## T1CF_TotalZ        0.69394    0.08991   7.719 1.18e-14 ***
## streamT2           1.28717    0.08707  14.783  < 2e-16 ***
## CondMath          -0.52581    0.22210  -2.367  0.01791 *  
## CondRead          -0.50547    0.19439  -2.600  0.00931 ** 
## streamT2:CondMath  0.49155    0.16299   3.016  0.00256 ** 
## streamT2:CondRead  0.82920    0.14329   5.787 7.17e-09 ***
## ---
## Signif. codes:  0 '***' 0.001 '**' 0.01 '*' 0.05 '.' 0.1 ' ' 1
## 
## Correlation of Fixed Effects:
##             (Intr) lvlLKE trlRTs T1CF_T strmT2 CndMth CondRd sT2:CM
## lvlLetKnEsy -0.260                                                 
## trialRTs    -0.567 -0.068                                          
## T1CF_TotalZ  0.060  0.004 -0.018                                   
## streamT2    -0.160 -0.007  0.035  0.037                            
## CondMath    -0.263  0.005  0.004 -0.058  0.176                     
## CondRead    -0.292  0.010  0.002  0.074  0.203  0.341              
## strmT2:CndM  0.085 -0.025  0.023  0.021 -0.510 -0.343 -0.111       
## strmT2:CndR  0.100 -0.018 -0.002  0.021 -0.575 -0.113 -0.360  0.317
```

```
(accLKB.3bs <- confint(accLKB.3, method = "boot", nsim = simulations, level = 0.95, parallel = bootparallel, ncpus = bootthreads))
```

```
##                         2.5 %      97.5 %
## .sig01             0.59560388  0.83133318
## .sig02             0.02222055  0.25507792
## .sig03             0.85457745  1.51701000
## .sig04            -1.00000000 -0.45365277
## .sig05             0.02338274  0.07390020
## (Intercept)       -0.30318317  0.73641901
## lvlLetKenEasy     -0.01075068  0.35034616
## trialRTs          -0.09971771 -0.04560826
## T1CF_TotalZ        0.52250826  0.87967190
## streamT2           1.11587513  1.46490254
## CondMath          -0.99941934 -0.09489142
## CondRead          -0.86550616 -0.12942681
## streamT2:CondMath  0.17570747  0.82409193
## streamT2:CondRead  0.54412934  1.11779943
```

##### 1.4.2.6.2 LSSI Response Time

```
bc <- MASS::boxcox(lm(trialRTs ~ lvl + log(T1RANcT) + T1.LetKenZ + TrialNum + prevRT + stream * Cond, data=LKrtB),plotit=FALSE)
lambda <- bc$x[which.max(bc$y)]
LKrtB$RTinv = LKrtB$trialRTs^lambda
LKrtB$prevRTinv = LKrtB$prevRT^lambda
rtLKB = lmer(RTinv ~ lvl + log(T1RANcT) + T1.LetKenZ + TrialNum + prevRTinv + stream * Cond + (1|itemsOnScreen) + (0+stream|Subj) + (1|correctResponse) + (1|Class), data=LKrtB)
LKrtB.2 = LKrtB[abs(scale(resid(rtLKB))) < 2 , ]
rtLKB.2 = lmer(RTinv ~ lvl + log(T1RANcT) + T1.LetKenZ + TrialNum + prevRTinv + stream * Cond + (1|itemsOnScreen) + (0+stream|Subj) + (1|correctResponse) + (1|Class), data=LKrtB.2 )
LKrtB.2$Cond <- relevel(LKrtB.2$Cond,ref="Passive") # refit the model to get passive as baseline for plotting
rtLKB.3 = lmer(RTinv ~ lvl + log(T1RANcT) + T1.LetKenZ + TrialNum + prevRTinv + stream * Cond + (1|itemsOnScreen) + (0+stream|Subj) + (1|correctResponse) + (1|Class), data=LKrtB.2)

LKrtef = effect("stream*Cond",rtLKB.3)
LKrtef$fit = LKrtef$fit^(1/lambda)
LKrtef$upper = LKrtef$upper^(1/lambda)
LKrtef$lower = LKrtef$lower^(1/lambda)
plot(LKrtef, ylab="Median response time (seconds)", main="Letter Knowledge Response Times", multiline=TRUE,
     confint=list(style="bars"), xlab="Session", lines=list(col=c("black"), lty=c(1,2,3)),x.var="stream",
     lattice=list(key.args=list(title="Condition",x=.62, y=.95,cex=1.2,cex.title=1.2)))
```

```
summary(rtLKB.2)
```

```
## Linear mixed model fit by REML. t-tests use Satterthwaite's method ['lmerModLmerTest']
## Formula: RTinv ~ lvl + log(T1RANcT) + T1.LetKenZ + TrialNum + prevRTinv +      stream * Cond + (1 | itemsOnScreen) + (0 + stream | Subj) +      (1 | correctResponse) + (1 | Class)
##    Data: LKrtB.2
## 
## REML criterion at convergence: -12039.9
## 
## Scaled residuals: 
##     Min      1Q  Median      3Q     Max 
## -3.3888 -0.6410  0.0046  0.6614  3.8514 
## 
## Random effects:
##  Groups          Name        Variance  Std.Dev. Corr
##  itemsOnScreen   (Intercept) 8.017e-05 0.008954     
##  Subj            streamT1    1.852e-04 0.013610     
##                  streamT2    1.350e-04 0.011618 0.47
##  correctResponse (Intercept) 1.745e-04 0.013211     
##  Class           (Intercept) 2.191e-05 0.004681     
##  Residual                    1.306e-03 0.036136     
## Number of obs: 3279, groups:  itemsOnScreen, 741; Subj, 105; correctResponse, 30; Class, 11
## 
## Fixed effects:
##                        Estimate Std. Error         df t value Pr(>|t|)    
## (Intercept)           9.373e-01  2.800e-02  1.135e+02  33.482  < 2e-16 ***
## lvlLetKenEasy        -1.496e-02  5.610e-03  2.828e+00  -2.667 0.080853 .  
## log(T1RANcT)         -1.500e-02  6.460e-03  9.404e+01  -2.322 0.022380 *  
## T1.LetKenZ            6.401e-03  1.814e-03  8.313e+01   3.528 0.000685 ***
## TrialNum             -1.765e-03  4.612e-04  5.220e+02  -3.827 0.000146 ***
## prevRTinv             3.195e-02  7.090e-03  3.184e+03   4.507 6.82e-06 ***
## streamT2              2.254e-02  4.184e-03  7.336e+01   5.389 8.23e-07 ***
## CondPassive          -1.017e-03  5.802e-03  1.065e+01  -0.175 0.864104    
## CondRead              1.413e-03  6.481e-03  1.161e+01   0.218 0.831148    
## streamT2:CondPassive -5.658e-03  4.980e-03  7.054e+01  -1.136 0.259813    
## streamT2:CondRead    -2.250e-03  5.552e-03  7.536e+01  -0.405 0.686474    
## ---
## Signif. codes:  0 '***' 0.001 '**' 0.01 '*' 0.05 '.' 0.1 ' ' 1
## 
## Correlation of Fixed Effects:
##             (Intr) lvlLKE l(T1RA T1.LKZ TrilNm prvRTn strmT2 CndPss CondRd sT2:CP
## lvlLetKnEsy -0.031                                                               
## lg(T1RANcT) -0.949  0.005                                                        
## T1.LetKenZ  -0.214 -0.005  0.258                                                 
## TrialNum    -0.071 -0.068 -0.009 -0.037                                          
## prevRTinv   -0.247  0.040  0.018 -0.032 -0.027                                   
## streamT2    -0.092 -0.017  0.009  0.052 -0.027 -0.046                            
## CondPassive -0.018 -0.007 -0.115 -0.047  0.014 -0.014  0.479                     
## CondRead     0.005 -0.002 -0.123 -0.006 -0.013 -0.011  0.430  0.572              
## strmT2:CndP  0.086  0.013 -0.010 -0.029 -0.015  0.026 -0.836 -0.568 -0.360       
## strmT2:CndR  0.080  0.002 -0.007  0.012  0.019 -0.004 -0.746 -0.361 -0.586  0.627
```

```
writeLines('Explained variance')
```

```
## Explained variance
```

```
r.squaredGLMM(rtLKB.2)
```

```
##             R2m       R2c
## [1,] 0.09523256 0.3187728
```

```
writeLines('Sample size by condition')
```

```
## Sample size by condition
```

```
summary(aggregate(Cond~Subj,data=rtLKB.2@frame,FUN=unique)$Cond)
```

```
##    Math Passive    Read 
##      23      49      33
```

```
writeLines('Trimmed observations:')
```

```
## Trimmed observations:
```

```
nrow(LKrtB[abs(scale(resid(rtLKB))) > 2 , ])
```

```
## [1] 166
```

```
writeLines('Trimmed in percent:')
```

```
## Trimmed in percent:
```

```
(1-nrow(LKrtB[abs(scale(resid(rtLKB))) < 2 , ]) / nrow(LKrtB))*100
```

```
## [1] 4.262969
```

```
performance::check_model(rtLKB.2)
```

```
acf(resid(rtLKB.2),main="autocorrelation of observations")
```

```
(LKrtB.2bs <- confint(rtLKB.2, method = "boot", nsim = simulations, level = 0.95, parallel = bootparallel, ncpus = bootthreads))
```

```
##                              2.5 %        97.5 %
## .sig01                3.056149e-10  0.0152181009
## .sig02                9.778588e-03  0.0171548371
## .sig03                1.617789e-01  0.7492298826
## .sig04                8.914382e-03  0.0140634091
## .sig05                9.397917e-03  0.0172127545
## .sig06                0.000000e+00  0.0087633184
## .sigma                3.518221e-02  0.0370543626
## (Intercept)           8.812632e-01  0.9921513987
## lvlLetKenEasy        -2.628612e-02 -0.0038535528
## log(T1RANcT)         -2.763350e-02 -0.0020181413
## T1.LetKenZ            2.887642e-03  0.0097897250
## TrialNum             -2.630172e-03 -0.0008140065
## prevRTinv             1.755197e-02  0.0459319950
## streamT2              1.446415e-02  0.0305706938
## CondPassive          -1.249804e-02  0.0101464817
## CondRead             -1.186895e-02  0.0135634652
## streamT2:CondPassive -1.530936e-02  0.0043331545
## streamT2:CondRead    -1.358167e-02  0.0088660316
```

```
summary(rtLKB.3)
```

```
## Linear mixed model fit by REML. t-tests use Satterthwaite's method ['lmerModLmerTest']
## Formula: RTinv ~ lvl + log(T1RANcT) + T1.LetKenZ + TrialNum + prevRTinv +      stream * Cond + (1 | itemsOnScreen) + (0 + stream | Subj) +      (1 | correctResponse) + (1 | Class)
##    Data: LKrtB.2
## 
## REML criterion at convergence: -12039.9
## 
## Scaled residuals: 
##     Min      1Q  Median      3Q     Max 
## -3.3888 -0.6410  0.0046  0.6614  3.8514 
## 
## Random effects:
##  Groups          Name        Variance  Std.Dev. Corr
##  itemsOnScreen   (Intercept) 8.017e-05 0.008954     
##  Subj            streamT1    1.852e-04 0.013610     
##                  streamT2    1.350e-04 0.011618 0.47
##  correctResponse (Intercept) 1.745e-04 0.013211     
##  Class           (Intercept) 2.191e-05 0.004681     
##  Residual                    1.306e-03 0.036136     
## Number of obs: 3279, groups:  itemsOnScreen, 741; Subj, 105; correctResponse, 30; Class, 11
## 
## Fixed effects:
##                     Estimate Std. Error         df t value Pr(>|t|)    
## (Intercept)        9.363e-01  2.849e-02  1.107e+02  32.865  < 2e-16 ***
## lvlLetKenEasy     -1.496e-02  5.610e-03  2.828e+00  -2.667 0.080853 .  
## log(T1RANcT)      -1.500e-02  6.460e-03  9.404e+01  -2.322 0.022380 *  
## T1.LetKenZ         6.401e-03  1.814e-03  8.313e+01   3.528 0.000685 ***
## TrialNum          -1.765e-03  4.612e-04  5.220e+02  -3.827 0.000146 ***
## prevRTinv          3.195e-02  7.090e-03  3.184e+03   4.507 6.82e-06 ***
## streamT2           1.689e-02  2.730e-03  6.703e+01   6.185 4.21e-08 ***
## CondMath           1.017e-03  5.802e-03  1.065e+01   0.175 0.864104    
## CondRead           2.431e-03  5.715e-03  7.387e+00   0.425 0.682739    
## streamT2:CondMath  5.658e-03  4.980e-03  7.054e+01   1.136 0.259813    
## streamT2:CondRead  3.408e-03  4.580e-03  7.498e+01   0.744 0.459138    
## ---
## Signif. codes:  0 '***' 0.001 '**' 0.01 '*' 0.05 '.' 0.1 ' ' 1
## 
## Correlation of Fixed Effects:
##             (Intr) lvlLKE l(T1RA T1.LKZ TrilNm prvRTn strmT2 CndMth CondRd sT2:CM
## lvlLetKnEsy -0.031                                                               
## lg(T1RANcT) -0.956  0.005                                                        
## T1.LetKenZ  -0.220 -0.005  0.258                                                 
## TrialNum    -0.067 -0.068 -0.009 -0.037                                          
## prevRTinv   -0.246  0.040  0.018 -0.032 -0.027                                   
## streamT2    -0.046 -0.002 -0.005  0.028 -0.068 -0.023                            
## CondMath    -0.186  0.007  0.115  0.047 -0.014  0.014  0.303                     
## CondRead    -0.051  0.005 -0.022  0.041 -0.028  0.002  0.310  0.367              
## strmT2:CndM  0.031 -0.013  0.010  0.029  0.015 -0.026 -0.543 -0.568 -0.169       
## strmT2:CndR  0.040 -0.012  0.003  0.046  0.039 -0.034 -0.590 -0.181 -0.546  0.328
```

```
(LKrtB.3bs <- confint(rtLKB.3, method = "boot", nsim = simulations, level = 0.95, parallel = bootparallel, ncpus = bootthreads))
```

```
##                          2.5 %       97.5 %
## .sig01             0.000000000  0.014776699
## .sig02             0.009875536  0.016936384
## .sig03             0.138955789  0.739840615
## .sig04             0.008887174  0.014125669
## .sig05             0.009191420  0.016975512
## .sig06             0.000000000  0.008720795
## .sigma             0.035154125  0.037148426
## (Intercept)        0.878857444  0.991797355
## lvlLetKenEasy     -0.026111591 -0.004250092
## log(T1RANcT)      -0.027156052 -0.001907220
## T1.LetKenZ         0.003288355  0.009821642
## TrialNum          -0.002668453 -0.000871387
## prevRTinv          0.017646804  0.045927019
## streamT2           0.012061735  0.022270030
## CondMath          -0.010175827  0.013044194
## CondRead          -0.008377829  0.013844676
## streamT2:CondMath -0.003909305  0.015519873
## streamT2:CondRead -0.005760380  0.012586055
```

##### 1.4.2.6.3 WLD Accuracy

```
accLDB <- glmer(correctSelections ~ Cond + targettype + T1CF_TotalZ + (1|target) + (1|Subj), family=binomial, data=LDaccB, glmerControl(optimizer="bobyqa",optCtrl=list(maxfun=50000) ))
LDaccB.2 = LDaccB[abs(scale(resid(accLDB))) < 2 , ]
accLDB.2 <- glmer(correctSelections ~ Cond + targettype + T1CF_TotalZ + (1|target) + (1|Subj), family=binomial, data=LDaccB.2, glmerControl(optimizer = "bobyqa", optCtrl=list(maxfun=50000) ))
LDaccB.2$Cond <- relevel(LDaccB.2$Cond,ref="Passive") # refit the model to get passive as baseline for plotting
accLDB.3 <- glmer(correctSelections ~ Cond + targettype + T1CF_TotalZ + (1|target) + (1|Subj), family=binomial, data=LDaccB.2, glmerControl(optimizer="bobyqa", optCtrl=list(maxfun=50000) ))
plot(effect("Cond",accLDB.3), ylab="probability of correct response", main="Written Lexical Decision Accuracy",multiline=TRUE, ci.style="bars", xlab="",lines=list(col=c("black"),lty=0))
```

```
summary(accLDB.2)
```

```
## Generalized linear mixed model fit by maximum likelihood (Laplace Approximation) ['glmerMod']
##  Family: binomial  ( logit )
## Formula: correctSelections ~ Cond + targettype + T1CF_TotalZ + (1 | target) +      (1 | Subj)
##    Data: LDaccB.2
## Control: glmerControl(optimizer = "bobyqa", optCtrl = list(maxfun = 50000))
## 
##      AIC      BIC   logLik deviance df.resid 
##   3229.8   3270.8  -1607.9   3215.8     2553 
## 
## Scaled residuals: 
##     Min      1Q  Median      3Q     Max 
## -3.0708 -0.9462  0.4744  0.8218  2.0182 
## 
## Random effects:
##  Groups Name        Variance Std.Dev.
##  Subj   (Intercept) 0.006573 0.08107 
##  target (Intercept) 0.510651 0.71460 
## Number of obs: 2560, groups:  Subj, 101; target, 30
## 
## Fixed effects:
##                   Estimate Std. Error z value Pr(>|z|)    
## (Intercept)      7.888e-01  2.173e-01   3.631 0.000283 ***
## CondPassive     -5.331e-05  1.208e-01   0.000 0.999648    
## CondRead        -1.952e-02  1.280e-01  -0.153 0.878787    
## targettypewrong -5.911e-01  2.755e-01  -2.146 0.031913 *  
## T1CF_TotalZ      2.674e-01  4.669e-02   5.726 1.03e-08 ***
## ---
## Signif. codes:  0 '***' 0.001 '**' 0.01 '*' 0.05 '.' 0.1 ' ' 1
## 
## Correlation of Fixed Effects:
##             (Intr) CndPss CondRd trgtty
## CondPassive -0.402                     
## CondRead    -0.378  0.691              
## trgttypwrng -0.641 -0.003 -0.004       
## T1CF_TotalZ  0.030  0.035  0.114 -0.015
```

```
writeLines('Explained variance')
```

```
## Explained variance
```

```
rsquared.glmm(list(accLDB.2))
```

```
##      Class   Family  Link   Marginal Conditional      AIC
## 1 glmerMod binomial logit 0.03794057    0.168644 3229.818
```

```
writeLines('Sample size by condition')
```

```
## Sample size by condition
```

```
summary(aggregate(Cond~Subj,data=accLDB.2@frame,FUN=unique)$Cond)
```

```
##    Math Passive    Read 
##      20      48      33
```

```
writeLines('Trimmed observations:')
```

```
## Trimmed observations:
```

```
nrow(LDaccB[abs(scale(resid(accLDB))) > 2 , ])
```

```
## [1] 1
```

```
writeLines('Trimmed in percent:')
```

```
## Trimmed in percent:
```

```
(1-nrow(LDaccB[abs(scale(resid(accLDB))) < 2 , ]) / nrow(LDaccB)) * 100
```

```
## [1] 0.03745318
```

```
(accLDB.2bs <- confint(accLDB.2, method = "boot", nsim = simulations, level = 0.95, parallel = bootparallel, ncpus = bootthreads))
```

```
##                      2.5 %      97.5 %
## .sig01           0.0000000  0.23260199
## .sig02           0.4758814  0.90127453
## (Intercept)      0.3776357  1.25051816
## CondPassive     -0.2537051  0.23896910
## CondRead        -0.2795643  0.23850201
## targettypewrong -1.0978086 -0.07098937
## T1CF_TotalZ      0.1759053  0.35860514
```

##### 1.4.2.6.4 WLD Response Time

```
bc <- MASS::boxcox(lm(trialRTs ~ T1.LetKenZ + T1CF_TotalZ + targettype + T1AgeF + prevRT + Cond, data=LDrtB),plotit=FALSE)
lambda <- bc$x[which.max(bc$y)]
LDrtB$RTinv = LDrtB$trialRTs^lambda
LDrtB$prevRTinv = LDrtB$prevRT^lambda
rtLDB = lmer(RTinv ~ T1.LetKenZ + T1CF_TotalZ + targettype + T1AgeF + prevRTinv + Cond + (1|target) + (1+prevRTinv|Subj),data=LDrtB)
LDrtB.2 = LDrtB[abs(scale(resid(rtLDB))) < 2 , ]
rtLDB.2 = lmer(RTinv ~ T1.LetKenZ + T1CF_TotalZ + targettype + T1AgeF + prevRTinv + Cond + (1|target) + (1+prevRTinv|Subj),data=LDrtB.2)
LDrtB.2$Cond <- relevel(LDrtB.2$Cond,ref="Passive") # refit the model to get passive as baseline for plotting
rtLDB.3 = lmer(RTinv ~ T1.LetKenZ + T1CF_TotalZ + targettype + T1AgeF + prevRTinv + Cond + (1|target) + (1+prevRTinv|Subj),data=LDrtB.2)
LKrtef = effect("Cond",rtLDB.3)
LKrtef$fit = LKrtef$fit^(1/lambda)
LKrtef$upper = LKrtef$upper^(1/lambda)
LKrtef$lower = LKrtef$lower^(1/lambda)
plot(LKrtef, ylab="Median response time (seconds)", main="Written Lexical Decision Response Times", multiline=TRUE, ci.style="bars", xlab="", lines=list(col=c("black"), lty=0))
```

```
# compute effect size for age
summary(rtLDB.2)
```

```
## Linear mixed model fit by REML. t-tests use Satterthwaite's method ['lmerModLmerTest']
## Formula: RTinv ~ T1.LetKenZ + T1CF_TotalZ + targettype + T1AgeF + prevRTinv +      Cond + (1 | target) + (1 + prevRTinv | Subj)
##    Data: LDrtB.2
## 
## REML criterion at convergence: 747.3
## 
## Scaled residuals: 
##     Min      1Q  Median      3Q     Max 
## -3.2168 -0.6345 -0.0459  0.5976  3.3212 
## 
## Random effects:
##  Groups   Name        Variance Std.Dev. Corr 
##  Subj     (Intercept) 0.066306 0.25750       
##           prevRTinv   0.004995 0.07068  -0.81
##  target   (Intercept) 0.008723 0.09339       
##  Residual             0.084286 0.29032       
## Number of obs: 1313, groups:  Subj, 100; target, 29
## 
## Fixed effects:
##                 Estimate Std. Error       df t value Pr(>|t|)    
## (Intercept)      2.69467    0.39484 87.54257   6.825  1.1e-09 ***
## T1.LetKenZ      -0.04831    0.03261 79.18842  -1.481 0.142479    
## T1CF_TotalZ      0.06623    0.02360 84.01687   2.807 0.006221 ** 
## targettypewrong  0.12262    0.03882 28.04879   3.159 0.003772 ** 
## T1AgeF          -0.19504    0.06153 84.83820  -3.170 0.002123 ** 
## prevRTinv        0.06794    0.01786 73.66440   3.805 0.000291 ***
## CondPassive      0.05352    0.05281 86.99414   1.013 0.313636    
## CondRead         0.06282    0.05615 86.58906   1.119 0.266329    
## ---
## Signif. codes:  0 '***' 0.001 '**' 0.01 '*' 0.05 '.' 0.1 ' ' 1
## 
## Correlation of Fixed Effects:
##             (Intr) T1.LKZ T1CF_T trgtty T1AgeF prvRTn CndPss
## T1.LetKenZ   0.306                                          
## T1CF_TotalZ -0.007 -0.483                                   
## trgttypwrng -0.052 -0.010 -0.011                            
## T1AgeF      -0.988 -0.284 -0.003  0.006                     
## prevRTinv   -0.124  0.035 -0.057 -0.012  0.049              
## CondPassive -0.170 -0.108  0.086 -0.002  0.077 -0.027       
## CondRead     0.023  0.060  0.104 -0.009 -0.105 -0.075  0.654
```

```
writeLines('Explained variance')
```

```
## Explained variance
```

```
rsquared.glmm(list(rtLDB.2))
```

```
##             Class   Family     Link  Marginal Conditional      AIC
## 1 lmerModLmerTest gaussian identity 0.1057042   0.4023973 733.3377
```

```
writeLines('Sample size by condition')
```

```
## Sample size by condition
```

```
summary(aggregate(Cond~Subj,data=rtLDB.2@frame,FUN=unique))
```

```
##       Subj         Cond   
##  7      : 1   Math   :20  
##  12     : 1   Passive:47  
##  57     : 1   Read   :33  
##  60     : 1               
##  66     : 1               
##  69     : 1               
##  (Other):94
```

```
writeLines('Trimmed observations:')
```

```
## Trimmed observations:
```

```
nrow(LDrtB[abs(scale(resid(rtLDB))) > 2 , ])
```

```
## [1] 68
```

```
writeLines('Trimmed in percent:')
```

```
## Trimmed in percent:
```

```
(1-nrow(LDrtB[abs(scale(resid(rtLDB))) < 2 , ]) / nrow(LDrtB)) * 100
```

```
## [1] 4.579125
```

```
performance::check_model(rtLDB)
```

```
acf(resid(rtLDB.2),main="autocorrelation of observations")
```

```
(rtLDB.2bs <- confint(rtLDB.2, method = "boot", nsim = simulations, level = 0.95, parallel = bootparallel, ncpus = bootthreads))
```

```
##                       2.5 %      97.5 %
## .sig01           0.18744544  0.32846060
## .sig02          -1.00000000 -0.54088864
## .sig03           0.01400177  0.12030901
## .sig04           0.05932034  0.12605630
## .sigma           0.27748255  0.30081130
## (Intercept)      1.88026530  3.41832904
## T1.LetKenZ      -0.11371788  0.01435871
## T1CF_TotalZ      0.02021556  0.11698591
## targettypewrong  0.04355840  0.20156252
## T1AgeF          -0.30489316 -0.06822864
## prevRTinv        0.03113782  0.10195543
## CondPassive     -0.04830069  0.15973385
## CondRead        -0.05305058  0.17761467
```

### 1.4.3 ANALYSIS 2 - Dutch Sample

This is the analysis for the Dutch sample (N = 86) which only features an active (math) control condition.

#### 1.4.3.1 Descriptives

Here, we show results of Chi-squared/Fisher’s exact test for Gender, Language, Handedness and Familial risk in Dutch sample There are more multilingual children in the Dutch math cohort.

##### 1.4.3.1.1 Chi-square test Gender x Condition

```
chisq.test(matrix(c(23,25,23,15),ncol=2,dimnames=list(c("female","male"), c("Math","Read"))))
```

```
## 
##  Pearson's Chi-squared test with Yates' continuity correction
## 
## data:  matrix(c(23, 25, 23, 15), ncol = 2, dimnames = list(c("female",     "male"), c("Math", "Read")))
## X-squared = 0.89606, df = 1, p-value = 0.3438
```

##### 1.4.3.1.2 Fisher’s exact test Multilingualism x Condition

```
fisher.test(matrix(c(12,36,2,36),ncol=2,dimnames=list(c("multilingual","monolingual"), c("Math","Read"))))
```

```
## 
##  Fisher's Exact Test for Count Data
## 
## data:  matrix(c(12, 36, 2, 36), ncol = 2, dimnames = list(c("multilingual", "monolingual"), c("Math", "Read")))
## p-value = 0.01783
## alternative hypothesis: true odds ratio is not equal to 1
## 95 percent confidence interval:
##   1.18392 57.86893
## sample estimates:
## odds ratio 
##   5.891757
```

##### 1.4.3.1.3 Fisher’s exact test Handedness x Condition

```
fisher.test(matrix(c(3,45,6,32),ncol=2,dimnames=list(c("lefthander","righthander"), c("Math","Read"))))
```

```
## 
##  Fisher's Exact Test for Count Data
## 
## data:  matrix(c(3, 45, 6, 32), ncol = 2, dimnames = list(c("lefthander", "righthander"), c("Math", "Read")))
## p-value = 0.1751
## alternative hypothesis: true odds ratio is not equal to 1
## 95 percent confidence interval:
##  0.05421993 1.83485472
## sample estimates:
## odds ratio 
##  0.3598873
```

##### 1.4.3.1.4 Chi-square test Familial Risk x Condition

```
chisq.test(matrix(c(11,37,6,32),ncol=2,dimnames=list(c("namilial risk","no familial risk"), c("Math","Read"))))
```

```
## 
##  Pearson's Chi-squared test with Yates' continuity correction
## 
## data:  matrix(c(11, 37, 6, 32), ncol = 2, dimnames = list(c("namilial risk",     "no familial risk"), c("Math", "Read")))
## X-squared = 0.30424, df = 1, p-value = 0.5812
```

---

ANOVAs comparing the remaining T1 measures revealed that the reading group knew marginally less letters at T1.

```
summary(aov(T1AgeF~Cond,data=datNL))
```

```
##             Df Sum Sq Mean Sq F value Pr(>F)
## Cond         1  0.079 0.07905   0.825  0.366
## Residuals   84  8.044 0.09576
```

```
summary(aov(T1.LetKen~Cond,data=datNL))
```

```
##             Df Sum Sq Mean Sq F value Pr(>F)  
## Cond         1  108.6  108.63   3.574 0.0621 .
## Residuals   84 2552.9   30.39                 
## ---
## Signif. codes:  0 '***' 0.001 '**' 0.01 '*' 0.05 '.' 0.1 ' ' 1
```

```
TukeyHSD(aov(T1.LetKen~Cond,data=datNL))
```

```
##   Tukey multiple comparisons of means
##     95% family-wise confidence level
## 
## Fit: aov(formula = T1.LetKen ~ Cond, data = datNL)
## 
## $Cond
##                diff       lwr       upr     p adj
## Read-Math -2.263158 -4.643618 0.1173023 0.0621234
```

```
summary(aov(T1CF_TotalPc~Cond,data=datNL))
```

```
##             Df Sum Sq Mean Sq F value Pr(>F)
## Cond         1    311   311.1   0.729  0.396
## Residuals   84  35858   426.9
```

```
summary(aov(T1PF_TotalPc~Cond,data=datNL))
```

```
##             Df Sum Sq Mean Sq F value Pr(>F)
## Cond         1   1243  1243.3   1.994  0.162
## Residuals   84  52368   623.4
```

```
summary(aov(T1RANoT~Cond,data=datNL))
```

```
##             Df Sum Sq Mean Sq F value Pr(>F)
## Cond         1    483   483.4    2.51  0.117
## Residuals   84  16176   192.6
```

```
summary(aov(T1RANcT~Cond,data=datNL))
```

```
##             Df Sum Sq Mean Sq F value Pr(>F)
## Cond         1     19   19.43   0.112  0.738
## Residuals   84  14549  173.20
```

```
summary(aov(SON_1st~Cond,data=datNL))
```

```
##             Df Sum Sq Mean Sq F value Pr(>F)
## Cond         1   0.14  0.1434   0.142  0.707
## Residuals   84  84.86  1.0102
```

---

Summaries of the Dutch data with number of observations, means, range and standard deviation per variable and gaming condition.

```
tab <- tableby(Gender ~ EMT + Cond + T1AgeF + HandScore + FamRisk + Lang + SON_1st + hoursPlayed + levelsPlayed + sessionsPlayed + MaxLevel + itemsSeen + responsesGiven + T1CF_Total + T1PF_Total + T1.LetKen + T1RANcT + T1RANoT, data = datNL, numeric.stats=c("meansd","medianrange"))
summary(tab, text=TRUE, digits=2, test=F)
```

```
## 
## 
## |                  |        female (N=46)        |         male (N=40)         |        Total (N=86)         |
## |:-----------------|:---------------------------:|:---------------------------:|:---------------------------:|
## |EMT               |                             |                             |                             |
## |-  Mean (SD)      |         0.17 (1.02)         |        -0.19 (0.96)         |         0.00 (1.00)         |
## |-  Median (Range) |     -0.20 (-1.23, 2.07)     |     -0.43 (-1.63, 2.07)     |     -0.34 (-1.63, 2.07)     |
## |Cond              |                             |                             |                             |
## |-  Math           |         23 (50.0%)          |         25 (62.5%)          |         48 (55.8%)          |
## |-  Read           |         23 (50.0%)          |         15 (37.5%)          |         38 (44.2%)          |
## |T1AgeF            |                             |                             |                             |
## |-  Mean (SD)      |         6.21 (0.33)         |         6.24 (0.29)         |         6.23 (0.31)         |
## |-  Median (Range) |      6.24 (5.46, 6.72)      |      6.28 (5.55, 6.83)      |      6.26 (5.46, 6.83)      |
## |HandScore         |                             |                             |                             |
## |-  Mean (SD)      |        -0.04 (1.08)         |         0.04 (0.92)         |        -0.00 (1.00)         |
## |-  Median (Range) |     0.32 (-3.14, 0.48)      |     0.40 (-3.14, 0.48)      |     0.32 (-3.14, 0.48)      |
## |FamRisk           |                             |                             |                             |
## |-  no             |         37 (80.4%)          |         32 (80.0%)          |         69 (80.2%)          |
## |-  yes            |          9 (19.6%)          |          8 (20.0%)          |         17 (19.8%)          |
## |Lang              |                             |                             |                             |
## |-  mono           |         37 (80.4%)          |         35 (87.5%)          |         72 (83.7%)          |
## |-  multi          |          9 (19.6%)          |          5 (12.5%)          |         14 (16.3%)          |
## |SON_1st           |                             |                             |                             |
## |-  Mean (SD)      |        -0.05 (1.04)         |         0.06 (0.96)         |         0.00 (1.00)         |
## |-  Median (Range) |     0.05 (-2.15, 4.87)      |     0.05 (-2.15, 2.24)      |     0.05 (-2.15, 4.87)      |
## |hoursPlayed       |                             |                             |                             |
## |-  Mean (SD)      |         2.94 (0.66)         |         2.94 (0.67)         |         2.94 (0.66)         |
## |-  Median (Range) |      2.94 (1.68, 4.95)      |      2.78 (1.98, 4.94)      |      2.93 (1.68, 4.95)      |
## |levelsPlayed      |                             |                             |                             |
## |-  Mean (SD)      |       281.28 (93.38)        |       287.70 (70.10)        |       284.27 (82.96)        |
## |-  Median (Range) |   268.50 (101.00, 607.00)   |   295.00 (134.00, 432.00)   |   280.00 (101.00, 607.00)   |
## |sessionsPlayed    |                             |                             |                             |
## |-  Mean (SD)      |        26.67 (2.98)         |        26.70 (3.54)         |        26.69 (3.23)         |
## |-  Median (Range) |    27.00 (20.00, 32.00)     |    26.00 (20.00, 34.00)     |    27.00 (20.00, 34.00)     |
## |MaxLevel          |                             |                             |                             |
## |-  Mean (SD)      |       144.67 (44.97)        |       147.07 (45.61)        |       145.79 (45.02)        |
## |-  Median (Range) |   136.00 (71.00, 265.00)    |   136.00 (62.00, 265.00)    |   136.00 (62.00, 265.00)    |
## |itemsSeen         |                             |                             |                             |
## |-  Mean (SD)      |      9216.80 (4254.36)      |      8874.80 (4140.55)      |      9057.73 (4180.63)      |
## |-  Median (Range) | 8229.00 (3304.00, 18509.00) | 7696.00 (3814.00, 20113.00) | 7844.00 (3304.00, 20113.00) |
## |responsesGiven    |                             |                             |                             |
## |-  Mean (SD)      |      2729.26 (1063.78)      |      2795.25 (815.21)       |      2759.95 (951.37)       |
## |-  Median (Range) |  2602.00 (999.00, 6984.00)  | 2928.00 (1295.00, 4522.00)  |  2686.00 (999.00, 6984.00)  |
## |T1CF_Total        |                             |                             |                             |
## |-  Mean (SD)      |        33.07 (6.24)         |        30.73 (6.97)         |        31.98 (6.66)         |
## |-  Median (Range) |    34.00 (17.00, 44.00)     |     31.50 (8.00, 40.00)     |     32.00 (8.00, 44.00)     |
## |T1PF_Total        |                             |                             |                             |
## |-  Mean (SD)      |        34.30 (4.06)         |        33.38 (3.94)         |        33.87 (4.01)         |
## |-  Median (Range) |    35.50 (23.00, 40.00)     |    33.50 (21.00, 40.00)     |    35.00 (21.00, 40.00)     |
## |T1.LetKen         |                             |                             |                             |
## |-  Mean (SD)      |        25.20 (4.18)         |        21.55 (6.38)         |        23.50 (5.60)         |
## |-  Median (Range) |    27.00 (12.00, 30.00)     |     23.00 (2.00, 30.00)     |     25.00 (2.00, 30.00)     |
## |T1RANcT           |                             |                             |                             |
## |-  Mean (SD)      |        57.02 (11.41)        |        60.98 (14.66)        |        58.86 (13.09)        |
## |-  Median (Range) |    57.00 (39.00, 82.00)     |    58.00 (43.00, 102.00)    |    57.50 (39.00, 102.00)    |
## |T1RANoT           |                             |                             |                             |
## |-  Mean (SD)      |        65.91 (13.85)        |        71.15 (13.81)        |        68.35 (14.00)        |
## |-  Median (Range) |    67.00 (43.00, 126.00)    |    66.50 (40.00, 97.00)     |    67.00 (40.00, 126.00)    |
```

```
tab <- tableby(FamRisk ~ EMT + Cond + T1AgeF + HandScore + Gender + Lang + SON_1st + hoursPlayed + levelsPlayed + sessionsPlayed + MaxLevel + itemsSeen + responsesGiven + T1CF_Total + T1PF_Total + T1.LetKen + T1RANcT + T1RANoT, data = datNL, numeric.stats=c("meansd","medianrange"))
summary(tab, text=TRUE, digits=2, test=F)
```

```
## 
## 
## |                  |          no (N=69)          |         yes (N=17)          |        Total (N=86)         |
## |:-----------------|:---------------------------:|:---------------------------:|:---------------------------:|
## |EMT               |                             |                             |                             |
## |-  Mean (SD)      |         0.01 (0.97)         |        -0.06 (1.15)         |         0.00 (1.00)         |
## |-  Median (Range) |     -0.34 (-1.23, 2.07)     |     -0.38 (-1.63, 2.07)     |     -0.34 (-1.63, 2.07)     |
## |Cond              |                             |                             |                             |
## |-  Math           |         37 (53.6%)          |         11 (64.7%)          |         48 (55.8%)          |
## |-  Read           |         32 (46.4%)          |          6 (35.3%)          |         38 (44.2%)          |
## |T1AgeF            |                             |                             |                             |
## |-  Mean (SD)      |         6.23 (0.33)         |         6.20 (0.23)         |         6.23 (0.31)         |
## |-  Median (Range) |      6.27 (5.46, 6.83)      |      6.22 (5.82, 6.56)      |      6.26 (5.46, 6.83)      |
## |HandScore         |                             |                             |                             |
## |-  Mean (SD)      |        -0.01 (1.04)         |         0.05 (0.85)         |        -0.00 (1.00)         |
## |-  Median (Range) |     0.48 (-3.14, 0.48)      |     0.32 (-3.14, 0.48)      |     0.32 (-3.14, 0.48)      |
## |Gender            |                             |                             |                             |
## |-  female         |         37 (53.6%)          |          9 (52.9%)          |         46 (53.5%)          |
## |-  male           |         32 (46.4%)          |          8 (47.1%)          |         40 (46.5%)          |
## |Lang              |                             |                             |                             |
## |-  mono           |         58 (84.1%)          |         14 (82.4%)          |         72 (83.7%)          |
## |-  multi          |         11 (15.9%)          |          3 (17.6%)          |         14 (16.3%)          |
## |SON_1st           |                             |                             |                             |
## |-  Mean (SD)      |        -0.04 (1.05)         |         0.15 (0.77)         |         0.00 (1.00)         |
## |-  Median (Range) |     0.05 (-2.15, 4.87)      |     0.05 (-0.83, 2.24)      |     0.05 (-2.15, 4.87)      |
## |hoursPlayed       |                             |                             |                             |
## |-  Mean (SD)      |         2.92 (0.67)         |         3.02 (0.66)         |         2.94 (0.66)         |
## |-  Median (Range) |      2.94 (1.68, 4.95)      |      2.82 (2.23, 4.94)      |      2.93 (1.68, 4.95)      |
## |levelsPlayed      |                             |                             |                             |
## |-  Mean (SD)      |       282.29 (85.66)        |       292.29 (72.74)        |       284.27 (82.96)        |
## |-  Median (Range) |   275.00 (101.00, 607.00)   |   316.00 (152.00, 414.00)   |   280.00 (101.00, 607.00)   |
## |sessionsPlayed    |                             |                             |                             |
## |-  Mean (SD)      |        26.91 (3.36)         |        25.76 (2.54)         |        26.69 (3.23)         |
## |-  Median (Range) |    27.00 (20.00, 34.00)     |    25.00 (23.00, 33.00)     |    27.00 (20.00, 34.00)     |
## |MaxLevel          |                             |                             |                             |
## |-  Mean (SD)      |       147.28 (45.24)        |       139.76 (44.97)        |       145.79 (45.02)        |
## |-  Median (Range) |   136.00 (71.00, 265.00)    |   136.00 (62.00, 241.00)    |   136.00 (62.00, 265.00)    |
## |itemsSeen         |                             |                             |                             |
## |-  Mean (SD)      |      9172.83 (4253.74)      |      8590.59 (3957.31)      |      9057.73 (4180.63)      |
## |-  Median (Range) | 8025.00 (3304.00, 20113.00) | 7664.00 (5069.00, 18594.00) | 7844.00 (3304.00, 20113.00) |
## |responsesGiven    |                             |                             |                             |
## |-  Mean (SD)      |      2763.04 (1006.70)      |      2747.41 (707.86)       |      2759.95 (951.37)       |
## |-  Median (Range) |  2842.00 (999.00, 6984.00)  | 2485.00 (1882.00, 4212.00)  |  2686.00 (999.00, 6984.00)  |
## |T1CF_Total        |                             |                             |                             |
## |-  Mean (SD)      |        32.64 (5.98)         |        29.29 (8.62)         |        31.98 (6.66)         |
## |-  Median (Range) |    33.00 (19.00, 44.00)     |     28.00 (8.00, 41.00)     |     32.00 (8.00, 44.00)     |
## |T1PF_Total        |                             |                             |                             |
## |-  Mean (SD)      |        34.04 (3.95)         |        33.18 (4.29)         |        33.87 (4.01)         |
## |-  Median (Range) |    35.00 (21.00, 40.00)     |    32.00 (23.00, 40.00)     |    35.00 (21.00, 40.00)     |
## |T1.LetKen         |                             |                             |                             |
## |-  Mean (SD)      |        23.38 (5.78)         |        24.00 (4.92)         |        23.50 (5.60)         |
## |-  Median (Range) |     25.00 (2.00, 30.00)     |    25.00 (15.00, 30.00)     |     25.00 (2.00, 30.00)     |
## |T1RANcT           |                             |                             |                             |
## |-  Mean (SD)      |        56.48 (12.19)        |        68.53 (12.45)        |        58.86 (13.09)        |
## |-  Median (Range) |    56.00 (39.00, 102.00)    |    68.00 (45.00, 96.00)     |    57.50 (39.00, 102.00)    |
## |T1RANoT           |                             |                             |                             |
## |-  Mean (SD)      |        66.33 (12.59)        |        76.53 (16.70)        |        68.35 (14.00)        |
## |-  Median (Range) |    66.00 (40.00, 94.00)     |    72.00 (56.00, 126.00)    |    67.00 (40.00, 126.00)    |
```

```
tab <- tableby(Cond ~ EMT + FamRisk + T1AgeF + HandScore + Gender + Lang + SON_1st + hoursPlayed + levelsPlayed + sessionsPlayed + MaxLevel + itemsSeen + responsesGiven + T1CF_Total + T1PF_Total + T1.LetKen + T1RANcT + T1RANoT, data = datNL, numeric.stats=c("meansd","medianrange"))
summary(tab, text=TRUE, digits=2, test=F)
```

```
## 
## 
## |                  |         Math (N=48)         |         Read (N=38)          |        Total (N=86)         |
## |:-----------------|:---------------------------:|:----------------------------:|:---------------------------:|
## |EMT               |                             |                              |                             |
## |-  Mean (SD)      |         0.18 (1.04)         |         -0.22 (0.92)         |         0.00 (1.00)         |
## |-  Median (Range) |     -0.20 (-1.63, 2.07)     |     -0.47 (-1.23, 2.07)      |     -0.34 (-1.63, 2.07)     |
## |FamRisk           |                             |                              |                             |
## |-  no             |         37 (77.1%)          |          32 (84.2%)          |         69 (80.2%)          |
## |-  yes            |         11 (22.9%)          |          6 (15.8%)           |         17 (19.8%)          |
## |T1AgeF            |                             |                              |                             |
## |-  Mean (SD)      |         6.25 (0.32)         |         6.19 (0.29)          |         6.23 (0.31)         |
## |-  Median (Range) |      6.32 (5.55, 6.83)      |      6.20 (5.46, 6.69)       |      6.26 (5.46, 6.83)      |
## |HandScore         |                             |                              |                             |
## |-  Mean (SD)      |         0.10 (0.85)         |         -0.12 (1.17)         |        -0.00 (1.00)         |
## |-  Median (Range) |     0.32 (-3.14, 0.48)      |      0.40 (-3.14, 0.48)      |     0.32 (-3.14, 0.48)      |
## |Gender            |                             |                              |                             |
## |-  female         |         23 (47.9%)          |          23 (60.5%)          |         46 (53.5%)          |
## |-  male           |         25 (52.1%)          |          15 (39.5%)          |         40 (46.5%)          |
## |Lang              |                             |                              |                             |
## |-  mono           |         36 (75.0%)          |          36 (94.7%)          |         72 (83.7%)          |
## |-  multi          |         12 (25.0%)          |           2 (5.3%)           |         14 (16.3%)          |
## |SON_1st           |                             |                              |                             |
## |-  Mean (SD)      |        -0.04 (0.87)         |         0.05 (1.15)          |         0.00 (1.00)         |
## |-  Median (Range) |     0.05 (-2.15, 2.24)      |      0.05 (-2.15, 4.87)      |     0.05 (-2.15, 4.87)      |
## |hoursPlayed       |                             |                              |                             |
## |-  Mean (SD)      |         2.70 (0.43)         |         3.26 (0.77)          |         2.94 (0.66)         |
## |-  Median (Range) |      2.72 (1.96, 3.80)      |      3.24 (1.68, 4.95)       |      2.93 (1.68, 4.95)      |
## |levelsPlayed      |                             |                              |                             |
## |-  Mean (SD)      |       318.25 (77.64)        |        241.34 (69.02)        |       284.27 (82.96)        |
## |-  Median (Range) |   317.50 (191.00, 607.00)   |   237.50 (101.00, 377.00)    |   280.00 (101.00, 607.00)   |
## |sessionsPlayed    |                             |                              |                             |
## |-  Mean (SD)      |        25.48 (2.02)         |         28.21 (3.81)         |        26.69 (3.23)         |
## |-  Median (Range) |    25.00 (20.00, 30.00)     |     29.00 (20.00, 34.00)     |    27.00 (20.00, 34.00)     |
## |MaxLevel          |                             |                              |                             |
## |-  Mean (SD)      |       135.94 (23.95)        |        158.24 (60.35)        |       145.79 (45.02)        |
## |-  Median (Range) |   135.50 (62.00, 178.00)    |    164.50 (71.00, 265.00)    |   136.00 (62.00, 265.00)    |
## |itemsSeen         |                             |                              |                             |
## |-  Mean (SD)      |      6652.12 (2116.28)      |      12096.39 (4179.95)      |      9057.73 (4180.63)      |
## |-  Median (Range) | 6722.50 (3304.00, 15832.00) | 11825.50 (4191.00, 20113.00) | 7844.00 (3304.00, 20113.00) |
## |responsesGiven    |                             |                              |                             |
## |-  Mean (SD)      |      2753.85 (1001.08)      |       2767.66 (897.87)       |      2759.95 (951.37)       |
## |-  Median (Range) | 2668.50 (1197.00, 6984.00)  |  2767.00 (999.00, 4522.00)   |  2686.00 (999.00, 6984.00)  |
## |T1CF_Total        |                             |                              |                             |
## |-  Mean (SD)      |        31.54 (6.71)         |         32.53 (6.64)         |        31.98 (6.66)         |
## |-  Median (Range) |     32.00 (8.00, 41.00)     |     32.50 (19.00, 44.00)     |     32.00 (8.00, 44.00)     |
## |T1PF_Total        |                             |                              |                             |
## |-  Mean (SD)      |        33.17 (4.26)         |         34.76 (3.51)         |        33.87 (4.01)         |
## |-  Median (Range) |    33.50 (21.00, 40.00)     |     35.00 (27.00, 40.00)     |    35.00 (21.00, 40.00)     |
## |T1.LetKen         |                             |                              |                             |
## |-  Mean (SD)      |        24.50 (4.74)         |         22.24 (6.36)         |        23.50 (5.60)         |
## |-  Median (Range) |    26.00 (13.00, 30.00)     |     24.00 (2.00, 30.00)      |     25.00 (2.00, 30.00)     |
## |T1RANcT           |                             |                              |                             |
## |-  Mean (SD)      |        58.44 (11.80)        |        59.39 (14.71)         |        58.86 (13.09)        |
## |-  Median (Range) |    57.50 (41.00, 96.00)     |    57.50 (39.00, 102.00)     |    57.50 (39.00, 102.00)    |
## |T1RANoT           |                             |                              |                             |
## |-  Mean (SD)      |        70.46 (15.11)        |        65.68 (12.13)         |        68.35 (14.00)        |
## |-  Median (Range) |    68.00 (40.00, 126.00)    |     65.50 (43.00, 90.00)     |    67.00 (40.00, 126.00)    |
```

#### 1.4.3.2 Correlations

Here, we show the correlation matrices of the main outcome variables and covariates at T1 and T2: EMT (one minute reading), CF\_Total (CELF PA raw scores), PF\_Total (PROEF PA raw scores), RAN (rapid automatized naming) of objects (o) or colors (c), as well as LK (in-game letter knowledge) and WLD (written lexical decision). An asterisk indicates significance at p < .05

```
corNL <- subset(datNL,select=c(EMT, T1CF_Total,T2CF_Total, T1PF_Total,T2PF_Total, T1RANcT, T2RANcT, T1RANoT, T2RANoT, T1.LetKen, T2.LetKen, T2.LexDec))
names(corNL) <- c("T2EMT","T1CF","T2CF","T1PF","T2PF","T1RANc","T2RANc","T1RANo","T2RANo","T1LK","T2LK","T2WLD")
corNLM <- subset(datNL,Cond=="Math",select=c(EMT, T1CF_Total,T2CF_Total, T1PF_Total,T2PF_Total, T1RANcT, T2RANcT, T1RANoT, T2RANoT, T1.LetKen, T2.LetKen, T2.LexDec))
names(corNLM) <- c("T2EMT","T1CF","T2CF","T1PF","T2PF","T1RANc","T2RANc","T1RANo","T2RANo","T1LK","T2LK","T2WLD")
corNLR <- subset(datNL,Cond=="Read",select=c(EMT, T1CF_Total,T2CF_Total, T1PF_Total,T2PF_Total, T1RANcT, T2RANcT, T1RANoT, T2RANoT, T1.LetKen, T2.LetKen, T2.LexDec))
names(corNLR) <- c("T2EMT","T1CF","T2CF","T1PF","T2PF","T1RANc","T2RANc","T1RANo","T2RANo","T1LK","T2LK","T2WLD")
```

##### 1.4.3.2.1 Entire Dutch Sample

```
##         T2EMT   T1CF   T2CF   T1PF   T2PF T1RANc T2RANc T1RANo T2RANo   T1LK   T2LK
## T2EMT                                                                              
## T1CF    0.38*                                                                      
## T2CF    0.34*  0.55*                                                               
## T1PF    0.31*  0.47*  0.17                                                         
## T2PF    0.37*  0.57*  0.48*  0.43*                                                 
## T1RANc -0.32* -0.41* -0.33* -0.13  -0.26*                                          
## T2RANc -0.33* -0.40* -0.39* -0.17  -0.20   0.73*                                   
## T1RANo -0.30* -0.32* -0.14  -0.20  -0.11   0.59*  0.44*                            
## T2RANo -0.34* -0.27* -0.19  -0.22* -0.21   0.49*  0.49*  0.66*                     
## T1LK    0.45*  0.47*  0.45*  0.19   0.36* -0.40* -0.46* -0.34* -0.33*              
## T2LK    0.32*  0.19   0.25*  0.17   0.26* -0.26* -0.25* -0.28* -0.18   0.37*       
## T2WLD   0.41*  0.41*  0.29*  0.18   0.34* -0.20  -0.17  -0.17  -0.13   0.41*  0.34*
```

---

##### 1.4.3.2.2 Dutch Math Group

```
##         T2EMT   T1CF   T2CF   T1PF   T2PF T1RANc T2RANc T1RANo T2RANo   T1LK   T2LK
## T2EMT                                                                              
## T1CF    0.35*                                                                      
## T2CF    0.16   0.70*                                                               
## T1PF    0.35*  0.49*  0.23                                                         
## T2PF    0.34*  0.68*  0.58*  0.58*                                                 
## T1RANc -0.34* -0.43* -0.21  -0.17  -0.18                                           
## T2RANc -0.30* -0.36* -0.23  -0.09  -0.09   0.69*                                   
## T1RANo -0.35* -0.33* -0.09  -0.14  -0.14   0.55*  0.36*                            
## T2RANo -0.36* -0.32* -0.12  -0.21  -0.23   0.51*  0.43*  0.67*                     
## T1LK    0.35*  0.51*  0.51*  0.16   0.40* -0.37* -0.26  -0.36* -0.27               
## T2LK    0.15   0.27   0.15   0.29*  0.16  -0.03   0.01  -0.27  -0.12   0.31*       
## T2WLD   0.34*  0.47*  0.36*  0.26   0.38* -0.31* -0.18  -0.25  -0.13   0.49*  0.17
```

---

##### 1.4.3.2.3 Dutch Read Group

```
##         T2EMT   T1CF   T2CF   T1PF   T2PF T1RANc T2RANc T1RANo T2RANo   T1LK   T2LK
## T2EMT                                                                              
## T1CF    0.47*                                                                      
## T2CF    0.61*  0.37*                                                               
## T1PF    0.41*  0.44*  0.10                                                         
## T2PF    0.44*  0.47*  0.38*  0.28                                                  
## T1RANc -0.32  -0.40* -0.44* -0.12  -0.33*                                          
## T2RANc -0.37* -0.46* -0.55* -0.30  -0.30   0.77*                                   
## T1RANo -0.33* -0.28  -0.23  -0.24  -0.07   0.71*  0.60*                            
## T2RANo -0.35* -0.18  -0.29  -0.20  -0.19   0.49*  0.59*  0.65*                     
## T1LK    0.53*  0.50*  0.42*  0.36*  0.35* -0.42* -0.64* -0.45* -0.48*              
## T2LK    0.43*  0.17   0.35   0.16   0.34  -0.42* -0.46* -0.56* -0.48*  0.32        
## T2WLD   0.51*  0.34   0.17   0.07   0.29  -0.06  -0.12  -0.11  -0.22   0.27   0.47*
```

#### 1.4.3.3 Reading Fluency

Single word reading fluency at T2 as measures by two custom lists with a time limit of one minute each.

```
## boundary (singular) fit: see ?isSingular
```

```
## Linear mixed model fit by REML. t-tests use Satterthwaite's method ['lmerModLmerTest']
## Formula: EMT ~ HandScore + T1PF_TotalZ + T1.LetKenZ + Cond + (1 | Class)
##    Data: datNLemt.2
## 
## REML criterion at convergence: 171.9
## 
## Scaled residuals: 
##     Min      1Q  Median      3Q     Max 
## -1.9108 -0.6936 -0.1244  0.6708  2.6002 
## 
## Random effects:
##  Groups   Name        Variance Std.Dev.
##  Class    (Intercept) 0.01109  0.1053  
##  Residual             0.46464  0.6816  
## Number of obs: 78, groups:  Class, 4
## 
## Fixed effects:
##             Estimate Std. Error       df t value Pr(>|t|)    
## (Intercept)  0.06727    0.13056  1.39930   0.515 0.675933    
## HandScore   -0.32140    0.08810 71.68593  -3.648 0.000497 ***
## T1PF_TotalZ  0.27146    0.08424 72.69679   3.222 0.001904 ** 
## T1.LetKenZ   0.24912    0.08090 71.59112   3.079 0.002940 ** 
## CondRead    -0.30637    0.19348  1.59783  -1.583 0.283579    
## ---
## Signif. codes:  0 '***' 0.001 '**' 0.01 '*' 0.05 '.' 0.1 ' ' 1
## 
## Correlation of Fixed Effects:
##             (Intr) HndScr T1PF_T T1.LKZ
## HandScore   -0.091                     
## T1PF_TotalZ  0.072  0.245              
## T1.LetKenZ  -0.089  0.062 -0.248       
## CondRead    -0.691  0.079 -0.128  0.183
```

```
## Explained variance
```

```
## boundary (singular) fit: see ?isSingular
```

```
##             Class   Family     Link  Marginal Conditional      AIC
## 1 lmerModLmerTest gaussian identity 0.4138466   0.4275107 171.2494
```

```
## Sample size by condition
```

```
## Math Read 
##   42   36
```

```
## Trimmed observations:
```

```
## [1] 6
```

```
## Trimmed in percent:
```

```
## [1] 7.142857
```

```
## `geom_smooth()` using formula 'y ~ x'
```

```
## `geom_smooth()` using formula 'y ~ x'
## `geom_smooth()` using formula 'y ~ x'
```

```
## `stat_bin()` using `bins = 30`. Pick better value with `binwidth`.
```

```
## `geom_smooth()` using formula 'y ~ x'
```

```
## Computing bootstrap confidence intervals ...
```

```
## 
## 483 message(s): boundary (singular) fit: see ?isSingular
```

```
##                   2.5 %      97.5 %
## .sig01       0.00000000  0.34621077
## .sigma       0.57004910  0.79366215
## (Intercept) -0.18228291  0.32647355
## HandScore   -0.49755926 -0.15494947
## T1PF_TotalZ  0.11243107  0.44528462
## T1.LetKenZ   0.08747288  0.40652492
## CondRead    -0.66142906  0.06869993
```

#### 1.4.3.4 PA

Phonological awareness at T2 as measured by the CELF-IV-NL and the PROEF.

##### 1.4.3.4.1 CELF-IV-NL Phonological Awareness

```
## 
## Call:
## lm(formula = T2CF_TotalZ ~ T1.LetKenZ + T1CF_TotalZ + Cond, data = datNLcf.2)
## 
## Residuals:
##     Min      1Q  Median      3Q     Max 
## -1.5770 -0.5057  0.1039  0.4939  1.2189 
## 
## Coefficients:
##             Estimate Std. Error t value Pr(>|t|)    
## (Intercept)  0.06284    0.10215   0.615   0.5402    
## T1.LetKenZ   0.19089    0.09018   2.117   0.0374 *  
## T1CF_TotalZ  0.52802    0.09143   5.775 1.45e-07 ***
## CondRead     0.03626    0.15742   0.230   0.8184    
## ---
## Signif. codes:  0 '***' 0.001 '**' 0.01 '*' 0.05 '.' 0.1 ' ' 1
## 
## Residual standard error: 0.6895 on 79 degrees of freedom
## Multiple R-squared:  0.4679, Adjusted R-squared:  0.4477 
## F-statistic: 23.16 on 3 and 79 DF,  p-value: 7.449e-11
```

```
## Sample size by condition
```

```
## Math Read 
##   47   36
```

```
## Trimmed observations:
```

```
## [1] 2
```

```
## Trimmed in percent:
```

```
## [1] 2.352941
```

```
##                   2.5 %    97.5 %
## (Intercept) -0.14048226 0.2661717
## T1.LetKenZ   0.01138896 0.3703962
## T1CF_TotalZ  0.34602735 0.7100189
## CondRead    -0.27708420 0.3495964
```

##### 1.4.3.4.2 PROEF Phonological Awareness

```
## Linear mixed model fit by REML. t-tests use Satterthwaite's method ['lmerModLmerTest']
## Formula: T2PF_TotalZ ~ T1PF_TotalZ + T1CF_TotalZ + Cond + (1 | Class)
##    Data: datNLpf.2
## 
## REML criterion at convergence: 166.5
## 
## Scaled residuals: 
##     Min      1Q  Median      3Q     Max 
## -2.5931 -0.6577  0.0413  0.7821  1.9531 
## 
## Random effects:
##  Groups   Name        Variance Std.Dev.
##  Class    (Intercept) 0.02245  0.1498  
##  Residual             0.40672  0.6377  
## Number of obs: 81, groups:  Class, 4
## 
## Fixed effects:
##             Estimate Std. Error       df t value Pr(>|t|)    
## (Intercept)  0.02227    0.14207  1.87792   0.157    0.891    
## T1PF_TotalZ  0.12135    0.08178 76.96299   1.484    0.142    
## T1CF_TotalZ  0.57917    0.08223 76.93570   7.044  6.9e-10 ***
## CondRead     0.06635    0.21173  2.27398   0.313    0.780    
## ---
## Signif. codes:  0 '***' 0.001 '**' 0.01 '*' 0.05 '.' 0.1 ' ' 1
## 
## Correlation of Fixed Effects:
##             (Intr) T1PF_T T1CF_T
## T1PF_TotalZ  0.074              
## T1CF_TotalZ -0.013 -0.475       
## CondRead    -0.679 -0.144  0.023
```

```
## Explained variance
```

```
##             Class   Family     Link  Marginal Conditional      AIC
## 1 lmerModLmerTest gaussian identity 0.4958194   0.5221933 167.1584
```

```
## Sample size by condition
```

```
## Math Read 
##   47   34
```

```
## Trimmed observations:
```

```
## [1] 5
```

```
## Trimmed in percent:
```

```
## [1] 5.813953
```

```
## `geom_smooth()` using formula 'y ~ x'
## `geom_smooth()` using formula 'y ~ x'
## `geom_smooth()` using formula 'y ~ x'
```

```
## `stat_bin()` using `bins = 30`. Pick better value with `binwidth`.
```

```
## `geom_smooth()` using formula 'y ~ x'
```

```
## Computing bootstrap confidence intervals ...
```

```
## 
## 384 message(s): boundary (singular) fit: see ?isSingular
```

```
##                   2.5 %    97.5 %
## .sig01       0.00000000 0.4093421
## .sigma       0.53173459 0.7371162
## (Intercept) -0.25788475 0.3067094
## T1PF_TotalZ -0.04396725 0.3011417
## T1CF_TotalZ  0.42764211 0.7538871
## CondRead    -0.34385814 0.5182438
```

#### 1.4.3.5 RAN

Rapid Automatized Naming of objects and colours at T2. Both of these models are problematic as they show a high multicollinearity of the Cond\*PreTest interaction - indicating that there is not much change from pre to post which can be attributed to condition.

##### 1.4.3.5.1 RAN Colours

```
## 
## Call:
## lm(formula = T2RANcT ~ Cond * T1RANcT, data = datNLrC.2)
## 
## Residuals:
##      Min       1Q   Median       3Q      Max 
## -13.9925  -3.9795   0.6341   3.5747  12.8613 
## 
## Coefficients:
##                  Estimate Std. Error t value Pr(>|t|)    
## (Intercept)      15.39457    4.52363   3.403  0.00106 ** 
## CondRead          1.07781    6.09448   0.177  0.86009    
## T1RANcT           0.62663    0.07676   8.163 4.93e-12 ***
## CondRead:T1RANcT -0.02757    0.10185  -0.271  0.78732    
## ---
## Signif. codes:  0 '***' 0.001 '**' 0.01 '*' 0.05 '.' 0.1 ' ' 1
## 
## Residual standard error: 5.974 on 77 degrees of freedom
## Multiple R-squared:  0.6559, Adjusted R-squared:  0.6425 
## F-statistic: 48.93 on 3 and 77 DF,  p-value: < 2.2e-16
```

```
## `geom_smooth()` using formula 'y ~ x'
## `geom_smooth()` using formula 'y ~ x'
## `geom_smooth()` using formula 'y ~ x'
```

```
## `stat_bin()` using `bins = 30`. Pick better value with `binwidth`.
```

```
##                        2.5 %     97.5 %
## (Intercept)        6.3868735 24.4022681
## CondRead         -11.0578429 13.2134596
## T1RANcT            0.4737814  0.7794834
## CondRead:T1RANcT  -0.2303855  0.1752374
```

##### 1.4.3.5.2 RAN Objects

```
## 
## Call:
## lm(formula = T2RANcT ~ Cond * T1RANcT, data = datNLrO.2)
## 
## Residuals:
##      Min       1Q   Median       3Q      Max 
## -15.4005  -4.9093   0.5972   3.1293  21.6014 
## 
## Coefficients:
##                  Estimate Std. Error t value Pr(>|t|)    
## (Intercept)       11.2436     5.9252   1.898   0.0619 .  
## CondRead           5.1870     7.8607   0.660   0.5115    
## T1RANcT            0.7193     0.1025   7.020 1.15e-09 ***
## CondRead:T1RANcT  -0.1044     0.1328  -0.786   0.4343    
## ---
## Signif. codes:  0 '***' 0.001 '**' 0.01 '*' 0.05 '.' 0.1 ' ' 1
## 
## Residual standard error: 7.348 on 70 degrees of freedom
## Multiple R-squared:  0.5943, Adjusted R-squared:  0.5769 
## F-statistic: 34.18 on 3 and 70 DF,  p-value: 1.019e-13
```

```
## `geom_smooth()` using formula 'y ~ x'
## `geom_smooth()` using formula 'y ~ x'
## `geom_smooth()` using formula 'y ~ x'
```

```
## `stat_bin()` using `bins = 30`. Pick better value with `binwidth`.
```

```
##                        2.5 %     97.5 %
## (Intercept)       -0.5737568 23.0609439
## CondRead         -10.4906007 20.8646639
## T1RANcT            0.5149382  0.9236265
## CondRead:T1RANcT  -0.3691707  0.1603897
```

#### 1.4.3.6 In-Game

Apart from the offline pencil and paper tests in 4.2.3 and 4.2.4 we also did in-game assessments in form of letter-speech-sound-identification (LSSI) and written lexical decision (WLD). Both of these feature seperate analyses for accuracy and response times.

##### 1.4.3.6.1 LSSI Accuracy

```
accLKNL <-glmer(correctSelections ~ T1CF_TotalZ + Cond * stream + Gender + lvl + trialRTs + (1+trialRTs|correctResponse) + (1+trialRTs|Subj), family=binomial, data=LKaccNL, glmerControl(optimizer="bobyqa",optCtrl=list(maxfun=50000) ))
LKaccNL.2 = LKaccNL[abs(scale(resid(accLKNL))) < 2 , ]
accLKNL.2 <-glmer(correctSelections ~ T1CF_TotalZ + Cond * stream + Gender + lvl + trialRTs + (1+trialRTs|correctResponse) + (1+trialRTs|Subj), family=binomial, data=LKaccNL.2, glmerControl(optimizer="bobyqa",optCtrl=list(maxfun=50000) ))
plot(effect("Cond*stream",accLKNL.2), ylab="probability of correct response", main="Letter Knowledge Accuracy",
     multiline=TRUE, ci.style="bars", xlab="", lines=list(col=c("black"), lty=c(2,3)),x.var="stream",
     lattice=list(key.args=list(title="Condition",x=.05, y=.95,cex=1.2,cex.title=1.2)))
```

```
summary(accLKNL.2)
```

```
## Generalized linear mixed model fit by maximum likelihood (Laplace Approximation) ['glmerMod']
##  Family: binomial  ( logit )
## Formula: correctSelections ~ T1CF_TotalZ + Cond * stream + Gender + lvl +      trialRTs + (1 + trialRTs | correctResponse) + (1 + trialRTs |      Subj)
##    Data: LKaccNL.2
## Control: glmerControl(optimizer = "bobyqa", optCtrl = list(maxfun = 50000))
## 
##      AIC      BIC   logLik deviance df.resid 
##   1638.1   1728.9   -805.0   1610.1     4849 
## 
## Scaled residuals: 
##     Min      1Q  Median      3Q     Max 
## -5.7842  0.0134  0.0436  0.1332  5.0556 
## 
## Random effects:
##  Groups          Name        Variance Std.Dev. Corr 
##  Subj            (Intercept) 3.49852  1.8704        
##                  trialRTs    0.04529  0.2128   -0.76
##  correctResponse (Intercept) 8.29981  2.8809        
##                  trialRTs    0.09177  0.3029   -0.54
## Number of obs: 4863, groups:  Subj, 75; correctResponse, 32
## 
## Fixed effects:
##                   Estimate Std. Error z value Pr(>|z|)    
## (Intercept)        4.92166    0.72330   6.804 1.01e-11 ***
## T1CF_TotalZ        0.82912    0.25532   3.247  0.00116 ** 
## CondRead          -1.11106    0.36549  -3.040  0.00237 ** 
## streamT2           2.40742    0.24199   9.948  < 2e-16 ***
## Gendermale        -1.61921    0.35255  -4.593 4.37e-06 ***
## lvlLetKenEasy      1.52361    0.22147   6.879 6.01e-12 ***
## trialRTs          -0.32342    0.08026  -4.030 5.59e-05 ***
## CondRead:streamT2 -0.40637    0.33620  -1.209  0.22677    
## ---
## Signif. codes:  0 '***' 0.001 '**' 0.01 '*' 0.05 '.' 0.1 ' ' 1
## 
## Correlation of Fixed Effects:
##             (Intr) T1CF_T CondRd strmT2 Gndrml lvlLKE trlRTs
## T1CF_TotalZ -0.223                                          
## CondRead    -0.202 -0.066                                   
## streamT2     0.007  0.062  0.085                            
## Gendermale  -0.335  0.152  0.084 -0.086                     
## lvlLetKnEsy -0.134  0.006 -0.019  0.083 -0.060              
## trialRTs    -0.540  0.021 -0.023 -0.021  0.029 -0.093       
## CndRd:strT2  0.049  0.000 -0.226 -0.637  0.017 -0.038 -0.008
```

```
writeLines('Explained variance')
```

```
## Explained variance
```

```
rsquared.glmm(list(accLKNL.2))
```

```
##      Class   Family  Link  Marginal Conditional      AIC
## 1 glmerMod binomial logit 0.2658415   0.8068688 1638.054
```

```
writeLines('Sample size by condition')
```

```
## Sample size by condition
```

```
summary(aggregate(Cond~Subj,data=accLKNL.2@frame,FUN=unique)$Cond)
```

```
## Math Read 
##   48   27
```

```
writeLines('Trimmed observations:')
```

```
## Trimmed observations:
```

```
nrow(LKaccNL[abs(scale(resid(accLKNL))) > 2 , ])
```

```
## [1] 470
```

```
writeLines('Trimmed in percent:')
```

```
## Trimmed in percent:
```

```
(1-nrow(LKaccNL[abs(scale(resid(accLKNL))) < 2 , ]) / nrow(LKaccNL)) * 100
```

```
## [1] 8.813051
```

```
(accLKNL.2bs <- confint(accLKNL.2, method = "boot", nsim = simulations, level = 0.95, parallel = bootparallel, ncpus = bootthreads))
```

```
##                         2.5 %      97.5 %
## .sig01             1.19234340  2.39135156
## .sig02            -0.95278169 -0.40591361
## .sig03             0.09770797  0.29889081
## .sig04             1.83756145  3.80710361
## .sig05            -0.79989811 -0.06947328
## .sig06             0.17859374  0.43548408
## (Intercept)        3.49035662  6.64625965
## T1CF_TotalZ        0.35344520  1.40175247
## CondRead          -1.90491248 -0.43822198
## streamT2           1.91238089  2.98846661
## Gendermale        -2.40844192 -0.90218306
## lvlLetKenEasy      1.03363459  1.97653552
## trialRTs          -0.47084218 -0.15539969
## CondRead:streamT2 -1.11882773  0.29219118
```

##### 1.4.3.6.2 LSSI Response Time

```
bc <- MASS::boxcox(lm(trialRTs ~ T1PF_TotalZ + T1AgeF + prevRT + TrialNum + stream * Cond, data=LKrtNL),plotit=FALSE)
lambda <- bc$x[which.max(bc$y)]
LKrtNL$RTinv = LKrtNL$trialRTs^lambda
LKrtNL$prevRTinv = LKrtNL$prevRT^lambda
rtLKNL = lmer(RTinv ~ T1PF_TotalZ + T1AgeF + prevRTinv + TrialNum + stream * Cond + (1+T1.LetKenZ|itemsOnScreen) + (0+stream|Subj) + (0+stream|correctResponse) + (1|Class), data=LKrtNL, control=lmerControl(optimizer = "bobyqa", optCtrl = list(maxfun=50000) ))
LKrtNL.2 = LKrtNL[abs(scale(resid(rtLKNL))) < 2 , ]
rtLKNL.2 = lmer(RTinv ~ T1PF_TotalZ + T1AgeF + prevRTinv + TrialNum + stream * Cond + (1+T1.LetKenZ|itemsOnScreen) + (0+stream|Subj) + (0+stream|correctResponse) + (1|Class), data=LKrtNL.2, control=lmerControl(optimizer = "bobyqa", optCtrl = list(maxfun=50000) ))
LKrtef = effect("stream*Cond",rtLKNL.2)
LKrtef$fit = LKrtef$fit^(1/lambda)
LKrtef$upper = LKrtef$upper^(1/lambda)
LKrtef$lower = LKrtef$lower^(1/lambda)

plot(LKrtef, ylab="Median response time (seconds)", main="Letter Knowledge Response Times", multiline=TRUE,
     ci.style="bars", xlab="Session", lines=list(col=c("black"), lty=c(2,3)),x.var="stream",
     lattice=list(key.args=list(title="Condition",x=.62, y=.95,cex=1.2,cex.title=1.2)))
```

```
pdf("Ch3_NL_LSSIrt.pdf", width=6,height=4)
plot(LKrtef, ylab="Median response time (seconds)", main="", multiline=TRUE,
     ci.style="bars", xlab="Session", lines=list(col=c("black"), lty=c(2,3)),x.var="stream",
     lattice=list(key.args=list(title="Condition",x=.62, y=.95,cex=1.2,cex.title=1.2)))
dev.off()
```

```
## quartz_off_screen 
##                 2
```

```
pdf("Ch3_NL_LSSIrtGG.pdf", width=6,height=4)
ggplot(as.data.frame(LKrtef), aes(Cond, fit, color=stream)) + geom_point() + geom_errorbar(aes(ymin=lower, ymax=upper), width=0.3) + theme_bw(base_size=12) + labs(x = "Condition", y = "Median response time (seconds)",colour="Session") + scale_colour_grey() + theme(panel.grid.major = element_blank(), axis.line = element_line(colour = "black"))
dev.off()
```

```
## quartz_off_screen 
##                 2
```

```
# compute effect size for age
summary(rtLKNL.2)
```

```
## Linear mixed model fit by REML. t-tests use Satterthwaite's method ['lmerModLmerTest']
## Formula: RTinv ~ T1PF_TotalZ + T1AgeF + prevRTinv + TrialNum + stream *      Cond + (1 + T1.LetKenZ | itemsOnScreen) + (0 + stream | Subj) +      (0 + stream | correctResponse) + (1 | Class)
##    Data: LKrtNL.2
## Control: lmerControl(optimizer = "bobyqa", optCtrl = list(maxfun = 50000))
## 
## REML criterion at convergence: -9702.5
## 
## Scaled residuals: 
##     Min      1Q  Median      3Q     Max 
## -3.6715 -0.5866  0.0451  0.6458  4.3321 
## 
## Random effects:
##  Groups          Name        Variance  Std.Dev. Corr 
##  itemsOnScreen   (Intercept) 2.174e-03 0.046631      
##                  T1.LetKenZ  1.994e-04 0.014122 -0.82
##  Subj            streamT1    6.166e-04 0.024832      
##                  streamT2    3.860e-04 0.019648 0.54 
##  correctResponse streamT1    3.821e-04 0.019547      
##                  streamT2    6.436e-04 0.025369 0.91 
##  Class           (Intercept) 4.608e-05 0.006788      
##  Residual                    3.451e-03 0.058742      
## Number of obs: 3646, groups:  itemsOnScreen, 729; Subj, 75; correctResponse, 30; Class, 4
## 
## Fixed effects:
##                     Estimate Std. Error         df t value Pr(>|t|)    
## (Intercept)        7.201e-01  5.174e-02  6.920e+01  13.918  < 2e-16 ***
## T1PF_TotalZ        9.661e-03  3.488e-03  6.814e+01   2.770  0.00722 ** 
## T1AgeF             1.811e-02  8.150e-03  6.737e+01   2.222  0.02964 *  
## prevRTinv          1.096e-02  4.622e-03  3.442e+03   2.372  0.01775 *  
## TrialNum          -3.368e-03  7.915e-04  3.041e+02  -4.255 2.79e-05 ***
## streamT2           3.519e-02  4.534e-03  6.233e+01   7.761 9.96e-11 ***
## CondRead          -5.703e-03  9.699e-03  3.071e+00  -0.588  0.59701    
## streamT2:CondRead  1.361e-02  6.895e-03  7.194e+01   1.975  0.05216 .  
## ---
## Signif. codes:  0 '***' 0.001 '**' 0.01 '*' 0.05 '.' 0.1 ' ' 1
## 
## Correlation of Fixed Effects:
##             (Intr) T1PF_T T1AgeF prvRTn TrilNm strmT2 CondRd
## T1PF_TotalZ -0.030                                          
## T1AgeF      -0.982  0.003                                   
## prevRTinv   -0.051 -0.031 -0.022                            
## TrialNum    -0.080 -0.010  0.000 -0.026                     
## streamT2    -0.030  0.003  0.000 -0.059 -0.027              
## CondRead    -0.131 -0.065  0.056  0.000 -0.002  0.245       
## strmT2:CndR  0.030 -0.003  0.001 -0.006  0.008 -0.515 -0.466
```

```
#writeLines('Explained variance')
#rsquared.glmm(list(rtLKB.2))
writeLines('Sample size by condition')
```

```
## Sample size by condition
```

```
summary(aggregate(Cond~Subj,data=rtLKNL.2@frame,FUN=unique)$Cond)
```

```
## Math Read 
##   48   27
```

```
writeLines('Trimmed observations:')
```

```
## Trimmed observations:
```

```
nrow(LKrtNL[abs(scale(resid(rtLKNL))) > 2 , ])
```

```
## [1] 212
```

```
writeLines('Trimmed in percent:')
```

```
## Trimmed in percent:
```

```
(1-nrow(LKrtNL[abs(scale(resid(rtLKNL))) < 2 , ]) / nrow(LKrtNL)) * 100
```

```
## [1] 4.945183
```

```
performance::check_model(rtLKNL.2)
```

```
acf(resid(rtLKNL.2),main="autocorrelation of observations")
```

```
(rtLKNL.2bs <- confint(rtLKNL.2, method = "boot", nsim = simulations, level = 0.95, parallel = bootparallel, ncpus = bootthreads))
```

```
##                           2.5 %       97.5 %
## .sig01             0.0319227211  0.058976568
## .sig02            -1.0000000000 -0.184127502
## .sig03             0.0031711996  0.025825506
## .sig04             0.0189560296  0.030002886
## .sig05             0.2336553559  0.781315594
## .sig06             0.0147215038  0.024330197
## .sig07             0.0136198846  0.025795823
## .sig08             0.7432755801  1.000000000
## .sig09             0.0179518590  0.033240985
## .sig10             0.0000000000  0.015090205
## .sigma             0.0571364805  0.060283589
## (Intercept)        0.6256435664  0.819885645
## T1PF_TotalZ        0.0028301959  0.016626501
## T1AgeF             0.0022217139  0.033196759
## prevRTinv          0.0015597797  0.019531984
## TrialNum          -0.0049193971 -0.001735082
## streamT2           0.0261570418  0.043518546
## CondRead          -0.0258081839  0.014071841
## streamT2:CondRead  0.0007131408  0.027173829
```

##### 1.4.3.6.3 WLD Accuracy

```
accLDNL <-glmer(correctSelections ~ Cond + (1|target) + (1|Subj), family=binomial, data=LDaccNL, glmerControl(optimizer = "bobyqa", optCtrl = list(maxfun = 50000) ))
LDaccNL.2 = LDaccNL[abs(scale(resid(accLDNL))) < 2 , ]
accLDNL.2 <-glmer(correctSelections ~ Cond + (1|target) + (1|Subj), family=binomial, data=LDaccNL.2, glmerControl(optimizer = "bobyqa", optCtrl = list(maxfun=50000) ))
plot(effect("Cond",accLDNL.2), ylab="probability of correct response", main="Lexical Decision Accuracy", multiline=TRUE, ci.style="bars", xlab="", lines=list(col=c("black"), lty=c(1,2)))
```

```
summary(accLDNL.2)
```

```
## Generalized linear mixed model fit by maximum likelihood (Laplace Approximation) ['glmerMod']
##  Family: binomial  ( logit )
## Formula: correctSelections ~ Cond + (1 | target) + (1 | Subj)
##    Data: LDaccNL.2
## Control: glmerControl(optimizer = "bobyqa", optCtrl = list(maxfun = 50000))
## 
##      AIC      BIC   logLik deviance df.resid 
##   1428.4   1450.4   -710.2   1420.4     1779 
## 
## Scaled residuals: 
##     Min      1Q  Median      3Q     Max 
## -4.1541  0.0646  0.1212  0.5248  2.2391 
## 
## Random effects:
##  Groups Name        Variance Std.Dev.
##  Subj   (Intercept) 0.404    0.6356  
##  target (Intercept) 5.598    2.3660  
## Number of obs: 1783, groups:  Subj, 75; target, 30
## 
## Fixed effects:
##             Estimate Std. Error z value Pr(>|z|)    
## (Intercept)  2.58222    0.47706   5.413 6.21e-08 ***
## CondRead    -0.02325    0.21052  -0.110    0.912    
## ---
## Signif. codes:  0 '***' 0.001 '**' 0.01 '*' 0.05 '.' 0.1 ' ' 1
## 
## Correlation of Fixed Effects:
##          (Intr)
## CondRead -0.158
```

```
writeLines('Explained variance')
```

```
## Explained variance
```

```
rsquared.glmm(list(accLDNL.2))
```

```
##      Class   Family  Link     Marginal Conditional      AIC
## 1 glmerMod binomial logit 1.332458e-05    0.645935 1428.427
```

```
writeLines('Sample size by condition')
```

```
## Sample size by condition
```

```
summary(aggregate(Cond~Subj,data=accLDNL.2@frame,FUN=unique)$Cond)
```

```
## Math Read 
##   48   27
```

```
writeLines('Trimmed observations:')
```

```
## Trimmed observations:
```

```
nrow(LDaccNL[abs(scale(resid(accLDNL))) > 2 , ])
```

```
## [1] 76
```

```
writeLines('Trimmed in percent:')
```

```
## Trimmed in percent:
```

```
(1-nrow(LDaccNL[abs(scale(resid(accLDNL))) < 2 , ]) / nrow(LDaccNL)) * 100
```

```
## [1] 4.088219
```

##### 1.4.3.6.4 WLD Response Time

```
bc <- MASS::boxcox(lm(trialRTs ~ targettype + T1.LetKenZ + prevRT + T1PF_TotalZ + Cond, data=LDrtNL),plotit=FALSE)
lambda <- bc$x[which.max(bc$y)]
if (lambda == 0) lambda <- 0.1
LDrtNL$RTinv = LDrtNL$trialRTs^lambda
LDrtNL$prevRTinv = LDrtNL$prevRT^lambda
rtLDNL = lmer(RTinv ~ targettype + T1.LetKenZ + prevRTinv + T1PF_TotalZ + Cond + (1+prevRTinv|target) + (1|Subj),data=LDrtNL)
LDrtNL.2 = LDrtNL[abs(scale(resid(rtLDNL))) < 2 , ]
rtLDNL.2 = lmer(RTinv ~ targettype + T1.LetKenZ + prevRTinv + T1PF_TotalZ + Cond + (1+prevRTinv|target) + (1|Subj),data=LDrtNL.2)
LKrtef = effect("Cond",rtLDNL.2)
LKrtef$fit = LKrtef$fit^(1/lambda)
LKrtef$upper = LKrtef$upper^(1/lambda)
LKrtef$lower = LKrtef$lower^(1/lambda)
plot(LKrtef, ylab="Median response time (seconds)", main="Lexical Decision Response Times",
     multiline=TRUE, ci.style="bars", xlab="", lines=list(col=c("black"), lty=c(1,2)))
```

```
summary(rtLDNL.2)
```

```
## Linear mixed model fit by REML. t-tests use Satterthwaite's method ['lmerModLmerTest']
## Formula: RTinv ~ targettype + T1.LetKenZ + prevRTinv + T1PF_TotalZ + Cond +      (1 + prevRTinv | target) + (1 | Subj)
##    Data: LDrtNL.2
## 
## REML criterion at convergence: -4477.5
## 
## Scaled residuals: 
##     Min      1Q  Median      3Q     Max 
## -5.0982 -0.5682 -0.0715  0.5719  4.7782 
## 
## Random effects:
##  Groups   Name        Variance Std.Dev. Corr 
##  Subj     (Intercept) 0.000795 0.02820       
##  target   (Intercept) 0.003459 0.05881       
##           prevRTinv   0.003295 0.05740  -0.94
##  Residual             0.001408 0.03752       
## Number of obs: 1285, groups:  Subj, 75; target, 29
## 
## Fixed effects:
##                  Estimate Std. Error        df t value Pr(>|t|)    
## (Intercept)      1.086226   0.018669 48.889145  58.185  < 2e-16 ***
## targettypewrong  0.016831   0.008089 27.647259   2.081 0.046840 *  
## T1.LetKenZ      -0.012869   0.006105 68.183531  -2.108 0.038729 *  
## prevRTinv        0.039916   0.015454 31.974073   2.583 0.014578 *  
## T1PF_TotalZ     -0.017019   0.004942 67.997993  -3.444 0.000987 ***
## CondRead         0.004246   0.007533 67.970149   0.564 0.574875    
## ---
## Signif. codes:  0 '***' 0.001 '**' 0.01 '*' 0.05 '.' 0.1 ' ' 1
## 
## Correlation of Fixed Effects:
##             (Intr) trgtty T1.LKZ prvRTn T1PF_T
## trgttypwrng -0.192                            
## T1.LetKenZ  -0.369 -0.003                     
## prevRTinv   -0.847 -0.017  0.009              
## T1PF_TotalZ -0.060  0.001 -0.185  0.022       
## CondRead    -0.212 -0.003  0.290 -0.013 -0.162
```

```
writeLines('Explained variance')
```

```
## Explained variance
```

```
rsquared.glmm(list(rtLDNL.2))
```

```
##             Class   Family     Link Marginal Conditional       AIC
## 1 lmerModLmerTest gaussian identity 0.113886   0.5381346 -4503.849
```

```
writeLines('Sample size by condition')
```

```
## Sample size by condition
```

```
summary(aggregate(Cond~Subj,data=rtLDNL.2@frame,FUN=unique)$Cond)
```

```
## Math Read 
##   48   27
```

```
writeLines('Trimmed observations:')
```

```
## Trimmed observations:
```

```
nrow(LDrtNL[abs(scale(resid(rtLDNL))) > 2 , ])
```

```
## [1] 67
```

```
writeLines('Trimmed in percent:')
```

```
## Trimmed in percent:
```

```
(1-nrow(LDrtNL[abs(scale(resid(rtLDNL))) < 2 , ]) / nrow(LDrtNL)) * 100
```

```
## [1] 4.820144
```

```
performance::check_model(rtLDNL)
```

```
acf(resid(rtLDNL.2),main="autocorrelation of observations")
```

```
(rtLDNL.2bs <- confint(rtLDNL.2, method = "boot", nsim = simulations, level = 0.95, parallel = bootparallel, ncpus = bootthreads))
```

```
##                        2.5 %        97.5 %
## .sig01           0.022947743  0.0337612316
## .sig02           0.013664174  0.0885066014
## .sig03          -0.985943981 -0.6127743629
## .sig04           0.019835472  0.0854363556
## .sigma           0.036041139  0.0390562154
## (Intercept)      1.043941424  1.1246766009
## targettypewrong  0.001115962  0.0332729634
## T1.LetKenZ      -0.024563366 -0.0003043497
## prevRTinv        0.009077692  0.0748518071
## T1PF_TotalZ     -0.026749376 -0.0070821460
## CondRead        -0.009408172  0.0190288079
```

### 1.4.4 ANALYSIS 3 - Game Exposure

Here, we also include subjects which were excluded for other analysis (e.g. not playing enough, or repeating first grade). The variables **hoursPlayed, levelsPlayed, sessionsPlayed, MaxLevel, itemsSeen, responsesGiven** are combined by principal component analyses.

#### 1.4.4.1 Descriptives

Here, we show a summary of the combined data with number of observations, means, range and standard deviation per variable and group.

```
tab <- tableby(Cond ~ EMT + FamRisk + Country + T1AgeF + HandScore + Gender + Lang + SON_1st + hoursPlayed + levelsPlayed + sessionsPlayed + MaxLevel + itemsSeen + responsesGiven + T1CF_TotalPc + T1PF_TotalPc + T1.LetKen + T1RANcT + T1RANoT, data = datExposure, numeric.stats=c("meansd","medianrange"))
summary(tab, text=TRUE, digits=2)
```

```
## 
## 
## |                  |         Read (N=104)         |        Math (N=106)         |        Total (N=210)        | p value|
## |:-----------------|:----------------------------:|:---------------------------:|:---------------------------:|-------:|
## |EMT               |                              |                             |                             |   0.137|
## |-  Mean (SD)      |         0.01 (0.93)          |         0.22 (1.15)         |         0.12 (1.05)         |        |
## |-  Median (Range) |     -0.19 (-1.44, 3.00)      |     -0.12 (-1.49, 3.00)     |     -0.19 (-1.49, 3.00)     |        |
## |FamRisk           |                              |                             |                             |   0.902|
## |-  no             |          87 (83.7%)          |         88 (83.0%)          |         175 (83.3%)         |        |
## |-  yes            |          17 (16.3%)          |         18 (17.0%)          |         35 (16.7%)          |        |
## |Country           |                              |                             |                             |   0.559|
## |-  B              |          63 (60.6%)          |         60 (56.6%)          |         123 (58.6%)         |        |
## |-  NL             |          41 (39.4%)          |         46 (43.4%)          |         87 (41.4%)          |        |
## |T1AgeF            |                              |                             |                             |   0.468|
## |-  Mean (SD)      |         6.25 (0.34)          |         6.28 (0.30)         |         6.26 (0.32)         |        |
## |-  Median (Range) |      6.25 (5.46, 7.39)       |      6.32 (5.55, 7.07)      |      6.30 (5.46, 7.39)      |        |
## |HandScore         |                              |                             |                             |   0.343|
## |-  Mean (SD)      |         0.03 (0.91)          |         0.14 (0.84)         |         0.08 (0.88)         |        |
## |-  Median (Range) |      0.35 (-3.20, 0.52)      |     0.35 (-3.20, 0.52)      |     0.35 (-3.20, 0.52)      |        |
## |Gender            |                              |                             |                             |   0.169|
## |-  female         |          50 (48.1%)          |         41 (38.7%)          |         91 (43.3%)          |        |
## |-  male           |          54 (51.9%)          |         65 (61.3%)          |         119 (56.7%)         |        |
## |Lang              |                              |                             |                             |   0.432|
## |-  mono           |          93 (89.4%)          |         91 (85.8%)          |         184 (87.6%)         |        |
## |-  multi          |          11 (10.6%)          |         15 (14.2%)          |         26 (12.4%)          |        |
## |SON_1st           |                              |                             |                             |   0.825|
## |-  Mean (SD)      |         -0.02 (1.03)         |         0.02 (0.97)         |        -0.00 (1.00)         |        |
## |-  Median (Range) |     -0.02 (-2.47, 4.87)      |     -0.02 (-2.47, 3.24)     |     -0.02 (-2.47, 4.87)     |        |
## |hoursPlayed       |                              |                             |                             |   0.678|
## |-  Mean (SD)      |         3.35 (1.01)          |         3.30 (0.93)         |         3.33 (0.97)         |        |
## |-  Median (Range) |      3.41 (1.16, 5.23)       |      3.07 (1.59, 5.24)      |      3.25 (1.16, 5.24)      |        |
## |levelsPlayed      |                              |                             |                             | < 0.001|
## |-  Mean (SD)      |        220.70 (69.38)        |       393.51 (157.29)       |       307.93 (149.34)       |        |
## |-  Median (Range) |    219.00 (87.00, 377.00)    |  349.50 (132.00, 1026.00)   |   275.50 (87.00, 1026.00)   |        |
## |sessionsPlayed    |                              |                             |                             |   0.937|
## |-  Mean (SD)      |         26.26 (6.46)         |        26.20 (4.66)         |        26.23 (5.61)         |        |
## |-  Median (Range) |     26.00 (11.00, 37.00)     |    26.00 (14.00, 35.00)     |    26.00 (11.00, 37.00)     |        |
## |MaxLevel          |                              |                             |                             |   0.012|
## |-  Mean (SD)      |        117.86 (59.56)        |       134.43 (30.58)        |       126.22 (47.82)        |        |
## |-  Median (Range) |    96.50 (24.00, 265.00)     |   136.00 (39.00, 178.00)    |   132.00 (24.00, 265.00)    |        |
## |itemsSeen         |                              |                             |                             |   0.008|
## |-  Mean (SD)      |      10219.71 (4292.51)      |      8649.07 (4245.75)      |      9426.91 (4330.88)      |        |
## |-  Median (Range) | 10090.00 (2010.00, 20113.00) | 7485.00 (2827.00, 22782.00) | 8448.00 (2010.00, 22782.00) |        |
## |responsesGiven    |                              |                             |                             | < 0.001|
## |-  Mean (SD)      |       2480.16 (918.20)       |      3718.32 (2074.57)      |      3105.14 (1721.27)      |        |
## |-  Median (Range) |  2486.50 (628.00, 4522.00)   | 3171.00 (1019.00, 10877.00) | 2850.00 (628.00, 10877.00)  |        |
## |T1CF_TotalPc      |                              |                             |                             |   0.382|
## |-  Mean (SD)      |        44.21 (25.62)         |        47.26 (24.96)        |        45.75 (25.27)        |        |
## |-  Median (Range) |     37.00 (0.10, 99.00)      |     50.00 (1.00, 91.00)     |     37.00 (0.10, 99.00)     |        |
## |T1PF_TotalPc      |                              |                             |                             |   0.802|
## |-  Mean (SD)      |        53.96 (28.82)         |        52.95 (29.14)        |        53.44 (28.91)        |        |
## |-  Median (Range) |     55.00 (0.00, 100.00)     |    55.00 (0.00, 100.00)     |    55.00 (0.00, 100.00)     |        |
## |T1.LetKen         |                              |                             |                             |   0.147|
## |-  Mean (SD)      |         14.12 (9.33)         |        16.00 (9.42)         |        15.07 (9.40)         |        |
## |-  Median (Range) |     13.00 (0.00, 30.00)      |     15.00 (0.00, 30.00)     |     15.00 (0.00, 30.00)     |        |
## |T1RANcT           |                              |                             |                             |   0.050|
## |-  Mean (SD)      |        68.71 (16.76)         |        64.41 (14.86)        |        66.54 (15.93)        |        |
## |-  Median (Range) |    66.00 (39.00, 122.00)     |    62.50 (40.00, 107.00)    |    65.00 (39.00, 122.00)    |        |
## |T1RANoT           |                              |                             |                             |   0.577|
## |-  Mean (SD)      |        76.27 (21.21)         |        74.77 (17.36)        |        75.51 (19.33)        |        |
## |-  Median (Range) |    73.00 (43.00, 180.00)     |    72.00 (40.00, 130.00)    |    72.00 (40.00, 180.00)    |        |
```

#### 1.4.4.2 PCA

Principal component analysis factor loadings and histograms for relevant variables to show data coverage.

```
writeLines('PCA component loadings of reading condition')
```

```
## PCA component loadings of reading condition
```

```
prcomp(readExposure[,12:17],scale=T)
```

```
## Standard deviations (1, .., p=6):
## [1] 2.20393928 0.83852686 0.50713198 0.37704198 0.19142493 0.05947579
## 
## Rotation (n x k) = (6 x 6):
##                      PC1         PC2        PC3         PC4         PC5         PC6
## hoursPlayed    0.3916816  0.46389432  0.3512667 -0.70978360  0.06344236  0.01347600
## levelsPlayed   0.4398018 -0.02069116  0.2489048  0.42083099  0.71758646  0.22839350
## sessionsPlayed 0.3658469  0.54475614 -0.7266712  0.19224675 -0.06471541 -0.01410298
## MaxLevel       0.3589094 -0.65066835 -0.4360174 -0.42682897  0.21663621 -0.16908380
## itemsSeen      0.4401304 -0.24352451  0.1028218  0.09566272 -0.57033493  0.63401531
## responsesGiven 0.4436048 -0.07029370  0.2931272  0.30135590 -0.32348643 -0.71895144
```

```
writeLines('PCA component loadings of math condition')
```

```
## PCA component loadings of math condition
```

```
prcomp(mathExposure[,12:17],scale=T)
```

```
## Standard deviations (1, .., p=6):
## [1] 2.10208576 0.86411560 0.68352379 0.54207365 0.26901839 0.03346937
## 
## Rotation (n x k) = (6 x 6):
##                      PC1         PC2         PC3         PC4         PC5          PC6
## hoursPlayed    0.4015318  0.15113785 -0.42408226  0.79641557 -0.03938854 -0.015956608
## levelsPlayed   0.4481709  0.16753175  0.27897981 -0.14992160 -0.81892883 -0.011195849
## sessionsPlayed 0.3873993  0.04261814 -0.71184497 -0.57817923  0.08399375  0.006004764
## MaxLevel       0.2647940 -0.96019347  0.03012305  0.06174105 -0.05226583 -0.021483786
## itemsSeen      0.4583667  0.09764005  0.33658744 -0.03704332  0.38241397  0.720737571
## responsesGiven 0.4556545  0.12557497  0.34842896 -0.06140329  0.41445989 -0.692574750
```

```
hist(datExposure$ExposurePCA,main="Exposure distribution",xlab="Exposure principal component")
```

```
hist(datExposure$T1CF_TotalZ,main="CELF PA distribution at T1",xlab="CELF PA (z-score)")
```

```
hist(datExposure$EMT,main="Reading fluency distribution at T2",xlab="One minute reading (z-score)")
```

#### 1.4.4.3 READ vs. MATH

```
RMexp <- gam(EMT ~ s(T1RANcT) + s(T1CF_TotalZ) + ti(T1CF_TotalZ, ExposurePCA, by=Cond) + Country, data=datExposure)
datExposure.2 = datExposure[abs(scale(resid(RMexp))) < 2, ]
RMexp.2 <- gam(EMT ~ s(T1RANcT) + s(T1CF_TotalZ) + ti(T1CF_TotalZ, ExposurePCA, by=Cond) + Country, data=datExposure.2)
par(mfcol=c(1,3))
par(mar=c(4,4,2,3))
fvisgam(RMexp.2,view=c("T1CF_TotalZ","ExposurePCA"), ylim=c(-2.1,2.1),zlim=c(-2.1,2.1), xlim=c(-2,2),
        n.grid=250,cond=list(Cond="Math"),
        xlab="PreTest PA (z-score)",
        ylab="Exposure PCA (z-score)", show.diff=T, col.diff="white", alpha.diff=0.7,
        main="A) Math", print.summary=F, add.color.legend = F, nCol=200, color=c("blue", "green", "red"), col=1)
fadeRug(subset(datExposure.2, Cond=="Math")$T1CF_TotalZ,subset(datExposure.2,Cond=="Math")$ExposurePCA, alpha=0.5,n.grid=1000,use.data.range=F,col="white")
box()
gradientLegend(valRange = c(-2,2), nCol=200 ,pos=0.87, side=4,color=c("blue", "green", "red"), col=1)

fvisgam(RMexp.2,view=c("T1CF_TotalZ","ExposurePCA"), ylim=c(-2.1,2.1),zlim=c(-2,2),xlim=c(-2,2),
        n.grid=250, cond=list(Cond="Read"),
        xlab="PreTest PA (z-score)", ylab="", show.diff=T,col.diff="white", alpha.diff=0.7,
        main="B) Read", print.summary=F, add.color.legend = F, nCol=200, color=c("blue", "green", "red"), col=1)
fadeRug(subset(datExposure.2,Cond=="Read")$T1CF_TotalZ,subset(datExposure.2,Cond=="Read")$ExposurePCA, alpha=0.5,n.grid=1000,use.data.range=F)
box()
gradientLegend(valRange = c(-2,2), nCol=200 ,pos=0.87, side=4, color=c("blue", "green", "red"), col=1)

plot_diff2(RMexp.2,view=c("T1CF_TotalZ","ExposurePCA"), ylim=c(-2.1,2.1),zlim=c(-1,1),xlim=c(-2,2),
        n.grid=250, comp=list(Cond=c("Read","Math")),
        xlab="PreTest PA (z-score)",
        ylab="",nCol=200,nlevels=4, show.diff=T,col.diff="white", alpha.diff=0.7,
        main="C) B-A Difference", print.summary=F, add.color.legend = F, color=c("blue", "green", "red"), col=1)
fadeRug(datExposure.2$T1CF_TotalZ,datExposure.2$ExposurePCA,alpha=0.5,n.grid=1000, use.data.range=F)
box()
gradientLegend(valRange = c(-1,1), nCol=200 ,pos=0.87, side=4, color=c("blue", "green", "red"), col=1)
```

```
par(mfcol=c(1,1))
summary(RMexp.2)
```

```
## 
## Family: gaussian 
## Link function: identity 
## 
## Formula:
## EMT ~ s(T1RANcT) + s(T1CF_TotalZ) + ti(T1CF_TotalZ, ExposurePCA, 
##     by = Cond) + Country
## 
## Parametric coefficients:
##             Estimate Std. Error t value Pr(>|t|)   
## (Intercept) -0.16331    0.05817  -2.808  0.00552 **
## CountryNL    0.17823    0.10190   1.749  0.08193 . 
## ---
## Signif. codes:  0 '***' 0.001 '**' 0.01 '*' 0.05 '.' 0.1 ' ' 1
## 
## Approximate significance of smooth terms:
##                                        edf Ref.df      F p-value    
## s(T1RANcT)                           1.845  2.327  4.535 0.00932 ** 
## s(T1CF_TotalZ)                       1.000  1.000 43.138 < 2e-16 ***
## ti(T1CF_TotalZ,ExposurePCA):CondRead 4.649  5.946  2.987 0.00883 ** 
## ti(T1CF_TotalZ,ExposurePCA):CondMath 1.000  1.000  0.203 0.65315    
## ---
## Signif. codes:  0 '***' 0.001 '**' 0.01 '*' 0.05 '.' 0.1 ' ' 1
## 
## R-sq.(adj) =  0.473   Deviance explained = 49.8%
## GCV = 0.36377  Scale est. = 0.34429   n = 196
```

```
writeLines('Sample size by condition')
```

```
## Sample size by condition
```

```
aggregate(EMT~Cond,RMexp.2$model,FUN=NROW)
```

```
##   Cond EMT
## 1 Read 101
## 2 Math  95
```

```
writeLines('Trimmed observations:')
```

```
## Trimmed observations:
```

```
nrow(datExposure[abs(scale(resid(RMexp))) > 2 , ])
```

```
## [1] 14
```

```
writeLines('Trimmed in percent:')
```

```
## Trimmed in percent:
```

```
(1-nrow(datExposure[abs(scale(resid(RMexp))) < 2 , ]) / nrow(datExposure)) * 100
```

```
## [1] 6.666667
```

```
gam.check(RMexp.2)
```

```
## 
## Method: GCV   Optimizer: magic
## Smoothing parameter selection converged after 18 iterations.
## The RMS GCV score gradient at convergence was 5.126103e-08 .
## The Hessian was positive definite.
## Model rank =  52 / 52 
## 
## Basis dimension (k) checking results. Low p-value (k-index<1) may
## indicate that k is too low, especially if edf is close to k'.
## 
##                                         k'   edf k-index p-value
## s(T1RANcT)                            9.00  1.84    1.03    0.69
## s(T1CF_TotalZ)                        9.00  1.00    0.94    0.23
## ti(T1CF_TotalZ,ExposurePCA):CondRead 16.00  4.65    1.06    0.78
## ti(T1CF_TotalZ,ExposurePCA):CondMath 16.00  1.00    1.04    0.74
```

```
# also plot individual smooths
plot(RMexp.2,select=1, ylab="Reading fluency (z-score)")
abline(0,0)
```

```
plot(RMexp.2,select=2, ylab="Reading fluency (z-score)")
abline(0,0)
```

#### 1.4.4.4 READ by Gender

```
RGexp <- gam(EMT ~ s(T1CF_TotalZ) + ti(T1CF_TotalZ, ExposurePCA, by=Gender), data=readExposure)
readExposure.2 = readExposure[abs(scale(resid(RGexp))) < 2, ]
RGexp.2 <- gam(EMT ~ s(T1CF_TotalZ) + ti(T1CF_TotalZ, ExposurePCA, by=Gender), data=readExposure.2)

par(mfcol=c(1,3))
fvisgam(RGexp.2,view=c("T1CF_TotalZ","ExposurePCA"), ylim=c(-2,2),zlim=c(-2,2),xlim=c(-2,2),
        n.grid=250,cond=list(Gender="male"),
        xlab="Phonological skills at pre-test(z-score)",
        ylab="Exposure PCA (z-score)",show.diff=T,col.diff="white", alpha.diff=0.7,
        main="D) Read: Male", print.summary=F,add.color.legend = F, nCol=200)
fadeRug(subset(readExposure.2, Gender=="male")$T1CF_TotalZ,subset(readExposure.2,Gender=="male")$ExposurePCA,
        alpha=0.75,n.grid=1000,use.data.range=F)
box()
gradientLegend(valRange = c(-2,2), nCol=200 ,pos=0.87, side=4)

fvisgam(RGexp.2,view=c("T1CF_TotalZ","ExposurePCA"), ylim=c(-2,2),zlim=c(-2,2),xlim=c(-2,2),
        n.grid=250,cond=list(Gender="female"),
        xlab="Phonological skills at pre-test(z-score)",
        ylab="",show.diff=T,col.diff="white", alpha.diff=0.7,
        main="E) Read: Female", print.summary=F, add.color.legend = F, nCol=200)
fadeRug(subset(readExposure.2,Gender=="female")$T1CF_TotalZ,subset(readExposure.2,Gender=="female")$ExposurePCA,alpha=0.75,n.grid=1000,use.data.range=F)
box()
gradientLegend(valRange = c(-2,2), nCol=200 ,pos=0.87, side=4)

plot_diff2(RGexp.2,view=c("T1CF_TotalZ","ExposurePCA"), ylim=c(-2,2), zlim=c(-2,2), xlim=c(-2,2),
        n.grid=250, comp=list(Gender=c("female","male")),
        xlab="Phonological skills at pre-test(z-score)",
        ylab="",nCol=200, nlevels=8,show.diff=T,col.diff="white", alpha.diff=0.7,
        main="F) E-D Difference", print.summary=F, add.color.legend = F,se=F)
fadeRug(readExposure.2$T1CF_TotalZ,readExposure.2$ExposurePCA,alpha=0.75,n.grid=1000,use.data.range=F, col="white")
box()
gradientLegend(valRange = c(-2,2), nCol=200 ,pos=0.87, side=4)
```

```
par(mfcol=c(1,1))
summary(RGexp.2)
```

```
## 
## Family: gaussian 
## Link function: identity 
## 
## Formula:
## EMT ~ s(T1CF_TotalZ) + ti(T1CF_TotalZ, ExposurePCA, by = Gender)
## 
## Parametric coefficients:
##             Estimate Std. Error t value Pr(>|t|)    
## (Intercept) -0.19958    0.05703  -3.499 0.000789 ***
## ---
## Signif. codes:  0 '***' 0.001 '**' 0.01 '*' 0.05 '.' 0.1 ' ' 1
## 
## Approximate significance of smooth terms:
##                                             edf Ref.df      F p-value    
## s(T1CF_TotalZ)                            8.396  8.830 10.765 < 2e-16 ***
## ti(T1CF_TotalZ,ExposurePCA):Genderfemale 10.077 11.630  8.281 < 2e-16 ***
## ti(T1CF_TotalZ,ExposurePCA):Gendermale    3.791  3.963  6.272 0.00027 ***
## ---
## Signif. codes:  0 '***' 0.001 '**' 0.01 '*' 0.05 '.' 0.1 ' ' 1
## 
## R-sq.(adj) =  0.742   Deviance explained = 80.1%
## GCV = 0.23032  Scale est. = 0.17564   n = 98
```

```
writeLines('Sample size by condition')
```

```
## Sample size by condition
```

```
aggregate(EMT~Gender,RGexp.2$model,FUN=NROW)
```

```
##   Gender EMT
## 1 female  47
## 2   male  51
```

```
writeLines('Trimmed observations:')
```

```
## Trimmed observations:
```

```
nrow(readExposure[abs(scale(resid(RGexp))) > 2 , ])
```

```
## [1] 6
```

```
writeLines('Trimmed in percent:')
```

```
## Trimmed in percent:
```

```
(1-nrow(readExposure[abs(scale(resid(RGexp))) < 2 , ]) / nrow(readExposure)) * 100
```

```
## [1] 5.769231
```

```
gam.check(RGexp.2)
```

```
## 
## Method: GCV   Optimizer: magic
## Smoothing parameter selection converged after 16 iterations.
## The RMS GCV score gradient at convergence was 5.461778e-08 .
## The Hessian was positive definite.
## Model rank =  42 / 42 
## 
## Basis dimension (k) checking results. Low p-value (k-index<1) may
## indicate that k is too low, especially if edf is close to k'.
## 
##                                             k'   edf k-index p-value
## s(T1CF_TotalZ)                            9.00  8.40    0.99    0.41
## ti(T1CF_TotalZ,ExposurePCA):Genderfemale 16.00 10.08    1.19    0.98
## ti(T1CF_TotalZ,ExposurePCA):Gendermale   16.00  3.79    1.04    0.65
```

```
plot(RGexp.2,select=1, ylab="Reading fluency (z-score)")
abline(0,0)
```

```
gc()
```

```
##            used  (Mb) gc trigger  (Mb) limit (Mb) max used  (Mb)
## Ncells  3059382 163.4    4742435 253.3         NA  4742435 253.3
## Vcells 27392077 209.0   53101616 405.2      73728 82970076 633.1
```

## 1.5 Extra Analyses

### 1.5.1 LK Belgium

This analysis is not shown in the manuscript but presents a conventional approach to evaluate potential changes in letter knowledge with an ANOVA on the aggregated in-game assessment. Here, we only find a main effect of time which is confirmed by a post-hoc wilcoxon test, showing an overall increase form T1 to T2.

```
LKdatB = subset(datB, T2.LetKen >0 & T1.LetKen >0, select=c("Subj","Cond","T1.LetKen","T2.LetKen"))
LKdatB = melt(LKdatB,id.vars=c("Subj","Cond"))
LKdatB$variable <- sub(".LetKen","",LKdatB$variable)
names(LKdatB) <- c("Subj","Cond","Session","LettersCorrect")
LKB = aov(LettersCorrect~Cond*Session,LKdatB) # main effect of session
summary(LKB)
```

```
##               Df Sum Sq Mean Sq F value Pr(>F)    
## Cond           2     19      10   0.265  0.767    
## Session        1   6398    6398 177.251 <2e-16 ***
## Cond:Session   2    161      80   2.227  0.111    
## Residuals    200   7219      36                   
## ---
## Signif. codes:  0 '***' 0.001 '**' 0.01 '*' 0.05 '.' 0.1 ' ' 1
```

```
shapiro.test(LKdatB$LettersCorrect) # not normal, so wilcox.test
```

```
## 
##  Shapiro-Wilk normality test
## 
## data:  LKdatB$LettersCorrect
## W = 0.95436, p-value = 3.742e-06
```

```
hist(LKdatB$LettersCorrect)
```

```
boxplot(LKdatB$LettersCorrect~LKdatB$Cond+LKdatB$Session)
```

```
wilcox.test(LettersCorrect~Session, data=LKdatB, paired=T) # T2 > T1
```

```
## 
##  Wilcoxon signed rank test with continuity correction
## 
## data:  LettersCorrect by Session
## V = 83.5, p-value < 2.2e-16
## alternative hypothesis: true location shift is not equal to 0
```

### 1.5.2 LK Netherlands

This analysis is not shown in the manuscript but presents a conventional approach to evaluate potential changes in letter knowledge with an ANOVA on the aggregated in-game assessment. Here, we only find a main effect of Time and a main effect of Condition

```
LKdatNL = subset(datNL, T2.LetKen >0 & T1.LetKen >0, select=c("Subj","Cond","T1.LetKen","T2.LetKen"))
LKdatNL = melt(LKdatNL,id.vars=c("Subj","Cond"))
LKdatNL$variable <- sub(".LetKen","",LKdatNL$variable)
names(LKdatNL) <- c("Subj","Cond","Session","LettersCorrect")
LKNL = aov(LettersCorrect~Cond*Session,LKdatNL) # main effect of session and main effect of condition
summary(LKNL)
```

```
##               Df Sum Sq Mean Sq F value   Pr(>F)    
## Cond           1    326   326.1  13.706 0.000302 ***
## Session        1    561   560.7  23.564 3.07e-06 ***
## Cond:Session   1      1     0.6   0.024 0.878262    
## Residuals    146   3474    23.8                     
## ---
## Signif. codes:  0 '***' 0.001 '**' 0.01 '*' 0.05 '.' 0.1 ' ' 1
```

```
shapiro.test(LKdatNL$LettersCorrect) # not normal
```

```
## 
##  Shapiro-Wilk normality test
## 
## data:  LKdatNL$LettersCorrect
## W = 0.89015, p-value = 3.816e-09
```

```
hist(LKdatNL$LettersCorrect)
```

```
boxplot(LKdatNL$LettersCorrect~LKdatNL$Cond+LKdatNL$Session)
```

```
wilcox.test(LettersCorrect~Session, data=LKdatNL, paired=T) # T2 > T1
```

```
## 
##  Wilcoxon signed rank test with continuity correction
## 
## data:  LettersCorrect by Session
## V = 297.5, p-value = 5.109e-08
## alternative hypothesis: true location shift is not equal to 0
```

```
wilcox.test(LettersCorrect~Cond, data=LKdatNL) # Math > Read
```

```
## 
##  Wilcoxon rank sum test with continuity correction
## 
## data:  LettersCorrect by Cond
## W = 3363.5, p-value = 0.002452
## alternative hypothesis: true location shift is not equal to 0
```

### 1.5.3 Raw PrePost

Here, we present the raw means for the outcome measures at both testing points

```
datB$EMTraw <- (datB$T2EMTaCor + datB$T2EMTbCor) / 2

tab <- tableby(Cond ~ EMTraw + T1CF_Total + T2CF_Total + T1CF_TotalPc + T2CF_TotalPc + T1PF_Total + T2PF_Total + T1PF_TotalPc + T2PF_TotalPc + T1.LetKen + T2.LetKen + T1RANcT + T2RANcT + T1RANoT +T2RANoT, data = datB, numeric.stats=c("meansd","medianrange"))
summary(tab, text=TRUE, digits=2, test=F)
```

```
## 
## 
## |                  |      Math (N=56)      |    Passive (N=52)     |      Read (N=53)      |     Total (N=161)     |
## |:-----------------|:---------------------:|:---------------------:|:---------------------:|:---------------------:|
## |EMTraw            |                       |                       |                       |                       |
## |-  Mean (SD)      |     12.02 (7.79)      |     10.05 (4.59)      |     12.62 (7.43)      |     11.58 (6.83)      |
## |-  Median (Range) |  10.00 (1.00, 41.50)  |  10.50 (2.50, 22.00)  |  12.50 (1.00, 32.50)  |  10.50 (1.00, 41.50)  |
## |T1CF_Total        |                       |                       |                       |                       |
## |-  Mean (SD)      |     23.44 (9.64)      |     23.38 (9.84)      |     22.08 (8.27)      |     22.96 (9.23)      |
## |-  Median (Range) |  26.00 (1.00, 40.00)  |  24.50 (1.00, 42.00)  |  23.00 (2.00, 39.00)  |  24.00 (1.00, 42.00)  |
## |T2CF_Total        |                       |                       |                       |                       |
## |-  Mean (SD)      |     32.04 (7.67)      |     29.58 (8.64)      |     30.45 (6.39)      |     30.72 (7.64)      |
## |-  Median (Range) | 33.00 (13.00, 44.00)  |  31.00 (2.00, 44.00)  |  31.00 (8.00, 43.00)  |  31.00 (2.00, 44.00)  |
## |T1CF_TotalPc      |                       |                       |                       |                       |
## |-  Mean (SD)      |     38.22 (23.68)     |     41.14 (23.91)     |     35.74 (20.05)     |     38.31 (22.56)     |
## |-  Median (Range) |  37.00 (1.00, 91.00)  |  37.00 (1.00, 95.00)  |  37.00 (5.00, 84.00)  |  37.00 (1.00, 95.00)  |
## |T2CF_TotalPc      |                       |                       |                       |                       |
## |-  Mean (SD)      |     55.71 (25.32)     |     50.50 (24.37)     |     50.87 (20.49)     |     52.44 (23.49)     |
## |-  Median (Range) |  56.50 (9.00, 98.00)  |  50.00 (0.10, 99.00)  |  50.00 (9.00, 95.00)  |  50.00 (0.10, 99.00)  |
## |T1PF_Total        |                       |                       |                       |                       |
## |-  Mean (SD)      |     29.93 (5.66)      |     30.21 (5.33)      |     29.23 (5.79)      |     29.79 (5.58)      |
## |-  Median (Range) | 30.00 (16.00, 40.00)  | 31.00 (11.00, 38.00)  |  30.00 (0.00, 37.00)  |  30.00 (0.00, 40.00)  |
## |T2PF_Total        |                       |                       |                       |                       |
## |-  Mean (SD)      |     33.75 (4.50)      |     32.50 (5.36)      |     31.60 (4.65)      |     32.64 (4.89)      |
## |-  Median (Range) | 34.00 (18.00, 40.00)  | 33.00 (13.00, 40.00)  | 32.00 (15.00, 40.00)  | 33.00 (13.00, 40.00)  |
## |T1PF_TotalPc      |                       |                       |                       |                       |
## |-  Mean (SD)      |     45.67 (29.28)     |     49.52 (25.94)     |     44.29 (25.37)     |     46.46 (26.89)     |
## |-  Median (Range) | 45.00 (0.00, 100.00)  |  47.50 (0.00, 97.50)  |  42.50 (0.00, 97.50)  | 42.50 (0.00, 100.00)  |
## |T2PF_TotalPc      |                       |                       |                       |                       |
## |-  Mean (SD)      |     61.03 (26.92)     |     55.91 (29.02)     |     48.73 (24.33)     |     55.33 (27.13)     |
## |-  Median (Range) | 55.00 (2.50, 100.00)  | 55.00 (0.00, 100.00)  | 47.50 (0.00, 100.00)  | 55.00 (0.00, 100.00)  |
## |T1.LetKen         |                       |                       |                       |                       |
## |-  Mean (SD)      |      9.36 (6.42)      |     11.27 (5.30)      |      8.23 (6.70)      |      9.59 (6.27)      |
## |-  Median (Range) |  8.00 (0.00, 26.00)   |  10.00 (0.00, 25.00)  |  6.00 (0.00, 25.00)   |  8.00 (0.00, 26.00)   |
## |T2.LetKen         |                       |                       |                       |                       |
## |-  Mean (SD)      |     20.78 (5.97)      |     21.32 (4.92)      |     22.58 (6.84)      |     21.59 (5.79)      |
## |-  Median (Range) |  21.00 (8.00, 30.00)  |  21.00 (8.00, 32.00)  |  25.00 (3.00, 30.00)  |  22.00 (3.00, 32.00)  |
## |T1RANcT           |                       |                       |                       |                       |
## |-  Mean (SD)      |     67.50 (15.37)     |     70.88 (15.92)     |     74.96 (16.02)     |     71.05 (15.97)     |
## |-  Median (Range) | 67.00 (40.00, 107.00) | 67.50 (48.00, 115.00) | 70.00 (49.00, 122.00) | 69.00 (40.00, 122.00) |
## |T2RANcT           |                       |                       |                       |                       |
## |-  Mean (SD)      |     55.88 (11.42)     |     57.37 (12.57)     |     59.40 (14.01)     |     57.51 (12.68)     |
## |-  Median (Range) | 55.00 (36.00, 78.00)  | 56.00 (37.00, 103.00) | 58.00 (35.00, 93.00)  | 56.00 (35.00, 103.00) |
## |T1RANoT           |                       |                       |                       |                       |
## |-  Mean (SD)      |     76.25 (17.33)     |     75.56 (17.49)     |     81.77 (23.82)     |     77.86 (19.83)     |
## |-  Median (Range) | 72.00 (50.00, 120.00) | 76.50 (46.00, 127.00) | 76.00 (49.00, 180.00) | 76.00 (46.00, 180.00) |
## |T2RANoT           |                       |                       |                       |                       |
## |-  Mean (SD)      |     69.93 (21.50)     |     68.08 (19.37)     |     73.55 (22.18)     |     70.52 (21.06)     |
## |-  Median (Range) | 67.50 (43.00, 186.00) | 64.00 (40.00, 134.00) | 69.00 (41.00, 177.00) | 66.00 (40.00, 186.00) |
```

```
datNL$EMTraw <- (datNL$T2EMTaCor + datNL$T2EMTbCor) / 2

tab <- tableby(Cond ~ EMTraw + T1CF_Total + T2CF_Total + T1CF_TotalPc + T2CF_TotalPc + T1PF_Total + T2PF_Total + T1PF_TotalPc + T2PF_TotalPc + T1.LetKen + T2.LetKen + T1RANcT + T2RANcT + T1RANoT +T2RANoT, data = datNL, numeric.stats=c("meansd","medianrange"))
summary(tab, text=TRUE, digits=2, test=F)
```

```
## 
## 
## |                  |      Math (N=48)      |      Read (N=38)      |     Total (N=86)      |
## |:-----------------|:---------------------:|:---------------------:|:---------------------:|
## |EMTraw            |                       |                       |                       |
## |-  Mean (SD)      |     23.78 (11.59)     |     19.30 (10.19)     |     21.76 (11.14)     |
## |-  Median (Range) |  19.50 (3.50, 45.00)  |  16.50 (8.00, 45.00)  |  18.00 (3.50, 45.00)  |
## |T1CF_Total        |                       |                       |                       |
## |-  Mean (SD)      |     31.54 (6.71)      |     32.53 (6.64)      |     31.98 (6.66)      |
## |-  Median (Range) |  32.00 (8.00, 41.00)  | 32.50 (19.00, 44.00)  |  32.00 (8.00, 44.00)  |
## |T2CF_Total        |                       |                       |                       |
## |-  Mean (SD)      |     36.73 (4.68)      |     36.49 (4.95)      |     36.62 (4.77)      |
## |-  Median (Range) | 38.00 (25.00, 44.00)  | 36.00 (23.00, 44.00)  | 37.00 (23.00, 44.00)  |
## |T1CF_TotalPc      |                       |                       |                       |
## |-  Mean (SD)      |     59.85 (19.35)     |     63.68 (22.22)     |     61.55 (20.63)     |
## |-  Median (Range) |  63.00 (9.00, 91.00)  | 56.50 (25.00, 99.00)  |  63.00 (9.00, 99.00)  |
## |T2CF_TotalPc      |                       |                       |                       |
## |-  Mean (SD)      |     72.50 (19.26)     |     73.46 (17.64)     |     72.92 (18.47)     |
## |-  Median (Range) | 75.00 (25.00, 95.00)  | 75.00 (25.00, 98.00)  | 75.00 (25.00, 98.00)  |
## |T1PF_Total        |                       |                       |                       |
## |-  Mean (SD)      |     33.17 (4.26)      |     34.76 (3.51)      |     33.87 (4.01)      |
## |-  Median (Range) | 33.50 (21.00, 40.00)  | 35.00 (27.00, 40.00)  | 35.00 (21.00, 40.00)  |
## |T2PF_Total        |                       |                       |                       |
## |-  Mean (SD)      |     35.88 (2.76)      |     36.00 (3.43)      |     35.93 (3.06)      |
## |-  Median (Range) | 36.00 (29.00, 40.00)  | 37.00 (28.00, 40.00)  | 36.00 (28.00, 40.00)  |
## |T1PF_TotalPc      |                       |                       |                       |
## |-  Mean (SD)      |     64.84 (26.55)     |     72.50 (22.80)     |     68.23 (25.11)     |
## |-  Median (Range) | 66.25 (7.50, 100.00)  | 75.00 (22.50, 100.00) | 75.00 (7.50, 100.00)  |
## |T2PF_TotalPc      |                       |                       |                       |
## |-  Mean (SD)      |     73.80 (19.63)     |     75.92 (22.75)     |     74.74 (20.97)     |
## |-  Median (Range) | 75.00 (32.50, 100.00) | 82.50 (25.00, 100.00) | 75.00 (25.00, 100.00) |
## |T1.LetKen         |                       |                       |                       |
## |-  Mean (SD)      |     24.50 (4.74)      |     22.24 (6.36)      |     23.50 (5.60)      |
## |-  Median (Range) | 26.00 (13.00, 30.00)  |  24.00 (2.00, 30.00)  |  25.00 (2.00, 30.00)  |
## |T2.LetKen         |                       |                       |                       |
## |-  Mean (SD)      |     28.46 (3.45)      |     25.26 (6.12)      |     27.31 (4.81)      |
## |-  Median (Range) | 29.00 (15.00, 32.00)  | 27.00 (10.00, 32.00)  | 29.00 (10.00, 32.00)  |
## |T1RANcT           |                       |                       |                       |
## |-  Mean (SD)      |     58.44 (11.80)     |     59.39 (14.71)     |     58.86 (13.09)     |
## |-  Median (Range) | 57.50 (41.00, 96.00)  | 57.50 (39.00, 102.00) | 57.50 (39.00, 102.00) |
## |T2RANcT           |                       |                       |                       |
## |-  Mean (SD)      |     52.46 (10.17)     |     53.11 (11.87)     |     52.74 (10.89)     |
## |-  Median (Range) | 49.00 (39.00, 81.00)  | 50.00 (34.00, 85.00)  | 49.00 (34.00, 85.00)  |
## |T1RANoT           |                       |                       |                       |
## |-  Mean (SD)      |     70.46 (15.11)     |     65.68 (12.13)     |     68.35 (14.00)     |
## |-  Median (Range) | 68.00 (40.00, 126.00) | 65.50 (43.00, 90.00)  | 67.00 (40.00, 126.00) |
## |T2RANoT           |                       |                       |                       |
## |-  Mean (SD)      |     66.54 (17.76)     |     63.67 (15.70)     |     65.31 (16.87)     |
## |-  Median (Range) | 64.00 (39.00, 120.00) | 63.00 (39.00, 111.00) | 63.00 (39.00, 120.00) |
```
